# An NHC-Catalyzed Desulfonylative Smiles Rearrangement of Pyrrole and Indole Carboxaldehydes

**DOI:** 10.1021/acs.joc.3c01089

**Published:** 2023-08-17

**Authors:** Caitlin Swaby, Alfie Taylor, Michael F. Greaney

**Affiliations:** Dept. of Chemistry, University of Manchester, Oxford Rd, Manchester, M13 9PL, U.K.

## Abstract

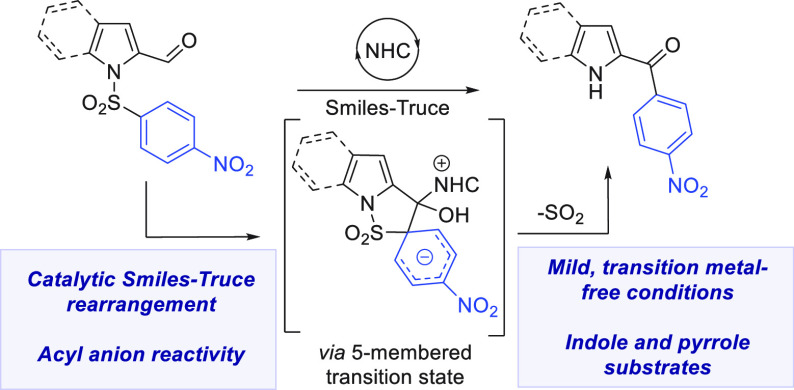

The use of catalysis methods to enable Smiles rearrangement opens up new substrate classes for arylation under mild conditions. Here, we describe an N-heterocyclic carbene (NHC) catalysis system that accesses indole and pyrrole aldehyde substrates in a desulfonylative Smiles process. The reaction proceeds under mild, transition-metal-free conditions and captures acyl anion reactivity for the synthesis of a diverse array of 2-aroyl indoles and pyrroles from readily available sulfonamide starting materials.

The Smiles–Truce rearrangement is a powerful approach to transition-metal-free arylation, interchanging easily formed carbon–heteroatom bonds for synthetically more challenging carbon–carbon bonds, often under very simple conditions (Scheme 1A).^1^ Advances over the past decade exhibit vast scope in terms of the leaving group, nucleophile, and substrate structures accessible to Smiles reactivity.^2^ A new frontier for Smiles–Truce rearrangement lies in the integration of both the polar and radical manifolds with new catalytic methods. The catalysis approach has been very successful in the radical regime, with metallophotoredox catalysis in particular being instrumental in the development of new single electron Smiles systems.^3^ Catalysis systems in the polar regime, in contrast, are less developed. Smiles–Truce systems that exploit carbanion rearrangements frequently require strongly basic conditions, with benzylic deprotonation with *n*-BuLi being the canonical example.^4^ The introduction of new catalysis approaches to carbanion reactivity could substantially enhance the applications of this arylation technology.

**Scheme 1:**
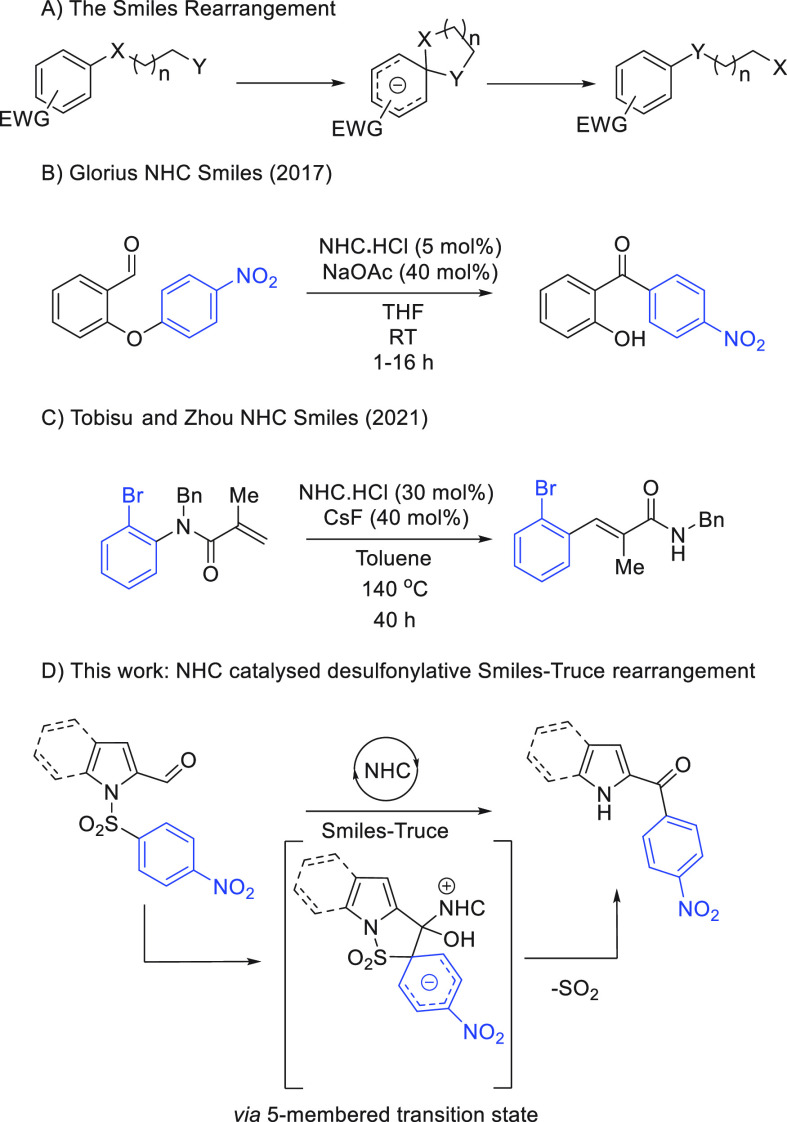
Smiles Rearrangements

We have developed a number of carbanion-based Smiles–Truce systems that harness desulfonylation of sulfonamides.^5^ This powerful class of arylation uses readily accessible sulfonamides and is driven to irreversible completion by SO_2_ extrusion.^6^ We wondered whether N-heterocyclic carbene (NHC) catalysis could be utilized for this substrate class, as it would open up new arylation pathways in the absence of strong bases. NHC chemistry is foundational to organocatalysis, driven by the innate ability of NHCs to enable acyl anion umpolung reactivity with aldehyde substrates.^7^

Notable examples exist for aldehyde arylation using aryl electrophiles such as aryl fluorides,^8^ iodoniums,^9^ and arynes.^10^ Smiles–Truce applications, in contrast, are limited: Glorius established NHC catalysis for the salicylaldehyde-derived phenolic ether system, demonstrating aryl transfer via Stetter-like NHC catalytic cycles to afford aroyl ketones (Scheme 1B).^11^ The group of Ye subsequently described a radical NHC approach for the same class of aldehyde substrates.^12^ Recently, the groups of Tobisu and Zhou described an NHC-catalyzed Smiles system on acrylamides, with the reaction hypothesized to proceed through a deoxy-Breslow intermediate arising from NHC addition to the Michael acceptor (Scheme 1C).^13^ Sulfonamide applications, in contrast, have yet to be developed.

We selected 2-aroyl pyrrole and 2-aroyl indole derivatives as our target structures, which are fundamental building blocks for biologically active heterocycles in the pharmaceutical industry. Our planned Smiles rearrangement would proceed through a favorable 5-membered transition state, delivering aroylated N–H products for further functionalization. The pyrrole products are typically accessed through classical Friedel–Crafts methods with strong Lewis acids as stoichiometric activators.^14^ However, competing functionalization of the N and C-3 positions often leads to reduced regioselectivity, yielding mixtures of mono- and disubstituted side-products. The indole series can be synthesized *de novo*, *e*.*g*. through a Cadogan cyclization of 2-nitrostyrenes,^15^ or via organometallic methods that require a directing group on nitrogen.^16^ The development of a metal-free transformation under mild conditions is thus highly desirable.

We began work with nosylated indole substrate **1a**, prepared in one step by facile sulfonamide formation on commercially available indol-2-carbaldehyde. A screen of various NHC catalysts was immediately successful, with Smiles reactivity observed when using triazolium based NHCs (1–5), with NHC 1 delivering the highest yield of ketone **2a** when used with NaOAc in DMF (75%, Scheme 2). Imidazolium and thioazolium NHCs proved ineffective for our process, failing to induce the desired reactivity (see Supporting Information (SI) for details). Further variation in base, solvent, and temperature did not afford improvements, and control experiments established the requirement for NHC and base for reaction (Table 1, entries 9 and 10).

**Table 1:**
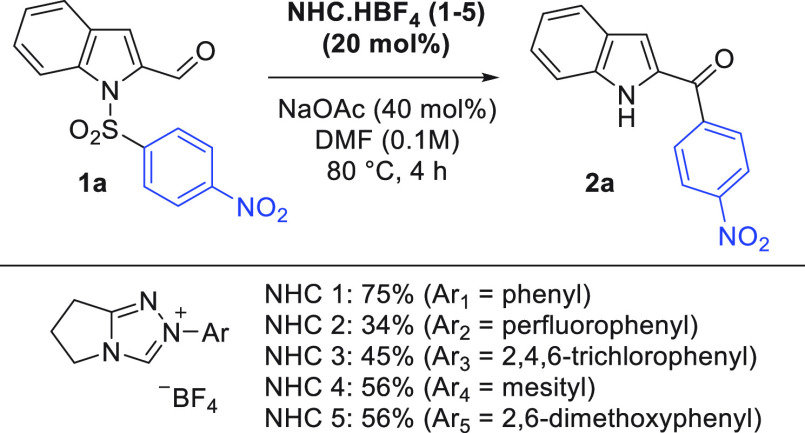
Reaction Optimization^a^

entry	deviation	yield (%)^b^
1	–	75%
2	DMSO	64%
3	toluene	0%
4	K_3_PO_4_	64%
5	DBU	15%
6	30 °C^c^	60%
7	50 °C^c^	69%
8	NHC 1 (10 mol %)	65%
9	No NHC	0%
10	No Base	0%

a 0.1 mmol scale.

b Isolated yield.

c 16 h reaction time.

**Scheme 2:**
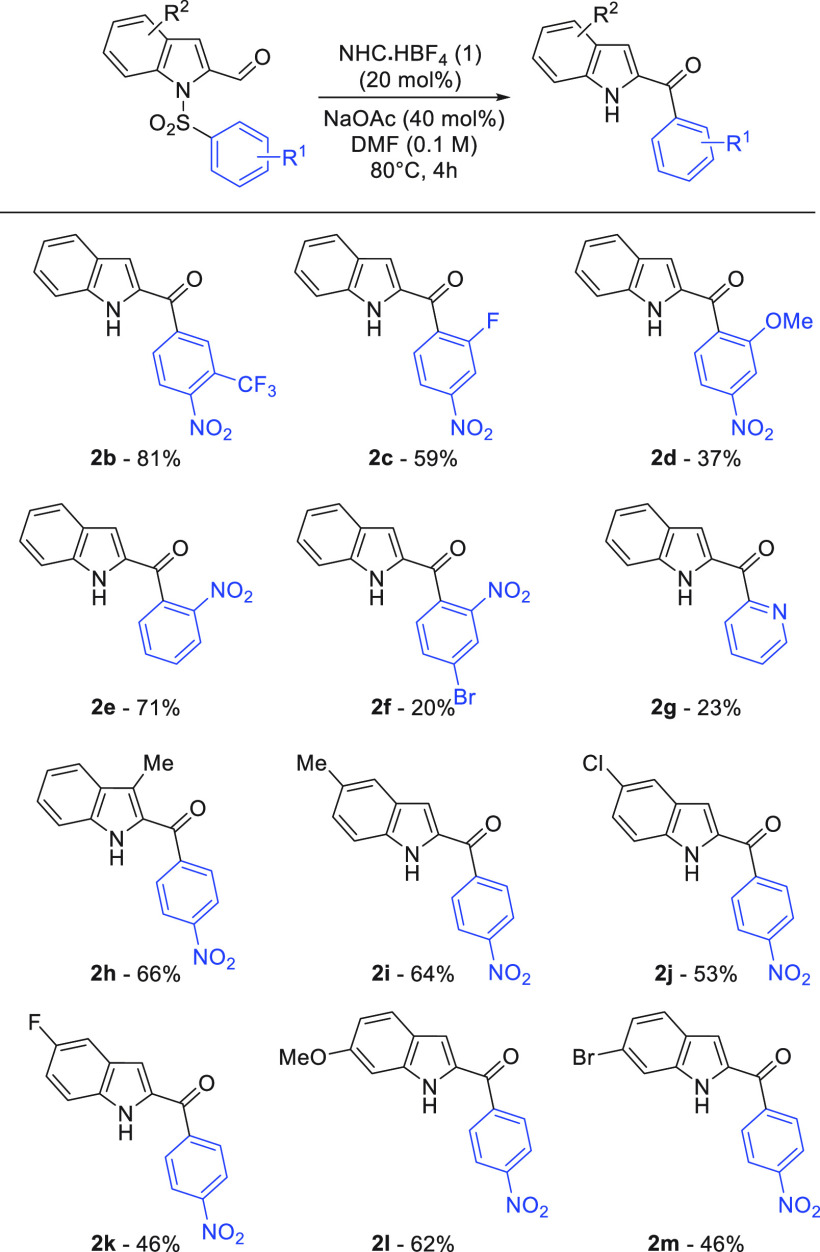
Substrate Scope 0.1 mmol scale.

With optimal conditions in hand, the scope of the reaction was examined, beginning with the migrating ring. The reaction proved successful with both *ortho-* and *para*-nitro groups due to their unique ability to stabilize the anionic Meisenheimer intermediate (Scheme 2, entries **2a**–**2f**). However, weaker electron-withdrawing functionalities such as *p*-CN, *p*-CF_3_, and *p*-CO_2_Me, which have been used successfully in anionic Smiles systems,^17^ were unsuccessful under the NHC catalysis conditions (SI Table 8). The 2-pyridyl substrate did deliver the expected product **2g**, but in a low yield. Instability of the starting sulfonamide **1g** could account for the reduced yields as high levels of degradation were observed in each case. Turning to the indole heteroarene structure, a selection of alkyl, methoxy, and halogen groups were all well-tolerated around the indole arene ring, and we could successfully substitute the 3-position with a Me group without penalty (**2h**–**2m**). The parent example (**2a**) could be scaled up to 1 mmol without penalty (62% yield) and to 1 g with a small reduction in efficiency (48% yield).

Turning to pyrrole sulfonamides, a brief optimization established comparable reactivity, but using NHC 5 as the optimal catalyst under more dilute conditions (Table 2). In comparison to the indole reaction, the aroylated pyrrole products appear to be less stable as there is a significant decrease in product yield when left heating for 16 h (Table 2, entry 3).

**Table 2:**
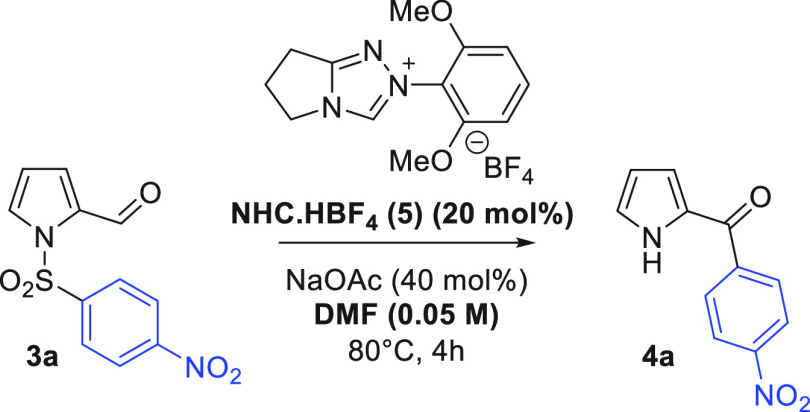
Pyrrole Reaction Optimization^a^

entry	deviation	yield of **2a**^b^
1	none	70%
2	DMF (0.1 M)	55%
3	50 °C^c^	56%
4	NHC 1^c^	24%

a 0.1 mmol scale.

b Isolated yield.

c 16 h reaction time at 50 °C.

The pyrrole series worked well for the cardinal *p*- and *o*-nosyl substrates, giving good yields of ketone **4a**–**4c** (Scheme 3). Production of **4a** was investigated on a 1 mmol scale and gave a very similar yield (65%). Further exemplifications were more challenging, with N–S bond fragmentation observed in a number of cases, eroding yields.^18^ Halogen substitution was tolerated in the migrating ring (**4d** and **4e**), and an iodopyrrole substrate was successfully transformed into **4g**, but in diminished yield.

**Scheme 3:**
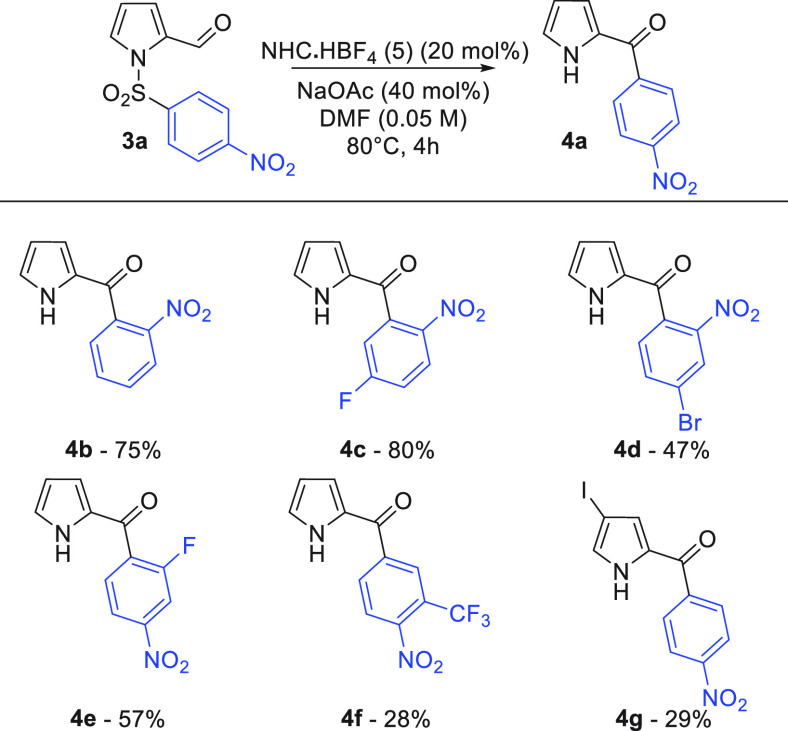
Pyrrole Smiles–Truce Optimization 0.1 mmol scale.

A diverse array of functionalizations were shown to be possible on both the 2-aroyl indole and pyrrole products (Scheme 4). Bromination in the 3-position of the indole proceeded smoothly, yielding bromoindole **5**, a substrate for downstream cross-coupling reactions. The 2-aroyl indole could be reduced using NaBH_4_, revealing secondary alcohol **6**, for further reactions. Functionalization at both indole and pyrrole nitrogen centers was successful through alkylation with iodomethane and K_2_CO_3_ in DMF (**7a** and **7b**). Importantly, conversion of the nitro group to the aniline can be achieved with zinc reduction (**8a** and **8b**). The aniline substrates can subsequently be utilized in a range of pharmaceutically useful chemistries including metal coupling, amide couplings, and S_N_Ar. Subjection of the *ortho*-fluoro product **4e** to S_N_Ar conditions gave the tricyclic 9*H*-pyrrolo[1,2-*a*]indol-9-one product **9**, representing a new entry into this heterocycle.

**Scheme 4:**
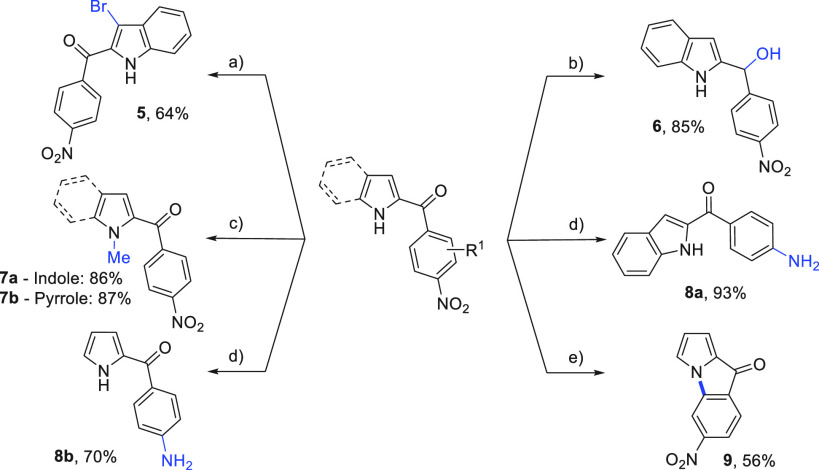
Product Derivatization Reaction conditions: (a) 0.1 mmol scale, NBS (1.2 equiv), DMF (0.1 M), rt 3 h; (b) 0.1 mmol scale, NaBH_4_ (3 equiv), MeOH (0.1 M), 0 °C, 16 h; (c) 0.1 mmol scale, MeI (2 equiv), K_2_CO_3_ (2.5 equiv), DMF (0.1 M), rt, 16 h; (d) 0.1 mmol scale, Zn (4.2 equiv), AcOH, EtOH:H_2_O (3 mL, 1:2 EtOH:H_2_O), 80 °C, 3 h; (e) 0.1 mmol scale, Cs_2_CO_3_ (2 equiv), DMF (0.1 M), substrate **4e**, 70 °C, 16 h.

Turning to the mechanism of the reaction, we carried out a crossover experiment to probe any intermolecularity in the proposed aryl transfer step (Scheme 5). Using substrates **1c** and **1h**, we observed no crossover product formation, supporting the Smiles–Truce reaction pathway. An outline mechanism is set out in Scheme 6: Based on control experiments with no base (Table 1, entry 10) and previous literature, NHC 1 must first be generated via deprotonation. Addition of the carbene to the aldehyde forms the Breslow intermediate, which reacts via the Smiles rearrangement through a Meisenheimer intermediate. Following loss of sulfur dioxide and catalyst regeneration, product 2-aroyl indole or pyrrole is formed.

**Scheme 5:**
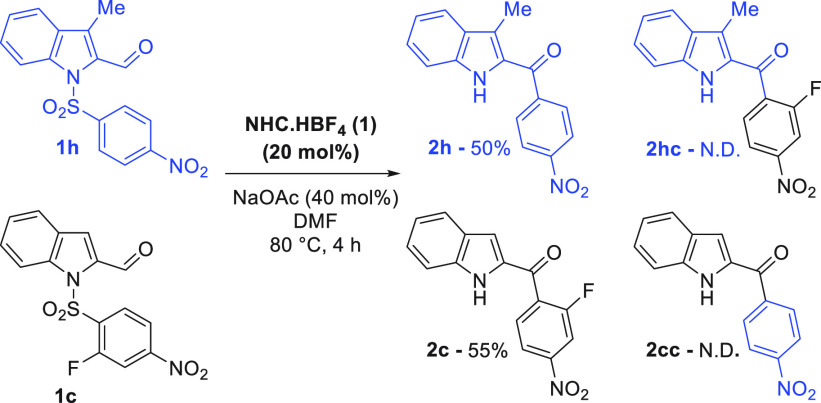
Crossover Experiment 0.1 mmol scale, standard conditions, NMR yields.

**Scheme 6:**
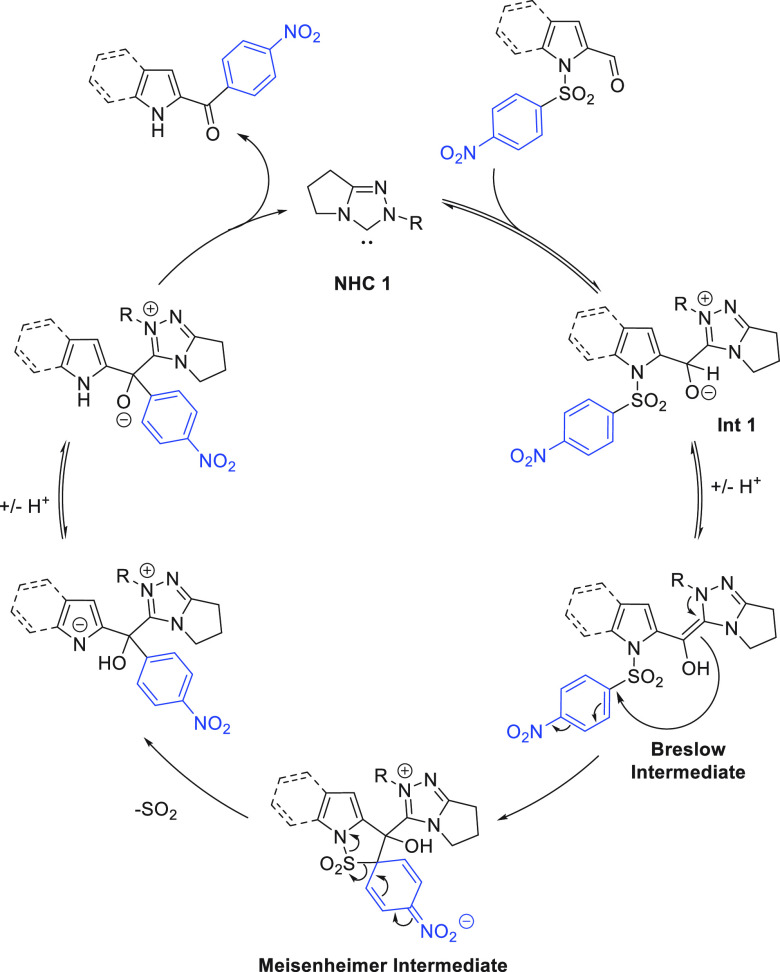
Mechanistic Pathway

The extrusion of SO_2_ could in principle impact the catalytic cycle by sequestering the NHC catalyst – NHC–SO_2_ adducts have been reported in the literature for some classes of NHC.^19^ Recent work from Maulide and co-workers identified this phenomenon for an amine-mediated desulfonylative Smiles reaction, where DABCO was used to set up a Smiles reaction via a Morita–Baylis–Hilman process.^2c^ We varied the NHC stoichiometry (Table 1) to investigate the possibility that the Lewis base catalyst is deactivated in this manner. While the reaction did start to lose efficiency at low loadings, higher loadings (>20 mol %) did not noticeably improve the reaction, suggesting that SO_2_ capture by NHC is not significant for the reaction at hand.

In conclusion, we have demonstrated the use of NHC catalysis for desulfonylative Smiles rearrangements. Extrusion of the Lewis acidic SO_2_ byproduct was found to be feasible under catalysis conditions, establishing a mild and direct route to the aroylated indole and pyrrole heterocycles.

## Data Availability

The data underlying this study are available in the published article and its Supporting Information.

## Supporting Information Available

Preparative procedures and spectroscopic data for all starting materials and Smiles rearrangement products, plus NMR spectra for synthesized compounds. (PDF)
