# Supplementary material for: An NHC-Catalyzed Desulfonylative Smiles Rearrangement of Pyrrole and Indole Carboxaldehydes
Source: J Org Chem. 2023 Aug 17;88(17):12821–5. doi: 10.1021/acs.joc.3c01089 (PMC10476196; doi:10.1021/acs.joc.3c01089)
Supplement: Supplementary file 1 — jo3c01089_si_001.pdf [file jo3c01089_si_001.pdf]

An NHC-Catalyzed Desulfonylative Smiles Rearrangement of Pyrrole and Indole Carboxaldehydes

Caitlin Swaby,[†] Alfie Taylor,[†] Michael F. Greaney*

Dept. of Chemistry, University of Manchester, Oxford Rd, Manchester, M13 9PL, UK

michael.greaney@manchester.ac.uk

[†]Both authors contributed equally to this work

Supporting information

Contents

1. General Remarks 2

2. Preparation of pre-catalysts 3

2.1. Synthesis of NHC 5 3

3. Indole Optimisation 4

4. General Procedures 9

5. Synthesis and characterisation of compounds 12

5.1. Preparations of (1H-indol-2-yl)methanols 12

5.2. Preparations of indole-2-carbaldehydes 14

5.3. Preparations of arylsulfonyl-indole-2-carbaldehydes 17

5.4. Preparations of 2-aroylindoles 24

5.5. Preparations of arylsulfonyl-pyrrole-2-carbaldehydes 31

5.6. Preparations of 2-aroylpyrroles 35

5.7. Derivatisation reactions 39

6. Crossover Experiment **43**

7. NMR Spectra for Synthesised Compounds **47**

8. References **124**

1. General Remarks

^{1}H NMR spectra were recorded on a Bruker Advance 400 (400 MHz) or 500 (500 MHz) spectrometers. Chemical shifts (δ) are reported in parts per million (ppm) to the closest 0.01 ppm and coupling constants (J) are recorded in Hertz (Hz) using $CDCl_3$ as reference. ^{13}C NMR spectra were recorded on Bruker Advance 500 (125 MHz) or 400 (100 MHz) spectrometers. Multiplicity recorded with the following abbreviations: singlet (s), broad singlet (brs), doublet (d), triplet (t), quartet (q), doublet of doublets (dd), doublet of doublet of doublets (ddd), quartet of doublets (qd), multiplet (m). High resolution mass spectrometry was recorded on a Waters QTOF with ESI/ESI ionization and a Thermo Finnigan MAT95XP. Thin layer chromatography (TLC) was performed using pre-coated Merck aluminium backed TLC Silica gel plates (60 F254) and spots were visualised by applying ultraviolet light (254 nm) or by coating with Ninhydride stain. Preparative TLC was carried out using Uniplate Silica Gel GF glass plates (20 x 20 cm, 2000 µM). Flash chromatography was performed using a Biotage Isolera Four purification system using Biotage SNAP KP-Sil 10, 25 or 50 g or Sfar Silica D 50, 100 g cartridges. All solvents and reagents were used as obtained from commercial sources without further purification, unless stated otherwise. NMR yields were calculated using nitromethane as internal standard. IR spectra were obtained using a Bruker Alpha infrared spectrometer, using the following abbreviations for peaks: broad (br), strong (s). Melting points were obtained using a Griffin melting point apparatus.

2. Preparation of pre-catalysts

NHC pre-catalyst 1[1], 3[1], 4[1, 2], 5[(see below)], 6[3], 7,[2, 3] 8[2, 4] and 9[4] were prepared according to literature procedures whilst NHC 2 was commercially available.

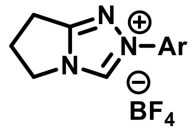

NHC 1: Ar_1 = Phenyl
NHC 2: Ar_2 = perfluorophenyl
NHC 3: Ar_3 = 2,4,6-trichlorophenyl
NHC 4: Ar_4 = mesityl
NHC 5: Ar_5 = 2,6-dimethoxyphenyl

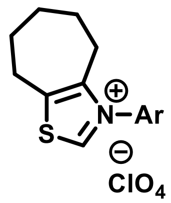

NHC 6: Ar_4 = mesityl
NHC 7: Ar_6 = 2,6-diisopropylphenyl

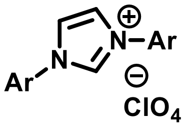

NHC 8: Ar_4 = mesityl
NHC 9: Ar_6 = 2,6-diisopropylphenyl

2.1. Synthesis of NHC 5

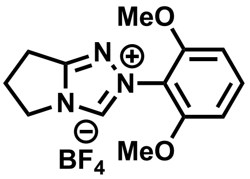

85 - NHC 5. HBF_4

An oven dried RBF (250 mL) was charged with 2-pyrrolidinone (2.02 g, 18.1 mmol) and DCM (120 mL). Trimethyloxonium tetrafluoroborate (2.96 g, 20 mmol) was added and the reaction mixture stirred for 16 hours at room temperature. (2,6-dimethoxyphenyl)hydrazine (3.36 g, 20 mmol) was added dropwise to the solution and stirred at room temperature for a further 16 hours. Solvent was removed *in vacuo* and crude used without further purification. Crude was dissolved in methanol: trimethyl orthoformate (1:4, 50 mL), the reaction mixture heated to 80 °C in an oil bath and stirred at this temperature for 6 hours. Solvent was removed in vacuo, recrystallised from hot MeOH to give NHC 5 as an off-white solid (1.41 g, 21%).
^{1}H NMR (400 MHz, CD_3OD) δ 7.62 (t, *J* = 8.6 Hz, 1H), 6.92 (d, *J* = 8.6 Hz, 2H), 4.57 – 4.49 (m, 2H, N-C*H₂*), 3.87 (s, 6H), 3.27 (dd, *J* = 8.3, 7.2 Hz, 2H), 2.95 – 2.83 (m, 2H).
^{13}C{^{1}H} NMR (101 MHz, CD_3OD) δ 162.9, 155.7, 133.4, 104.2, 55.6, 47.9, 26.4, 21.1.
^{19}F NMR (376 MHz, CD_3OD) δ -154.84 (dt, *J* = 2.6, 1.2 Hz, B*F_4*).
^{11}B NMR (128 MHz, CD_3OD) δ = -1.2 (*B*F_4).
Data consistent with literature.[5]

***Synthesis of** 1,2-Bis(1,1-dimethylethyl) 1-(2,6-dimethoxyphenyl)-1,2-hydrazinedicarboxylate*

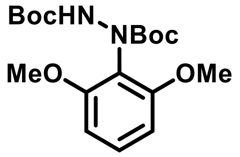

n-BuLi (1.6 M solution in hexanes, 13.8 mL, 22.0 mmol, 1.1 equiv) was added dropwise to a solution of 1,3-dimethoxybenzene (2.76 g, 20.0 mmol, 1.0 equiv) and tetramethylethylenediamine (3.28 mL, 22.0 mmol, 1.1 equiv) in THF (160 mL, 0.125 M) at 0 °C. After 1 h of stirring at this temperature the reaction mixture was cooled to –78 °C and a solution of di-tert-butyl diazene-1,2-dicarboxylate (4.60 g, 20.0 mmol, 1.0 equiv) in THF (40 mL, 0.5 M) added dropwise via cannula. After 20 minutes, the reaction mixture was quenched with addition of AcOH (1.15 mL, 20.0 mmol, 1.0 equiv) at –78 °C. The resulting solution was allowed to warm to room temperature, diluted with EtOAc (300 mL) and poured into distilled water (400 mL). Organics collected and the aqueous layer was extracted with EtOAc (3 x 300 mL) and combined organics washed with brine before drying over Na_2SO_4, filtering and dry loading onto silica. From which a 100 g Biotage SNAP KP-Sil column was run on a Biotage Isolera Four purification system (0 – 50% EtOAc in hexanes) to afford di-tert-butyl 1-(2,6-dimethoxyphenyl)hydrazine-1,2-dicarboxylate as pale yellow foam (1.6 g, 22%).

^{1}H NMR (400 MHz,$CDCl_3$) δ 7.21 (td, *J* = 8.5, 2.9 Hz, 1H, Ar), 7.05 – 6.68 (m, 1H), 6.57 (t, *J* = 8.2 Hz, 2H, Ar), 3.87 (s, 6H, OMe), 1.59 – 1.36 (m, 18H, BOC).
HRMS (ESI) Calculated for: $[C_{13}H_{16}O_2N_3]^+$ 246.1237, found 246.1229.
Data consistent with literature.[5]

3. Indole Optimisation

General Procedure for optimisation

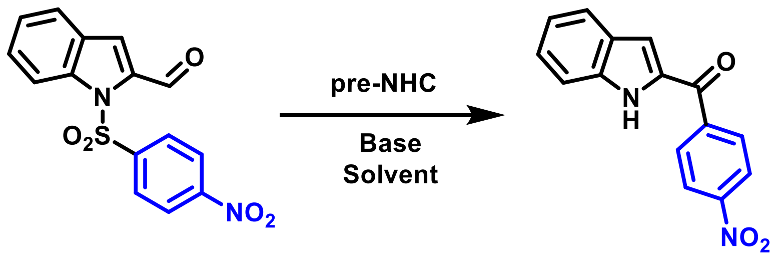

An oven-dried microwave vial (2-5 mL) containing 1-((4-nitrophenyl)sulfonyl)-1H-indole-2-carbaldehyde (33 mg, 0.1 mmol, 1 Eq.), pre-NHC (0.02 mmol, 20 mol%) and base (0.04 mmol, 40 mol%) was evacuated and backfilled 3 times with nitrogen. Solvent (0.2 - 2 mL) added* and the sealed vessel was heated at the appropriate temperature (oil bath) for set time. Reaction allowed to cool to room temperature, brine (2 mL) added and aqueous extracted with EtOAc (3 mL x3). Combined organics were dry loaded onto silica before purifying via a biotage isolera system (0 – 25% EtOAc in hexanes) to give isolated yields.

*From Table 4 (optimisation of time and temperature) and all optimisation and reactions following include 15 minutes of bubbling N_2 through reaction mixture post addition of solvent.

Optimisation of NHC

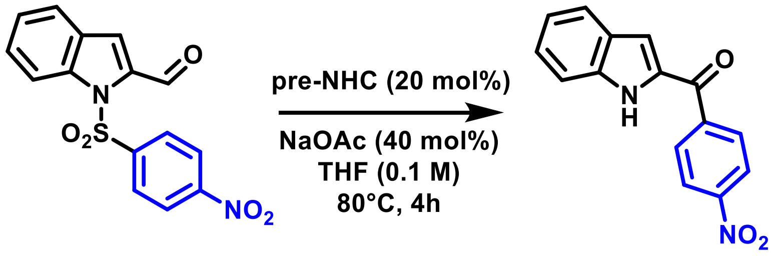

Entry	Pre-NHC	Yield
**1**	NHC 1	15%
**2**	NHC 2	<5%
**3**	NHC 3	<5%
**4**	NHC 4	11%
**5**	NHC 5	49%*
**6**	NHC 6	0%
**7**	NHC 7	0%
**8**	NHC 8	0%
**9**	NHC 9	0%

*Table 1: Reactions were carried out at 80 °C for 4 hours with **1a** (33 mg, 0.1 mmol, 1 Eq.) NHC 1-9 (0.02 mmol, 20 mol%), NaOAc (3.3 mg, 0.04 mmol, 40 mol%) and THF (1 mL). *Catalyst had not been synthesised at time of optimisation.*

Optimisation of Solvent

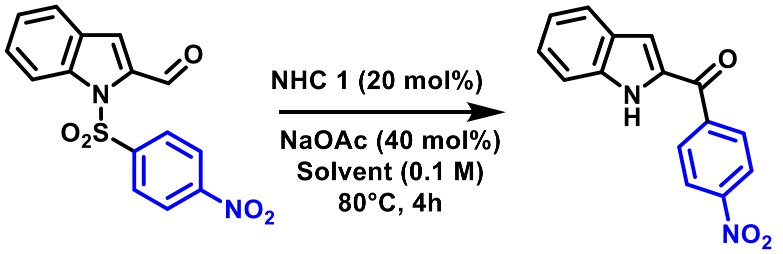

Entry	Solvent	Yield
**1**	Dioxane	<5%
**2**	DCE	5%
**3**	Toluene	0%
**4**	EtOAc	5%
**5**	DMF	56%
**6**	DMSO	49%
**7**	IPA	10%
**8**	THF	15%

*Table 2: Reactions were carried out at 80 °C for 4 hours with **1a** (33 mg, 0.1 mmol, 1 Eq.) NHC 1 (5.5 mg, 0.02 mmol, 20 mol%), NaOAc (3.3 mg, 0.04 mmol, 40 mol%) and solvent (1 mL)*

Screening of pre-NHC under optimised conditions

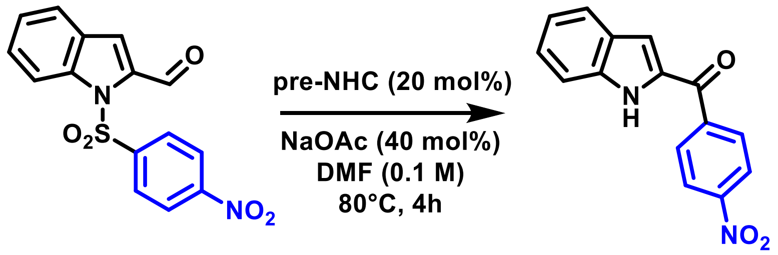

Entry	Pre-NHC	Yield
**1**	NHC 1	75%
**2**	NHC 2	34%
**3**	NHC 3	45%
**4**	NHC 4	56%
**5**	NHC 5	56%

*Table 3: Reactions were nitrogen sparged for 15 minutes then carried out at 80 °C for 4 hours with **1a** (33 mg, 0.1 mmol, 1 Eq.) NHC 1-5 (0.02 mmol, 20 mol%), NaOAc (3.3 mg, 0.04 mmol, 40 mol%) and DMF (1 mL)*

Optimisation of Base

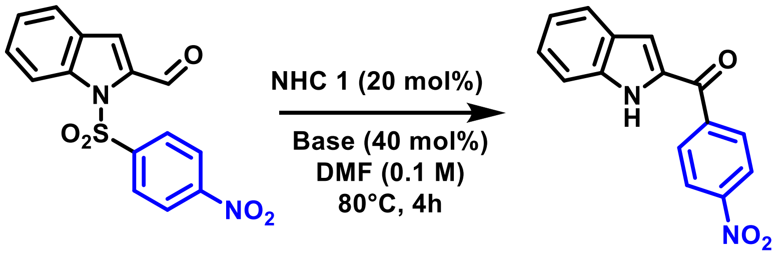

Entry	Base	Yield
**1**	NaOAc	75%
**2**	DBU	15%
**3**	K_2CO_3	60%
**4**	Cs_2CO_3	56%
**5**	^tBuOK	53%
**6**	K_3PO_4	64%
**7**	NEt_3	53%
**8**	DIPEA	41%

*Table 4: Reactions were nitrogen sparged for 15 minutes then carried out at 80 °C for 4 hours with **1a** (33 mg, 0.1 mmol, 1 Eq.) NHC 1 (5.5 mg, 0.02 mmol, 20 mol%), base (0.04 mmol, 40 mol%) and DMF (1 mL).*

Optimisation of time and temperature

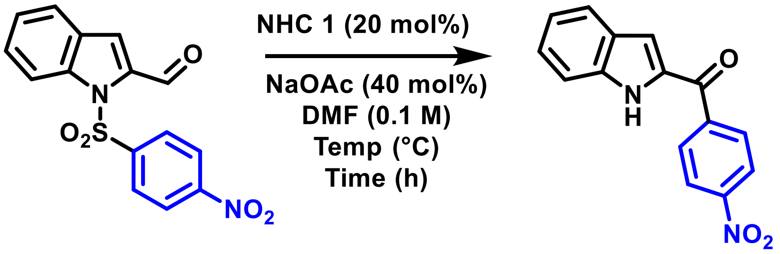

Entry	Temp (°C)	Time (h)	Yield
**1**	15	16	48%
**2**	30	16	60%
**3**	50	16	70%
**4**	50	4	61%
**5**	80	4	75%

Table 5: Reactions were nitrogen sparged for 15 minutes then carried out at stated times and temperatures with ***1a*** *(33 mg, 0.1 mmol, 1 Eq.) NHC 1 (5.5 mg, 0.02 mmol, 20 mol%), base (0.04 mmol, 40 mol%) and DMF (1 mL)*

Optimisation of Catalyst Loading

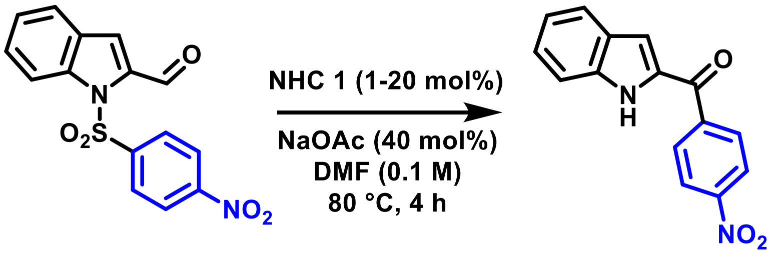

Entry	Catalyst loading (mol%)	Yield
**1**	1	11%
**2**	2	31%
**3**	5	46%
**4**	10	70%
**5**	20	75%

Table 6: Reactions were nitrogen sparged for 15 minutes then carried out at 80 °C for 4 hours with ***1a*** *(33 mg, 0.1 mmol, 1 Eq.) NHC 1 (1-20 mol%), NaOAc (3.3 mg, 0.04 mmol, 40 mol%) and DMF (1 mL)*

Optimisation of reaction concentration

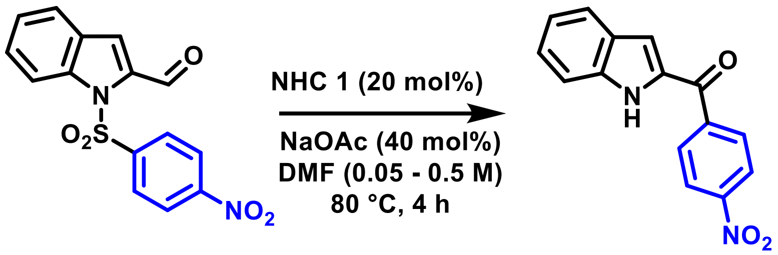

Entry	Concentration (M)	Yield
**1**	0.5	39%
**2**	0.2	47%
**3**	0.1	75%
**4**	0.05	73%

*Table 7: Reactions were nitrogen sparged for 15 minutes then carried out at 80 °C for 4 hours with **1a** (33 mg, 0.1 mmol, 1 Eq.) NHC 1 (1-20 mol%), NaOAc (3.3 mg, 0.04 mmol, 40 mol%) and DMF (0.2 - 2 mL)*

Failed Migrating Ring Scope

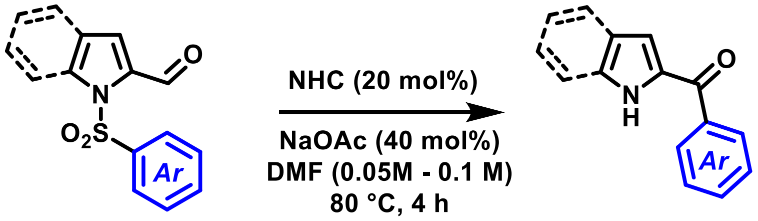

Entry	Substrate	R	Residual
**1**	Indole	4-F	SM + Minor Degradation
**2**	Indole	4-CF_3	SM only
**3**	Indole	4-CN	SM only
**4**	Pyrrole	4-C(O)Me	Degradation
**5**	Pyrrole	4-CN	SM only
**6**	Pyrrole	4-CO_2Me	SM + Minor Degradation
**7**	Pyrrole	2- CO_2Me	SM + Minor Degradation
**8**	Pyrrole	2,4-NO_2	Degradation
**9**	Pyrrole	4-CF_3	SM only

Table 8: Reactions were nitrogen sparged for 15 minutes then carried out at 80 °C for 4 hours with corresponding sulfonamide (0.1 mmol, 1 Eq.) NHC (0.02 mmol, 20 mol%), NaOAc (3.3 mg, 0.04 mmol, 40 mol%) and DMF (1-2 mL)

4. General Procedures

General Procedure A *– For the reduction of alkyl-indole-2-carboxylate.* [6]

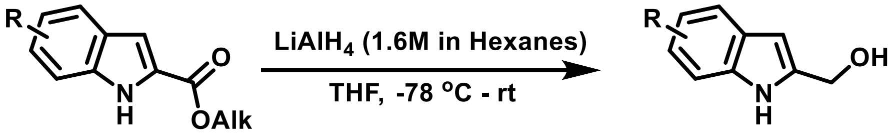

Indole ester (1 Eq.) was dissolved in dry THF (0.1 M) and cooled to -78 °C under a nitrogen atmosphere. A solution of $LiAlH_4$ (2 M in THF, 3 Eq.) was added dropwise, and the reaction mixture stirred whilst warming to room temperature for 16 hours. The reaction was cooled to 0 °C, water (0.12 Eq.) added dropwise, followed by NaOH (2M Aq. Sol., 0.12 Eq.) and water (0.3 Eq.). The reaction stirred at room temperature for 15 minutes and the resultant precipitate removed by filtration. The filtrate was dry loaded onto silica before purifying via a biotage isolera system (0 – 50% EtOAc in hexanes).

General Procedure B *– For preparation of indole-2-carbaldehydes.* [7]

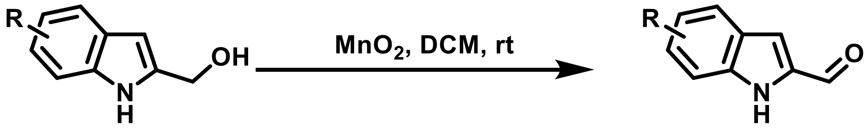

MnO_2 (5 Eq.) was added to indole alcohol (1 Eq.) in DCM (0.1 M) and the reaction mixture stirred at room temperature for 16 hours. Reaction was filtered through celite and the filtrate dry loaded onto silica before purifying via a biotage isolera system (0 – 20% EtOAc in hexanes).

General Procedure C *– For the synthesis arylsulfonyl-indole-2-carbaldehyde.*

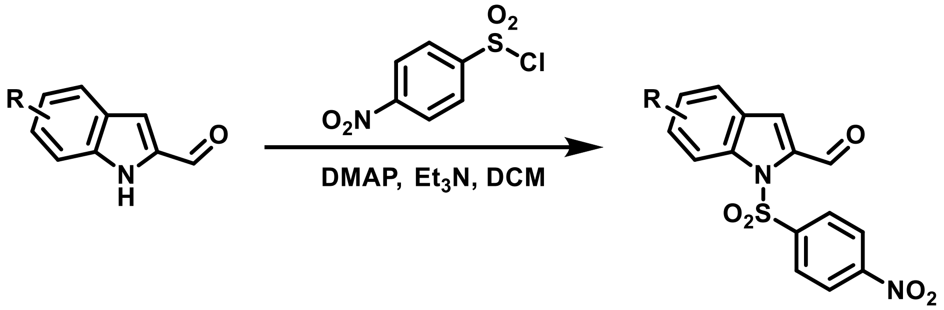

Triethylamine (1.25 Eq.) was added to indole aldehyde (1 Eq.) and DMAP (0.125 Eq.) in DCM (0.1M) followed by the appropriate sulfonyl chloride (1.2 Eq.) and the reaction mixture stirred at room temperature for 16 hours. NH_4Cl (Sat. Sol. Aq.) was added, organics collected and the aqueous extracted with DCM (x 3). Combined organics were washed with brine, dried over $MgSO_4$ and the filtrate dry loaded onto silica before purifying via a biotage isolera system (0 – 20% EtOAc in hexanes).

General Procedure D *– For NHC catalysed desulfonylative Smiles-Truce rearrangement of indoles.*

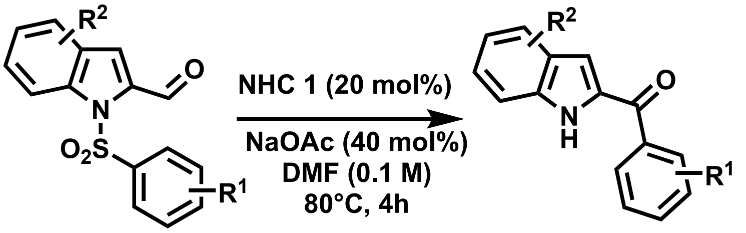

An oven-dried microwave vial (2 – 5 mL) containing indole sulfonamide (0.1 mmol, 1 Eq.)C 1 (5.5 mg, 0.02 mmol, 20 mol%) and NaOAc (3.3 mg, 0.04 mmol, 40 mol%) was evacuated and backfilled 3 times with nitrogen. DMF (1 mL) added and the reaction mixture degassed by sparging N_2 for 15 minutes at room temperature. The sealed vessel was heated in an oil bath for 4 hours at 80 °C, allowed to cool to room temperature, brine (2 mL) added and aqueous extracted with EtOAc (3 mL x3). Combined organics were dry loaded onto silica before purifying via a biotage isolera system (0 – 25% EtOAc in hexanes).

General Procedure E *- For the synthesis of pyrrole sulfonamides.*

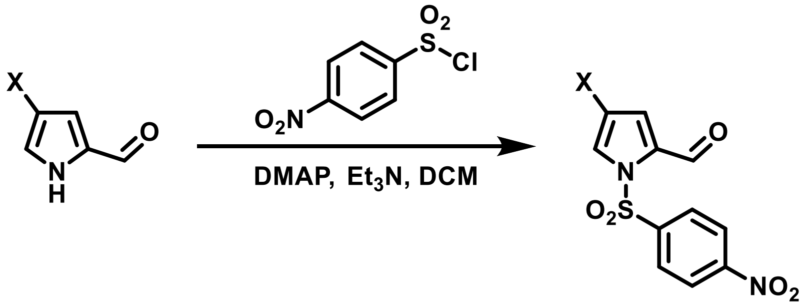

Triethylamine (1.25 Eq.) was added to pyrrole aldehyde (1 Eq.) and DMAP (0.125 Eq.) in DCM (0.1M) followed by the appropriate sulfonyl chloride (1.2 Eq.) and the reaction mixture stirred at room temperature for 16 hours. NH_4Cl (Sat. Sol. Aq.) was added, organics collected and the aqueous extracted with DCM (x 3). Combined organics were washed with brine, dried over $MgSO_4$ and the filtrate dry loaded onto silica before purifying via a biotage isolera system (0 – 20% EtOAc in hexanes).

General Procedure F *for NHC catalysed desulfonylative Truce-Smiles rearrangement of pyrrole.*

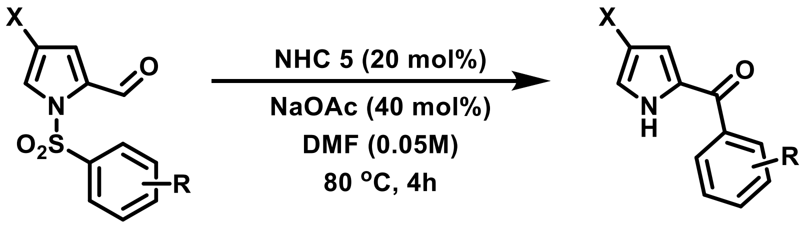

An oven-dried microwave vial (2 – 5 mL) containing pyrrole sulfonamide (0.1 mmol, 1 Eq.)C 5 (6.7 mg, 0.02 mmol, 20 mol%) and NaOAc (3.3 mg, 0.04 mmol, 40 mol%) was evacuated and

backfilled 3 times with nitrogen. DMF (2 mL) added and the reaction mixture degassed with N_2 bubbling through the solvent for 15 minutes at room temperature. The sealed vessel was heated in an oil bath for 4 hours at 80 °C, allowed to cool to room temperature, brine (2 mL) added and aqueous extracted with EtOAc (3 mL x3). Combined organics were dry loaded onto silica before purifying via a biotage isolera system (0 – 5% EtOAc in Toluene or 0-25% EtOAc in hexane).

***General Procedure G** - For the scaled up desulfonylative Truce-Smiles rearrangement of indole **1a**.*

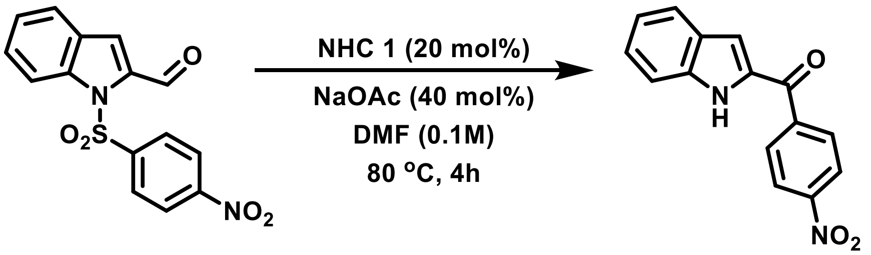

An oven-dried RBF (50 mL) containing 1-((4-nitrophenyl)sulfonyl)-1H-indole-2-carbaldehyde (330 mg, 1 mmol, 1 Eq.)C 1 (55 mg, 0.2 mmol, 20 mol%) and NaOAc (3.3 mg, 0.04 mmol, 40 mol%) was evacuated and backfilled 3 times with nitrogen. DMF (10 mL) added and the reaction stirred at room temperature under N_2 for 15 minutes. The reaction mixture was heated in an oil bath for 4 hours at 80 °C, allowed to cool to room temperature, brine (20 mL) added and aqueous extracted with EtOAc (20 mL x3). Combined organics were washed with LiCl (Sat. Sol. Aq.) before drying with $MgSO_4$ and dry loading onto silica. From which a 50 g Biotage SNAP KP-Sil column was run on a Biotage Isolera Four purification system (0 – 25% EtOAc in hexanes) to give (1H-indol-2-yl)(4-nitrophenyl)methanone as a yellow solid (166 mg, 62 %).

5. Synthesis and characterisation of compounds

5.1. Preparations of (1H-indol-2-yl)methanols

(1*H*-indol-2-yl)methanol (1aa) – Alternative starting material preparation of 1a

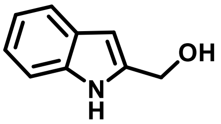

Prepared according to general procedure **A** using 1H-indole-2-carboxylate (7.00 g, 40 mmol). From which a Sfar Silica D 100 g column was run on a Biotage Isolera Four purification system (0 – 40% EtOAc in hexanes) to yield titled compound as a white solid (3.50 g, 60%).

^{1}H NMR (400 MHz, $CDCl_3$) δ 8.36 (s, 1H), 7.59 (d, *J* = 7.8 Hz, 1H), 7.36 – 7.30 (m, 1H), 7.22 – 7.16 (m, 1H), 7.14 – 7.09 (m, 1H), 6.45 – 6.36 (m, 1H), 4.79 (s, 2H), 2.05 (s, 1H).

^{13}C{^{1}H} NMR (101 MHz, $CDCl_3$) δ 137.6, 136.4, 128.1, 122.2, 120.6, 110.0, 100.6, 58.7.

HRMS (ESI) Calculated for: $[C_9H_8NO]^-$ 146.0611, found 146.0598.

Data consistent with literature.[8]

(3-methyl-1H-indol-2-yl)methanol (1ha)

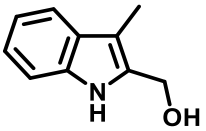

Prepared according to general procedure **A** using ethyl 3-methyl-1H-indole-2-carboxylate (1.02 g, 5 mmol). From which a 50 g Biotage SNAP KP-Sil column was run on a Biotage Isolera Four purification system (0 – 50% EtOAc in hexanes) to yield titled compound as an off white solid (682 mg, 77%).

^{1}H NMR (400 MHz, $CDCl_3$) δ 8.13 (s, 1H), 7.54 (d, *J* = 7.8 Hz, 1H), 7.32 (d, *J* = 8.1 Hz, 1H), 7.19 (ddd, *J* = 8.2, 7.1, 1.2 Hz, 1H), 7.11 (ddd, *J* = 8.0, 7.0, 1.1 Hz, 1H), 4.86 – 4.81 (m, 2H), 2.29 (s, 3H), 1.62 (s, 1H).

^{13}C{^{1}H} NMR (101 MHz, $CDCl_3$) δ 135.8, 133.0, 129.0, 122.4, 119.4, 119.0, 110.9, 108.6, 56.8, 8.5.

HRMS (ESI) Calculated for: $[C_{10}H_{11}NNaO]^+$ 184.0733, found 184.0727.

Data consistent with literature.[9]

(5-methyl-1H-indol-2-yl)methanol (1ia)

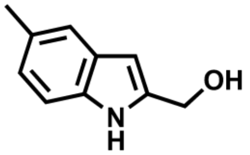

Prepared according to general procedure **A** using ethyl 5-methyl-1H-indole-2-carboxylate (1.01 g, 5 mmol). From which a 50 g Biotage SNAP KP-Sil column was run on a Biotage Isolera

Four purification system (0 – 50% EtOAc in hexanes) to yield titled compound as an off white solid (361 mg, 44%).
^{1}H NMR (400 MHz, $CDCl_3$) δ 8.24 (s, 1H), 7.37 (dd, *J* = 1.8, 0.9 Hz, 1H), 7.21 (d, *J* = 8.2 Hz, 1H), 7.02 (dd, *J* = 8.3, 1.7 Hz, 1H), 6.32 (dd, *J* = 2.1, 1.0 Hz, 1H), 4.77 (s, 2H), 2.44 (s, 3H), 1.98 (s, 1H).
^{13}C{^{1}H} NMR (101 MHz, $CDCl_3$) δ 137.8, 134.8, 129.3, 128.5, 123.9, 120.4, 110.7, 100.3, 58.9, 21.6.
HRMS (ESI) Calculated for $[C_{10}H_{10}NO]^-$ 160.0768, found 160.0764.
Data consistent with literature.[9]

(5-chloro-1H-indol-2-yl)methanol (1ja)

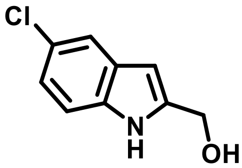

Prepared according to general procedure **A** using ethyl 5-chloro-1H-indole-2-carboxylate (1.12 g, 5 mmol). From which a 50 g Biotage SNAP KP-Sil column was run on a Biotage Isolera Four purification system (0 – 50% EtOAc in hexanes) to yield titled compound as an off white solid (204 mg, 23%).
^{1}H NMR (400 MHz, CDCl3) δ = 8.38 (s, 1H), 7.56 – 7.52 (m, 1H), 7.27 (m), 7.13 (dd, *J* = 8.6, 2.0, 1H), 6.35 (dq, *J* = 1.7, 0.8, 1H), 4.89 – 4.80 (m, 2H), 1.78 (s, 1H).
^{13}C{^{1}H} NMR (101 MHz, $CDCl_3$) δ 139.1. 134.7, 129.4, 125.7, 122.6, 120.1, 112.0, 100.1, 58.8.
HRMS (ESI) Calculated for: $[C9H_7(35)ClNO]^-$ 180.0213, found 180.0222.
Data consistent with literature.[9]

(5-fluoro-1H-indol-2-yl)methanol (1ka)

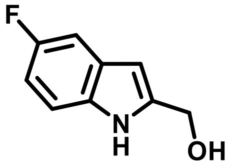

Prepared according to general procedure **A** using ethyl 5-fluoro-1H-indole-2-carboxylate (1.03 g, 5 mmol). From which a 50 g Biotage SNAP KP-Sil column was run on a Biotage Isolera Four purification system (0 – 50% EtOAc in hexanes) to yield titled compound as an off white solid (493 mg, 60%).
^{1}H NMR (500 MHz, $CDCl_3$) δ 8.40 (s, 1H), 7.26 (ddd, *J* = 18.1, 9.2, 3.7 Hz, 2H), 6.95 (td, *J* = 9.1, 2.5 Hz, 1H), 6.39 (d, *J* = 2.1 Hz, 1H), 4.85 (d, *J* = 4.3 Hz, 2H), 1.89 (s, 1H).
^{13}C{^{1}H} NMR (126 MHz, $CDCl_3$) δ 158.1 (d, *J* = 234.3 Hz), 139.5, 132.9, 128.6 (d, *J* = 10.1 Hz), 111.6 (d, *J* = 9.7 Hz), 110.6 (d, *J* = 26.2 Hz), 105.5 (d, *J* = 23.5 Hz), 100.6 (d, *J* = 4.8 Hz), 58.8.
^{19}F NMR (471 MHz, $CDCl_3$) δ -124.56 – -124.64 (m).
HRMS (ESI) Calculated for $[C_9H_7FNO]^-$ 164.0517, found 164.0523.
Data consistent with literature.[9]

(6-methoxy-1H-indol-2-yl)methanol (1la)

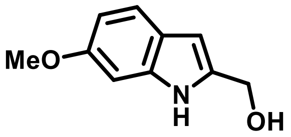

Prepared according to general procedure **A** using ethyl 6-methoxy-1H-indole-2-carboxylate (1.02 g, 5 mmol). From which a 50 g Biotage SNAP KP-Sil column was run on a Biotage Isolera Four purification system (0 – 50% EtOAc in hexanes) to yield titled compound as an off white solid (682 mg, 77%).

^{1}H NMR (500 MHz, $CDCl_3$) δ 8.22 (s, 1H), 7.24 (d, *J* = 9.0 Hz, 1H), 7.04 (d, *J* = 2.4 Hz, 1H), 6.85 (dd, *J* = 8.7, 2.4 Hz, 1H), 6.34 (dd, *J* = 2.1, 1.0 Hz, 1H), 4.81 (d, *J* = 5.3 Hz, 2H), 3.84 (s, 3H), 1.79 (t, *J* = 5.5, 4.2 Hz, 1H).

$^{13}C\{^1H\}$ NMR (126 MHz, $CDCl_3$) δ 154.3, 138.3, 131.5, 128.6, 112.4, 111.6, 102.4, 100.4, 58.8, 55.9.

HRMS (ESI) Calculated for $[C_{10}H_{10}NO_2]^-$ 176.0717, found 176.0717.

Data consistent with literature.[10]

5.2. *Preparations of indole-2-carbaldehydes*

1*H*-indole-2-carbaldehyde (1ab)

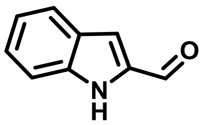

Prepared according to general procedure **B** using 1H-indole-2-carbaldehyde (812 mg, 5.5 mmol) and MnO_2 (2.4 g, 27.6 mmol). From which a 25 g Biotage SNAP KP-Sil column was run on a Biotage Isolera Four purification system (0 – 15% EtOAc in hexanes) to yield titled compound as a white solid (410 mg, 54%).

^{1}H NMR (400 MHz, $CDCl_3$) δ 9.87 (s, 1H), 9.45 (s, 1H), 7.76 (dd, *J* = 8.3, 1.2 Hz, 1H), 7.49 (dt, *J* = 8.4, 1.0 Hz, 1H), 7.40 (ddd, *J* = 8.4, 6.9, 1.2 Hz, 1H), 7.29 (dd, *J* = 2.1, 1.0 Hz, 1H), 7.19 (ddd, *J* = 8.0, 7.0, 1.1 Hz, 1H).

$^{13}C\{^1H\}$ NMR (101 MHz, $CDCl_3$) δ 182.3, 138.3, 136.1, 127.5, 127.5, 123.4, 121.4, 115.1, 112.7.

HRMS (ESI) Calculated for: $[C_9H_6NO]^-$ 144.0455, Found 144.0442.

Data consistent with literature.[11]

3-methyl-1H-indole-2-carbaldehyde (1hb)

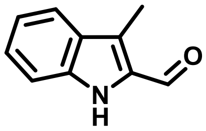

Prepared according to general procedure **B** using (3-methyl-1H-indol-2-yl)methanol (520 mg, 3.23 mmol) and MnO_2 (1.40 g, 16.13 mmol). From which a 25 g Biotage SNAP KP-Sil column

was run on a Biotage Isolera Four purification system (0 – 20% EtOAc in hexanes) to yield titled compound as an off white solid (270 mg, 53%).

^{1}H NMR (400 MHz, CDCl$_3$) δ 10.05 (s, 1H), 8.98 (s, 1H), 7.71 (dq, *J* = 8.2, 1.0 Hz, 1H), 7.44 – 7.34 (m, 2H), 7.16 (ddd, *J* = 8.0, 4.6, 3.3 Hz, 1H), 2.65 (s, 3H).

^{13}C{^{1}H} NMR (101 MHz, CDCl$_3$) δ 180.6, 137.6, 132.3, 128.3, 127.8, 125.1, 121.5, 120.6, 112.4, 8.5.

HRMS (ESI) Calculated for: $[C_{10}H_9NNaO]^+$ 182.0576, Found 182.05730.

Data consistent with literature.[12]

5-methyl-1H-indole-2-carbaldehyde (1ib)

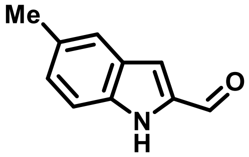

Prepared according to general procedure **B** using (5-methyl-1H-indol-2-yl)methanol (361 mg, 2.24 mmol) and MnO_2 (975 mg, 11.2 mmol). From which a 25 g Biotage SNAP KP-Sil column was run on a Biotage Isolera Four purification system (0 – 20% EtOAc in hexanes) to yield titled compound as an off white solid (242 mg, 68%).

^{1}H NMR (400 MHz, CDCl$_3$) δ 9.82 (s, 1H), 9.00 (s, 1H), 7.52 (d, *J* = 0.9 Hz, 1H), 7.35 (d, *J* = 8.5 Hz, 1H), 7.23 (dd, *J* = 8.4, 1.7 Hz, 1H), 7.19 (dd, *J* = 2.1, 1.0 Hz, 1H), 2.45 (d, *J* = 0.9 Hz, 3H).

^{13}C{^{1}H} NMR (101 MHz, CDCl$_3$) δ 182.1, 136.5, 136.2, 130.8, 129.6, 127.8, 122.7, 114.4, 112.2, 21.5.

HRMS (ESI) Calculated for: $[C_{10}H_{10}NO]^+$ 160.0757, found 160.0756.

Data consistent with literature.[12]

5-chloro-1H-indole-2-carbaldehyde (1jb)

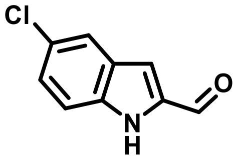

Prepared according to general procedure **B** using (5-chloro-1H-indol-2-yl)methanol (204 g, 1.12 mmol) and MnO_2 (487 mg, 5.6 mmol). From which a 25 g Biotage SNAP KP-Sil column was run on a Biotage Isolera Four purification system (0 – 20% EtOAc in hexanes) to yield titled compound as an off white solid (165 mg, 92%).

^{1}H NMR (400 MHz, CDCl$_3$) δ 9.85 (s, 1H), 9.04 (s, 1H), 7.73 (dt, *J* = 1.8, 0.8 Hz, 1H), 7.39 (dt, *J* = 8.8, 0.8 Hz, 1H), 7.35 (dd, *J* = 8.8, 1.9 Hz, 1H), 7.21 (dd, *J* = 2.2, 0.9 Hz, 1H).

^{13}C{^{1}H} NMR (101 MHz, CDCl$_3$) δ = 182.1, 137.0, 136.2, 128.4, 127.9, 127.1, 122.7, 113.7, 113.7.

HRMS (ESI) Calculated for: $[C_9H_5ClNO]^-$ 178.0065, found 178.0056.

Data consistent with literature.[13]

5-fluoro-1H-indole-2-carbaldehyde (1kb)

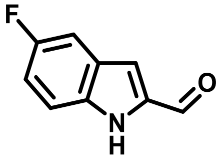

Prepared according to general procedure **B** using (5-fluoro-1H-indol-2-yl)methanol (493 mg, 2.99 mmol) and MnO_2 (1.30 g, 14.9 mmol). From which a 25 g Biotage SNAP KP-Sil column was run on a Biotage Isolera Four purification system (0 – 20% EtOAc in hexanes) to yield titled compound as an off white solid (296 mg, 61%).

^{1}H NMR (400 MHz, $CDCl_3$) δ 9.88 (s, 1H), 9.36 (s, 1H), 7.43 (dddt, *J* = 14.2, 9.0, 2.5, 0.8 Hz, 2H), 7.27 (dd, *J* = 2.2, 1.0 Hz, 1H), 7.19 (td, *J* = 9.0, 2.5 Hz, 1H).

^{13}C{^{1}H} NMR (101 MHz, $CDCl_3$) δ 182.2, 158.3 (d, *J* = 237.9 Hz), 135.9 (d, *J* = 254.2 Hz), 127.5 (d, *J* = 10.3 Hz), 116.8, 116.5, 114.4 (d, *J* = 5.6 Hz), 113.6 (d, *J* = 9.4 Hz), 107.6.

^{19}F NMR (471 MHz, $CDCl_3$) δ -121.98 (m).

HRMS (ESI) Calculated for: $[C_9H_5FNO]^-$ 162.0360, found 162.0362.

Data consistent with literature.[13]

6-methoxy-1H-indole-2-carbaldehyde (1lb)

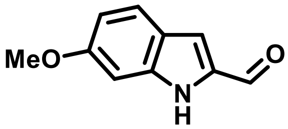

Prepared according to general procedure **B** using (6-methoxy-1H-indol-2-yl)methanol (642 mg, 3.61 mmol) and MnO_2 (1.57 g, 18.1 mmol). From which a 25 g Biotage SNAP KP-Sil column was run on a Biotage Isolera Four purification system (0 – 20% EtOAc in hexanes) to yield titled compound as an off white solid (214 mg, 34%).

^{1}H NMR (500 MHz, $CDCl_3$) δ 9.81 (s, 1H), 9.15 (s, 1H), 7.36 (d, *J* = 9.0 Hz, 1H), 7.20 (dd, *J* = 2.2, 1.0 Hz, 1H), 7.11 (d, *J* = 2.5 Hz, 1H), 7.08 (dd, *J* = 9.0, 2.4 Hz, 1H), 3.86 (s, 3H).

^{13}C{^{1}H} NMR (126 MHz, $CDCl_3$) δ 182.0, 155.1, 136.5, 133.6, 127.8, 119.5, 114.4, 113.6, 102.9, 55.8.

HRMS (ESI) Calculated for: $[C_{10}H_9NNaO_2]^+$ 198.0525, found 198.0521.

Data consistent with literature.[14]

5.3. *Preparations of arylsulfonyl-indole-2-carbaldehydes*

1-((4-nitrophenyl)sulfonyl)-1H-indole-2-carbaldehyde (1a)

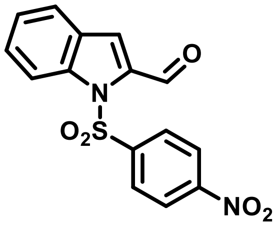

Prepared using general procedure **C** using 1H-indole-2-carbaldehyde (145 mg, 1.00 mmol), DMAP (14 mg, 0.125 mmol), NEt_3 (174 µL, 1.25 mmol) and 4-nitrobenzenesulfonyl chloride (265.0 mg, 1.20 mmol). From which a 25 g Biotage SNAP KP-Sil column was run on a Biotage Isolera Four purification system (0 – 30% EtOAc in hexanes) to yield titled compound as a yellow solid (161 mg, 49%).

^{1}H NMR (400 MHz, $CDCl_3$) δ 10.39 (s, 1H), 8.29 – 8.19 (m, 3H), 7.99 (dt, *J* = 9.0, 2.0 Hz, 2H), 7.66 (dt, *J* = 7.9, 1.0 Hz, 1H), 7.58 (ddd, *J* = 8.5, 7.2, 1.3 Hz, 1H), 7.52 (d, *J* = 0.8 Hz, 1H), 7.38 (ddd, *J* = 8.0, 7.2, 1.0 Hz, 1H).

^{13}C{^{1}H} NMR (101 MHz, $CDCl_3$) δ 182.3, 151.0, 142.8, 138.7, 137.8, 129.7, 128.5, 128.4, 125.7, 124.6, 124.2, 121.5, 115.5.

HRMS (ESI) Calculated for: $[C_{15}H_{10}N_2NaO_5S]^+$ 353.0203, found 353.0200.

Data consistent with literature.[15]

1-((2-fluoro-4-nitrophenyl)sulfonyl)-1H-indole-2-carbaldehyde (1b)

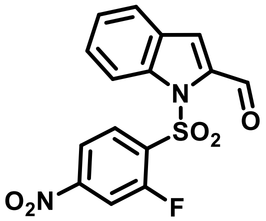

Prepared using general procedure **C** using 1H-indole-2-carbaldehyde (145 mg, 1.00 mmol), DMAP (14 mg, 0.125 mmol), NEt_3 (174 µL, 1.25 mmol) and 2-fluoro-4-nitrobenzenesulfonyl chloride (288 mg, 1.20 mmol). From which a 25 g Biotage SNAP KP-Sil column was run on a Biotage Isolera Four purification system (0 – 30% EtOAc in hexanes) to yield titled compound as a pale yellow solid (181 mg, 52%).

^{1}H NMR (400 MHz, $CDCl_3$) δ 10.19 (s, 1H), 8.37 (dd, *J* = 8.7, 6.8 Hz, 1H), 8.23 – 8.18 (m, 1H), 8.14 (d, *J* = 8.6 Hz, 1H), 7.96 (dd, *J* = 9.4, 2.1 Hz, 1H), 7.73 (d, *J* = 7.9 Hz, 1H), 7.60 – 7.51 (m, 2H), 7.37 (t, *J* = 7.6 Hz, 1H).

^{13}C{^{1}H} NMR (101 MHz, $CDCl_3$) δ 181.4 (d, *J* = 2.1 Hz), 159.2 (d, *J* = 262.8 Hz), 152.1 (d, *J* = 8.4 Hz), 138.7, 137.9, 132.4, 132.0 (d, *J* = 14.1 Hz), 129.5, 127.9, 125.4, 124.1, 121.9, 119.5 (d, *J* = 4.3 Hz), 115.5 (d, *J* = 1.8 Hz), 113.4 (d, *J* = 26.4 Hz).

^{19}F NMR (471 MHz, $CDCl_3$) δ -103.05 – -103.14 (m).

HRMS (ESI) Calculated for: $[C_{15}H_9FN_2NaO_5S]^+$ 371.0108, found 371.0116.

1-((2-methoxy-4-nitrophenyl)sulfonyl)-1H-indole-2-carbaldehyde (1c)

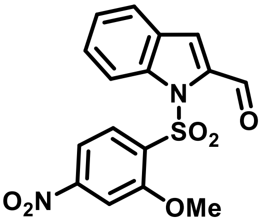

Prepared using general procedure **C** using 1H-indole-2-carbaldehyde (145 mg, 1.00 mmol), DMAP (14 mg, 0.125 mmol), NEt_3 (174 µL, 1.25 mmol) and 2-methoxy-4-nitrobenzenesulfonyl chloride (302 mg, 1.20 mmol). From which a 25 g Biotage SNAP KP-Sil column was run on a Biotage Isolera Four purification system (0 – 30% EtOAc in hexanes) to yield titled compound as an orange gum (126 mg, 35%).

^{1}H NMR (500 MHz, $CDCl_3$) δ 10.45 (s, 1H), 8.35 (d, *J* = 8.7 Hz, 1H), 7.96 (dd, *J* = 8.6, 1.0 Hz, 1H), 7.92 (dd, *J* = 8.7, 2.0 Hz, 1H), 7.69 (d, *J* = 2.1 Hz, 1H), 7.66 (dt, *J* = 7.7, 1.0 Hz, 1H), 7.47 (d, *J* = 0.9 Hz, 1H), 7.45 (ddd, *J* = 8.6, 7.3, 1.3 Hz, 1H), 7.31 (ddd, *J* = 8.1, 7.2, 1.0 Hz, 1H), 3.74 (s, 3H).

^{13}C{^{1}H} NMR (126 MHz, $CDCl_3$) δ 183.6, 157.9, 152.5, 139.2, 138.0, 132.3, 131.2, 128.7, 127.9, 124.9, 123.8, 117.5, 115.2, 114.9, 107.9, 56.7.

HRMS (ESI) Calculated for: $[C_{16}H_{12}N_2NaO_6S]^+$ 383.0308, found 383.0310.

1-((4-nitro-3-(trifluoromethyl)phenyl)sulfonyl)-1H-indole-2-carbaldehyde (1d)

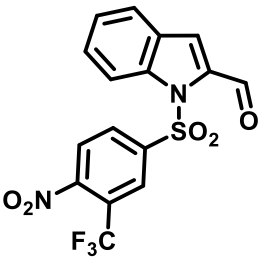

Prepared using general procedure **C** using 1H-indole-2-carbaldehyde (145 mg, 1.00 mmol), DMAP (14 mg, 0.125 mmol), NEt_3 (174 µL, 1.25 mmol) and 4-nitro-3-(trifluoromethyl)benzenesulfonyl chloride (348 mg, 1.20 mmol). From which a 25 g Biotage SNAP KP-Sil column was run on a Biotage Isolera Four purification system (0 – 30% EtOAc in hexanes) to yield titled compound as an orange solid (155 mg, 39%).

^{1}H NMR (400 MHz, $CDCl_3$) δ 10.24 (s, 1H), 8.32 (d, *J* = 2.0 Hz, 1H), 8.23 (d, *J* = 8.6 Hz, 1H), 8.19 (dd, *J* = 8.5, 2.1 Hz, 1H), 7.89 (d, *J* = 8.5 Hz, 1H), 7.71 (d, *J* = 7.9 Hz, 1H), 7.62 (ddd, *J* = 8.6, 7.2, 1.3 Hz, 1H), 7.55 (s, 1H), 7.42 (t, *J* = 7.6 Hz, 1H).

^{13}C{^{1}H} NMR (101 MHz, $CDCl_3$) δ 181.4, 141.8, 138.9, 137.7, 132.1, 130.0, 128.4, 127.2 (d, *J* = 5.3 Hz), 126.1, 125.9, 124.4, 123.1, 115.4.

^{19}F NMR (376 MHz, $CDCl_3$) δ -60.35.

HRMS (ESI) Calculated for: $[C_{16}H_9F_3N_2NaO_5S\ ]^+$ 421.0076, found 421.0097.

1-((2-nitrophenyl)sulfonyl)-1H-indole-2-carbaldehyde (1e)

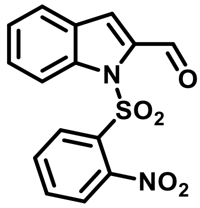

Prepared using general procedure **C** using 1H-indole-2-carbaldehyde (145 mg, 1.00 mmol), DMAP (14 mg, 0.125 mmol), NEt_3 (174 µL, 1.25 mmol) and 2-nitrobenzenesulfonyl chloride (283.0 mg, 1.20 mmol). From which a 25 g Biotage SNAP KP-Sil column was run on a Biotage Isolera Four purification system (0 – 30% EtOAc in hexanes) to yield titled compound as an orange solid (147 mg, 41%).

^{1}H NMR (500 MHz, $CDCl_3$) δ 10.16 (s, 1H), 8.05 (d, *J* = 8.6 Hz, 1H), 7.93 – 7.89 (m, 1H), 7.80 (dd, *J* = 7.9, 1.5 Hz, 1H), 7.78 – 7.74 (m, 1H), 7.73 (d, *J* = 8.3 Hz, 1H), 7.68 (td, *J* = 7.8, 1.5 Hz, 1H), 7.59 (s, 1H), 7.56 – 7.50 (m, 1H), 7.37 (t, *J* = 7.6 Hz, 1H).

^{13}C{^{1}H} NMR (126 MHz, $CDCl_3$) δ 181.7, 147.8, 138.9, 138.5, 135.2, 132.6, 130.5, 129.4, 127.7, 125.4, 125.2, 124.0, 120.7, 115.5.

HRMS (ESI) Calculated for: $[C_{15}H_{10}N_2NaO_5S]^+$ 353.0203, found 353.02110.

Data consistent with literature.[16]

1-((4-bromo-2-nitrophenyl)sulfonyl)-1H-indole-2-carbaldehyde (1f)

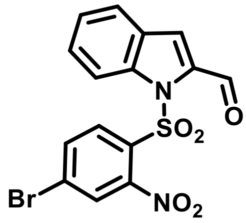

Prepared using general procedure **C** using 1H-indole-2-carbaldehyde (145 mg, 1.00 mmol), DMAP (14 mg, 0.125 mmol), NEt_3 (174 µL, 1.25 mmol) and 2-nitro-4-bromobenzenesulfonyl chloride (300.0 mg, 1.20 mmol). From which a 25 g Biotage SNAP KP-Sil column was run on a Biotage Isolera Four purification system (0 – 30% EtOAc in hexanes) to yield titled compound as a yellow solid (24 mg, 6%).

^{1}H NMR (500 MHz, $CDCl_3$) δ 10.07 (s, 1H), 8.05 (dd, *J* = 8.7, 0.9 Hz, 1H), 7.92 (d, *J* = 2.0 Hz, 1H), 7.87 (d, *J* = 8.6 Hz, 1H), 7.82 (dd, *J* = 8.6, 1.9 Hz, 1H), 7.74 (dt, *J* = 8.0, 1.0 Hz, 1H), 7.59 (d, *J* = 0.9 Hz, 1H), 7.55 (ddd, *J* = 8.6, 7.2, 1.3 Hz, 1H), 7.41 – 7.37 (m, 1H).

^{13}C{^{1}H} NMR (126 MHz, $CDCl_3$) δ 181.2, 148.0, 139.2, 138.3, 135.6, 132.1, 131.5, 129.7, 129.6, 128.3, 127.7, 125.3, 124.1, 122.0, 115.6.

HRMS (ESI) Calculated for: $[C_{15}H_9BrN_2NaO_5S]^+$ 430.9321, found 430.9308.

1-(pyridin-2-ylsulfonyl)-1H-indole-2-carbaldehyde (1g)

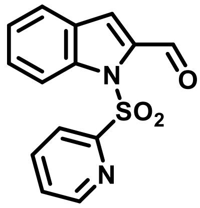

NaOCl (10-15%bw, 17 mL) was added dropwise 2-mecaptopyridine (222 mg, 2 mmol) in H_2SO_4 (Conc. 8 mL) at 0 °C under N_2. Reaction was stirred at this temperature for 1 hour, H_2O (30 mL) added followed by DCM (20 mL). Organics collected and aqueous extracted with DCM (2 x 20 mL), combined organics were washed with brine, dried over $MgSO_4$ and filtered. Volitiles removed *in vacuo* and crude redissolved in DCM (10 mL) to which 1H-indole-2-carbaldehyde (145 mg, 1 mmol), DMAP (14 mg, 0.13 mmol) and NEt_3 (174 µL, 1.25 mmol) were added. Reaction was stirred whilst warming to room temperature for 16 $hours_4Cl$ added followed by DCM (20 mL), organics collected and aqueous extracted with DCM (2 x 20 mL). Combined organics were washed with brine and dried over $MgSO_4$ before dry loading onto silica from which a 25 g Biotage SNAP KP-Sil column was run on a Biotage Isolera Four purification system (0 – 30% EtOAc in hexanes) to yield titled compound as an off white solid (139 mg, 48%).

^{1}H NMR (500 MHz, $CDCl_3$) δ 10.58 (s, 1H), 8.56 (ddd, *J* = 4.7, 1.8, 0.9 Hz, 1H), 8.23 – 8.17 (m, 1H), 8.16 (dt, *J* = 7.8, 1.1 Hz, 1H), 7.91 (td, *J* = 7.8, 1.7 Hz, 1H), 7.65 (dt, *J* = 7.9, 1.0 Hz, 1H), 7.52 (d, *J* = 0.8 Hz, 1H), 7.51 – 7.43 (m, 2H), 7.31 (ddd, *J* = 8.0, 7.1, 1.0 Hz, 1H).

^{13}C{^{1}H} NMR (126 MHz, $CDCl_3$) δ 183.8, 155.3, 150.6, 138.6, 138.4, 138.4, 128.6, 128.1, 128.0, 124.8, 123.6, 122.3, 117.9, 115.3.

HRMS (ESI) Calculated for: $[C_{14}H_{10}N_2NaO_3S]^+$ 309.0304, found 309.0303.

Data consistent with literature.[17]

3-methyl-1-((4-nitrophenyl)sulfonyl)-1H-indole-2-carbaldehyde (1h)

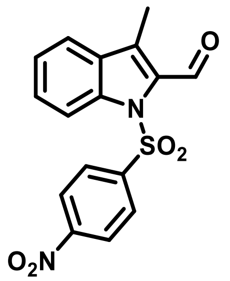

Prepared using general procedure **C** using 3-methyl-1H-indole-2-carbaldehyde (270 mg, 1.70 mmol), DMAP (26 mg, 0.21 mmol), NEt_3 (296 µL, 2.13 mmol) and 4-nitrobenzenesulfonyl chloride (452 mg, 2.04 mmol). From which a 25 g Biotage SNAP KP-Sil column was run on a Biotage Isolera Four purification system (0 – 30% EtOAc in hexanes) to yield titled compound as a yellow solid (43 mg, 33%).

^{1}H NMR (500 MHz, $CDCl_3$) δ 10.52 (s, 1H), 8.22 – 8.15 (m, 3H), 7.89 – 7.83 (m, 2H), 7.62 – 7.54 (m, 2H), 7.37 (t, *J* = 7.6 Hz, 1H), 2.52 (s, 3H).

$^{13}C\{^1H\}$ **NMR** (126 MHz, $CDCl_3$) δ 184.4, 150.8, 142.1, 137.5, 134.2, 132.9, 131.0, 129.9, 128.3, 125.6, 124.4, 122.2, 115.9, 10.6.
HRMS (ESI) Calculated for: $[C_{16}H_{12}N_2NaO_5S]^+$ 367.0359, found 367.0366.
Data consistent with literature.[18]

5-methyl-1-((4-nitrophenyl)sulfonyl)-1H-indole-2-carbaldehyde (1i)

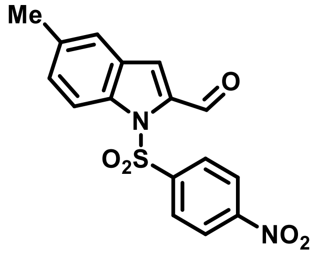

Prepared using general procedure **C** using 5-methyl-1H-indole-2-carbaldehyde (242 mg, 1.48 mmol), DMAP (23 mg, 0.19 mmol), NEt_3 (258 µL, 1.85 mmol) and 4-nitrobenzenesulfonyl chloride (396.0 mg, 1.78 mmol). From which a 25 g Biotage SNAP KP-Sil column was run on a Biotage Isolera Four purification system (0 – 30% EtOAc in hexanes) to yield titled compound as a yellow solid (258 mg, 51%).
^1H **NMR** (400 MHz, $CDCl_3$) δ 10.37 (s, 1H), 8.27 – 8.20 (m, 2H), 8.08 (d, *J* = 8.6 Hz, 1H), 7.99 – 7.92 (m, 2H), 7.47 – 7.35 (m, 3H), 2.43 (s, 3H).
$^{13}C\{^1H\}$ **NMR** (101 MHz, $CDCl_3$) δ 182.4, 150.9, 142.7, 137.9, 137.0, 135.7, 131.4, 128.8, 128.3, 124.6, 123.8, 121.3, 115.1, 21.3.
HRMS (ESI) Calculated for: $[C_{16}H_{12}N_2NaO_5S]^+$ 367.0359, found 367.0360.

5-chloro-1-((4-nitrophenyl)sulfonyl)-1H-indole-2-carbaldehyde (1j)

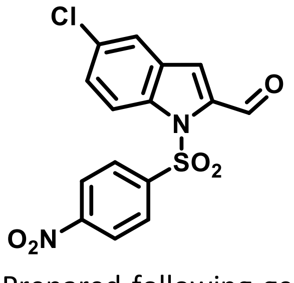

Prepared following general procedure **C** using 5-chloro-1H-indole-2-carbaldehyde (150 mg, 0.83 mmol), DMAP (13 mg, 0.10 mmol), Et_3N (145 µL, 1.04 mmol), 4-nitrobenzenesulfonyl chloride (222 mg, 1.00 mmol). From which a 25 g Biotage SNAP KP-Sil column was run on a Biotage Isolera Four purification system (0 – 30% EtOAc in hexanes) to yield titled compound as a yellow solid (154 mg, 92%).
^1H **NMR** (400 MHz, $CDCl_3$) δ 10.37 (s, 1H), 8.32 – 8.24 (m, 2H), 8.17 (dt, *J* = 9.1, 0.8 Hz, 1H), 8.02 – 7.95 (m, 2H), 7.64 (d, *J* = 2.1 Hz, 1H), 7.53 (dd, *J* = 9.0, 2.1 Hz, 1H), 7.44 (d, *J* = 0.8 Hz, 1H).
$^{13}C\{^1H\}$ **NMR** (101 MHz, $CDCl_3$) δ 182.0, 151.1, 142.6, 138.7, 136.9, 131.6, 129.9, 129.6, 128.4, 124.8, 123.5, 120.1, 116.6.
HRMS (ESI) Calculated for: $[C_{15}H_{10}O_5N_2ClS]^+$ 364.9993, found 364.9994.

IR (neat, cm^{-1}) 2980, 2889, 1700, 1527, 1473, 1382, 1251, 1152.

5-fluoro-1-((4-nitrophenyl)sulfonyl)-1H-indole-2-carbaldehyde (1k)

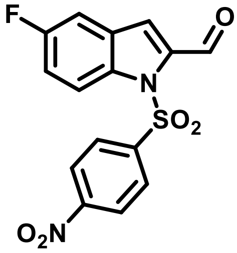

Prepared using general procedure **C** using 5-fluoro-1H-indole-2-carbaldehyde (296 mg, 1.82 mmol), DMAP (28 mg, 0.23 mmol), NEt_3 (317 µL, 2.18 mmol) and 4-nitrobenzenesulfonyl chloride (485.0 mg, 2.18 mmol). From which a 25 g Biotage SNAP KP-Sil column was run on a Biotage Isolera Four purification system (0 – 30% EtOAc in hexanes) to yield titled compound as a yellow solid (269 mg, 41%).

^{1}H NMR (400 MHz, $CDCl_3$) δ 10.38 (s, 1H), 8.30 – 8.25 (m, 2H), 8.24 – 8.15 (m, 1H), 8.00 – 7.93 (m, 2H), 7.46 (s, 1H), 7.36 – 7.28 (m, 2H).

^{13}C{^{1}H} NMR (101 MHz, $CDCl_3$) δ 182.1, 160.5 (d, *J* = 245.0 Hz), 151.1, 142.6, 139.0, 134.9, 129.5 (d, *J* = 10.2 Hz), 128.3, 124.7, 120.6 (d, *J* = 4.4 Hz), 118.1 (d, *J* = 25.9 Hz), 116.9 (d, *J* = 9.2 Hz), 109.3 (d, *J* = 23.9 Hz).

^{19}F NMR (471 MHz, $CDCl_3$) δ -115.70

HRMS (ESI) Calculated for: $[C_{15}H_9FN_2NaO_5S]^+$ 371.0108, found 371.0117.

6-methoxy-1-((4-nitrophenyl)sulfonyl)-1H-indole-2-carbaldehyde (1l)

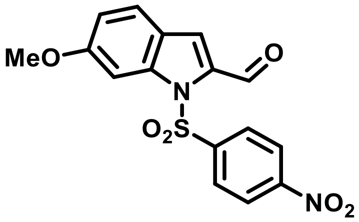

Prepared using general procedure **C** using 5-methoxy-1H-indole-2-carbaldehyde (390 mg, 2.23 mmol), DMAP (34 mg, 0.28 mmol), NEt_3 (388 µL, 2.79 mmol) and 4-nitrobenzenesulfonyl chloride (594 mg, 2.67 mmol). From which a 25 g Biotage SNAP KP-Sil column was run on a Biotage Isolera Four purification system (0 – 30% EtOAc in hexanes) to yield titled compound as a yellow solid (440 mg, 55%).

^{1}H NMR (400 MHz, $CDCl_3$) δ 10.38 (s, 1H), 8.27 – 8.20 (m, 2H), 8.10 (dt, *J* = 9.3, 0.7 Hz, 1H), 7.97 – 7.89 (m, 2H), 7.43 (d, *J* = 0.8 Hz, 1H), 7.18 (dd, *J* = 9.2, 2.6 Hz, 1H), 7.01 (d, *J* = 2.5 Hz, 1H), 3.83 (s, 3H).

^{13}C{^{1}H} NMR (101 MHz, $CDCl_3$) δ 182.5, 157.9, 150.9, 142.5, 138.3, 133.2, 129.7, 128.2, 124.6, 121.2, 119.8, 116.6, 104.8, 55.8.

HRMS (ESI) Calculated for: $[C_{16}H_{12}N_2NaO_6S]^+$ 383.0316, found 383.0308.

IR (neat, cm^{-1}) 2980, 2888, 1703, 1604, 1528, 1474, 1379.

6-bromo-1-((4-nitrophenyl)sulfonyl)-1H-indole-2-carbaldehyde (1m)

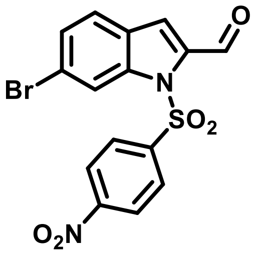

Prepared using general procedure **C** using 6-bromo-1H-indole-2-carbaldehyde (412 mg, 1.84 mmol), DMAP (28 mg, 0.23 mmol), NEt_3 (320 µL, 2.30 mmol) and 4-nitrobenzenesulfonyl chloride (490 mg, 2.21 mmol). From which a 25 g Biotage SNAP KP-Sil column was run on a Biotage Isolera Four purification system (0 – 30% EtOAc in hexanes) to yield titled compound as an orange solid (425 mg, 56%).

^{1}H NMR (500 MHz, $CDCl_3$) δ 10.33 (s, 1H), 8.44 (dt, *J* = 1.6, 0.8 Hz, 1H), 8.34 – 8.28 (m, 2H), 8.06 – 7.99 (m, 2H), 7.57 – 7.48 (m, 2H), 7.47 (d, *J* = 0.8 Hz, 1H).

^{13}C{^{1}H} NMR (126 MHz, $CDCl_3$) δ 181.7, 151.1, 142.8, 139.2, 137.9, 129.3, 128.5, 127.1, 125.1, 124.8, 123.8, 121.0, 118.5.

HRMS (ESI) Calculated for: $[C_{15}H_{10}BrN_2O_5S]^+$ 408.9488, found 408.94920.

6-chloro-1-((4-nitrophenyl)sulfonyl)-1H-indole-2-carbaldehyde (1n)

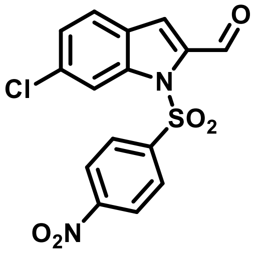

Prepared using general procedure **C** using 5-chloro-1H-indole-2-carbaldehyde (179 mg, 1.00 mmol), DMAP (14 mg, 0.125 mmol), NEt_3 (174 µL, 1.25 mmol) and 4-nitrobenzenesulfonyl chloride (265.0 mg, 1.20 mmol). From which a 25 g Biotage SNAP KP-Sil column was run on a Biotage Isolera Four purification system (0 – 30% EtOAc in hexanes) to yield titled compound as an orange solid (188 mg, 52%).

^{1}H NMR (400 MHz, $CDCl_3$) δ 10.32 (s, 1H), 8.35 – 8.28 (m, 2H), 8.28 – 8.24 (m, 1H), 8.07 – 7.99 (m, 2H), 7.60 (d, *J* = 8.4 Hz, 1H), 7.47 (s, 1H), 7.36 (dd, *J* = 8.5, 1.8 Hz, 1H).

^{13}C{^{1}H} NMR (101 MHz, $CDCl_3$) δ 181.5, 151.0, 142.7, 138.9, 138.0, 135.7, 128.3, 126.7, 126.5, 124.8, 124.7, 120.8, 115.5.

HRMS (ESI) Calculated for: $[C_{15}H_{10}ClN_2O_5S]^+$ 365.0006, found 365.0006.

5.4. Preparations of 2-aroylindoles

(1H-indol-2-yl)(4-nitrophenyl)methanone (2a)

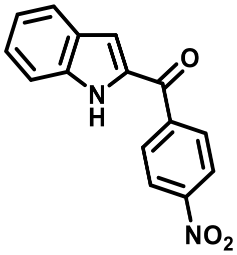

Prepared using general procedure **D** using 1-((4-nitrophenyl)sulfonyl)-1H-indole -2-carbaldehyde (33.0 mg, 0.1 mmol). From which a 10 g Biotage SNAP KP-Sil column was run on a Biotage Isolera Four purification system (0 – 20% EtOAc in hexanes) to yield titled compound as a yellow solid (20 mg, 75 %).

^{1}H NMR (400 MHz, $CDCl_3$) δ 9.32 (s, 1H), 8.39 (dt, *J* = 8.8, 2.0 Hz, 2H), 8.12 (dt, *J* = 8.9, 2.0 Hz, 2H), 7.74 (dq, *J* = 8.1, 1.0 Hz, 1H), 7.50 (dd, *J* = 8.4, 1.0 Hz, 1H), 7.43 (ddd, *J* = 8.3, 6.9, 1.1 Hz, 1H), 7.20 (ddd, *J* = 8.0, 6.9, 1.0 Hz, 1H), 7.14 (dd, *J* = 2.2, 1.0 Hz, 1H).

^{13}C{^{1}H} NMR (101 MHz, $CDCl_3$) δ 185.2, 150.0, 143.3, 138.1, 133.8, 130.2, 127.8, 127.6, 123.9, 123.7, 121.7, 113.9, 112.4.

HRMS (ESI) Calculated for: $[C_{15}H_9O_3N_2]^-$ 265.0619, found 265.0611.

Data consistent with literature.[19]

(1H-indol-2-yl)(4-nitro-3-(trifluoromethyl)phenyl)methanone (2b)

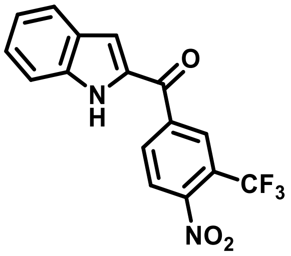

Prepared using general procedure **D** using 1-((4-nitro-3-(trifluoromethyl)phenyl) sulfonyl)-1H-indole-2-carbaldehyde (39.8 mg, 0.1 mmol). From which a 10 g Biotage SNAP KP-Sil column was run on a Biotage Isolera Four purification system (0 – 25% EtOAc in hexanes) to yield titled compound as a yellow solid (27 mg, 81%).

^{1}H NMR (500 MHz, $CDCl_3$) δ 9.36 – 9.29 (m, 1H), 8.40 (d, *J* = 1.8 Hz, 1H), 8.30 (dd, *J* = 8.2, 1.8 Hz, 1H), 8.03 (d, *J* = 8.2 Hz, 1H), 7.75 (dd, *J* = 8.1, 1.0 Hz, 1H), 7.51 (dd, *J* = 8.4, 1.0 Hz, 1H), 7.45 (ddd, *J* = 8.3, 6.9, 1.1 Hz, 1H), 7.22 (ddd, *J* = 8.0, 6.8, 1.0 Hz, 1H), 7.13 (dd, *J* = 2.2, 1.0 Hz, 1H).

^{13}C{^{1}H} NMR (126 MHz, $CDCl_3$) δ 183.4, 149.9, 141.6, 138.3, 133.6, 133.2, 128.9 (q, *J* = 5.2 Hz), 128.0, 127.8, 125.4, 123.8, 121.8, 114.1, 112.5.

^{19}F NMR (471 MHz, $CDCl_3$) δ -60.01

HRMS (ESI) Calculated for: $[C_{16}H_8O_3N_2F_3]^-$ 333.0493, found 333.0489.

(2-fluoro-4-nitrophenyl)(1H-indol-2-yl)methanone (2c)

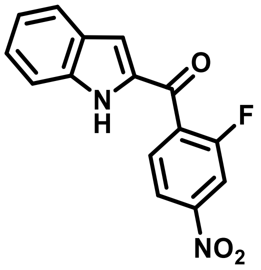

Prepared using general procedure **D** using 1-((2-fluoro-4-nitrophenyl)sulfonyl)-1H-indole-2-carbaldehyde (32 mg, 0.1 mmol). From which a 10 g Biotage SNAP KP-Sil column was run on a Biotage Isolera Four purification system (0 – 25% EtOAc in hexanes) to yield titled compound as a yellow solid (15 mg, 59%).

^{1}H NMR (400 MHz, $CDCl_3$) δ 9.29 (s, 1H), 8.18 (ddd, *J* = 8.4, 2.1, 0.8 Hz, 1H), 8.11 (dd, *J* = 9.0, 2.1 Hz, 1H), 7.85 (dd, *J* = 8.4, 6.6 Hz, 1H) , 7.70 (dd, *J* = 8.2, 1.0 Hz, 1H , 7.49 (dq, *J* = 8.4, 1.1 Hz, 1H), 7.43 (ddd, *J* = 8.4, 6.8, 1.1 Hz, 1H), 7.19 (ddd, *J* = 8.0, 6.8, 1.1 Hz, 1H), 7.04 – 7.00 (m, 1H).

^{13}C{^{1}H} NMR (101 MHz, $CDCl_3$) δ 181.7, 159.6 (d, *J* = 257.8 Hz, CF_{Ar}), 150.1, 138.4, 134.2, 132.5 (d, *J* = 16.0 Hz), 131.2 (d, *J* = 3.4 Hz), 127.9, 127.5, 123.6, 121.7, 119.2 (d, *J* = 4.0 Hz), 114.5 (d, *J* = 2.6 Hz), 112.6, 112.5.

^{19}F NMR (471 MHz, $CDCl_3$) δ -108.44 – -108.57 (m).

HRMS (ESI) Calculated for: $[C_{15}H_8FN_2O_3]^-$ 283.0524, found 283.0517.

IR (neat, cm^{-1}) 3344, 2922, 2852, 2165, 2034,1637, 1340, 743.

(1H-indol-2-yl)(2-methoxy-4-nitrophenyl)methanone (2d)

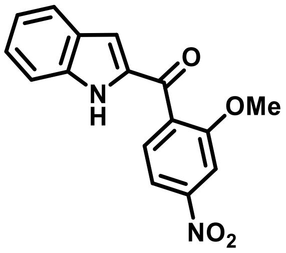

Prepared using general procedure **D** using 1-((2-methoxy-4-nitrophenyl)sulfonyl)-1H-indole-2-carbaldehyde (36 mg, 0.1 mmol). From which a 10 g Biotage SNAP KP-Sil column was run on a Biotage Isolera Four purification system (0 – 25% EtOAc in hexanes) to yield titled compound as a yellow solid (11 mg, 37%).

^{1}H NMR (500 MHz, $CDCl_3$) δ 9.29 (s, 1H), 7.95 (dd, *J* = 8.2, 2.0 Hz, 1H), 7.88 (d, *J* = 2.0 Hz, 1H), 7.66 (dd, *J* = 8.1, 1.0 Hz, 1H), 7.62 (d, *J* = 8.2 Hz, 1H), 7.48 (dq, *J* = 8.5, 1.0 Hz, 1H), 7.40 (ddd, *J* = 8.2, 6.9, 1.1 Hz, 1H), 7.16 (ddd, *J* = 7.9, 6.9, 1.0 Hz, 1H), 6.87 (dd, *J* = 2.2, 1.0 Hz, 1H), 3.93 (s, 3H).

^{13}C{^{1}H} NMR (126 MHz, $CDCl_3$) δ 185.1, 158.1, 150.2, 138.2, 135.0, 134.0, 129.9, 127.6, 127.5, 123.6, 121.5, 115.5, 114.0, 112.5, 106.8, 56.6.

HRMS (ESI) Calculated for: $[C_{16}H_{12}N_2NaO_4]^+$ 319.0689, found 319.0692

(1H-indol-2-yl)(2-nitrophenyl)methanone (2e)

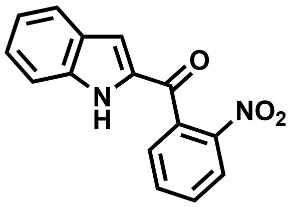

Prepared using general procedure **D** using 1-((2-nitrophenyl)sulfonyl)-1H-indole-2-carbaldehyde (33.0 mg, 0.1 mmol). From which a 10 g Biotage SNAP KP-Sil column was run on a Biotage Isolera Four purification system (0 – 20% EtOAc in hexanes) to yield titled compound as a yellow solid (19 mg, 71 %).

^{1}H NMR (500 MHz,$CDCl_3$) δ 9.32 (s, 1H), 8.22 (dd, *J* = 8.2, 1.2 Hz, 1H), 7.79 (td, *J* = 7.5, 1.2 Hz, 1H), 7.71 (td, *J* = 7.8, 1.5 Hz, 1H), 7.65 (dd, *J* = 7.4, 1.5 Hz, 1H), 7.61 (dd, *J* = 8.1, 1.0 Hz, 1H), 7.48 (dd, *J* = 8.4, 1.0 Hz, 1H), 7.39 (ddd, *J* = 8.3, 7.0, 1.2 Hz, 1H), 7.15 (ddd, *J* = 8.0, 6.9, 1.0 Hz, 1H), 6.73 (dd, *J* = 2.2, 0.9 Hz, 1H).

^{13}C{^{1}H} NMR (126 MHz, $CDCl_3$) δ 184.7, 147.5, 138.2, 135.1, 134.5, 133.8, 131.1, 129.5, 127.6, 127.3, 124.8, 123.5, 121.5, 112.7, 112.5.

HRMS (ESI) Calculated for: [$C_{15}H_{10}N_2O_3Na$] 289.0584, found 289.0596.

Data consistent with literature.[20]

(4-bromo-2-nitrophenyl)(1H-indol-2-yl)methanone (2f)

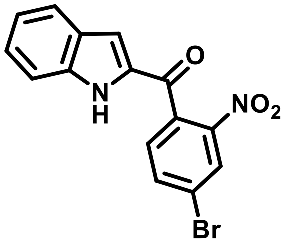

Prepared using general procedure **D** using 1-((4-bromo-2-nitrophenyl)sulfonyl)-1H-indole-2-carbaldehyde (38 mg, 0.1 mmol). From which a 10 g Biotage SNAP KP-Sil column was run on a Biotage Isolera Four purification system (0 – 25% EtOAc in hexanes) to yield titled compound as a green solid (7 mg, 20%).

^{1}H NMR (400 MHz, $CDCl_3$) δ 9.26 (s, 1H), 8.35 (d, *J* = 1.8 Hz, 1H), 7.91 (dd, *J* = 8.1, 1.8 Hz, 1H), 7.62 (dd, *J* = 8.2, 1.1 Hz, 1H), 7.53 (d, *J* = 8.1 Hz, 1H), 7.48 (dt, *J* = 8.4, 0.9 Hz, 1H), 7.40 (ddd, *J* = 8.3, 7.0, 1.1 Hz, 1H), 7.16 (ddd, *J* = 8.1, 7.0, 1.1 Hz, 1H), 6.74 (dd, *J* = 2.2, 1.0 Hz, 1H).

^{13}C{^{1}H} NMR (101 MHz, $CDCl_3$) δ 183.6, 138.3, 136.8, 134.2, 133.7, 130.7, 127.9, 127.6, 124.7, 124.3, 123.4, 121.5, 112.8, 112.4.

HRMS (ESI) Calculated for: [$C_{15}H_9BrN_2NaO_3$]$^+$ 366.9689, found 366.9697.

IR (neat, cm^{-1}) 3150, 2981, 1670, 1580, 1446, 1406, 1362,1172.

(1H-indol-2-yl)(pyridin-2-yl)methanone (2g)

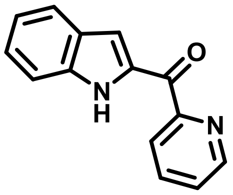

Prepared according to general procedure **D** using 1-(pyridin-2-ylsulfonyl)-1H-indole-2-carbaldehyde (29 mg, 0.1 mmol). From which a 10 g Biotage SNAP KP-Sil column was run on a Biotage Isolera Four purification system (0 – 20% EtOAc in hexanes) to yield titled compound as an off-white solid (5 mg, 23%).

^{1}H NMR (500 MHz, $CDCl_3$) δ 10.85 (s, 1H), 8.81 (ddd, *J* = 4.8, 1.8, 0.9 Hz, 1H), 8.29 (dt, *J* = 7.9, 1.1 Hz, 1H), 7.94 (td, *J* = 7.7, 1.8 Hz, 1H), 7.85 (dd, *J* = 2.0, 1.1 Hz, 1H), 7.77 (dt, *J* = 8.1, 1.0 Hz, 1H), 7.54 (ddd, *J* = 7.6, 4.7, 1.2 Hz, 1H), 7.50 (dq, *J* = 8.4, 1.0 Hz, 1H), 7.37 (ddd, *J* = 8.2, 7.0, 1.1 Hz, 1H), 7.16 (ddd, *J* = 8.0, 6.9, 0.9 Hz, 1H).

^{13}C{^{1}H} NMR (126 MHz, $CDCl_3$) δ 181.3, 155.2, 148.6, 137.8, 137.5, 135.2, 127.6, 126.9, 126.5, 124.4, 123.5, 121.0, 113.5, 112.5.

HRMS (ESI) Calculated for: $[C_{14}H_{10}N_2NaO]^+$ 245.0685, found 245.0686.

Data consistent with literature.[21]

(3-methyl-1H-indol-2-yl)(4-nitrophenyl)methanone (2h)

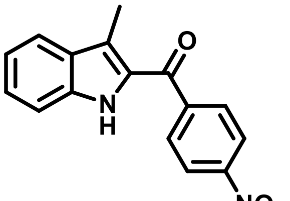

Prepared using general procedure **D** using 3-methyl-1-((4-nitrophenyl)sulfonyl)-1H-indole-2-carbaldehyde (34.0 mg, 0.1 mmol). From which a 10 g Biotage SNAP KP-Sil column was run on a Biotage Isolera Four purification system (0 – 20% EtOAc in hexanes) to yield titled compound as a yellow solid (18 mg, 66 %).

^{1}H NMR (500 MHz, $CDCl_3$) δ 8.87 (s, 1H), 8.42 – 8.34 (m, 2H), 7.93 – 7.88 (m, 2H), 7.68 (dd, *J* = 8.2, 1.0 Hz, 1H), 7.44 – 7.40 (m, 2H), 7.19 (ddd, *J* = 8.0, 4.8, 3.1 Hz, 1H), 2.22 (s, 3H).

^{13}C{^{1}H} NMR (126 MHz, $CDCl_3$) δ 187.1, 149.8, 145.1, 137.1, 131.1, 129.7, 129.1, 127.7, 124.0, 121.9, 121.7, 120.9, 112.1, 11.6.

HRMS (ESI) Calculated for: $[C_{16}H_{11}N_2O_3]^-$ 279.0775, found 279.0765.

Data consistent with literature.[22]

(5-methyl-1H-indol-2-yl)(4-nitrophenyl)methanone (2i)

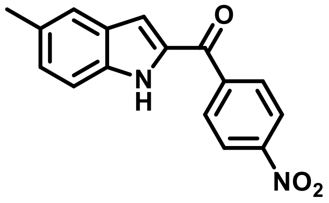

Prepared using general procedure **D** using 5-methyl-1-((4-nitrophenyl)sulfonyl)-1H-indole-2-carbaldehyde (34.0 mg, 0.1 mmol). From which a 10 g Biotage SNAP KP-Sil column was run on a Biotage Isolera Four purification system (0 – 20% EtOAc in hexanes) to yield titled compound as a yellow solid (18 mg, 64 %).

^{1}H NMR (400 MHz, DMSO) δ 12.00 (s, 1H), 8.43 – 8.37 (m, 2H), 8.16 – 8.09 (m, 2H), 7.48 (s, 1H), 7.41 (d, *J* = 8.4 Hz, 1H), 7.19 (dd, *J* = 8.6, 1.6 Hz, 1H), 7.06 (dd, *J* = 2.2, 0.9 Hz, 1H), 2.37 (s, 3H).

^{13}C{^{1}H} NMR (101 MHz, DMSO) δ 184.9, 149.3, 143.5, 137.1, 133.9, 130.2, 129.5, 128.6, 127.3, 123.8, 122.1, 112.9, 112.7, 21.2.

HRMS (ESI) Calculated for: $[C_{16}H_{13}N_2O_3]^+$ 281.0926, found 281.0932.

(5-chloro-1H-indol-2-yl)(4-nitrophenyl)methanone (2j)

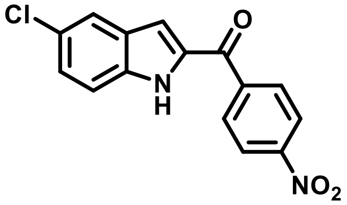

Prepared using general procedure **D** using 5-chloro-1-((4-nitrophenyl)sulfonyl)-1H-indole-2-carbaldehyde (37 mg, 0.1 mmol). From which a 10 g Biotage SNAP KP-Sil column was run on a Biotage Isolera Four purification system (0 – 20% EtOAc in hexanes) to yield titled compound as a yellow solid (16 mg, 53 %).

^{1}H NMR (400 MHz, DMSO) δ 12.32 (s, 1H), 8.45 – 8.37 (m, 2H), 8.18 – 8.11 (m, 2H), 7.80 (d, *J* = 2.1 Hz, 1H), 7.53 (dt, *J* = 8.8, 0.8 Hz, 1H), 7.36 (dd, *J* = 8.8, 2.1 Hz, 1H), 7.15 (dd, *J* = 2.2, 0.9 Hz, 1H).

^{13}C{^{1}H} NMR (101 MHz, DMSO) δ 185.2, 149.5, 143.0, 136.8, 135.0, 130.3, 127.9, 126.5, 125.1, 123.8, 122.1, 114.6, 112.5.

HRMS (ESI) Calculated for: $[C_{15}H_8ClN_2O_3]^-$ 299.0229, found 299.0230.

(5-fluoro-1H-indol-2-yl)(4-nitrophenyl)methanone (2k)

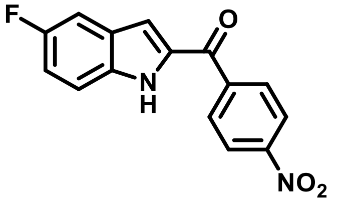

Prepared using general procedure **D** using 5-fluoro-1-((4-nitrophenyl)sulfonyl)-1H-indole-2-carbaldehyde (35.0 mg, 0.1 mmol). From which a 10 g Biotage SNAP KP-Sil column was run on a Biotage Isolera Four purification system (0 – 20% EtOAc in hexanes) to yield titled compound as a yellow solid (13 mg, 46 %).

^{1}H NMR (400 MHz, DMSO) δ 12.23 (s, 1H), 8.44 – 8.34 (m, 2H), 8.18 – 8.10 (m, 2H), 7.57 – 7.42 (m, 2H), 7.23 (td, *J* = 9.2, 2.6 Hz, 1H), 7.18 – 7.12 (m, 1H).

^{13}C{^{1}H} NMR (101 MHz, DMSO) δ 185.1, 158.6, 156.2, 149.5, 143.1, 135.3, 130.3, 127.0 (d, *J* = 10.9 Hz), 123.8, 115.6 (d, *J* = 26.9 Hz), 114.4 (d, *J* = 9.7 Hz), 113.0 (d, *J* = 6.0 Hz), 106.9 (d, *J* = 23.0 Hz).

^{19}F NMR (376 MHz, DMSO) δ -122.45 (td, *J* = 9.7, 4.7 Hz).

HRMS (ESI) Calculated for: $[C_{15}H_8FN_2O_3]^-$ 283.0524, found 283.0520.

(6-methoxy-1H-indol-2-yl)(4-nitrophenyl)methanone (2l)

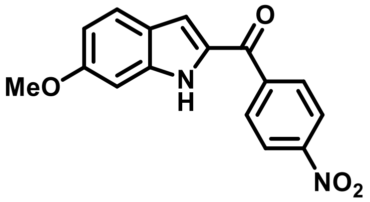

Prepared using general procedure **D** using 6-methoxy-1-((4-nitrophenyl)sulfonyl)-1H-indole-2-carbaldehyde (36.0 mg, 0.1 mmol). From which a 10 g Biotage SNAP KP-Sil column was run on a Biotage Isolera Four purification system (0 – 20% EtOAc in hexanes) to yield titled compound as an orange solid (18 mg, 62 %).

^{1}H NMR (400 MHz, $CDCl_3$) δ 9.25 (s, 1H) , 8.42 – 8.30 (m, 2H), 8.15 – 8.07 (m, 2H), 7.39 (dt, *J* = 8.8, 0.9 Hz, 1H), 7.14 – 7.07 (m, 2H), 7.04 (dd, *J* = 2.2, 1.0 Hz, 1H), 3.86 (s, 3H).

^{13}C{^{1}H} NMR (101 MHz, $CDCl_3$) δ 184.9, 155.3, 150.0, 143.4, 134.1, 133.7, 130.2, 128.2, 123.8, 119.7, 113.4, 113.3, 102.8, 55.8.

HRMS (ESI) Calculated for: $[C_{16}H_{11}N_2O_4]^-$ 295.0724, found 295.0719.

(6-bromo-1H-indol-2-yl)(4-nitrophenyl)methanone (2m)

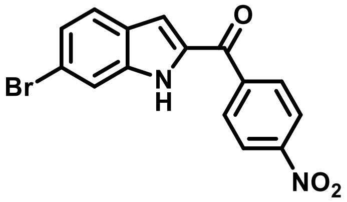

Prepared using general procedure **D** using 6-bromo-1-((4-nitrophenyl)sulfonyl)-1H-indole-2-carbaldehyde (41.0 mg, 0.1 mmol). From which a 10 g Biotage SNAP KP-Sil column was run on a Biotage Isolera Four purification system (0 – 20% EtOAc in hexanes) to yield titled compound as a yellow solid (16 mg, 46 %).

^{1}H NMR (400 MHz, DMSO) δ 12.24 (s, 1H), 8.42 – 8.37 (m, 2H), 8.18 – 8.11 (m, 2H), 7.73 – 7.67 (m, 2H), 7.26 (dd, *J* = 8.5, 1.8 Hz, 1H), 7.20 (d, *J* = 1.0 Hz, 1H).

^{13}C{^{1}H} NMR (101 MHz, DMSO) δ 185.1, 149.5, 143.0, 139.1, 134.5, 130.3, 126.0, 125.1, 123.9, 123.8, 119.4, 115.3, 113.4.

HRMS (ESI) Calculated for: $[C_{15}H_{10}BrN_2O_3]^+$ 344.9869, found 344.9882.

(6-chloro-1H-indol-2-yl)(4-nitrophenyl)methanone (2n)

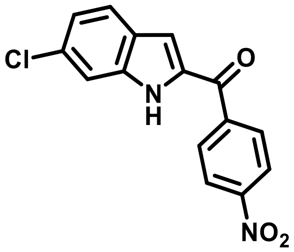

Prepared according to general procedure **D** using 6-chloro-1-((4-nitrophenyl) sulfonyl)-1H-indole-2-carbaldehyde (37 mg, 0.1 mmol). From which a 10 g Biotage SNAP KP-Sil column was run on a Biotage Isolera Four purification system (0 – 20% EtOAc in hexanes) to yield titled compound as a yellow solid (14 mg, 46%).

^{1}H NMR (500 MHz, DMSO-d_6) δ 12.30 – 12.19 (m, 1H), 8.42 – 8.39 (m, 2H), 8.17 – 8.13 (m, 2H), 7.77 (d, *J* = 8.6 Hz, 1H), 7.53 (dt, *J* = 1.8, 0.8 Hz, 1H), 7.23 – 7.20 (m, 1H), 7.15 (dd, *J* = 8.6, 1.9 Hz, 1H).

^{13}C{^{1}H} NMR (126 MHz, DMSO-d_6) δ 185.4, 149.9, 143.4, 139.1, 135.2, 131.4, 130.7, 126.2, 125.3, 124.2, 121.8, 113.7, 112.6.

HRMS (ESI) Calculated for: $[C_{15}H_{10}O_3N_2Cl]^+$ 301.0374, found 301.0387

5.5. Preparations of arylsulfonyl-pyrrole-2-carbaldehydes

1-((4-nitrophenyl)sulfonyl)-1H-pyrrole-2-carbaldehyde (3a)

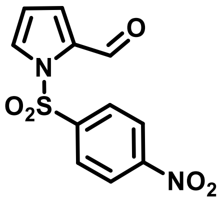

Prepared following general procedure **E** using 1H-pyrrole-2-carbaldehyde (95.0 mg, 1.00 mmol), DMAP (14.0 mg, 0.125 mmol), NEt_3 (174 µL, 1.25 mmol), 4-nitrobenzenesulfonyl chloride (283.0 mg, 1.20 mmol). From which a 10 g Biotage SNAP KP-Sil column was run on a Biotage Isolera Four purification system (0 – 30% EtOAc in hexanes) to yield titled compound as an off-white solid (226 mg, 80%).

^{1}H NMR (400 MHz, $CDCl_3$) δ 9.69 (d, *J* = 0.7 Hz, 1H), 8.37 (dt, *J* = 9.1, 2.1 Hz, 2H), 8.18 (dt, *J* = 9.1, 2.1 Hz, 2H), 7.75 (ddd, *J* = 3.1, 1.8, 0.7 Hz, 1H), 7.19 (t, *J* = 3.8, 1.8 Hz, 1H), 6.50 (t, *J* = 3.6 Hz, 1H).

^{13}C{^{1}H} NMR (101 MHz, $CDCl_3$) δ 177.7, 151.0, 143.6, 133.6, 130.4, 129.6, 128.2, 124.6, 113.0.

HRMS (ESI) Calculated for $[C_{11}H_8N_2NaO_5S]$+ 303.0052, found 303.0058.

IR (neat, cm^{-1}) 3154, 3110, 2981, 2864, 1671, 750, 735.

Data consistent with literature.[23]

1-((2-nitrophenyl)sulfonyl)-1H-pyrrole-2-carbaldehyde (3b)

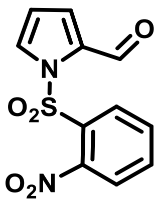

Prepared following general procedure **E** using 1H-pyrrole-2-carbaldehyde (95.0 mg, 1.00 mmol), DMAP (14.0 mg, 0.125 mmol), NEt_3 (174 µL, 1.25 mmol), 2-nitrobenzenesulfonyl chloride (283.0 mg, 1.20 mmol). From which a 10 g Biotage SNAP KP-Sil column was run on a Biotage Isolera Four purification system (0 – 30% EtOAc in hexanes) to yield titled compound as an orange gum (72 mg, 47%).

^{1}H NMR (500 MHz, $CDCl_3$) δ 9.60 (d, *J* = 0.8 Hz, 1H), 8.55 – 8.45 (m, 1H), 7.85 – 7.80 (m, 3H), 7.74 (ddd, *J* = 3.0, 1.8, 0.8 Hz, 1H), 7.22 (dd, *J* = 3.8, 1.8 Hz, 1H), 6.49 (t, *J* = 3.5 Hz, 1H).

^{13}C{^{1}H} NMR (126 MHz, $CDCl_3$) δ 177.4, 135.6, 133.8, 133.5, 132.5, 131.5, 129.2, 125.08, 111.9.

HRMS (ESI) Calculated for: $[C_{11}H_8N_2NaO_5S]^+$ 303.0055, found 303.0046

Data consistent with literature.[24]

1-((5-fluoro-2-nitrophenyl)sulfonyl)-1H-pyrrole-2-carbaldehyde (3c)

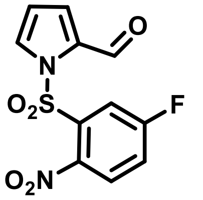

Prepared following general procedure **E** using 1H-pyrrole-2-carbaldehyde (95.0 mg, 1.00 mmol), DMAP (14.0 mg, 0.125 mmol), NEt_3 (174 µL, 1.25 mmol), 2-nitro-5-fluorobenzenesulfonyl chloride (287.0 mg, 1.20 mmol). From which a 10 g Biotage SNAP KP-Sil column was run on a Biotage Isolera Four purification system (0 – 30% EtOAc in hexanes) to yield titled compound as an orange/yellow solid (22mg, 7%).

^{1}H NMR (500 MHz, $CDCl_3$) δ = 9.54 (d, *J* = 1.0, 1H), 8.25 (dd, *J* = 8.2, 2.7, 1H), 7.92 (dd, *J* = 8.8, 4.5, 1H), 7.79 – 7.74 (m, 1H), 7.48 (ddd, *J* = 9.1, 6.6, 2.7, 1H), 7.23 (dd, *J* = 3.8, 1.8, 1H), 6.51 (t, *J* = 3.5, 1H).

^{13}C{^{1}H} NMR (126 MHz, $CDCl_3$) δ 177.2, 163.3 (d, *J* = 260.7 Hz), 144.2, 134.6 (d, *J* = 7.9 Hz), 133.5, 132.8, 130.0, 127.8 (d, *J* = 8.9 Hz), 122.10 – 121.63 (m), 112.1.

^{19}F NMR (471 MHz, $CDCl_3$) δ -100.31 (td, *J* = 7.4, 4.6 Hz).

HRMS (ESI) Calculated for: $[C_{11}H_7FN_2NaO_5S]^+$ 320.9950, found 320.9952.

1-((4-bromo-2-nitrophenyl)sulfonyl)-1H-pyrrole-2-carbaldehyde (3d)

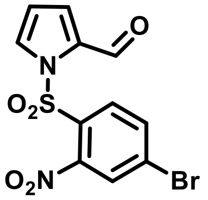

Prepared following general procedure **E** using 1H-pyrrole-2-carbaldehyde (95.0 mg, 1.00 mmol), DMAP (14.0 mg, 0.125 mmol), NEt_3 (174 µL, 1.25 mmol), 2-nitro-4-bromobenzenesulfonyl chloride (287.0 mg, 1.20 mmol). From which a 10 g Biotage SNAP KP-Sil column was run on a Biotage Isolera Four purification system (0 – 30% EtOAc in hexanes) to yield titled compound as a white solid (185 mg, 51%).

^{1}H NMR (500 MHz, $CDCl_3$) δ 9.54 (d, *J* = 0.9 Hz, 1H), 8.45 (d, *J* = 8.5 Hz, 1H), 7.95 (dd, *J* = 8.6, 2.0 Hz, 1H), 7.93 (d, *J* = 1.9 Hz, 1H), 7.73 (ddd, *J* = 3.0, 1.8, 1.0 Hz, 1H), 7.22 (dd, *J* = 3.8, 1.8 Hz, 1H), 6.49 (t, *J* = 3.5 Hz, 1H).

^{13}C{^{1}H} NMR (126 MHz, $CDCl_3$) δ 177.1, 148.2, 135.3, 135.3, 133.3, 132.5, 130.1, 130.0, 130.0, 127.9, 111.8.

HRMS (ESI) Calculated for: $[C_{11}H_7BrN_2NaO_5S]^+$ 380.9150, found 380.9149.

1-((2-fluoro-4-nitrophenyl)sulfonyl)-1H-pyrrole-2-carbaldehyde (3e)

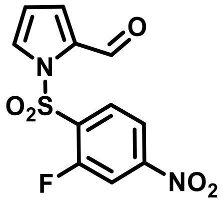

Prepared following general procedure **E** using 1H-pyrrole-2-carbaldehyde (95.0 mg, 1.00 mmol), DMAP (14.0 mg, 0.125 mmol), NEt_3 (174 μL, 1.25 mmol), 2-fluoro-4-nitrobenzenesulfonyl chloride (288.0 mg, 1.20 mmol). From which a 10 g Biotage SNAP KP-Sil column was run on a Biotage Isolera Four purification system (0 – 30% EtOAc in hexanes) to yield titled compound as an off white solid (88 mg, 30%).

^{1}H NMR (500 MHz, $CDCl_3$) δ 9.56 (d, *J* = 1.0 Hz, 1H), 8.52 (dd, *J* = 8.7, 6.9 Hz, 1H), 8.24 (dd, *J* = 8.8, 2.1, 1.0 Hz, 1H), 8.01(dd, *J* = 9.3, 2.1 Hz, 1H), 7.82 (ddq, *J* = 3.3, 1.8, 0.9 Hz, 1H), 7.16 (dd, *J* = 3.8, 1.8 Hz, 1H), 6.44 (t, *J* = 3.5 Hz, 1H).

^{13}C{^{1}H} NMR (126 MHz, $CDCl_3$) δ 177.3, 163.1 (d, *J* = 263.3 Hz), 153.2 (d, *J* =8.2), 133.7, 133.3, 131.6 (d, *J* = 13.3), 131.4 (d, *J* = 2.0), 129.1, 119.2 (d, *J* = 4.3), 113.0 (d, *J* = 26.0), 112.4.

^{19}F NMR (471 MHz, $CDCl_3$) δ -104.11 – -104.21 (m).

HRMS (ESI) Calculated for: $[C_{11}H_8FN_2O_5S]^+$ 299.0132, found 299.0130.

1-((4-nitro-3-(trifluoromethyl)phenyl)sulfonyl)-1H-pyrrole-2-carbaldehyde (3f)

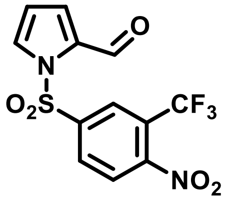

Prepared following general procedure **E** using 1H-pyrrole-2-carbaldehyde (95.0 mg, 1.00 mmol), DMAP (14.0 mg, 0.125 mmol), NEt_3 (174 μL, 1.25 mmol), 4-nitro-3-(trifluoromethyl)benzenesulfonyl chloride (288.0 mg, 1.20 mmol). From which a 10 g Biotage SNAP KP-Sil column was run on a Biotage Isolera Four purification system (0 – 30% EtOAc in hexanes) to yield titled compound as a yellow solid (154 mg, 44%).

^{1}H NMR (500 MHz, $CDCl_3$) δ 9.52 (d, *J* = 0.9 Hz, 1H, 8.38 (m, 2H), 7.93 (d, *J* = 8.3 Hz, 1H), 7.73 (ddd, *J* = 2.9, 1.7, 0.9 Hz, 1H), 7.15 (dd, *J* = 3.8, 1.8 Hz, 1H), 6.47 (t, *J* = 3.5 Hz, 1H).

^{13}C{^{1}H} NMR (126 MHz, $CDCl_3$) δ 177.4, 150.9, 142.1, 133.8, 133.6, 130.8, 129.8, 128.7 (q, *J* = 5.3 Hz), 125.9, 125.0 (q, *J* = 35.7 Hz), 121.1 (d, *J* = 274.6 Hz, *C*F3), 113.2.

^{19}F NMR (471 MHz, $CDCl_3$) δ -60.21 (s).

HRMS (ESI) Calculated for: $[C_{12}H_8F_3N_2O_5S]^+$ 349.0101, found 349.0101.

4-iodo-1-((4-nitrophenyl)sulfonyl)-1H-pyrrole-2-carbaldehyde (3g)

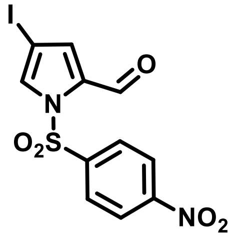

Triethylamine (244 µL, 1.75 mmol) was added to 4-iodo-1H-pyrrole-2-carbaldehyde (310 mg, 1.4 mmol) and DMAP (22 mg, 0.18 mmol) in DCM (0.1M) followed by 4-nitrobenzenesulfonyl chloride (447 mg, 1.7 mmol) and the reaction mixture stirred at room temperature for 16 hours. NH_4Cl (Sat. Sol. Aq.) was added, organics collected and the aqueous extracted with DCM (x 3). Combined organics were washed with brine, dried over $MgSO_4$ and the filtrate dry loaded onto silica before purifying via a biotage isolera system (0 – 20% EtOAc in hexanes) to yield titled compound as a white solid (174 mg, 31%).

^{1}H NMR (400 MHz, $CDCl_3$) δ 9.65 (s, 1H), 8.43 – 8.35 (m, 2H), 8.24 – 8.16 (m, 2H), 7.78 (d, *J* = 1.8 Hz, 1H), 7.22 (d, *J* = 1.8 Hz, 1H).

^{13}C{^{1}H} NMR (101 MHz, $CDCl_3$) δ 176.8, 150.8, 143.0, 134.6, 133.7, 133.6, 129.8, 124.7, 66.3.

HRMS (ESI) Calculated for: $[C_{11}H_7IN_2O_5S]^+$ 406.9193, found 406.9193.

5.6. Preparations of 2-aroylpyrroles

(4-nitrophenyl)(1H-pyrrol-2-yl)methanone (4a)

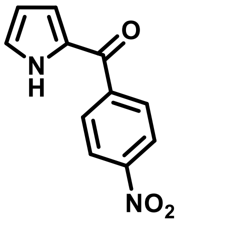

Prepared using general procedure **F** using 1-((4-nitrophenyl)sulfonyl)-1H-pyrrole-2-carbaldehyde (28 mg, 0.10 mmol). From which a 25 g Biotage SNAP KP-Sil column was run on a Biotage Isolera Four purification system (0 – 5% EtOAc in toluene) to yield titled compound as a yellow solid (16 mg, 70%).

^{1}H NMR (400 MHz, $CDCl_3$) δ 9.70 (s, 1H), 8.39 – 8.28 (m, 2H), 8.08 – 7.98 (m, 2H), 7.22 (td, *J* = 2.7, 1.3 Hz, 1H), 6.86 (ddd, *J* = 3.8, 2.4, 1.3 Hz, 1H), 6.39 (dt, *J* = 3.9, 2.5 Hz, 1H).

^{13}C{^{1}H} NMR (101 MHz, $CDCl_3$) δ 182.6, 149.8, 143.7, 130.7, 129.9, 126.6, 123.8, 120.4, 111.9.

HRMS (ESI) Calculated for: $[C_{11}H_9N_2O_3]^+$ 217.0608, found 217.0607.

Data consistent with the literature.[25]

(2-nitrophenyl)(1H-pyrrol-2-yl)methanone (4b)

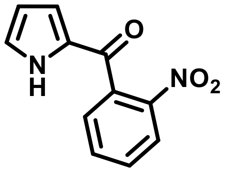

Prepared using general procedure **F** using 1-((2-nitrophenyl)sulfonyl)-1H-pyrrole-2-carbaldehyde (28 mg, 0.10 mmol). From which a 25 g Biotage SNAP KP-Sil column was run on a Biotage Isolera Four purification system (0 – 5% EtOAc in toluene) to yield titled compound as a yellow solid (17 mg, 75%).

^{1}H NMR (500 MHz, $CDCl_3$) δ 9.73 (s, 1H), 8.14 (dd, *J* = 8.1, 1.2 Hz, 1H), 7.73 (td, *J* = 7.5, 1.2 Hz, 1H), 7.72 – 7.62 (m, 1H), 7.60 (dd, *J* = 7.5, 1.5 Hz, 1H), 7.18 (td, *J* = 2.7, 1.3 Hz, 1H), 6.48 (ddd, *J* = 3.9, 2.4, 1.3 Hz, 1H), 6.27 (dt, *J* = 3.9, 2.5 Hz, 1H).

^{13}C{^{1}H} NMR (126 MHz, $CDCl_3$) δ 182.1, 147.6, 135.3, 133.6, 131.2, 130.7, 129.5, 126.7, 124.7, 119.6, 111.6.

HRMS (ESI) Calculated for: $[C_{11}H_8N_2NaO_3]^+$ 239.0421, found 239.0421.

Data consistent with the literature.[26]

(5-fluoro-2-nitrophenyl)(1H-pyrrol-2-yl)methanone (4c)

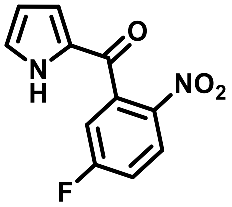

Prepared using general procedure **F** using 1-((5-fluoro-2-nitrophenyl)sulfonyl)-1H-pyrrole-2-carbaldehyde (29.8 mg, 0.10 mmol). From which a 25 g Biotage SNAP KP-Sil column was run on a Biotage Isolera Four purification system (0 – 5% EtOAc in toluene) to yield titled compound as an off white solid (19 mg, 80%).

^{1}H NMR (500 MHz, $CDCl_3$) δ 9.95 (s, 1H), 8.21 (dd, *J* = 9.0, 4.7 Hz, 1H), 7.34 – 7.26 (m, 2H), 7.21 (td, *J* = 2.7, 1.3 Hz, 1H), 6.50 (ddd, *J* = 3.9, 2.4, 1.3 Hz, 1H), 6.29 (dt, *J* = 4.0, 2.5 Hz, 1H).

^{13}C{^{1}H} NMR (126 MHz, $CDCl_3$) δ 180.4, 164.9 (d, *J* = 259.8), 143.4, 138.2 (d, *J* = 8.0), 130.6, 127.6 (d, *J* = 9.6), 127.4, 120.0, 117.5 (d, *J* = 23.4), 116.9 (d, *J* = 24.9), 111.8.

^{19}F NMR (471 MHz, $CDCl_3$) δ -101.82 (td, *J* = 7.5, 4.6 Hz).

HRMS (ESI) Calculated for: $[C_{11}H_7FN_2NaO_3]^+$ 257.0333, found 257.0337

(4-bromo-2-nitrophenyl)(1H-pyrrol-2-yl)methanone (4d)

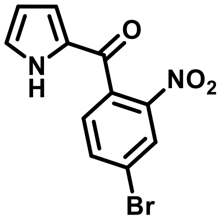

Prepared using general procedure **F** using 1-((4-bromo-2-nitrophenyl)sulfonyl)-1H-pyrrole-2-carbaldehyde (36 mg, 0.10 mmol). From which a 25 g Biotage SNAP KP-Sil column was run on a Biotage Isolera Four purification system (0 – 5% EtOAc in toluene) to yield titled compound as an off white solid (14 mg, 47%).

^{1}H NMR (400 MHz, $CDCl_3$) δ 9.59 (s, 1H), 8.28 (d, *J* = 1.9 Hz, 1H), 7.86 (dd, *J* = 8.1, 1.9 Hz, 1H), 7.47 (d, *J* = 8.1 Hz, 1H), 7.19 (td, *J* = 2.7, 1.3 Hz, 1H), 6.48 (ddd, *J* = 3.9, 2.5, 1.3 Hz, 1H), 6.29 (dt, *J* = 4.0, 2.5 Hz, 1H).

^{13}C{^{1}H} NMR (101 MHz, $CDCl_3$) δ 180.8, 136.5, 134.0, 130.9, 130.8, 127.8, 126.9, 124.3, 119.6, 111.8.

HRMS (ESI) Calculated for: $[C_{11}H_7BrN_2NaO_3]^+$ 316.9532, found 316.9531.

(2-fluoro-4-nitrophenyl)(1H-pyrrol-2-yl)methanone (4e)

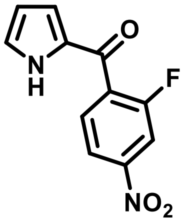

Prepared using general procedure **F** using 1-((2-fluoro-4-nitrophenyl)sulfonyl)-1H-pyrrole-2-carbaldehyde (30 mg, 0.10 mmol). From which a 25 g Biotage SNAP KP-Sil column was run on a Biotage Isolera Four purification system (0 – 5% EtOAc in toluene) to yield titled compound as a yellow solid (13 mg, 57%).

^{1}H NMR (400 MHz, $CDCl_3$) δ 9.75 (s, 1H), 8.14 (ddd, *J* = 8.4, 2.1, 0.8 Hz, 1H), 8.06 (dd, *J* = 9.0, 2.1 Hz, 1H), 7.77 (dd, *J* = 8.4, 6.6 Hz, 1H), 7.23 (td, *J* = 2.8, 1.3 Hz, 1H), 6.72 (tt, *J* = 3.9, 2.8, 1.5 Hz, 1H), 6.36 (dt, *J* = 4.0, 2.4 Hz, 1H).

^{13}C{^{1}H} NMR (101 MHz, $CDCl_3$) δ 178.9, 159.5 (d, *J* = 257.0 Hz), 158.2 133.0 (d, *J* = 16.2 Hz), 131.3, 131.2 (d, *J* = 3.6 Hz), 127.4, 121.0 (d, *J* = 2.8 Hz), 119.3 (d, *J* = 4.0 Hz), 112.45, 112.1.

^{19}F NMR (471 MHz, $CDCl_3$) δ -108.93 (t, *J* = 7.9 Hz).

HRMS (ESI) Calculated for: $[C_{11}H_6FN_2O_3]^+$ 233.0368, found 233.0357.

(4-nitro-3-(trifluoromethyl)phenyl)(1H-pyrrol-2-yl)methanone (4f)

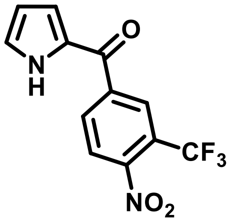

Prepared using general procedure **F** using 1-((4-nitro-3-(trifluoromethyl)phenyl)sulfonyl)-1H-pyrrole-2-carbaldehyde (30 mg, 0.10 mmol). From which a 25 g Biotage SNAP KP-Sil column was run on a Biotage Isolera Four purification system (0 – 5% EtOAc in toluene) to yield titled compound as a yellow solid (8 mg, 28%).

^{1}H NMR (400 MHz, $CDCl_3$) δ 9.61 (s, 1H), 8.32 (d, *J* = 1.8 Hz, 1H), 8.21 (dd, *J* = 8.3, 1.8 Hz, 1H), 7.99 (d, *J* = 8.2 Hz, 1H), 7.25 (dd, *J* = 2.8, 1.3 Hz, 1H), 6.85 (ddd, *J* = 3.9, 2.5, 1.3 Hz, 1H), 6.42 (dt, *J* = 3.9, 2.5 Hz, 1H).

^{13}C{^{1}H} NMR (101 MHz, $CDCl_3$) δ 180.7, 142.0, 133.3, 130.2, 128.6 (d, *J* = 5.3 Hz), 127.1, 125.3, 120.4, 112.3.

^{19}F NMR (471 MHz, $CDCl_3$) δ -60.04.

HRMS (ESI) Calculated for: $[C_{12}H_6F_3N_2O_3]^-$ 283.0336, found 283.0323.

(4-iodo-1H-pyrrol-2-yl)(4-nitrophenyl)methanone (4g)

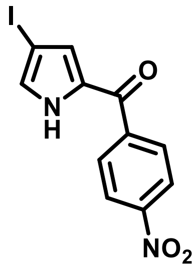

Prepared using general procedure **F** using 4-iodo-1-((4-nitrophenyl)sulfonyl)-1H-pyrrole-2-carbaldehyde (41 mg, 0.10 mmol). From which a 10 g Biotage SNAP KP-Sil column was run on a Biotage Isolera Four purification system (0 – 20% EtOAc in hexanes) to yield titled compound as a yellow solid (10 mg, 29%).

^{1}H NMR (500 MHz, $(CD_3)_2CO$) δ 11.43 (s, 1H), 8.42 – 8.38 (m, 2H), 8.15 – 8.08 (m, 2H), 7.45 (d, *J* = 1.4 Hz, 1H), 7.04 (d, *J* = 1.4 Hz, 1H).

^{13}C{^{1}H} NMR (126 MHz, $(CD_3)_2CO$) δ 182.1, 150.7, 144.3, 133.1, 132.4, 130.8, 126.6, 124.5, 62.7 (CI_{qPy}).

HRMS (ESI) Calculated for: $[C_{11}H_7IN_2O_3]^+$ 341.9496, found 341.9492.

5.7. Derivatisation reactions

(3-bromo-1*H*-indol-2-yl)(4-nitrophenyl)methanone (5)

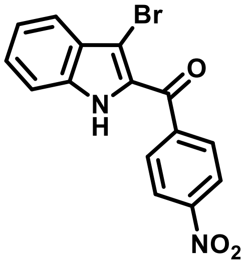

N-Bromosuccinimide (21 mg, 0.12 mmol) was added to (1H-indol-2-yl)(4-nitrophenyl)methanone (27 mg, 0.1 mmol) in DMF (0.1 M) and the reaction mixture stirred at room temperature for 3 hours. Brine (2 mL) added and aqueous extracted with EtOAc (3 x 5 mL). Combined organics were dry loaded onto silica, from which a 10 g Biotage SNAP KP-Sil column was run on a Biotage Isolera Four purification system (0 – 20% EtOAc in hexanes) to yield titled compound as an orange solid (22 mg, 64%).

^{1}H NMR (400 MHz, $CDCl_3$) δ 9.39 (s, 1H), 8.41 – 8.32 (m, 2H), 8.01 – 7.94 (m, 2H), 7.68 (dq, *J* = 8.3, 1.0 Hz, 1H), 7.49 – 7.43 (m, 2H), 7.31 – 7.27 (m, 1H).

^{13}C{^{1}H} NMR (101 MHz, $CDCl_3$) δ 186.5, 150.1, 143.1, 136.5, 130.9, 130.4, 128.4, 123.7, 122.4, 122.2, 112.5, 100.8.

HRMS (ESI) Calculated for: $[C_{15}H_8BrN_2O_3]^-$ 342.9797, found 342.9797.

IR (neat, cm^{-1}) 3347, 2921, 2852, 1595, 1510, 1455, 1419, 1338, 1290, 719.

(1*H*-indol-2-yl)(4-nitrophenyl)methanol (6)

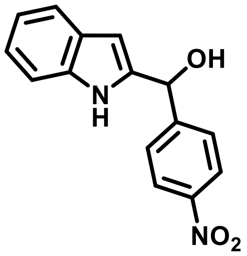

$NaBH_4$ (11 mg, 0.3 mmol) was added portionwise to (1H-indol-2-yl)(4-nitrophenyl)methanone (27 mg, 0.1 mmol) in MeOH (0.1 M) at 0 °C under a N_2 atmosphere. Reaction was stirred whilst warming to room temperature for 16 hours before quenching with $NaHCO_3$ (Sat. Sol Aq.). Aqueous was extracted with DCM (3 x 10 mL) and combined organics washed with brine, dried over $MgSO_4$ and filtered before dry loading onto silica. From which a 10 g Biotage SNAP KP-Sil column was run on a Biotage Isolera Four purification system (0 – 40% EtOAc in hexanes) to yield titled compound as an orange solid (23 mg, 85%).

^{1}H NMR (400 MHz, $CDCl_3$) δ 8.29 – 8.18 (m, 3H + CH_{Ar}), 7.68 – 7.60 (m, 2H), 7.57 (dd, *J* = 7.8, 1.2 Hz, 1H), 7.33 (dd, *J* = 8.2, 1.0 Hz, 1H), 7.20 (ddd, *J* = 8.2, 7.0, 1.3 Hz, 1H), 7.11 (ddd, *J* = 8.1, 7.1, 1.1 Hz, 1H), 6.35 (d, *J* = 1.9 Hz, 1H), 6.13 (s, 1H), 2.72 (s, 1H).

^{13}C{^{1}H} NMR (101 MHz, $CDCl_3$) δ 148.7, 147.7, 138.6, 136.5, 127.9, 127.4, 123.9, 122.9, 121.0, 120.4, 111.3, 101.8, 70.0.

HRMS (ESI) Calculated for: $[C_{15}H_{11}N_2O_3]^-$ 267.0848, found 267.0766.

(1-methyl-1*H*-indol-2-yl)(4-nitrophenyl)methanone (7a)

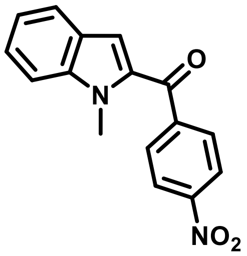

MeI (12.5 µL, 0.2 mmol) was added to (1H-indol-2-yl)(4-nitrophenyl)methanone (27 mg, 0.1 mmol) and K_2CO_3 (35 mg, 0.25 mmol) in DMF (0.1 M) and the reaction stirred at room temperature for 16 hours. Brine (2 mL) added and aqueous extracted with EtOAc (3 x 5 mL), combined organics were washed with LiCl (Sat. Sol. Aq.) before drying over $MgSO_4$. Organics were dry loaded onto silica from which a 10 g Biotage SNAP KP-Sil column was run on a Biotage Isolera Four purification system (0 – 20% EtOAc in hexanes) to yield titled compound as a yellow solid (24 mg, 86%).

^{1}H NMR (500 MHz, $CDCl_3$) δ 8.38 – 8.32 (m, 2H), 8.07 – 8.00 (m, 2H), 7.68 (dt, *J* = 8.0, 1.0 Hz, 1H), 7.49 – 7.42 (m, 2H), 7.20 (ddd, *J* = 8.0, 4.7, 3.1 Hz, 1H), 6.99 (s, 1H), 4.16 (s, 3H).

^{13}C{^{1}H} NMR (126 MHz, $CDCl_3$) δ 186.6, 149.8, 144.8, 140.9, 134.1, 130.5, 127.0, 125.9, 123.6, 123.4, 121.4, 116.2, 110.6, 32.3.

HRMS (ESI) Calculated for: $[C_{16}H_{13}N_2O_3]^+$ 281.0912, found 281.0912.

Data consistent with the literature.[27]

(1-methyl-1*H*-pyrrol-2-yl)(4-nitrophenyl)methanone (7b)

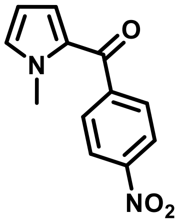

MeI (12.5 µL, 0.2 mmol) was added to (4-nitrophenyl)(1H-pyrrol-2-yl)methanone (22 mg, 0.1 mmol) and K_2CO_3 (35 mg, 0.25 mmol) in DMF (0.1 M) and the reaction stirred at room temperature for 16 hours. Brine (2 mL) added and aqueous extracted with EtOAc (3 x 5 mL), combined organics were washed with LiCl (Sat. Sol. Aq.) before drying over $MgSO_4$. Organics were dry loaded onto silica from which a 10 g Biotage SNAP KP-Sil column was run on a Biotage Isolera Four purification system (0 – 20% EtOAc in hexanes) to yield titled compound as a yellow solid (20 mg, 87%).

^{1}H NMR (400 MHz, $CDCl_3$) δ 8.34 – 8.26 (m, 2H), 7.95 – 7.87 (m, 2H), 6.99 (t, *J* = 2.1 Hz, 1H), 6.68 (dd, *J* = 4.2, 1.7 Hz, 1H), 6.19 (dd, *J* = 4.2, 2.5 Hz, 1H), 4.05 (s, 3H).

^{13}C{^{1}H} NMR (101 MHz, $CDCl_3$) δ 183.8, 149.4, 145.5, 132.9, 129.9, 129.9, 123.8, 123.5, 109.0, 37.7.

HRMS (ESI) Calculated for: $[C_{12}H_{11}N_2O_3]^+$ 231.0764, found 231.0762.

Data consistent with the literature.[28]

(4-aminophenyl)(1*H*-indol-2-yl)methanone (8a)

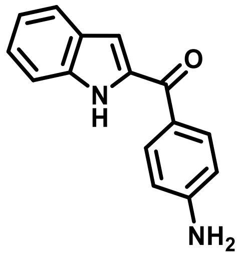

A drop of glacial acetic acid was added to (1H-indol-2-yl)(4-nitrophenyl)methanone (27 mg, 0.1 mmol), Zn (27 mg, 0.42 mmol) in EtOH:H_2O (3 mL,1:2 EtOH:H_2O) and the reaction heated to 80 °C for 2 hours in an oil bath. Volatiles were removed *in vacuo,* water added and aqueous extracted with DCM (x3), before washing combined organics with brine and drying over $MgSO_4$. Organics were filtered, dry loaded onto silica from which a 10 g Biotage SNAP KP-Sil column was run on a Biotage Isolera Four purification system (0-40 % EtOAc in hexanes) to give titled compound as a yellow gum (22 mg, 93%).

^{1}H NMR (500 MHz, $CDCl_3$) δ 9.57 (s, 1H), 8.03 – 7.92 (m, 2H), 7.72 (dd, *J* = 8.1, 1.1 Hz, 1H), 7.47 (dd, *J* = 8.4, 1.0 Hz, 1H), 7.35 (ddd, *J* = 8.3, 7.0, 1.1 Hz, 1H), 7.19 – 7.13 (m, 2H), 6.79 – 6.68 (m, 2H), 4.17 (s, 2H).

^{13}C{^{1}H} NMR (126 MHz, $CDCl_3$) δ 185.6, 151.0, 137.2, 134.9, 132.0, 128.1, 127.9, 125.9, 122.0, 120.9, 114.0, 112.2, 111.2.

HRMS (ESI) Calculated for: $[C_{15}H_{13}N_2O]^+$ 237.1022, found 237.1020.

(4-aminophenyl)(1H-pyrrol-2-yl)methanone (8b)

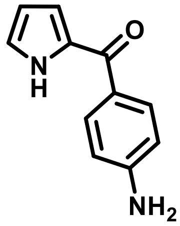

A drop of glacial acetic acid was added to (4-nitrophenyl)(1H-pyrrol-2-yl)methanone (22 mg, 0.1 mmol), Zn (27 mg, 0.42 mmol) in EtOH:H_2O (3 mL,1:2 EtOH:H_2O) and the reaction heated to 80 °C for 2 hours in an oil bath. Volatiles were removed *in vacuo,* water added and aqueous extracted with DCM (x3), before washing combined organics with brine and drying over $MgSO_4$. Organics were filtered, dry loaded onto silica from which a 10 g Biotage SNAP KP-Sil column was run on a Biotage Isolera Four purification system (0-40 % EtOAc in hexanes) to give titled compound as an off white solid (13 mg, 70%).

^{1}H NMR (400 MHz, $CDCl_3$) δ 9.62 (s, 1H), 7.89 – 7.80 (m, 2H), 7.08 (td, *J* = 2.7, 1.3 Hz, 1H), 6.89 (ddd, *J* = 3.8, 2.4, 1.3 Hz, 1H), 6.75 – 6.67 (m, 2H), 6.32 (dt, *J* = 3.9, 2.6 Hz, 1H), 4.07 (s, 2H).

^{13}C{^{1}H} NMR (101 MHz, $CDCl_3$) δ 183.5, 150.5, 131.6, 131.4, 128.4, 124.1, 117.9, 114.0, 110.8.

HRMS (ESI) Calculated for: $[C_{11}H_{11}N_2O]^+$ 187.0866, found 187.0864.

6-nitro-9H-pyrrolo[1,2-a]indol-9-one (9)

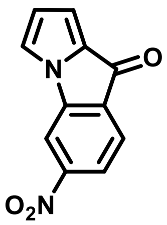

Cs_2CO_3 (66 mg, 0.2 mmol) was added to (2-fluoro-4-nitrophenyl)(1H-pyrrol-2-yl)methanone (23 mg, 0.1 mmol) in DMF (0.1 M) and the reaction heated to 70 °C in an oil bath for 16 hours. Reaction was allowed to cool to rt before addition of brine (2 mL) and EtOAc (3 mL) and the organics were collected. Aqueous was extracted with EtOAc (x 3) and combined organics was with brine, dried over $MgSO_4$ and filtered before dry loading onto silica. From which a 10 g Biotage SNAP KP-Sil column was run on a Biotage Isolera Four purification system (0-30 % EtOAc in hexanes) to give titled compound as a yellow solid (13 mg, 56%).

^{1}H NMR (400 MHz, $CDCl_3$) δ 8.06 (dd, *J* = 8.1, 1.9 Hz, 1H), 7.96 (d, *J* = 1.9 Hz, 1H), 7.74 (d, *J* = 8.1 Hz, 1H), 7.21 (dd, *J* = 2.6, 0.9 Hz, 1H), 6.91 (dd, *J* = 3.8, 0.8 Hz, 1H), 6.44 (dd, *J* = 3.8, 2.6 Hz, 1H).

^{13}C{^{1}H} NMR (101 MHz, $CDCl_3$) δ 177.0, 151.8, 144.1, 135.2, 132.9, 125.1, 121.4, 120.7, 117.9, 116.1, 105.9.

HRMS (ESI) Calculated for: $[C_{11}H_6O_3N_2]^+$ 214.0384, found 214.0377.

6. Crossover Experiment

Procedure – Crossover Experiment.

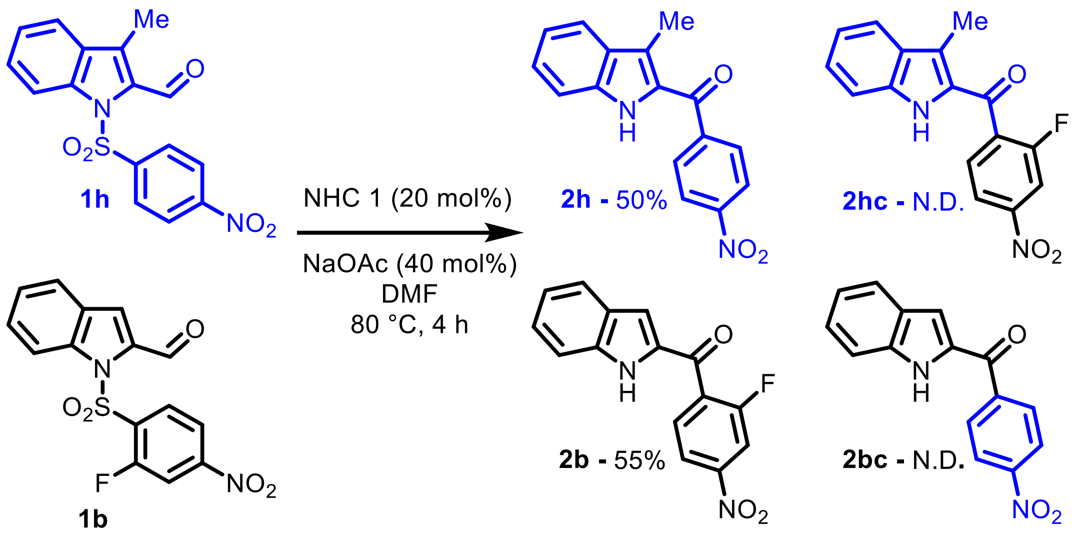

An oven-dried microwave vial (2 – 5 mL) containing **1h** (0.1 mmol, 1 eq.), **1b** (0.1 mmol, 1 eq.)C 1 (11.0 mg, 0.04 mmol, 40 mol%) and NaOAc (6.6 mg, 0.08 mmol, 80 mol%) was evacuated and backfilled 3 times with nitrogen. DMF (2 mL) added and the reaction mixture degassed by sparging N_2 for 15 minutes at room temperature. The sealed vessel was heated for 4 hours at 80 °C in an oil bath, allowed to cool to room temperature, brine (2 mL) added and aqueous extracted with EtOAc (3 mL x 3). Combined organics were dry loaded onto silica before purifying via a biotage isolera system, where all fractions were collected and combined (0 – 40% EtOAc in hexanes). ^{1}H NMR yield was then obtained, using nitromethane as an internal standard.

Data – Crossover Experiment.

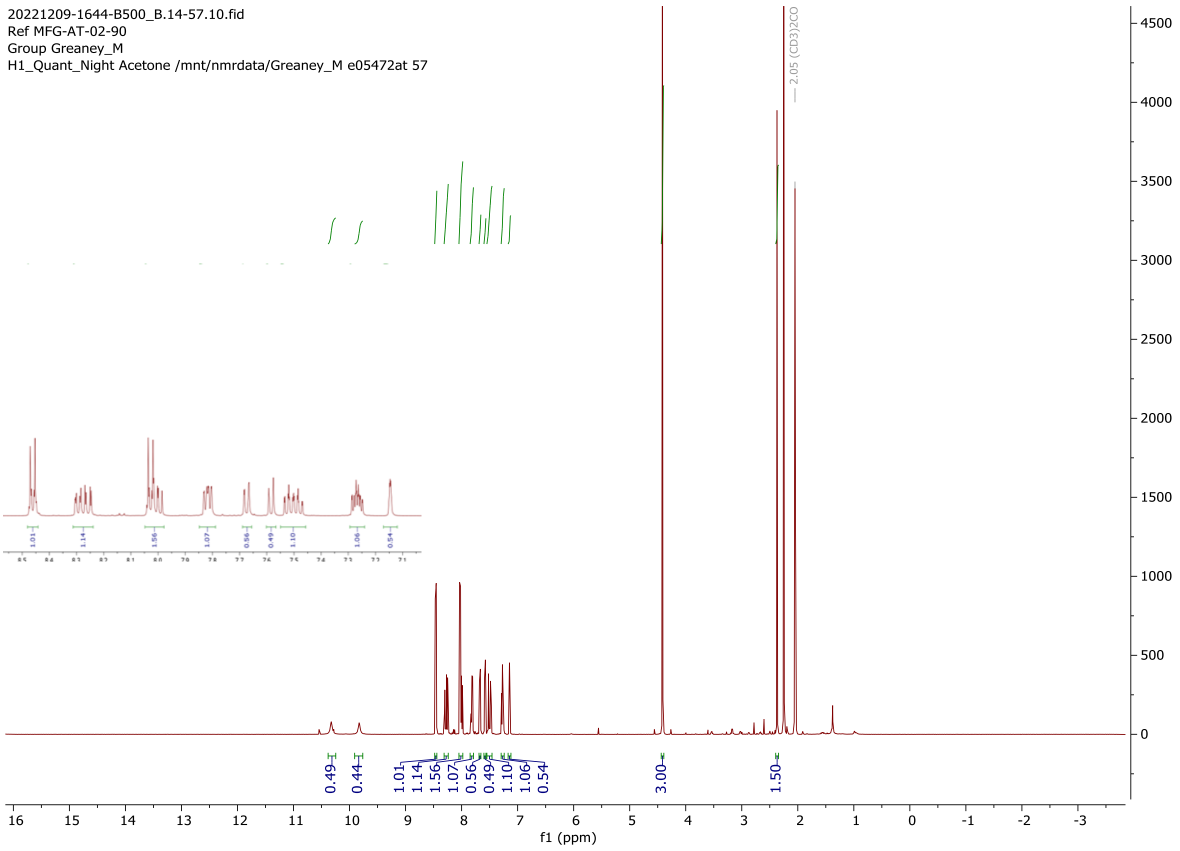

```
File        :D:\msdchem\1\Raw_Data\69792.D
Operator    :
Acquired    : 15 Dec 2022   14:36      using AcqMethod 50T300@25C_MIN_20_SPLIT_HOLD_20MIN_EI.M
Instrument :     GC-MSD
Sample Name: AT-02-90
Misc Info   :
Vial Number: 92
```

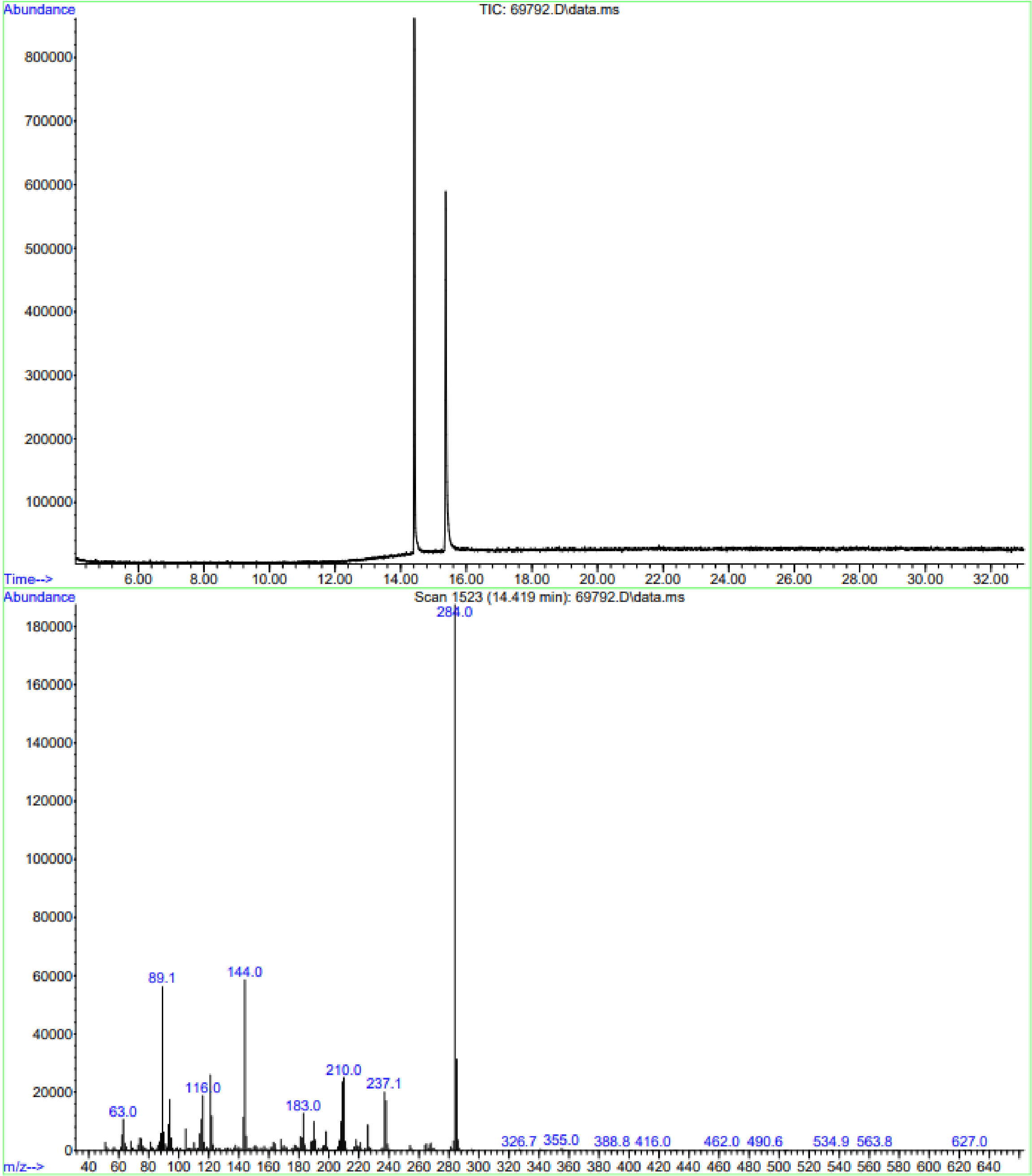

```
File        :D:\msdchem\1\Raw_Data\69792.D
Operator    :
Acquired    : 15 Dec 2022  14:36     using AcqMethod 50T300@25C_MIN_20_SPLIT_HOLD_20MIN_EI.M
Instrument  :     GC-MSD
Sample Name: AT-02-90
Misc Info   :
Vial Number: 92
```

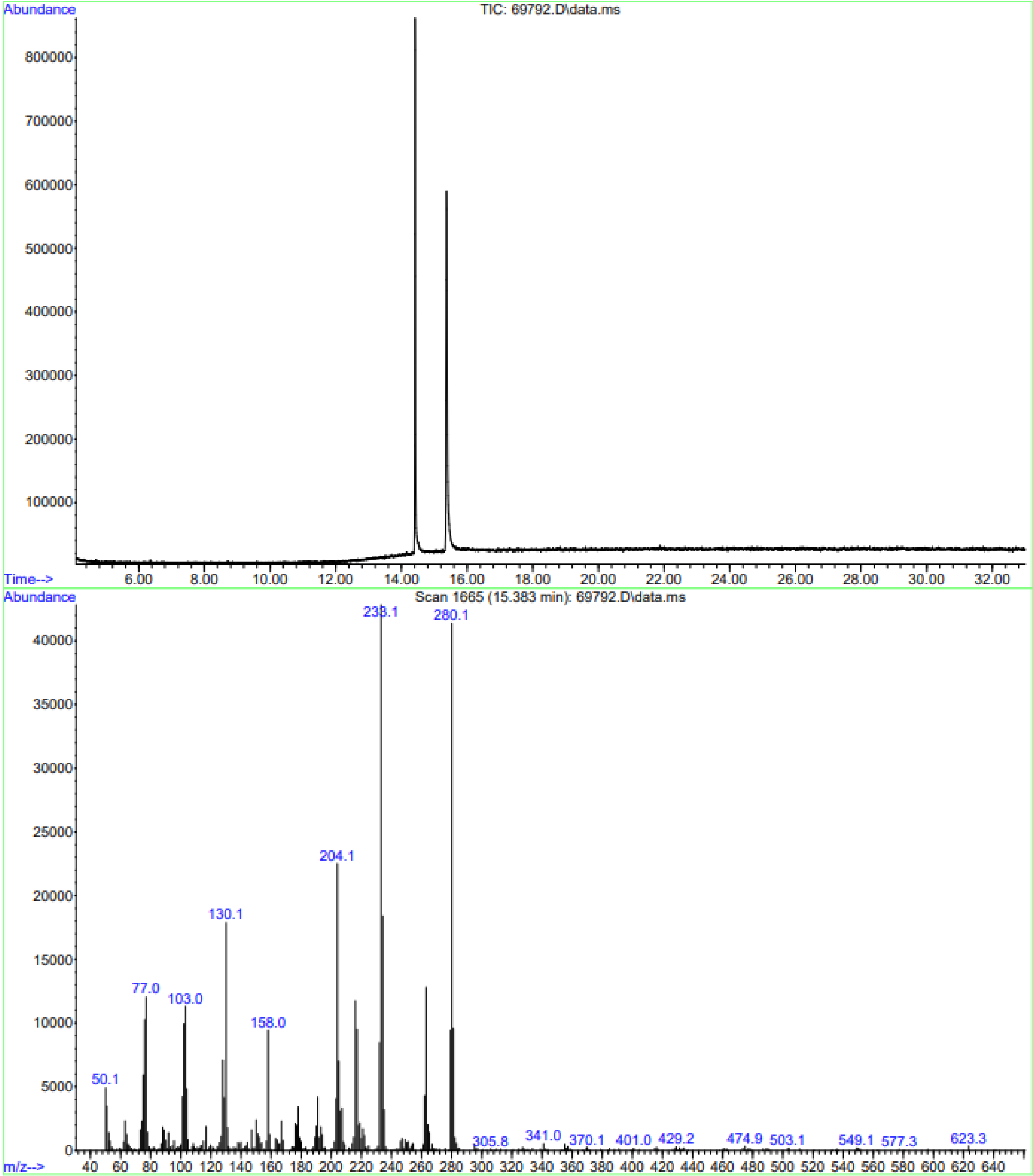

7. NMR Spectra for Synthesised Compounds

(1*H*-indol-2-yl)methanol (1aa)

^{1}H NMR (400 MHz, $CDCl_3$)

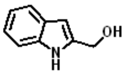

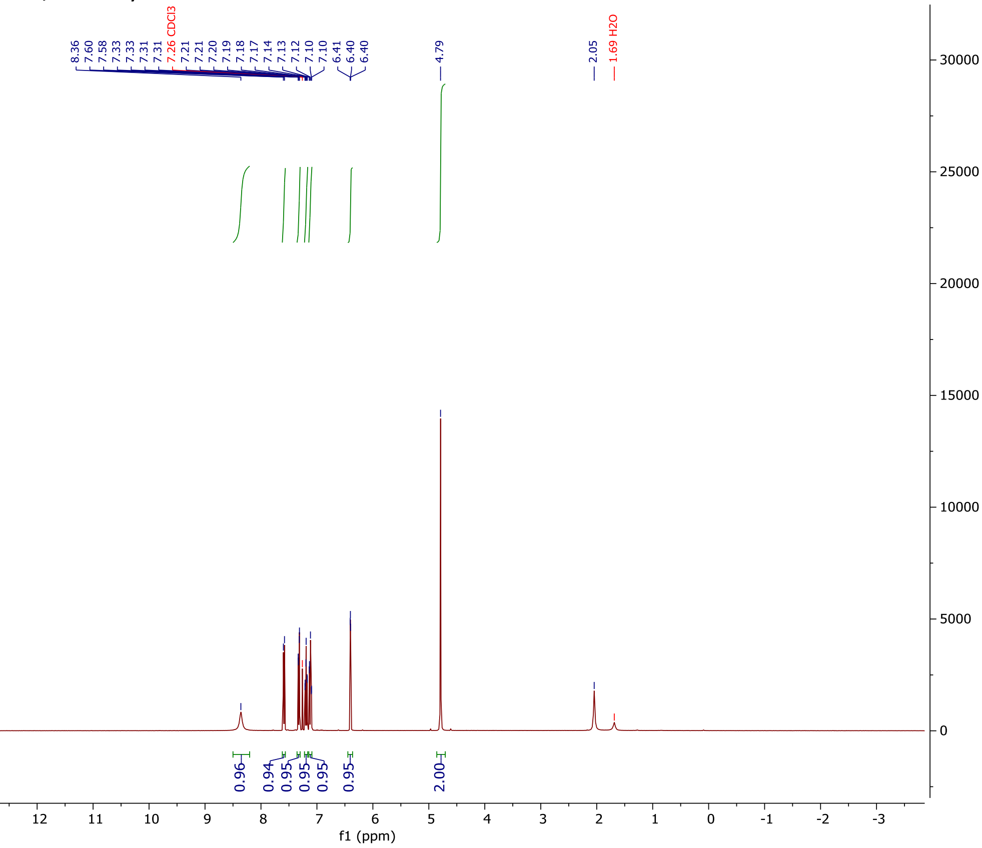

$^{13}C\{^{1}H\}$ NMR (101 MHz, $CDCl_3$)

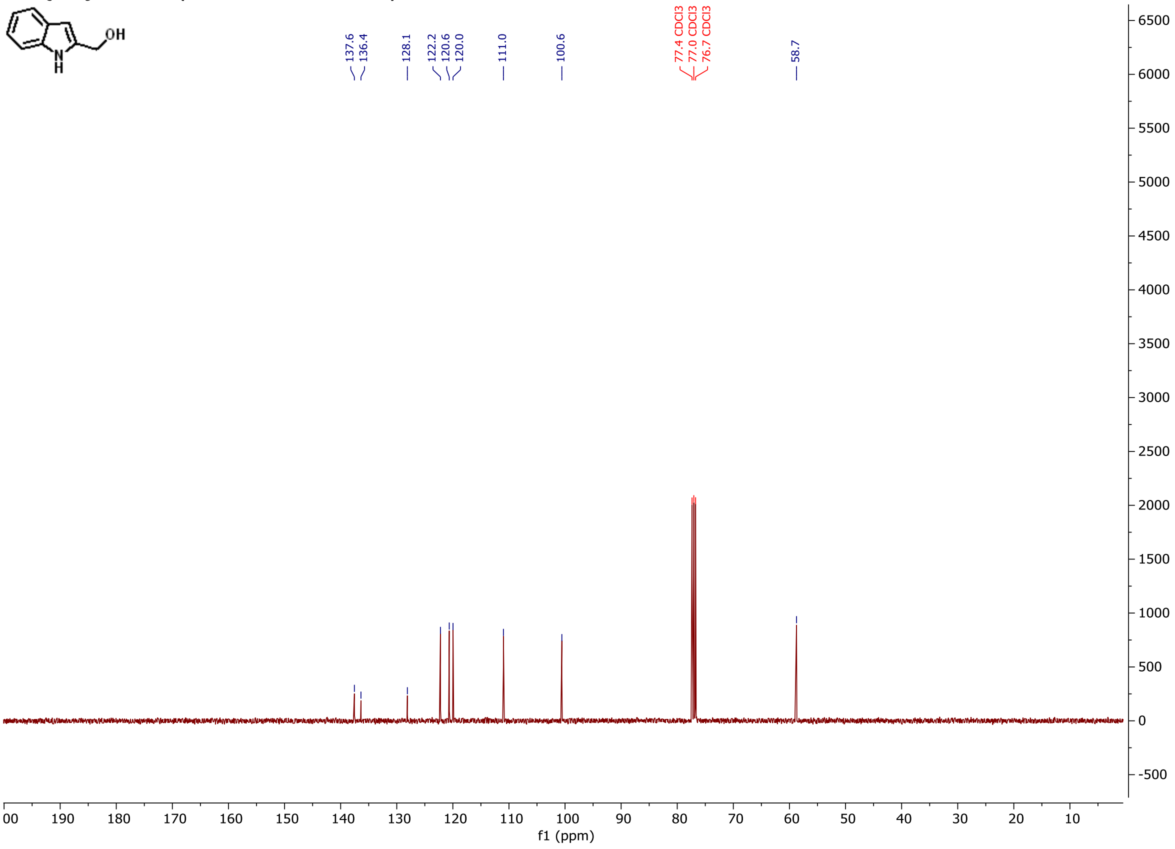

(3-methyl-1H-indol-2-yl)methanol (1ha)

^{1}H NMR (400 MHz, $CDCl_3$)

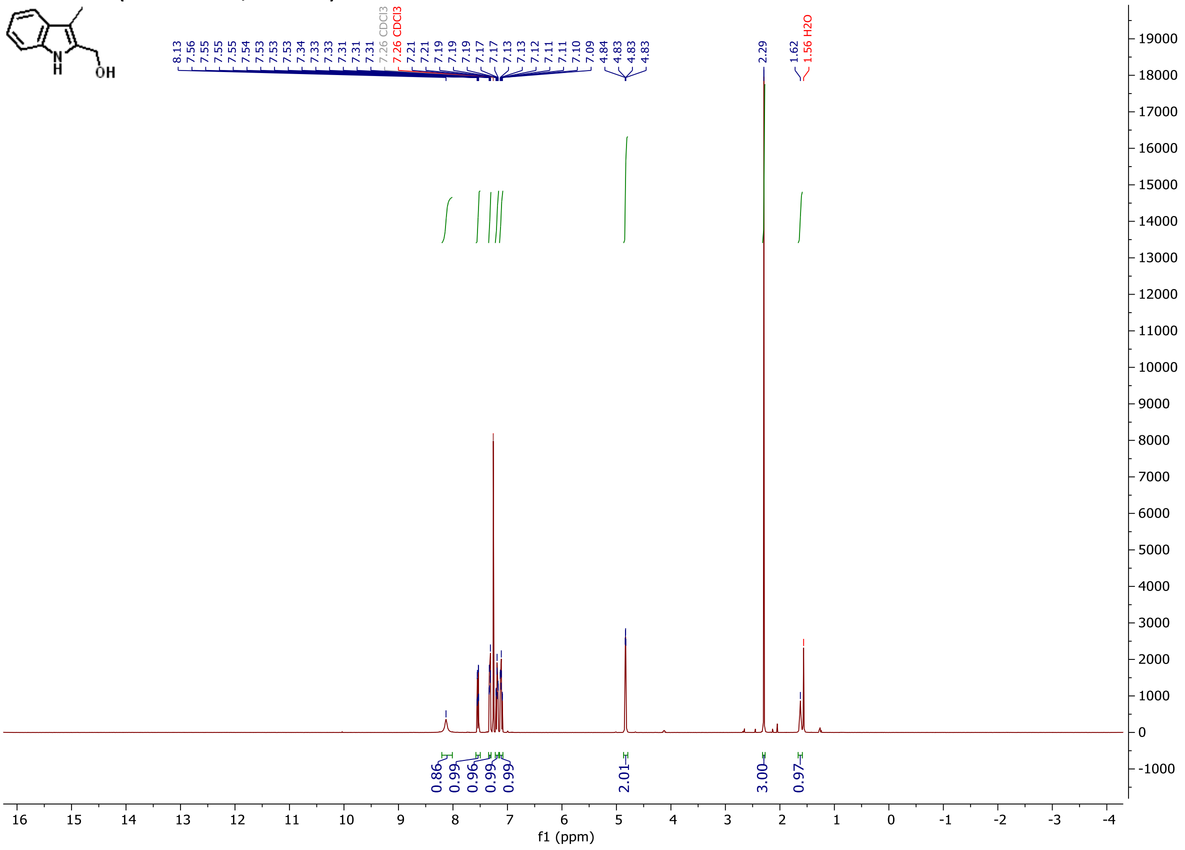

$^{13}C\{^{1}H\}$ NMR (101 MHz, $CDCl_3$)

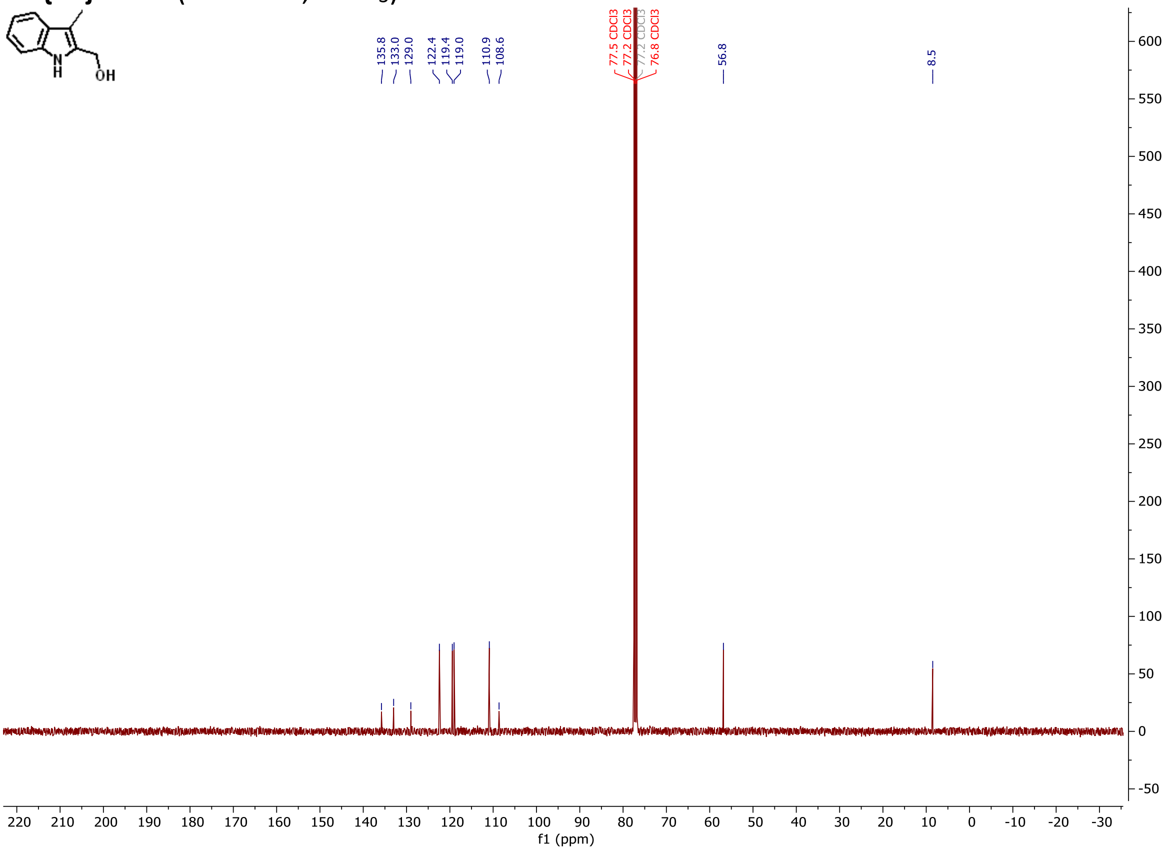

(5-methyl-1H-indol-2-yl)methanol (1ia)

^{1}H NMR (400 MHz, $CDCl_3$)

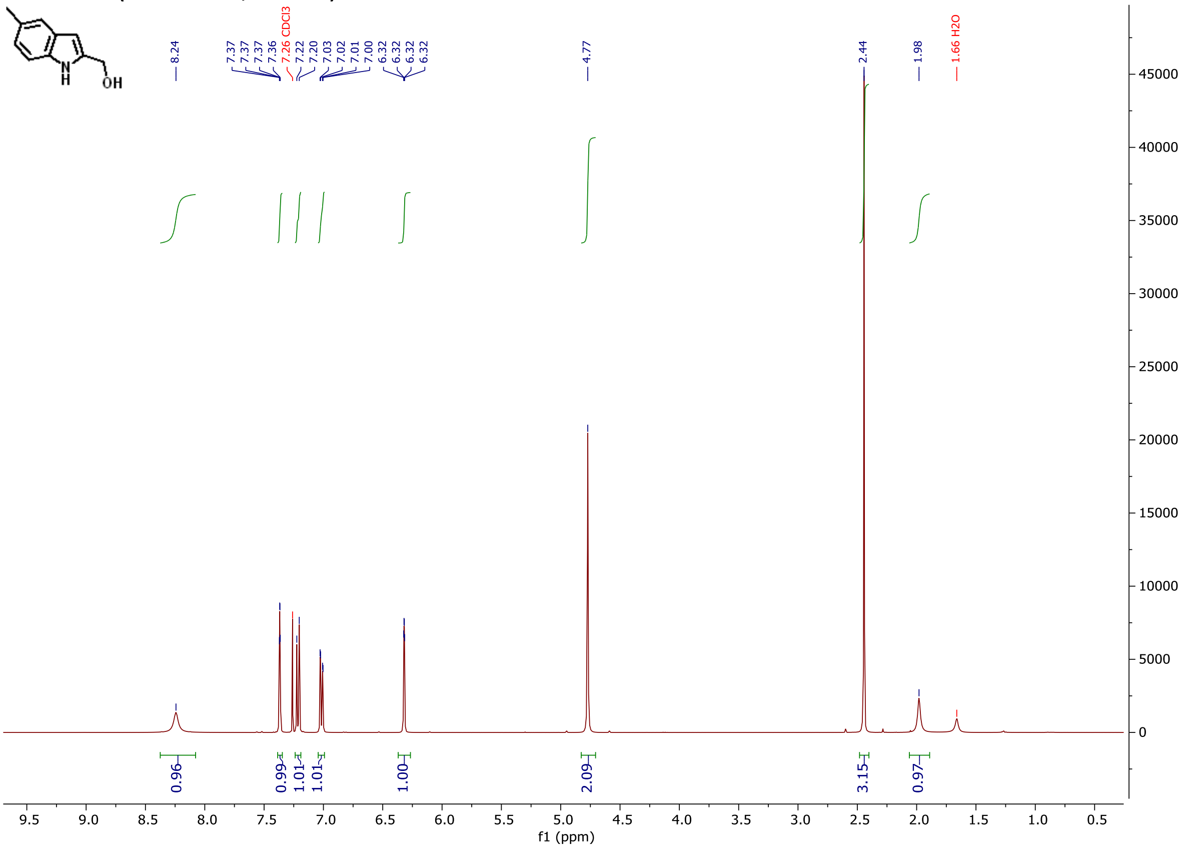

^{13}C{^{1}H} NMR (101 MHz, $CDCl_3$)

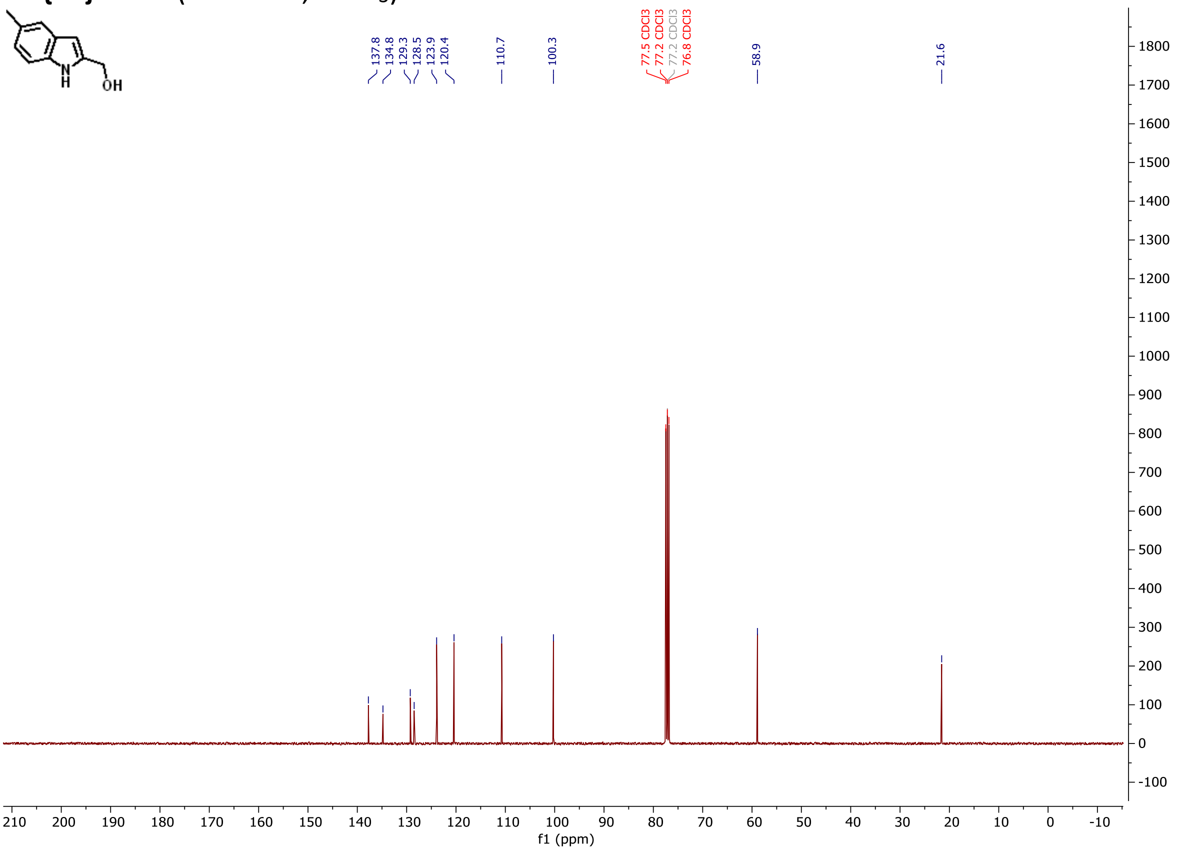

(5-chloro-1H-indol-2-yl)methanol (1ja)

^{1}H NMR (400 MHz, $CDCl_3$)

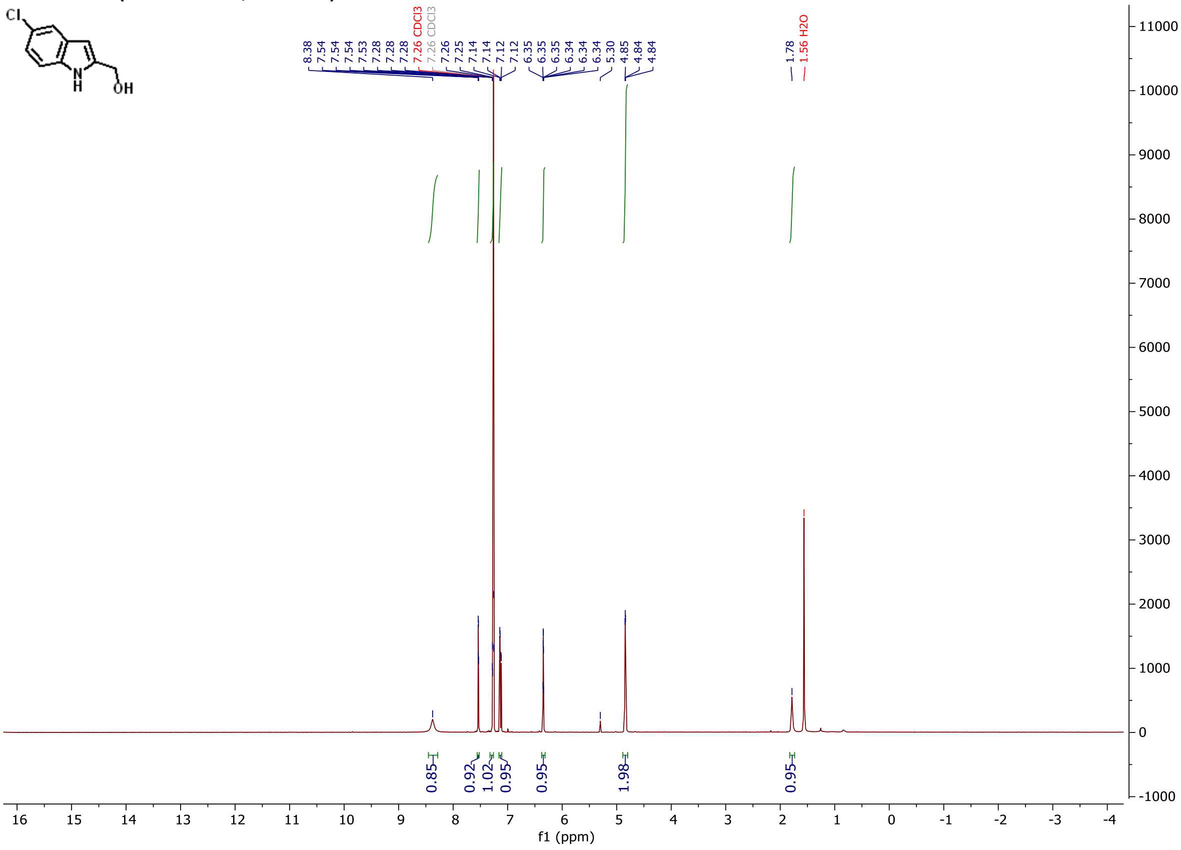

$^{13}C\{^1H\}$ NMR (101 MHz, $CDCl_3$)

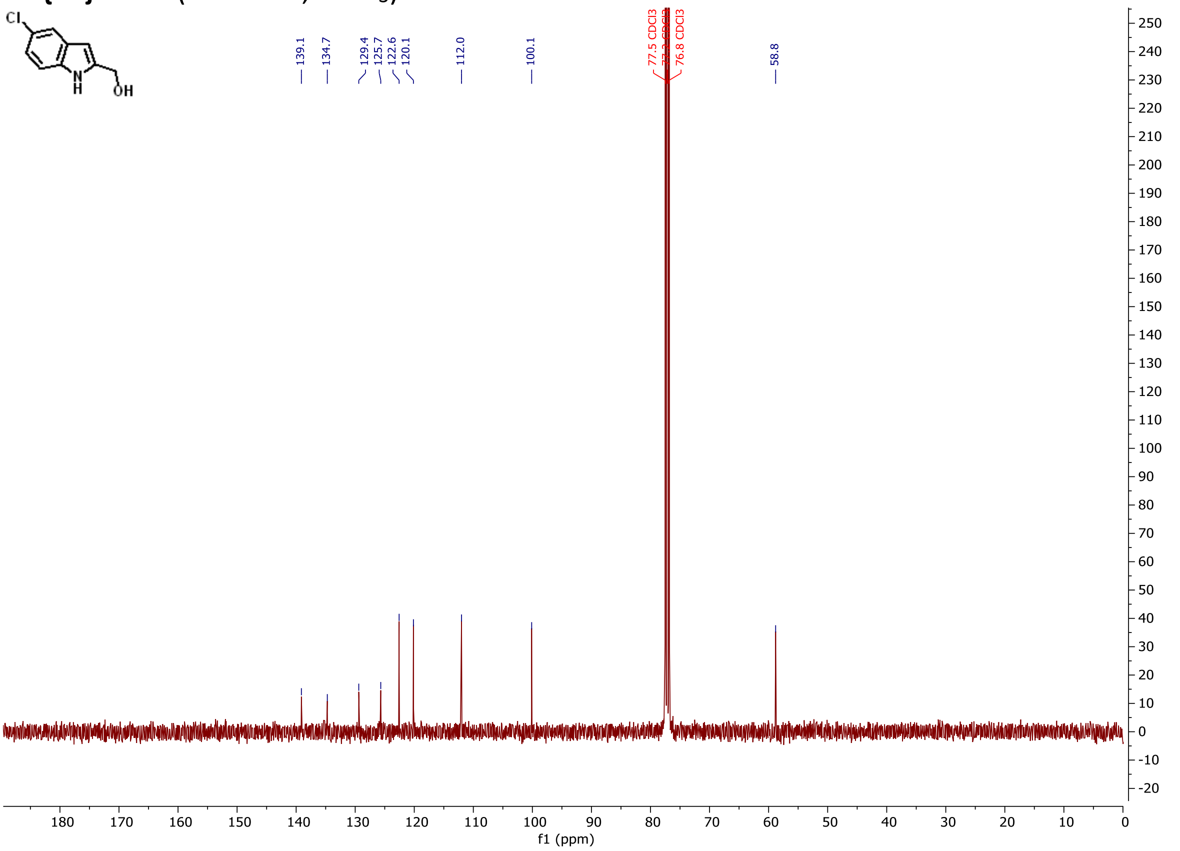

(5-fluoro-1H-indol-2-yl)methanol (1ka)

^{1}H NMR (500 MHz, $CDCl_3$)

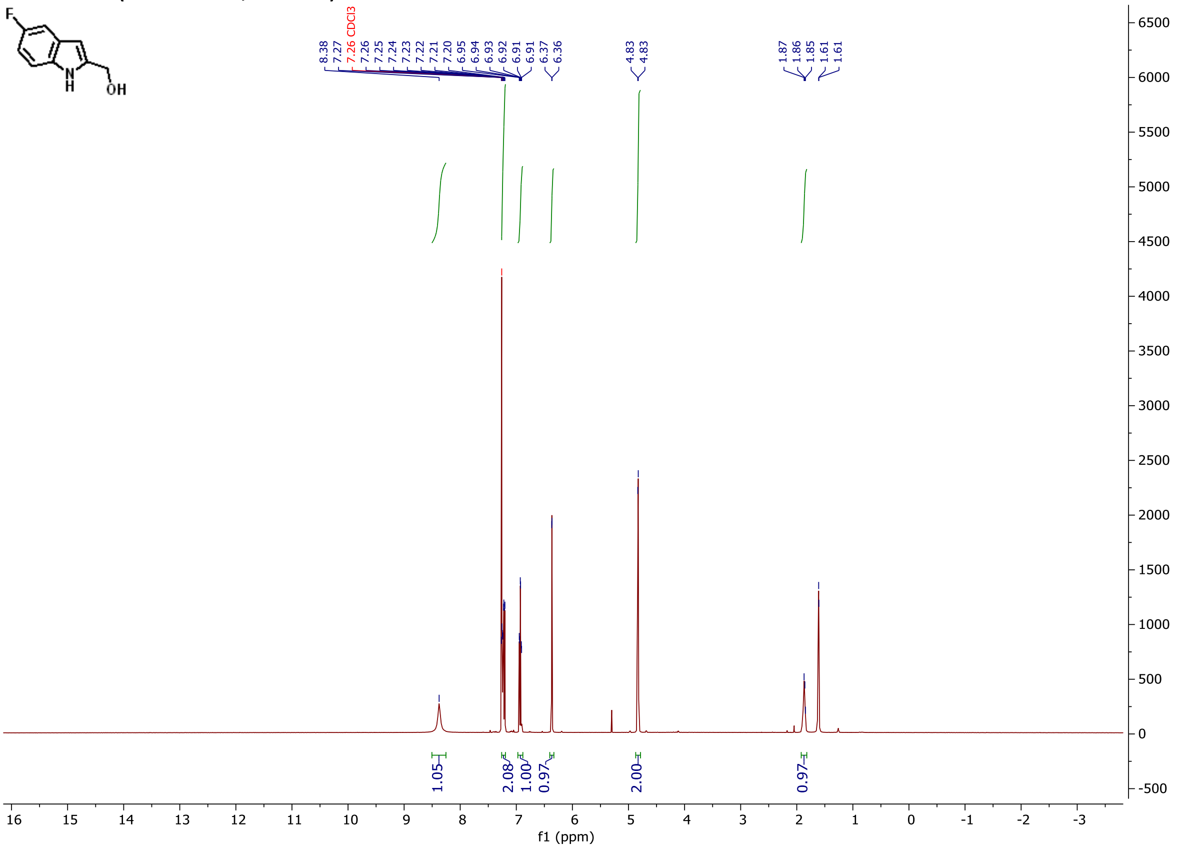

^{13}C{^{1}H} NMR (126 MHz, $CDCl_3$)

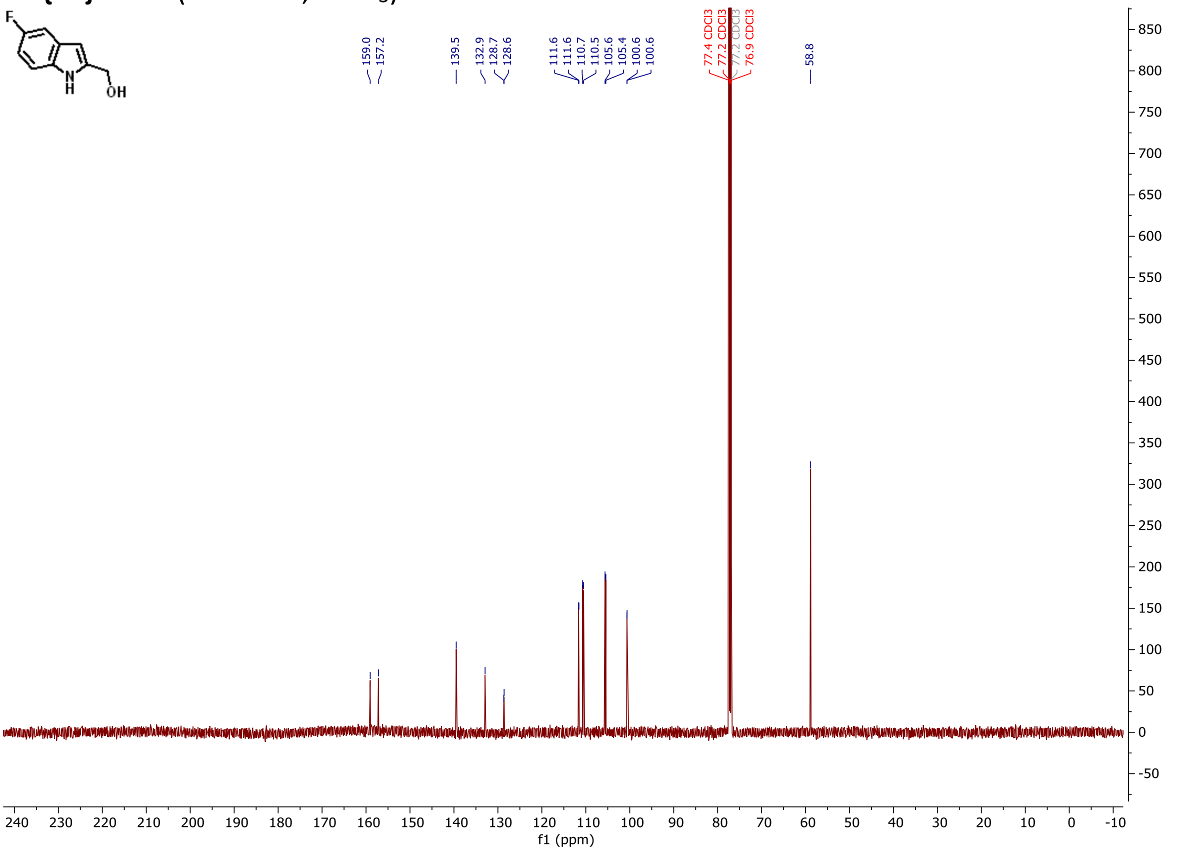

^{19}F NMR (471 MHz, $CDCl_3$)

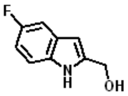

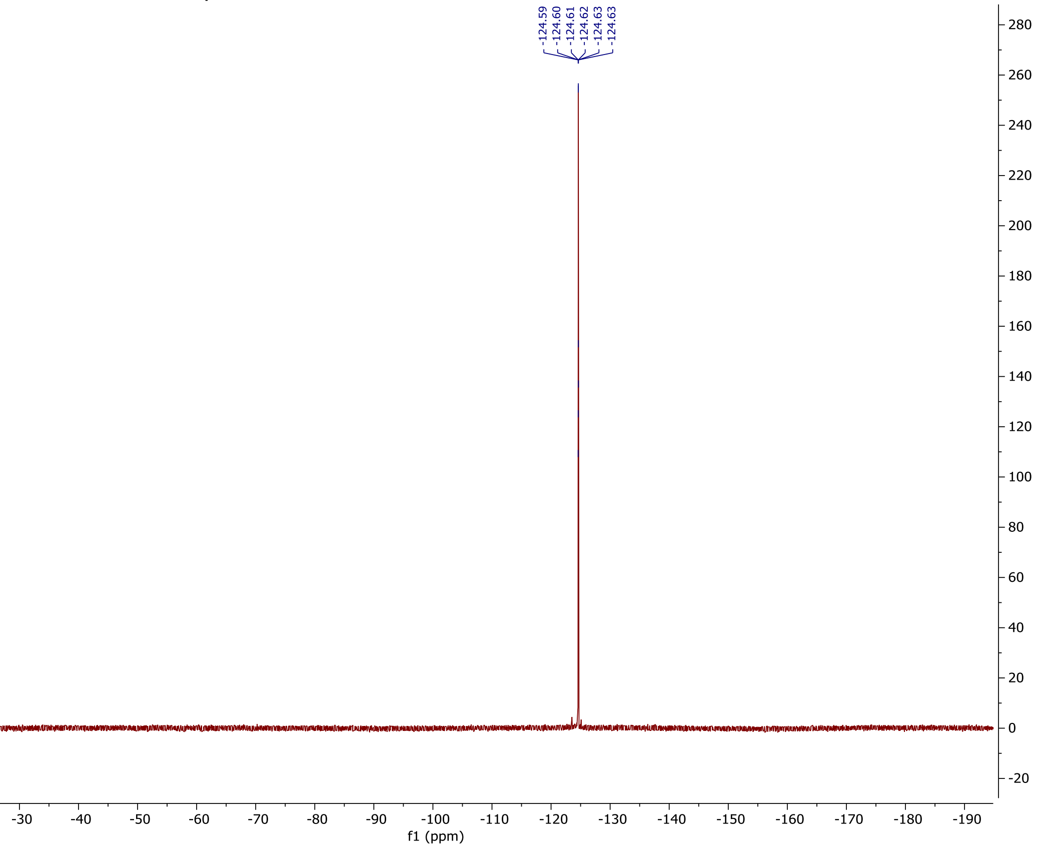

(6-methoxy-1H-indol-2-yl)methanol (1la)

^{1}H NMR (500 MHz, $CDCl_3$)

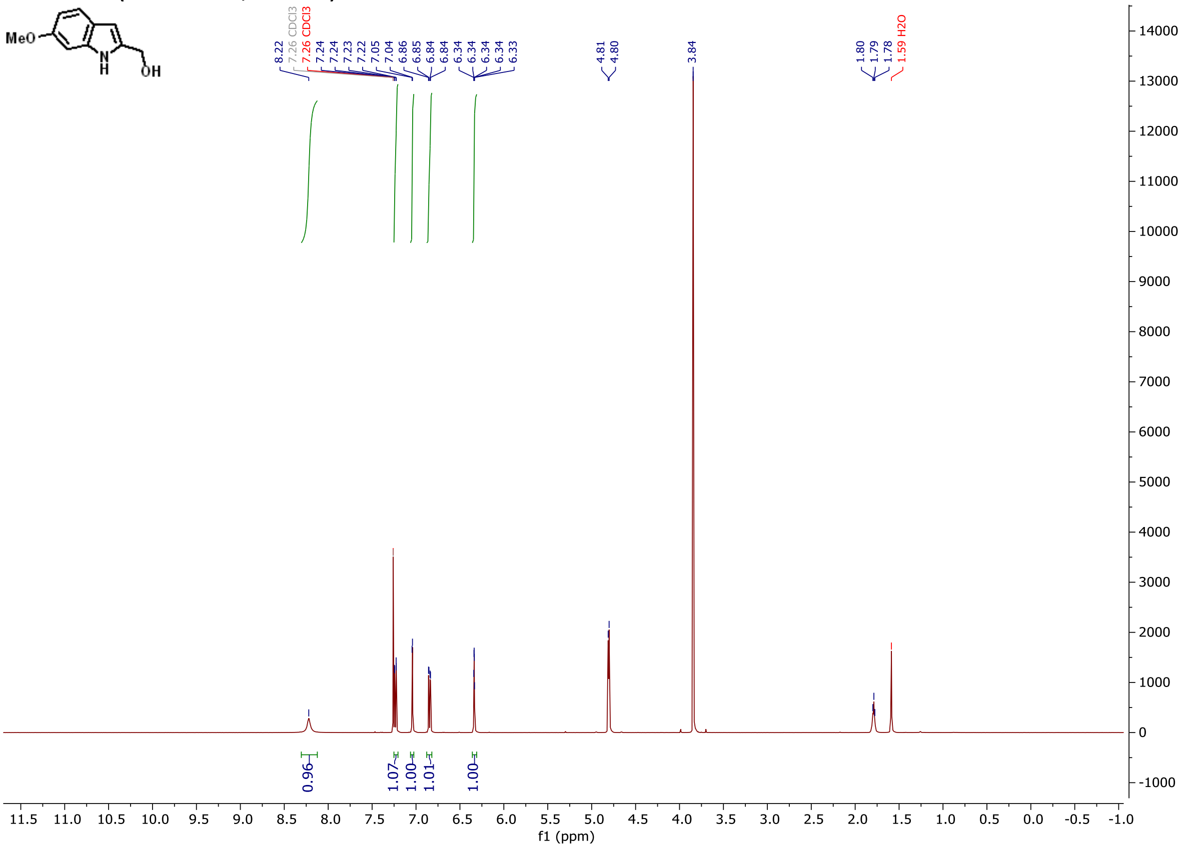

$^{13}C\{^{1}H\}$ NMR (126 MHz, $CDCl_3$)

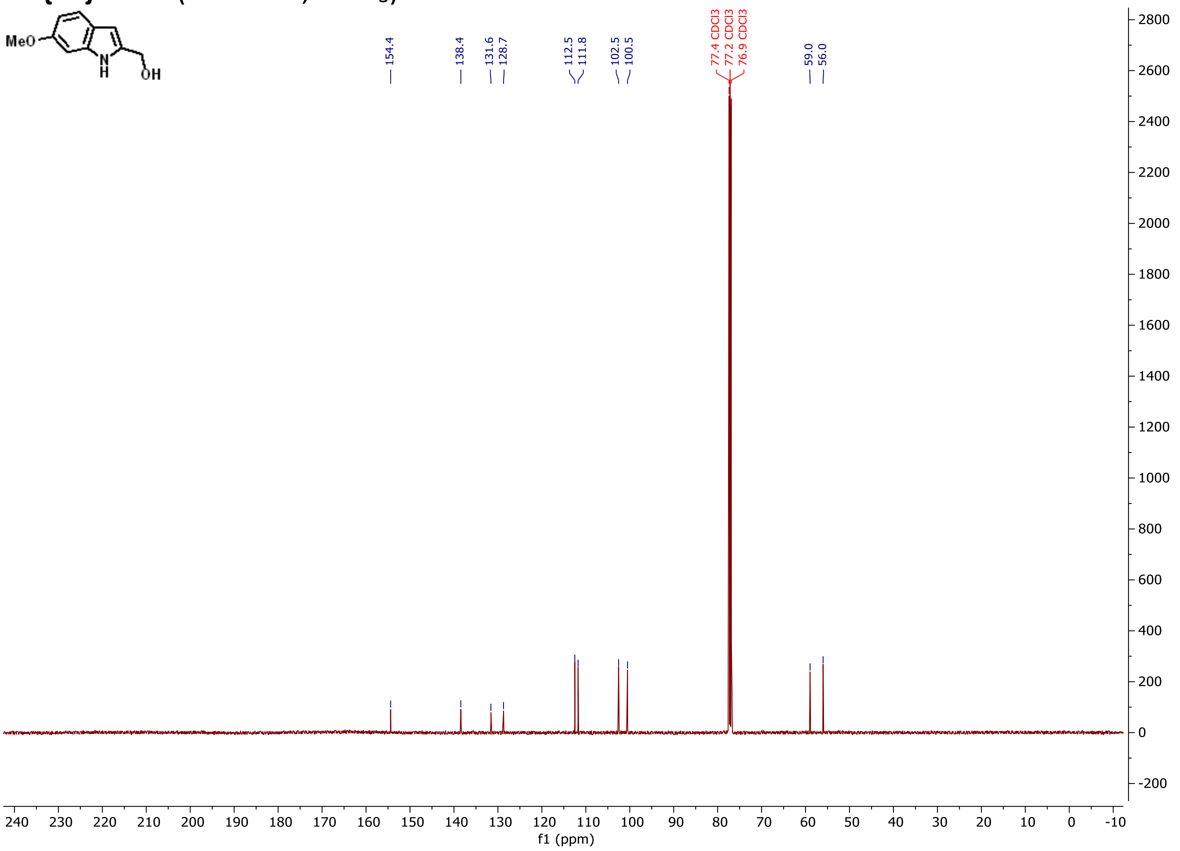

1*H*-indole-2-carbaldehyde (1ab)

^{1}H NMR (400 MHz, $CDCl_3$)

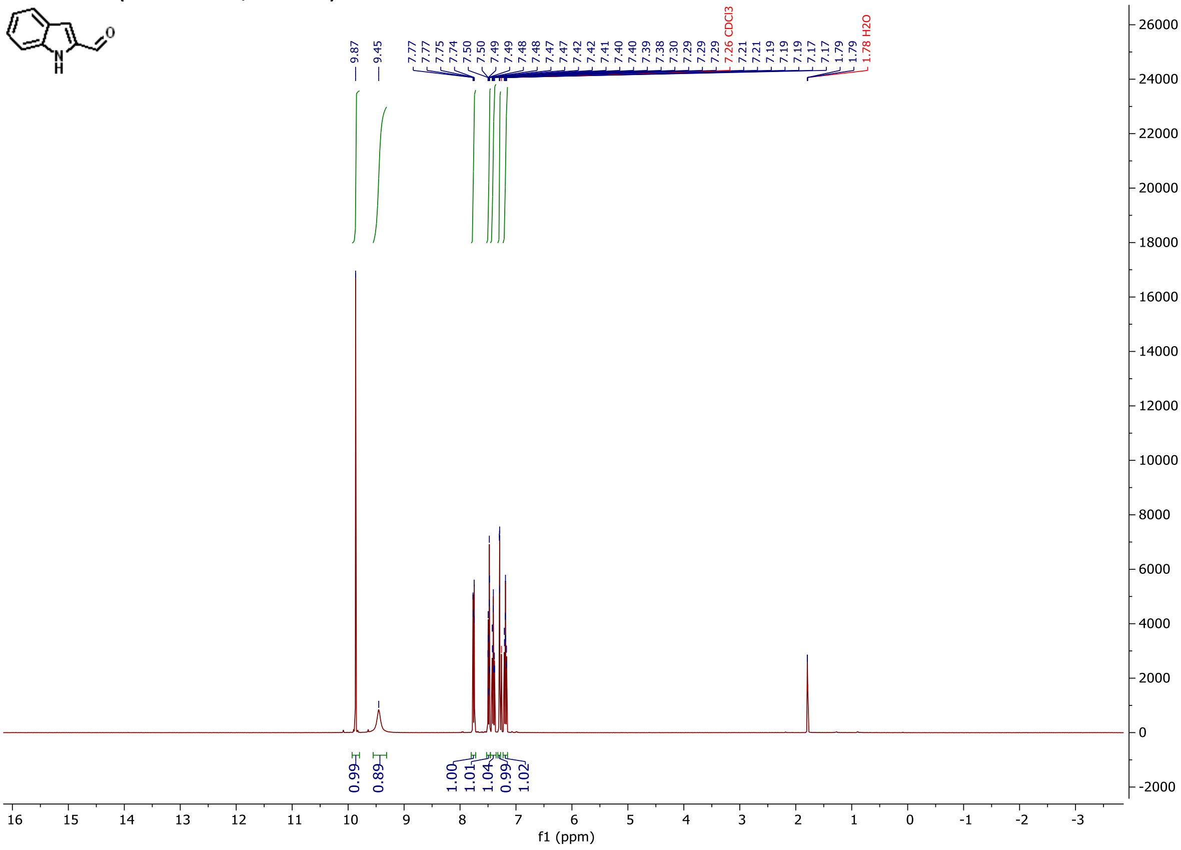

^{13}C{^{1}H} NMR (101 MHz, $CDCl_3$)

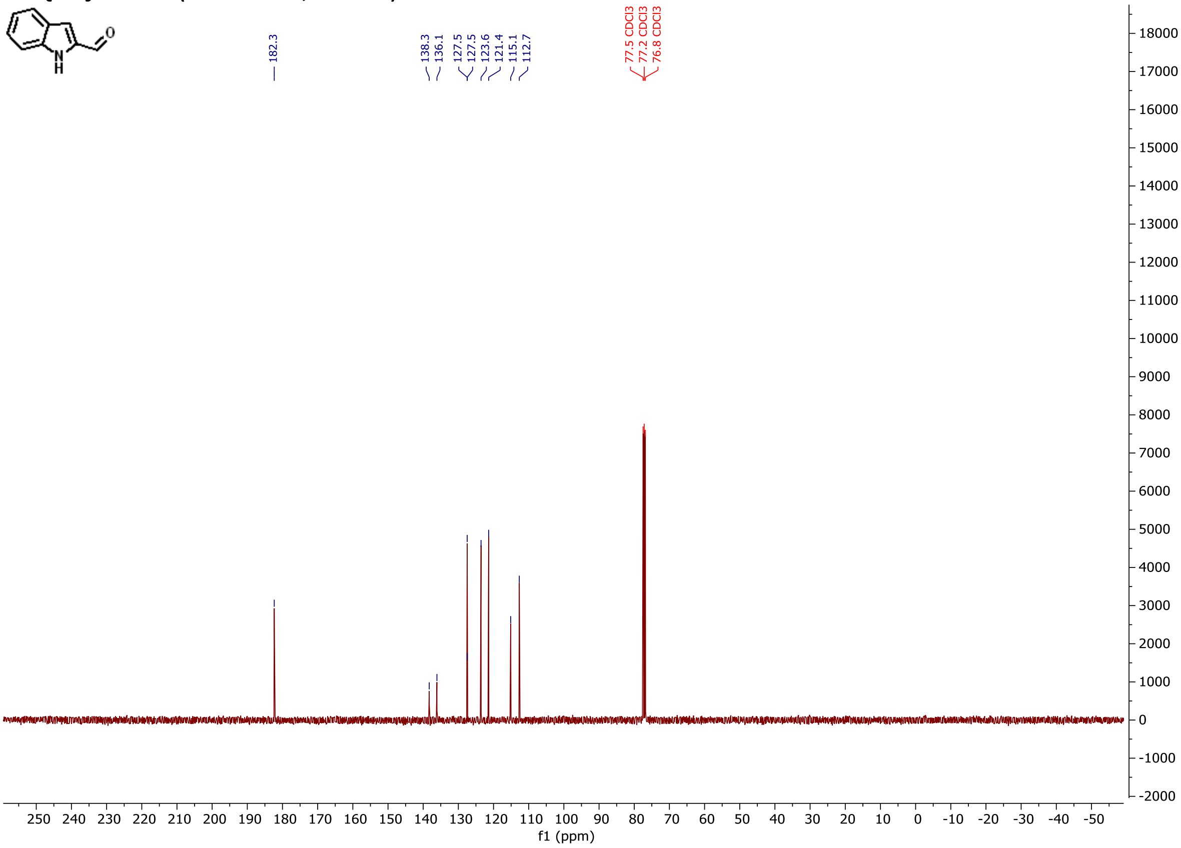

3-methyl-1H-indole-2-carbaldehyde (1hb)

^{1}H NMR (400 MHz, $CDCl_3$)

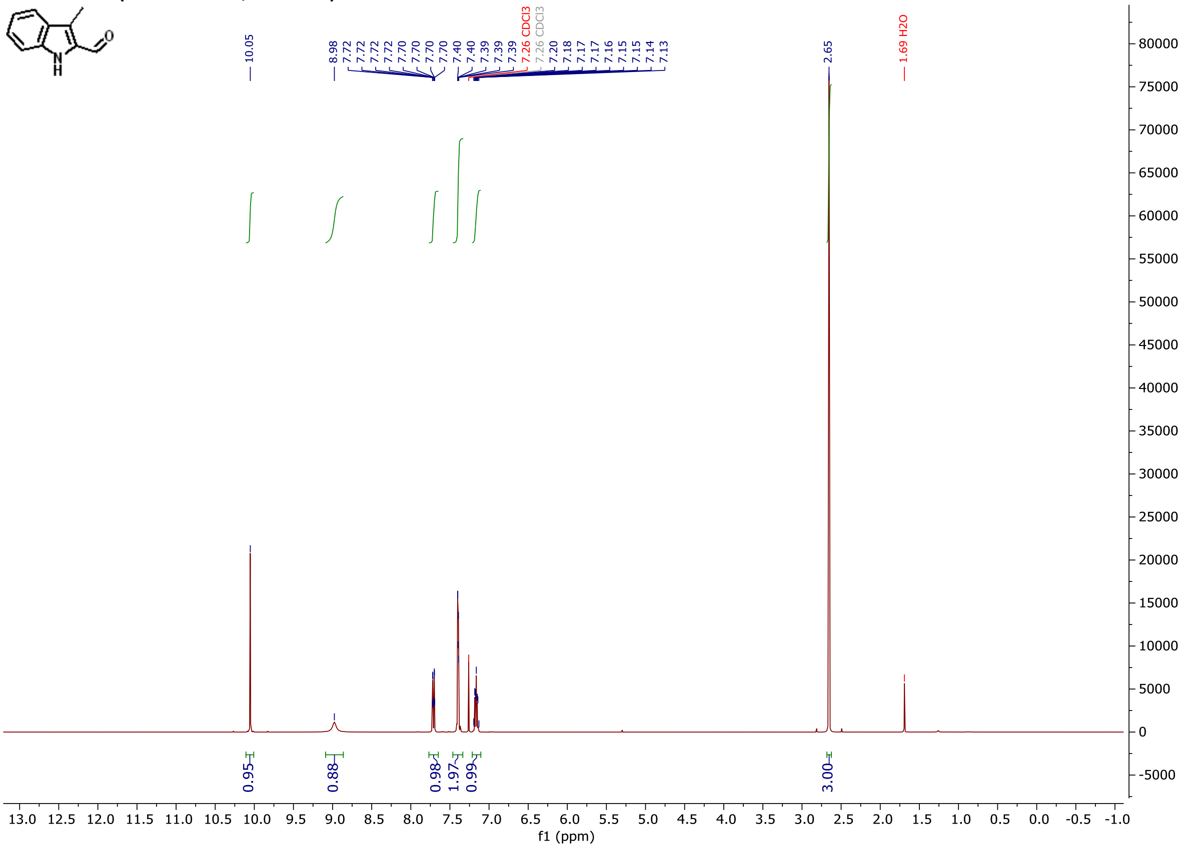

$^{13}C\{^{1}H\}$ NMR (101 MHz, $CDCl_3$)

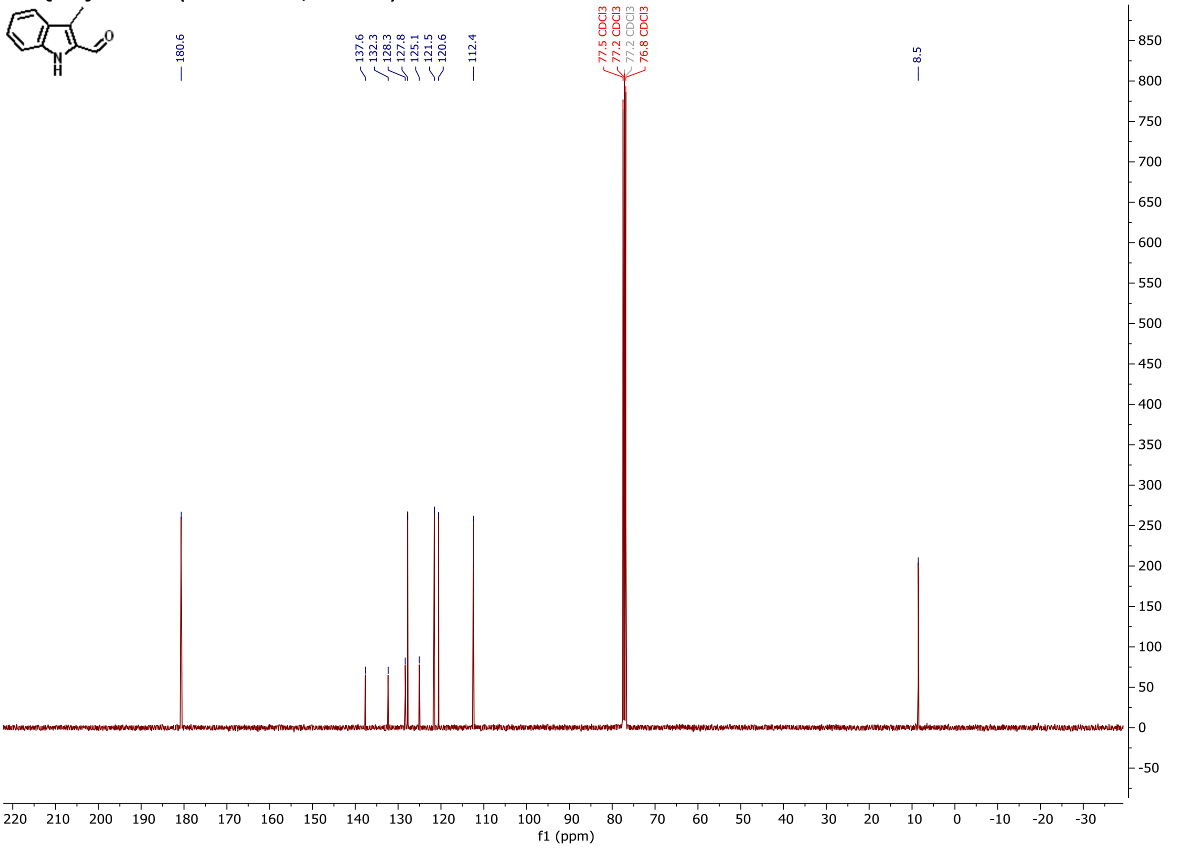

5-methyl-1H-indole-2-carbaldehyde (1ib)

^{1}H NMR (400 MHz, $CDCl_3$)

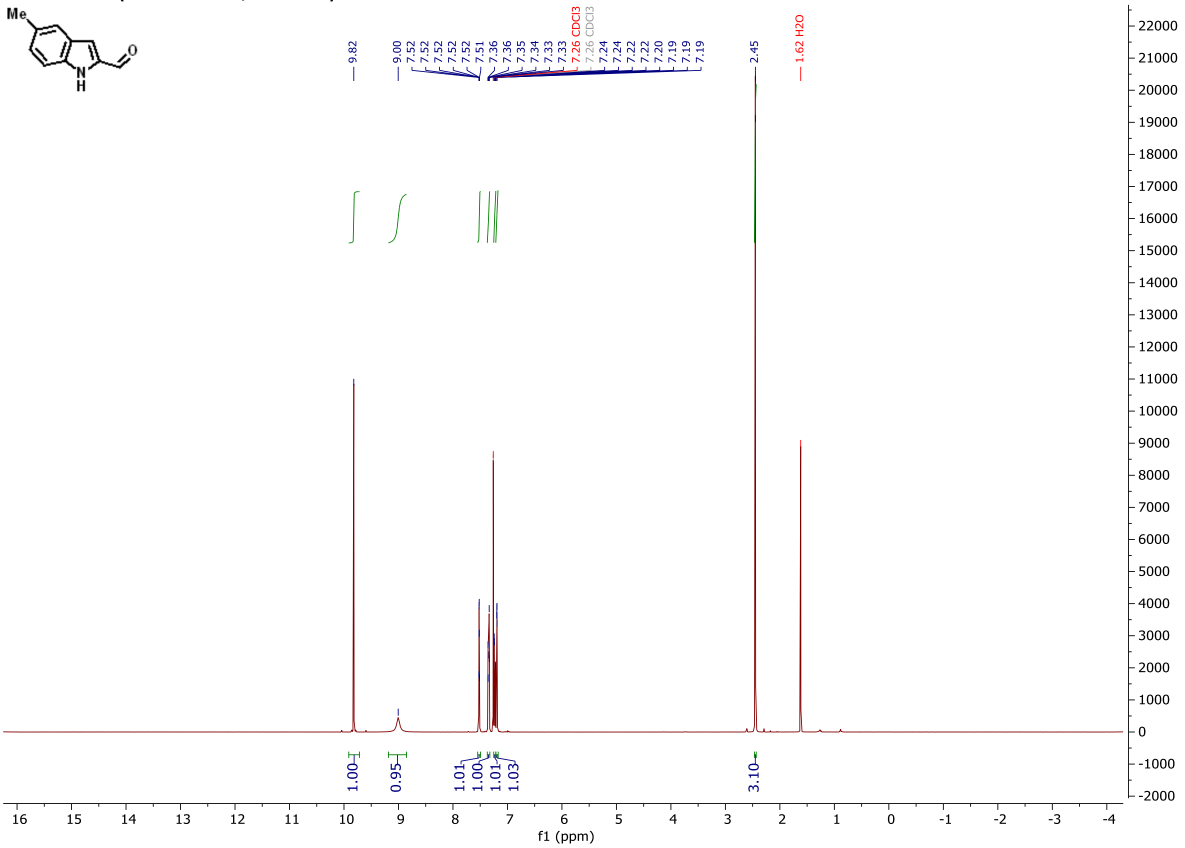

$^{13}C\{^1H\}$ NMR (101 MHz, $CDCl_3$)

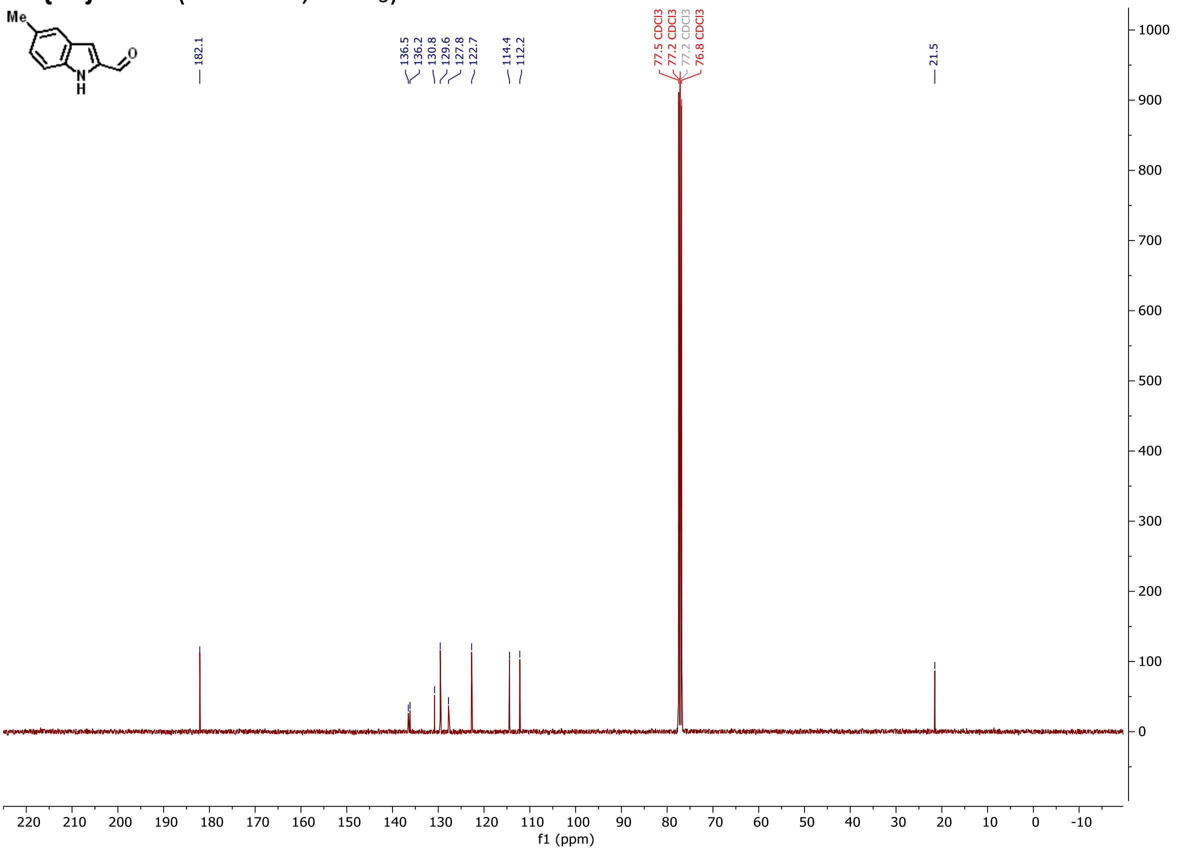

5-chloro-1H-indole-2-carbaldehyde (1jb)

^{1}H NMR (400 MHz, $CDCl_3$)

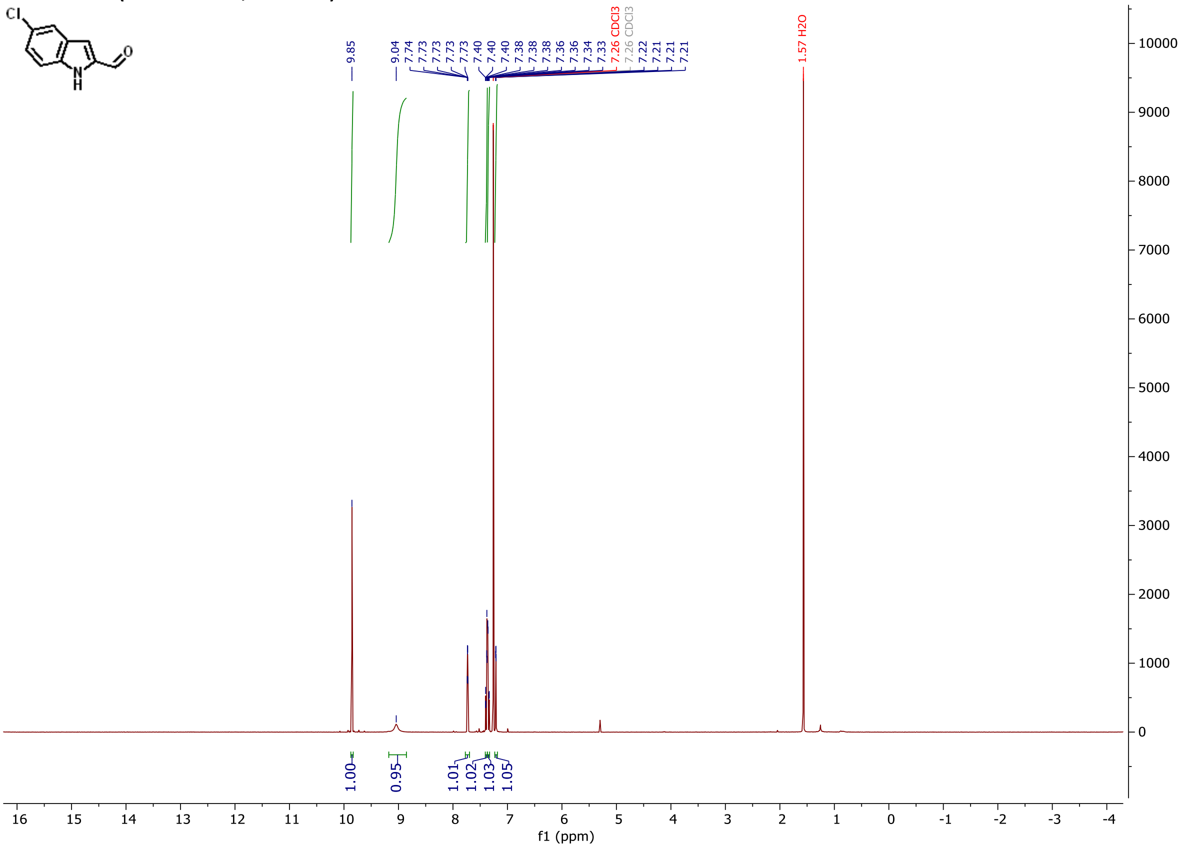

^{13}C{^{1}H} NMR (101 MHz, $CDCl_3$)

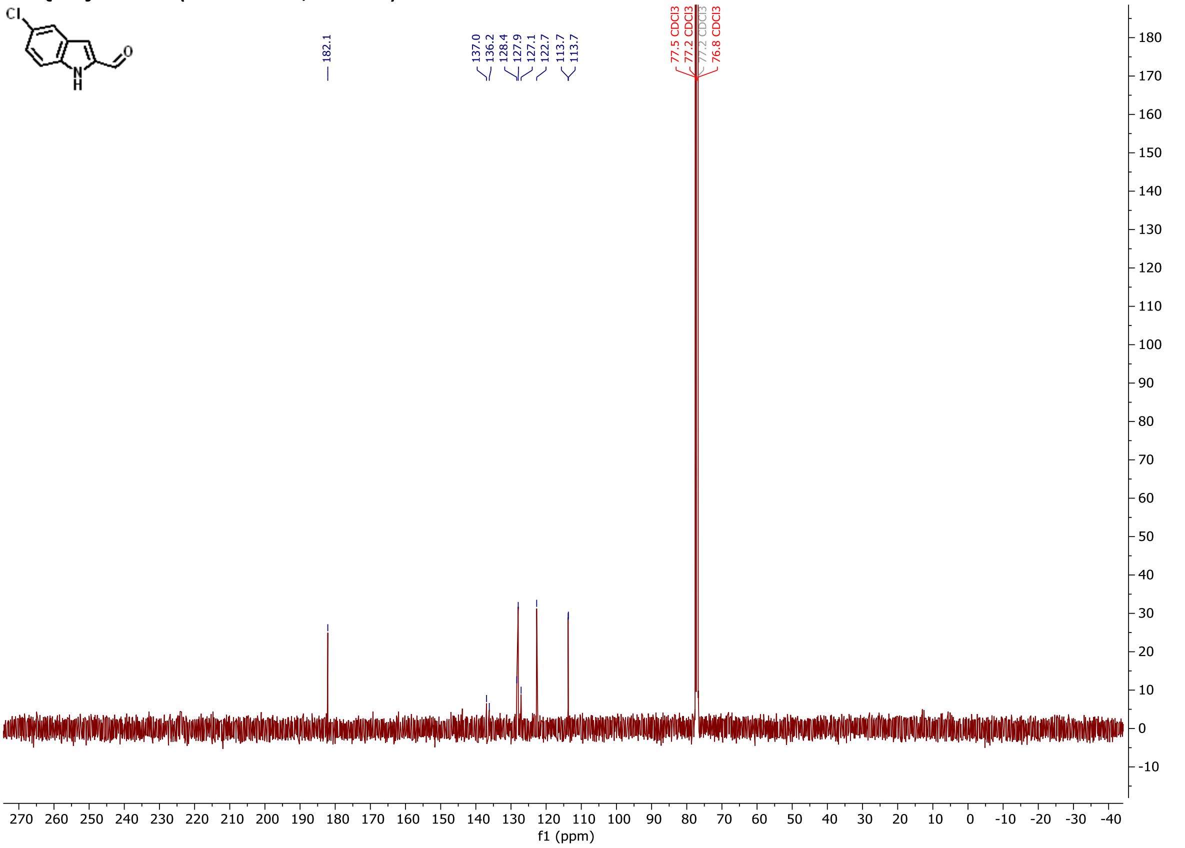

5-fluoro-1H-indole-2-carbaldehyde (1kb)

^{1}**H NMR** (400 MHz, $CDCl_3$)

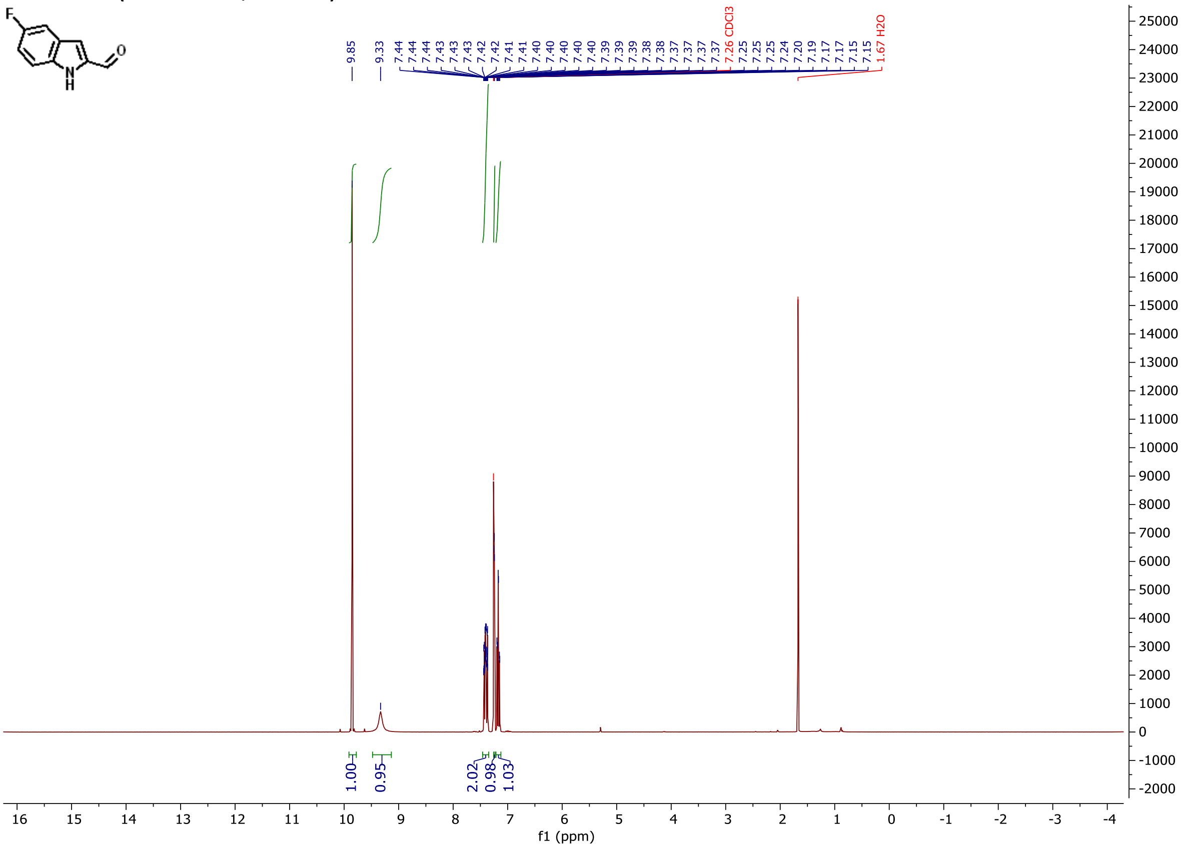

^{13}**C{^{1}H} NMR** (126 MHz, $CDCl_3$)

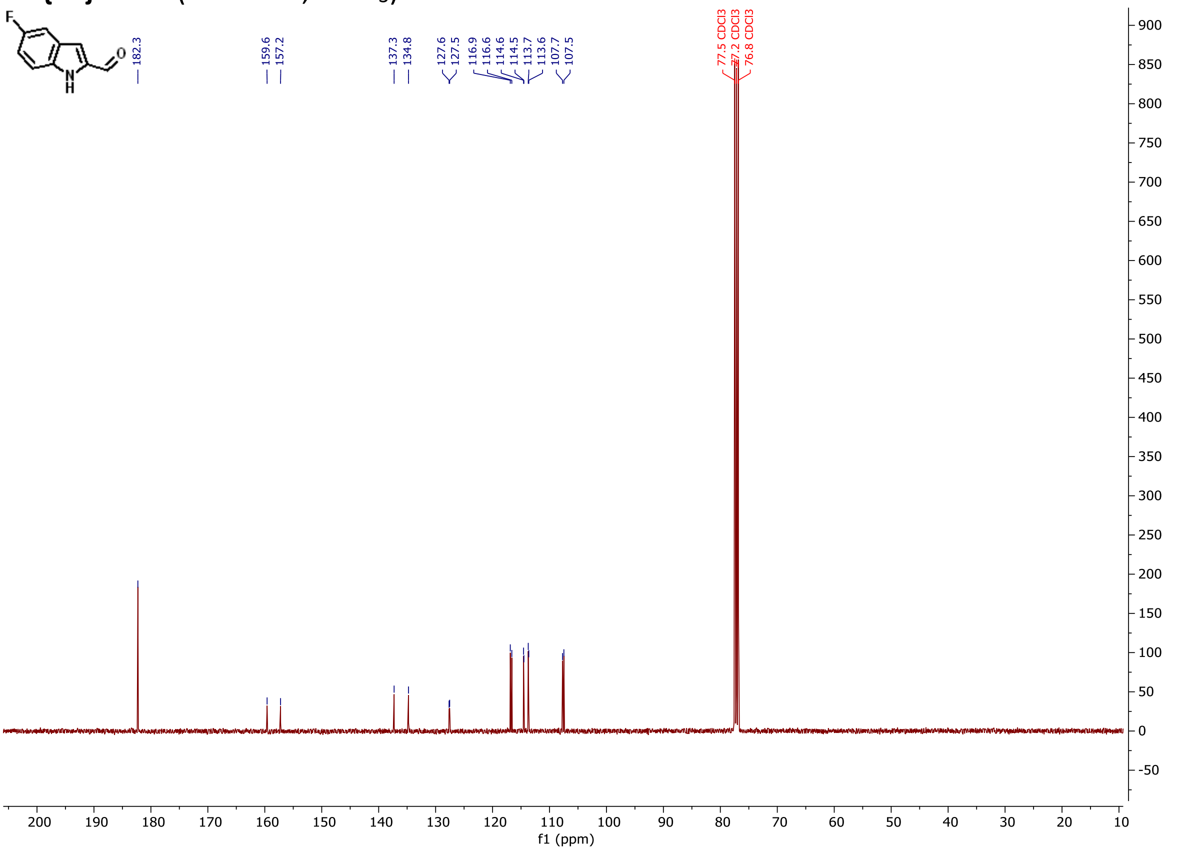

^{19}F NMR (471 MHz, $CDCl_3$)

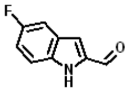

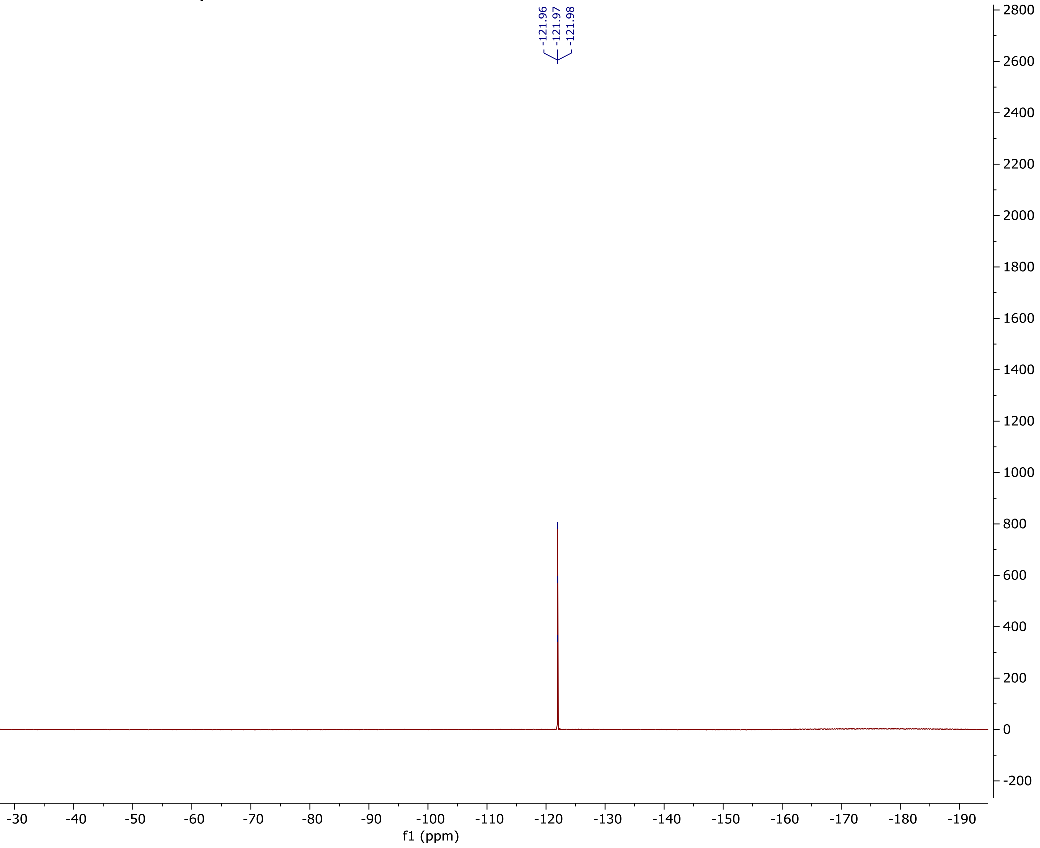

6-methoxy-1H-indole-2-carbaldehyde (1lb)

^{1}H NMR (500 MHz, $CDCl_3$)

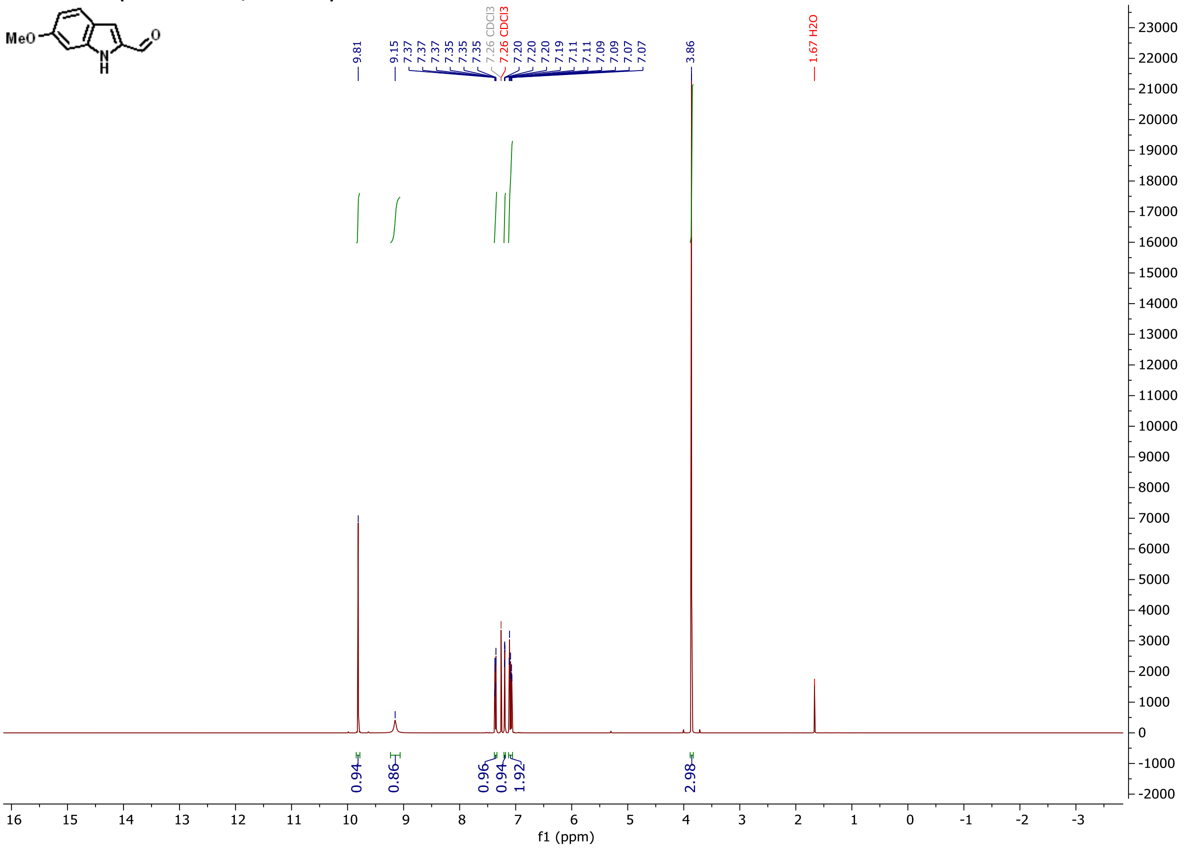

$^{13}C\{^1H\}$ NMR (126 MHz, $CDCl_3$)

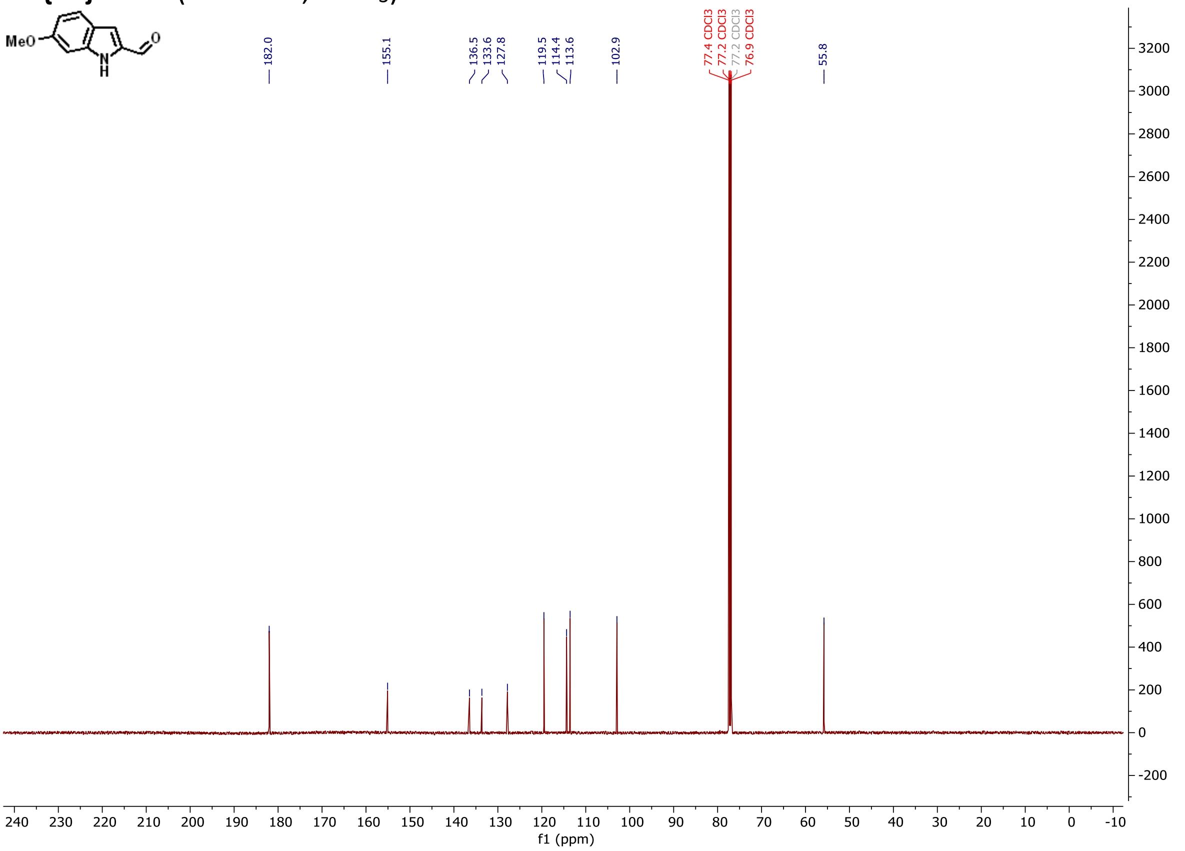

1-((4-nitrophenyl)sulfonyl)-1H-indole-2-carbaldehyde (1a)

^{1}H NMR (400 MHz, $CDCl_3$)

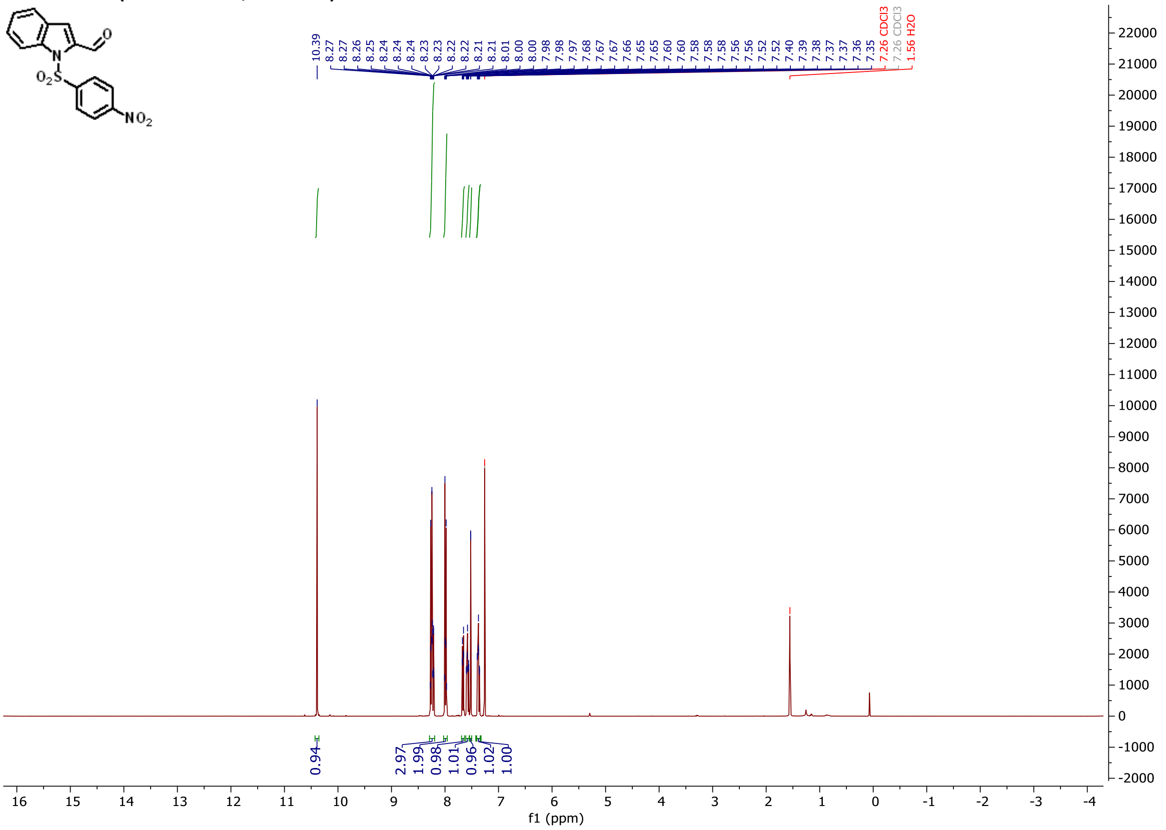

$^{13}C\{^1H\}$ NMR (101 MHz, $CDCl_3$)

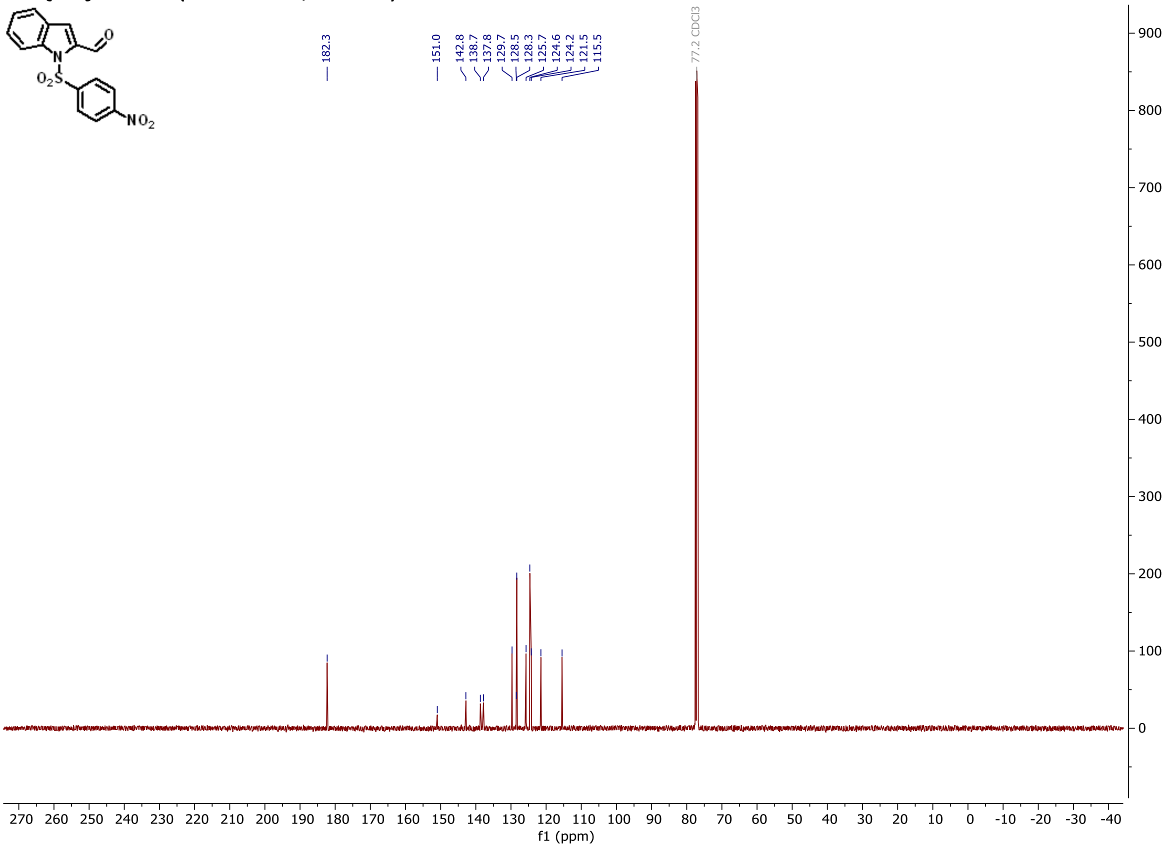

1-((2-fluoro-4-nitrophenyl)sulfonyl)-1H-indole-2-carbaldehyde (1b)

^{1}H NMR (400 MHz, $CDCl_3$)

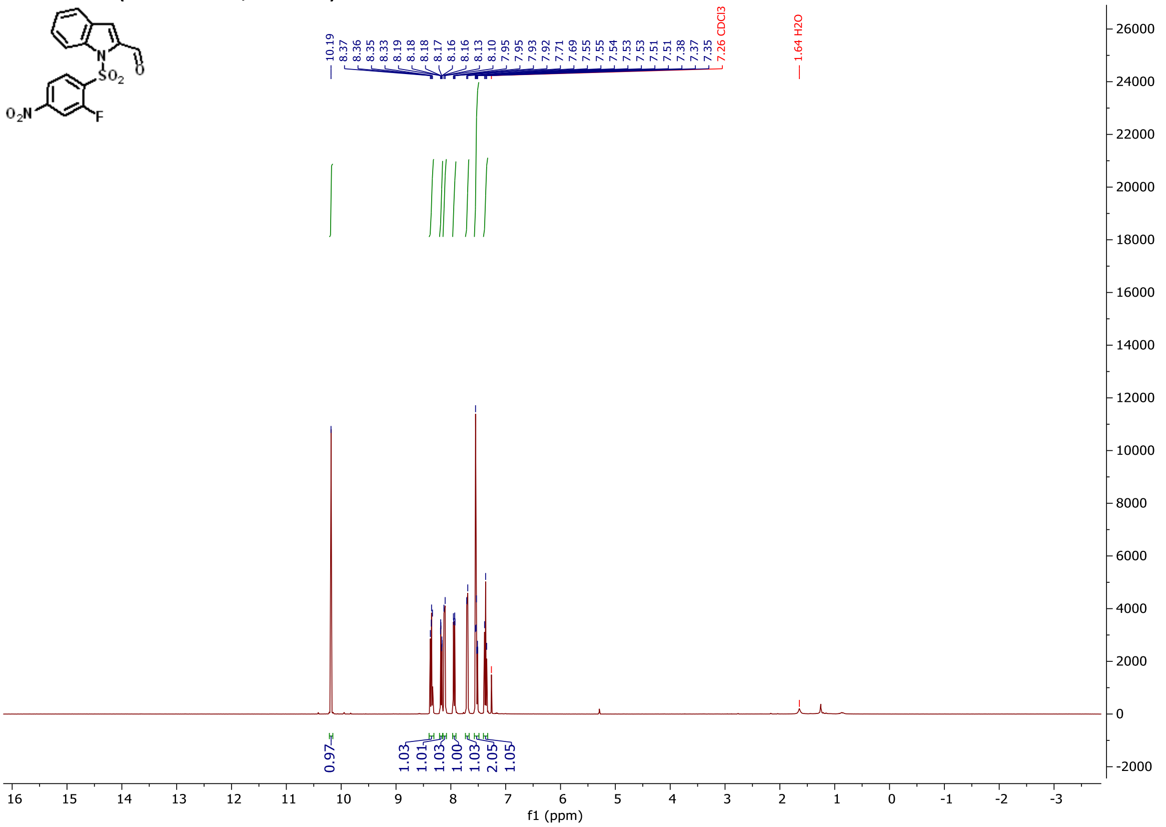

^{13}C{^{1}H} NMR (101 MHz, $CDCl_3$)

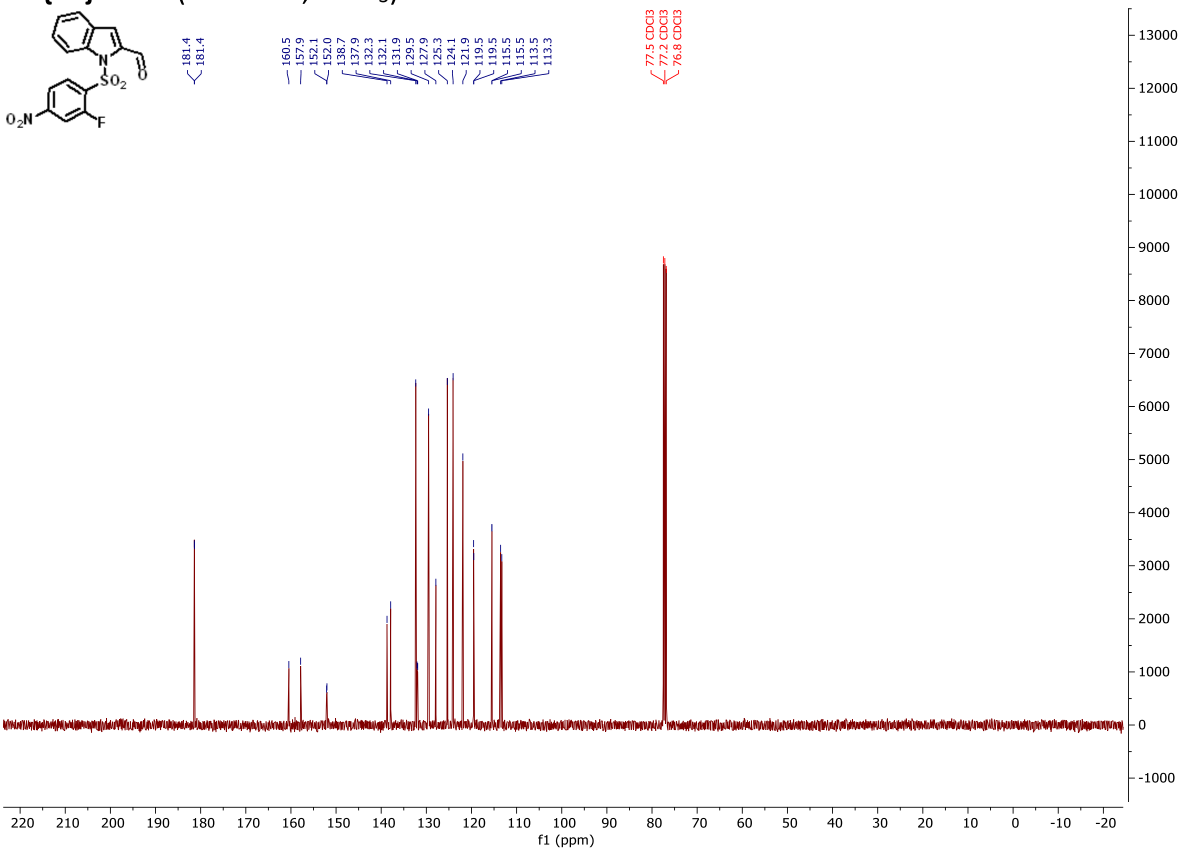

^{19}F NMR (471 MHz, $CDCl_3$)

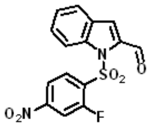

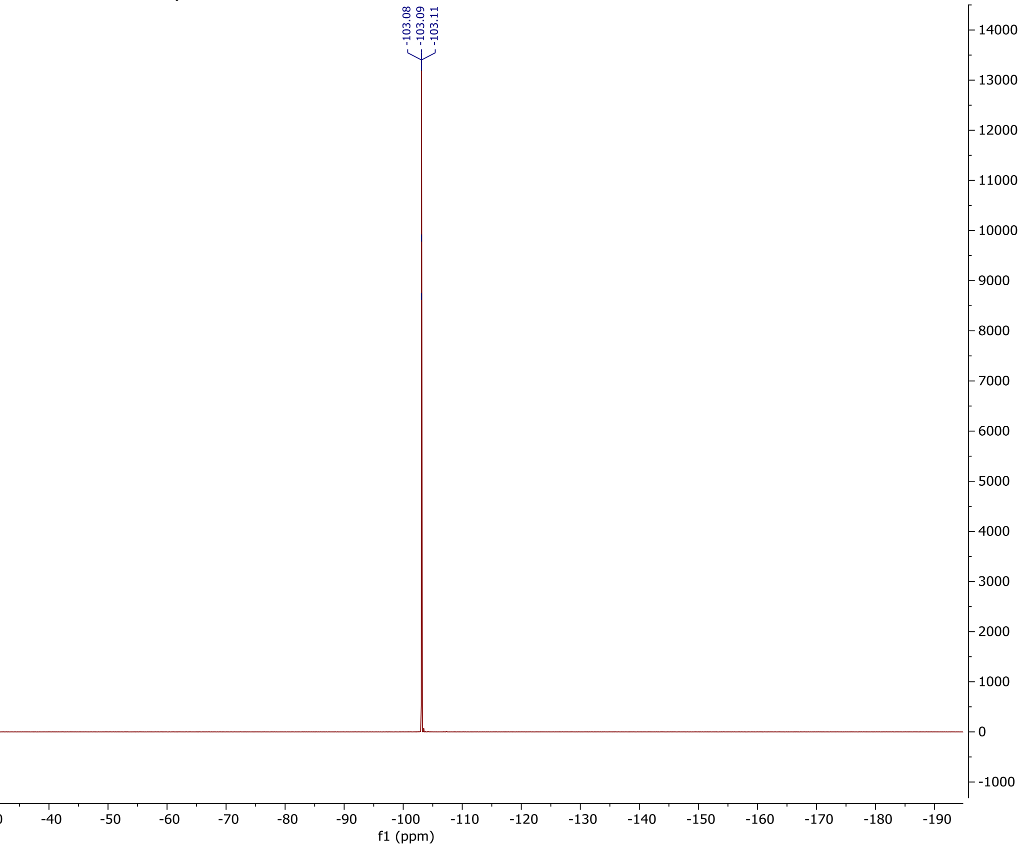

1-((2-methoxy-4-nitrophenyl)sulfonyl)-1H-indole-2-carbaldehyde (1c)

^{1}H NMR (500 MHz, $CDCl_3$)

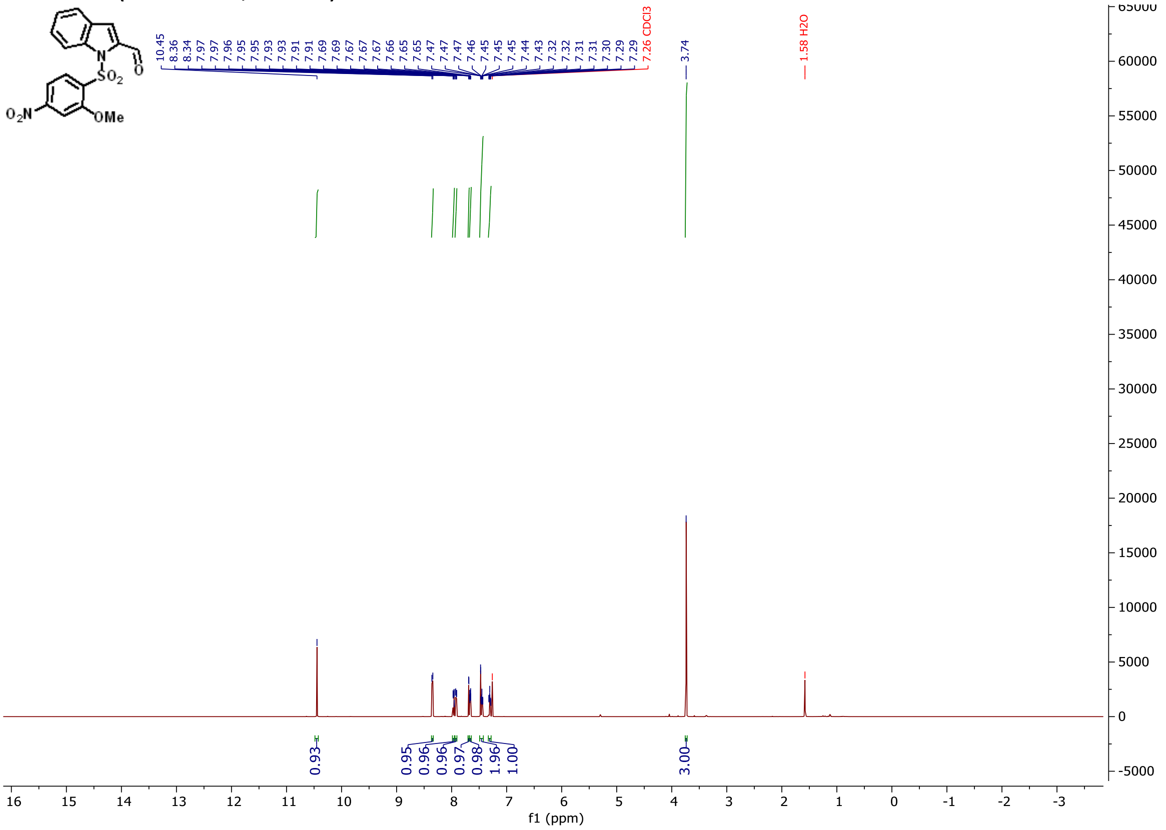

^{13}C{^{1}H} NMR (126 MHz, $CDCl_3$)

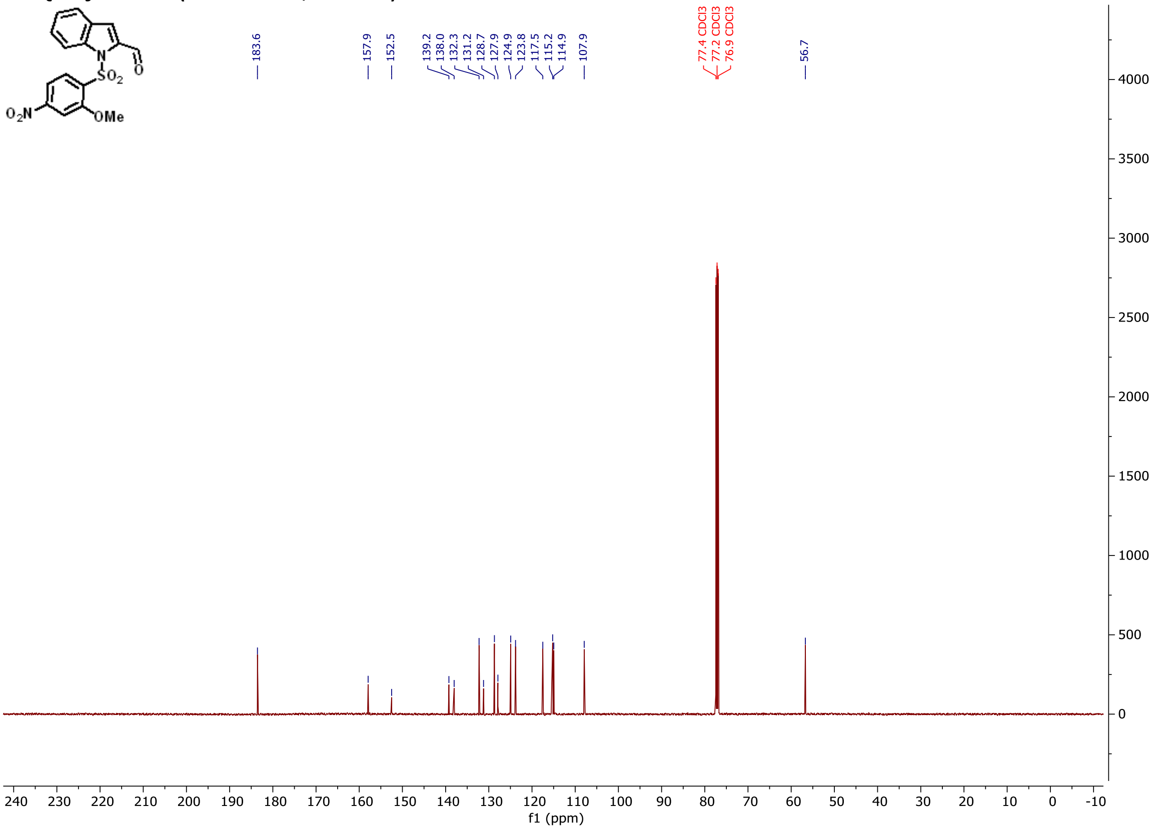

1-((4-nitro-3-(trifluoromethyl)phenyl)sulfonyl)-1H-indole-2-carbaldehyde (1d)

^{1}**H NMR** (400 MHz, $CDCl_3$)

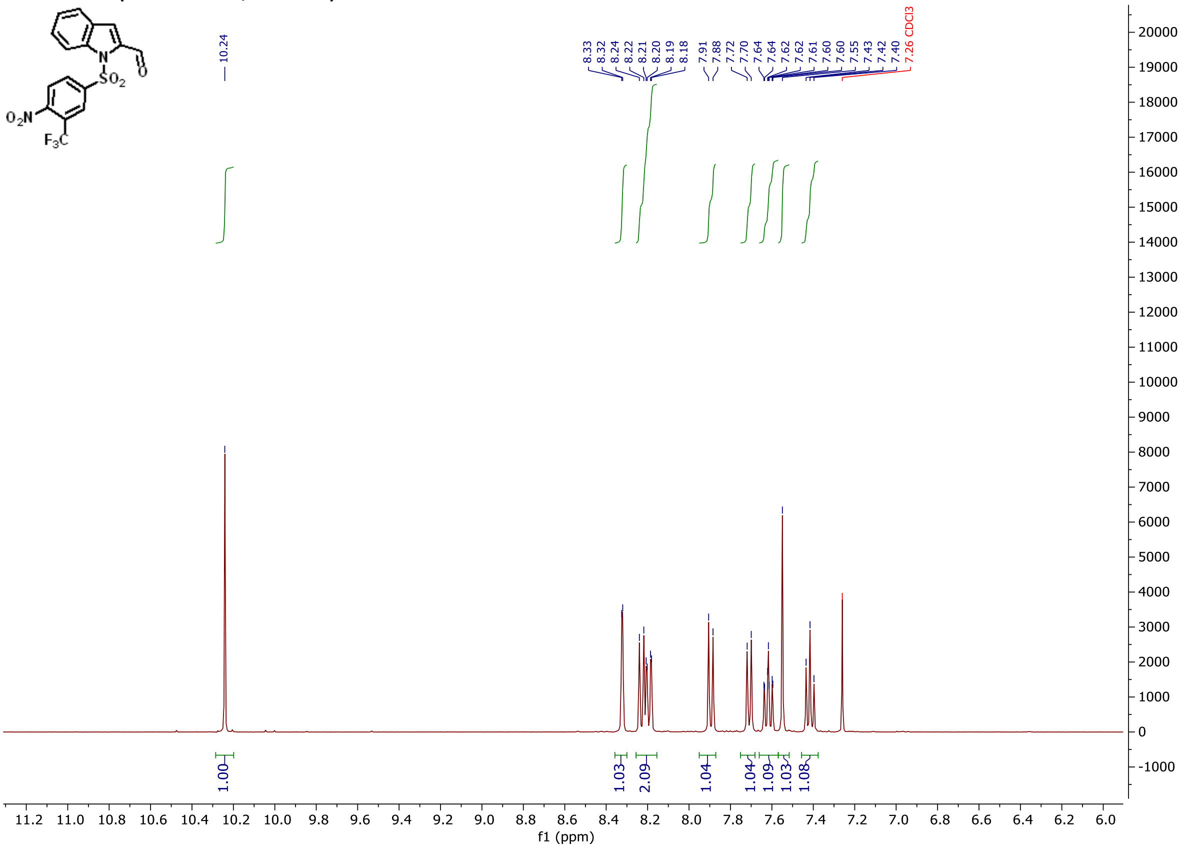

^{13}**C{**^{1}**H} NMR** (101 MHz, $CDCl_3$)

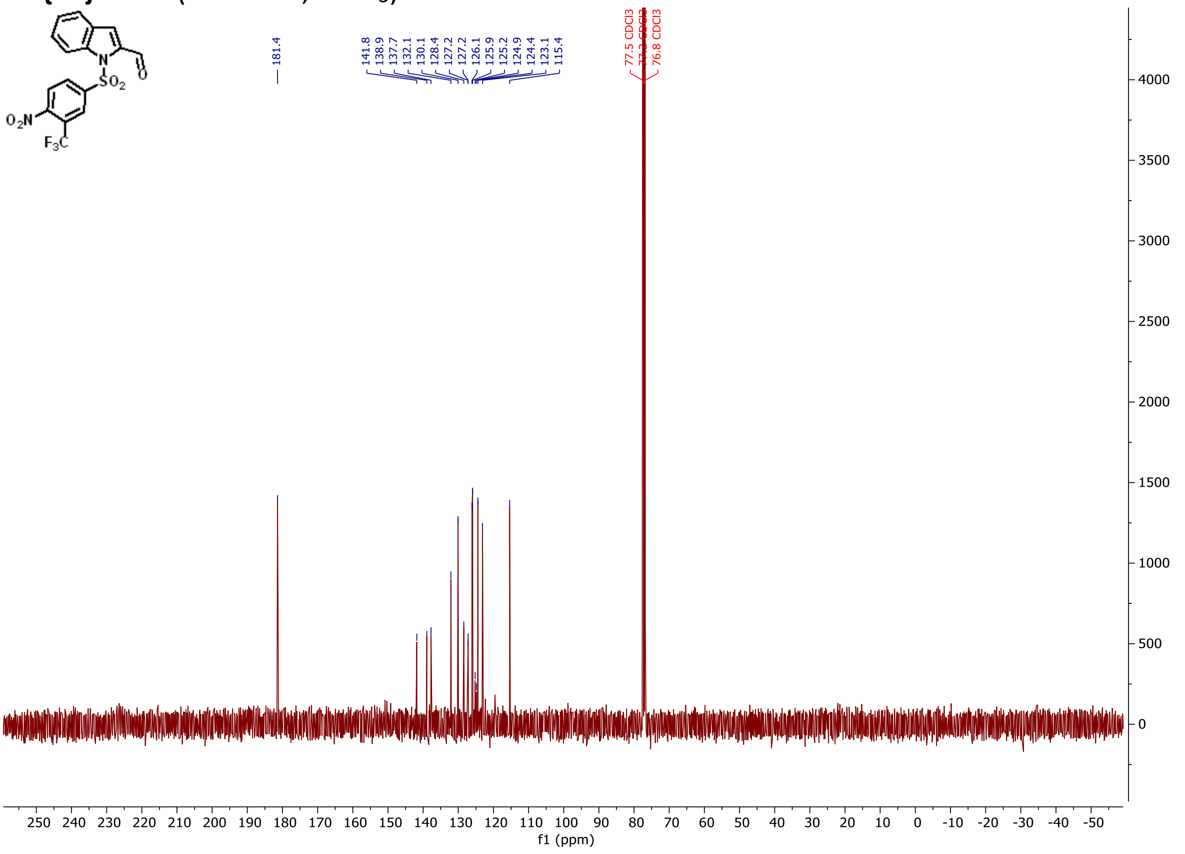

^{19}F NMR (376 MHz, $CDCl_3$)

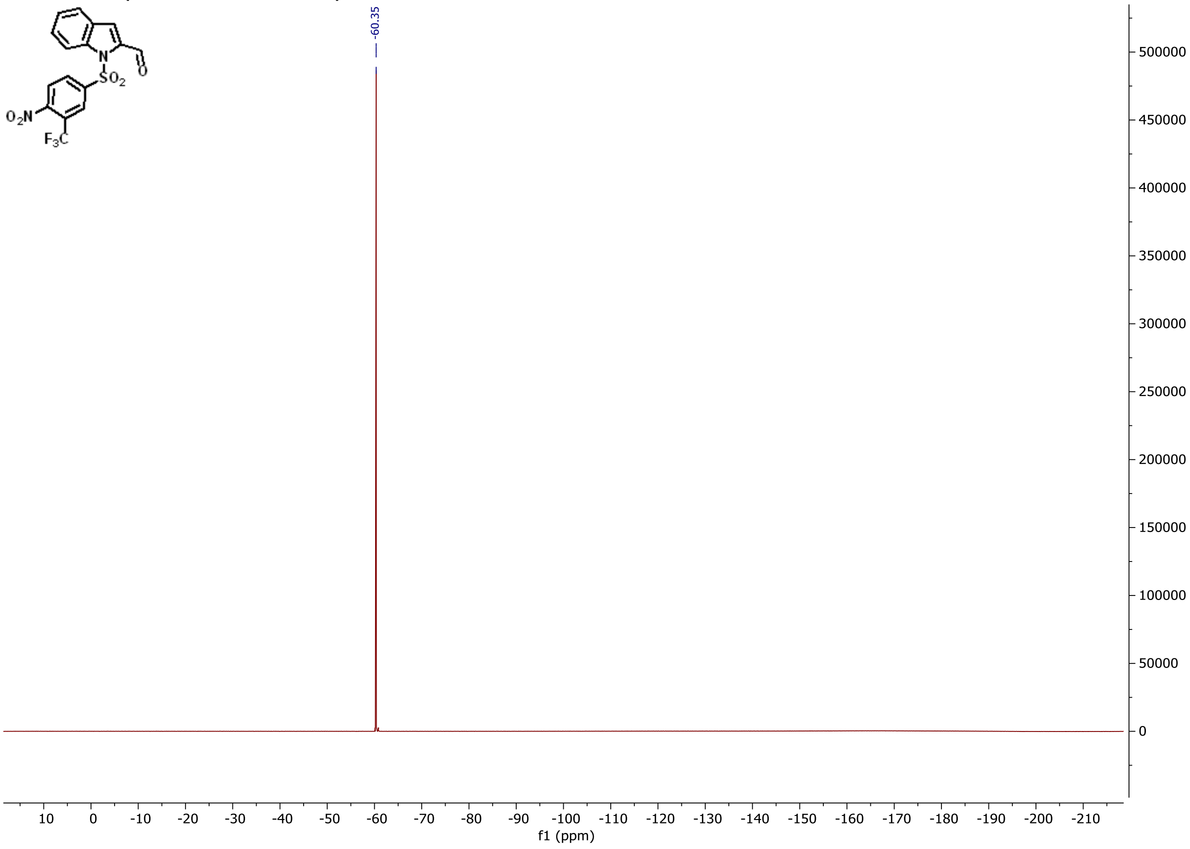

1-((2-nitrophenyl)sulfonyl)-1H-indole-2-carbaldehyde (1e)

^{1}H NMR (500 MHz, $CDCl_3$)

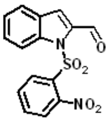

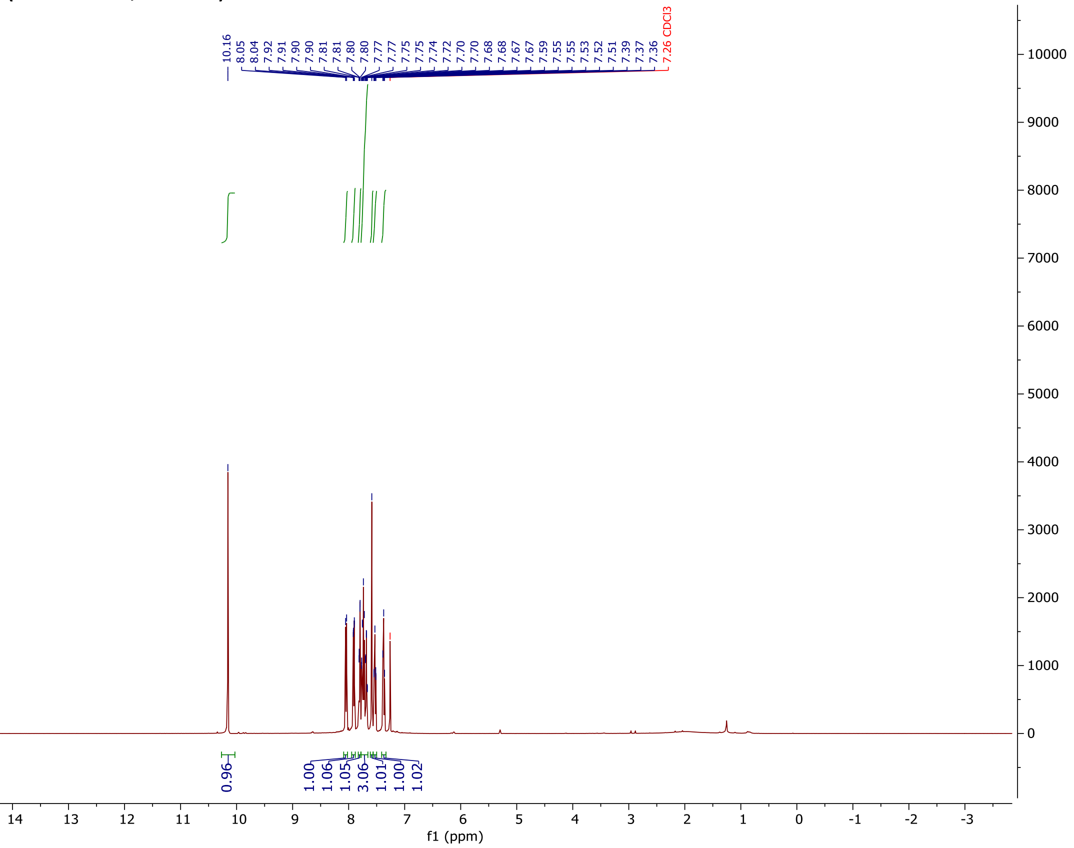

^{13}C{^{1}H} NMR (126 MHz, $CDCl_3$)

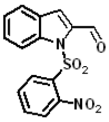

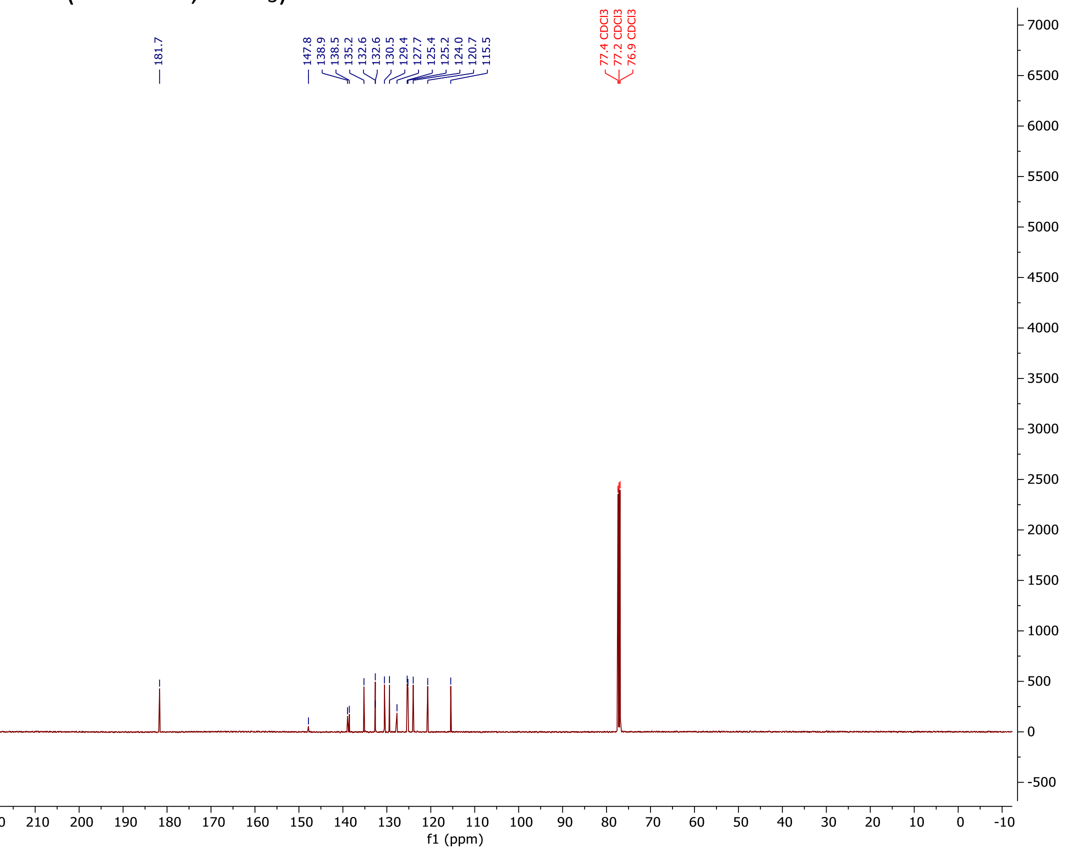

1-((4-bromo-2-nitrophenyl)sulfonyl)-1H-indole-2-carbaldehyde (1f)

^{1}H NMR (500 MHz, $CDCl_3$)

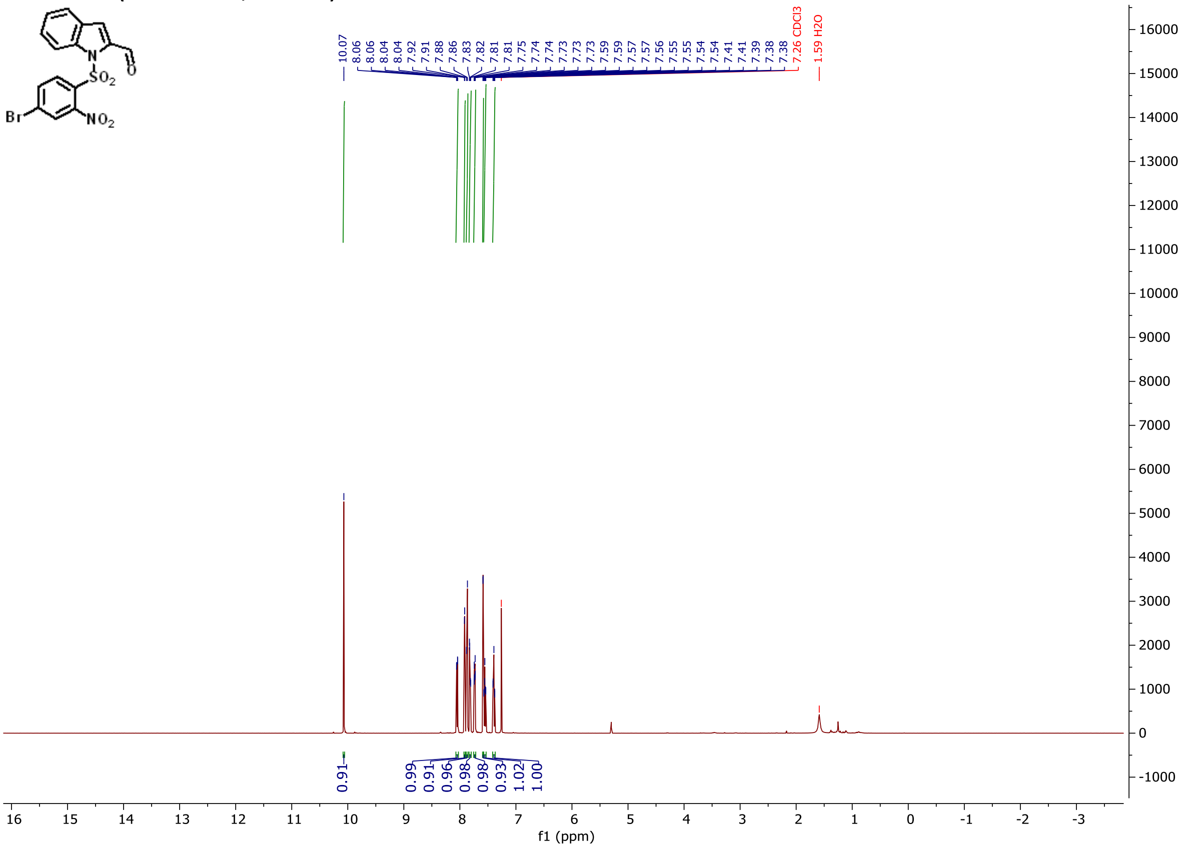

^{13}C{^{1}H} NMR (500 MHz, $CDCl_3$)

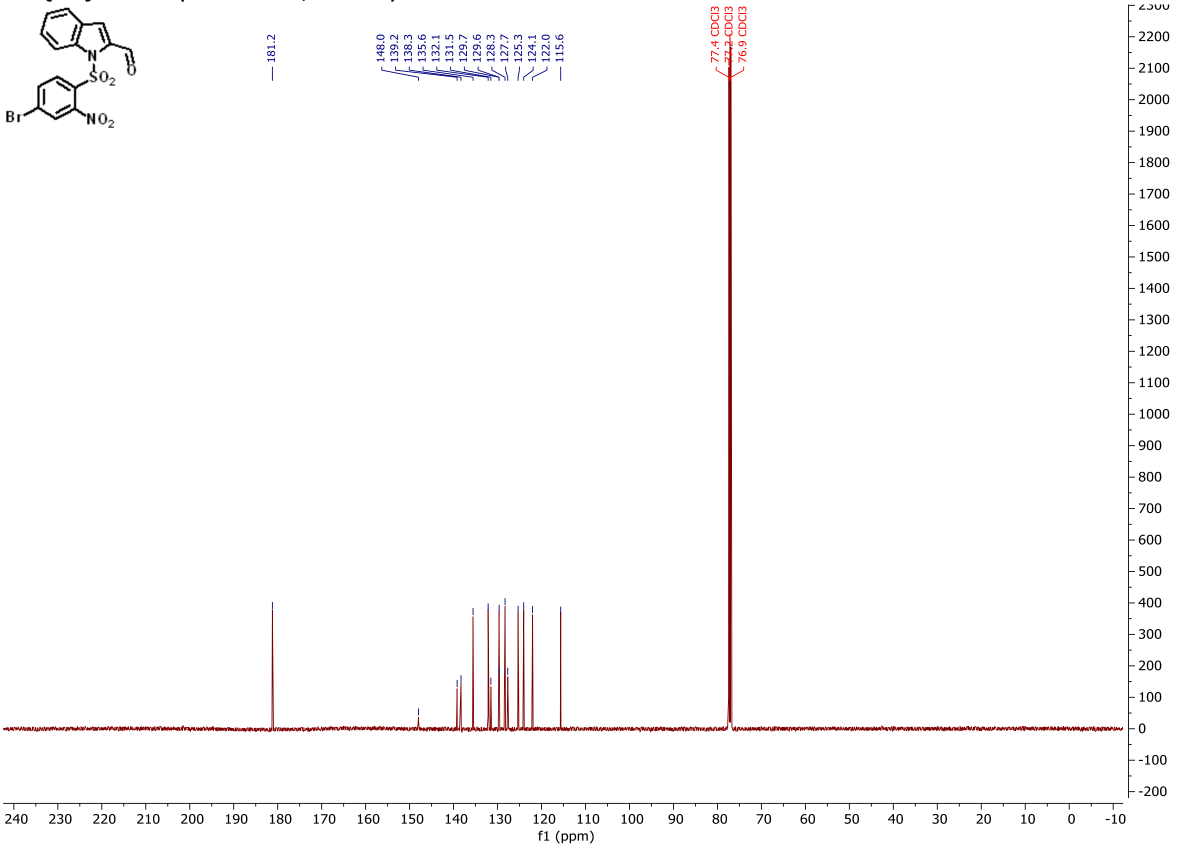

1-(pyridin-2-ylsulfonyl)-1H-indole-2-carbaldehyde (1g)

¹H NMR (500 MHz, $CDCl_3$)

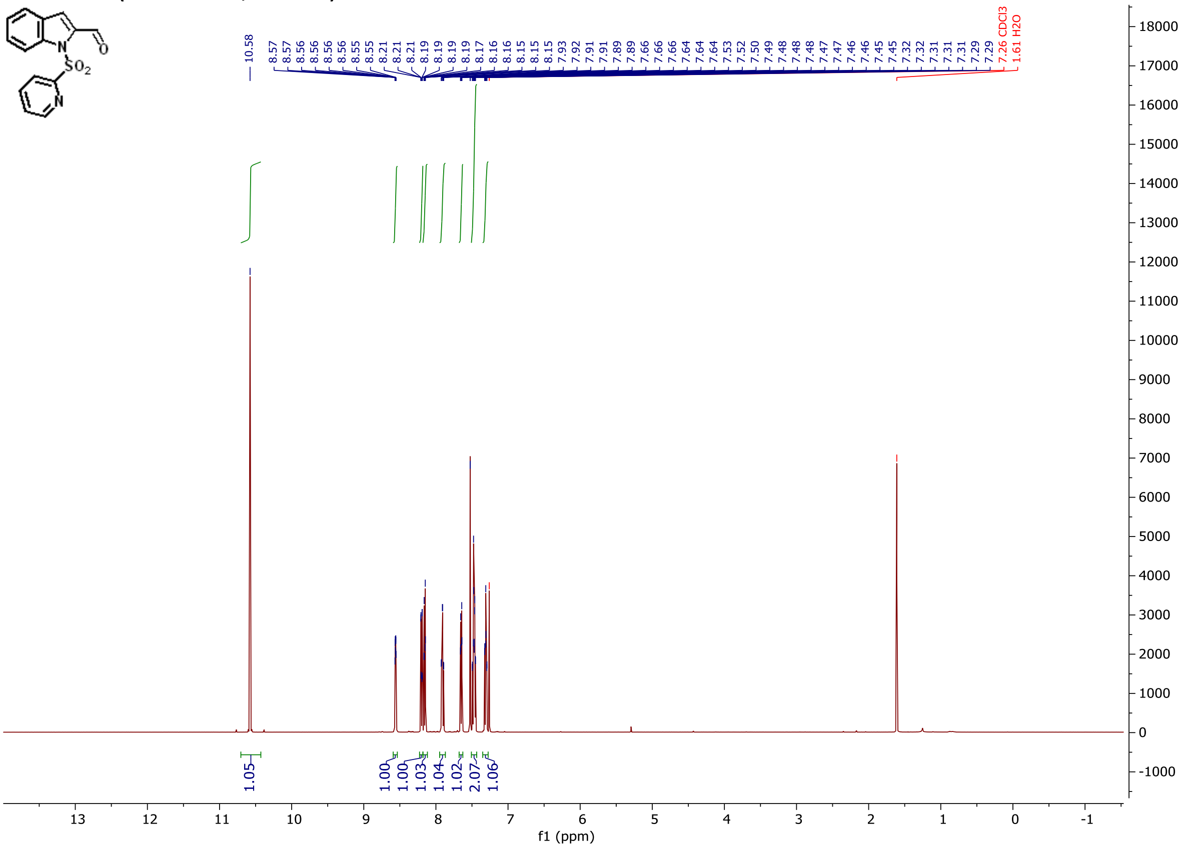

¹³C{¹H} NMR (126 MHz, $CDCl_3$)

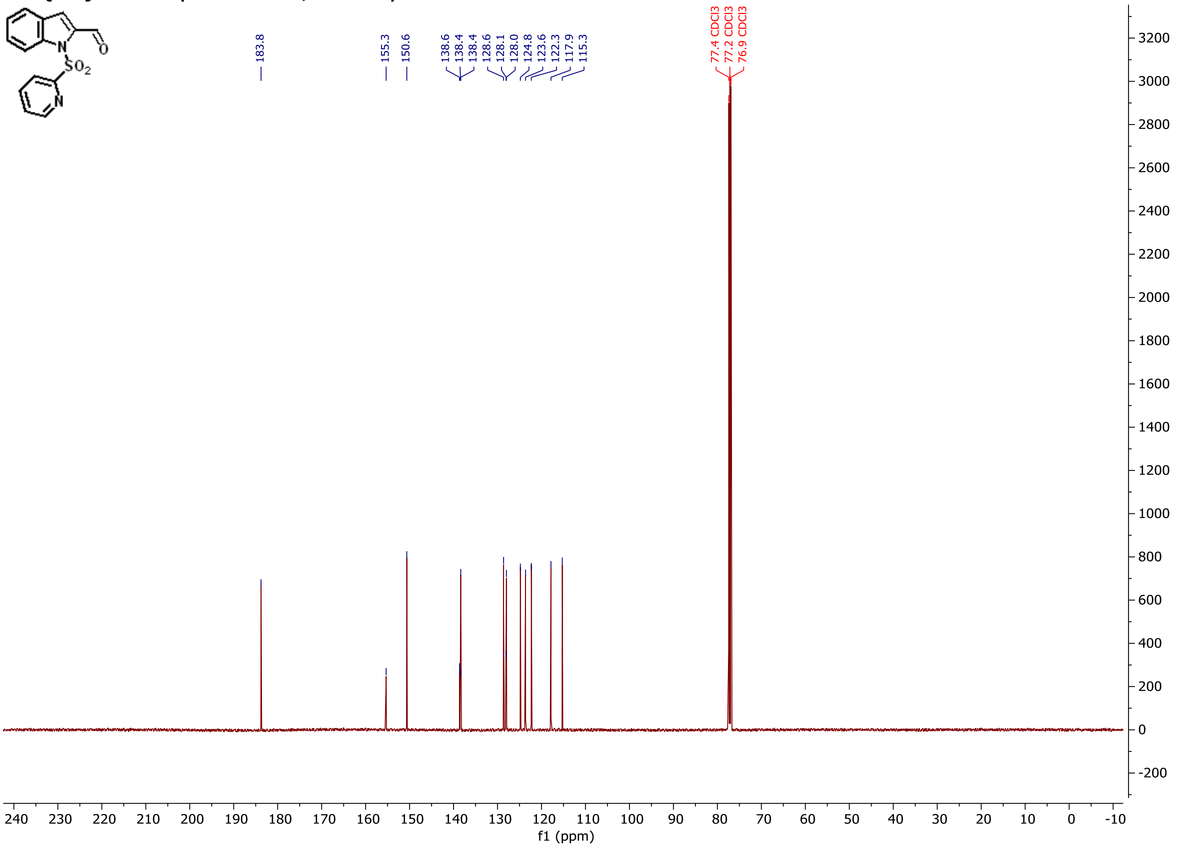

3-methyl-1-((4-nitrophenyl)sulfonyl)-1H-indole-2-carbaldehyde (1h)

^{1}H NMR (500 MHz, $CDCl_3$)

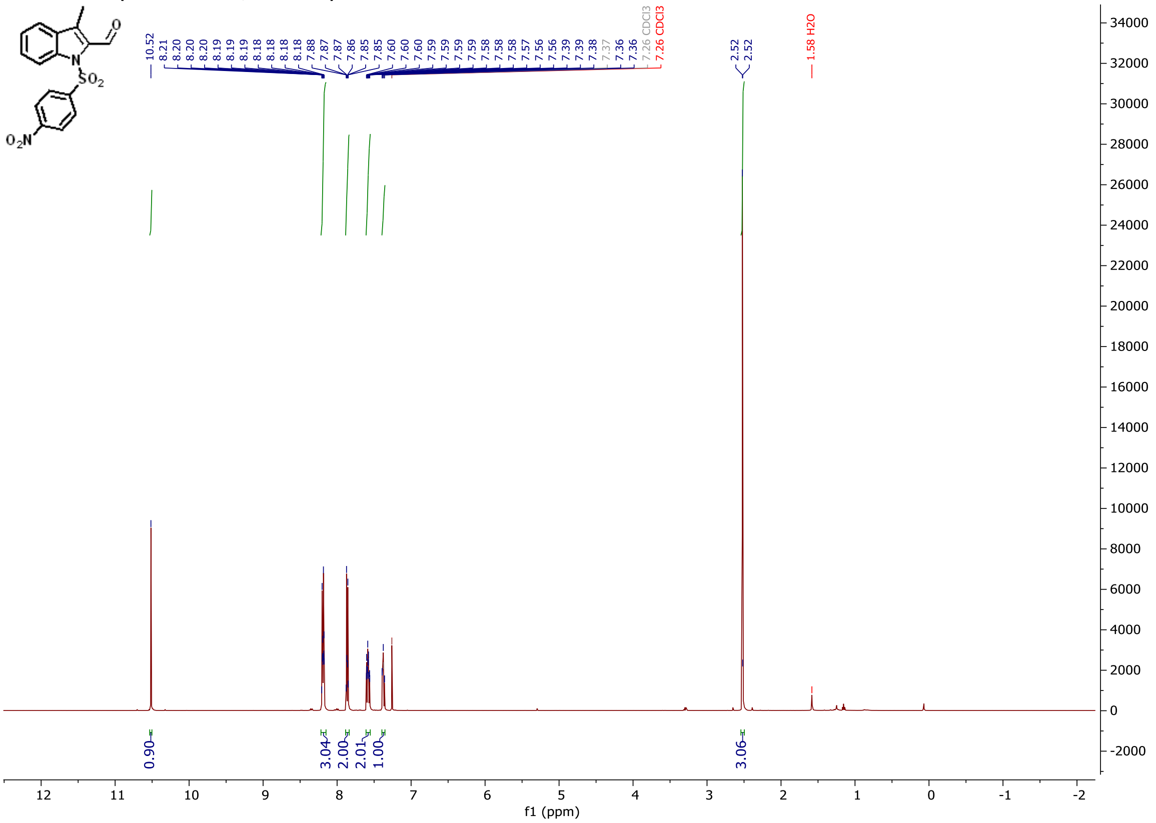

^{13}C{^{1}H} NMR (126 MHz, $CDCl_3$)

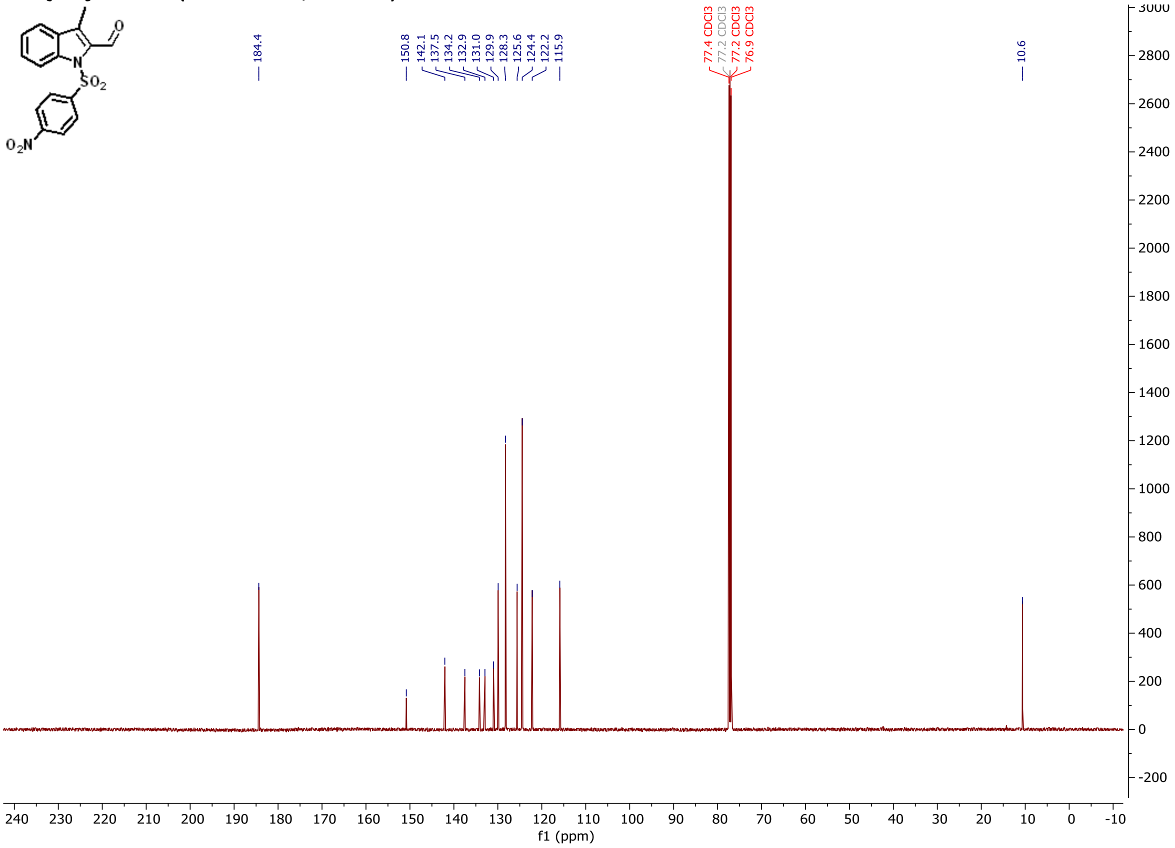

5-methyl-1-((4-nitrophenyl)sulfonyl)-1H-indole-2-carbaldehyde (1i)

^{1}H NMR (400 MHz, $CDCl_3$)

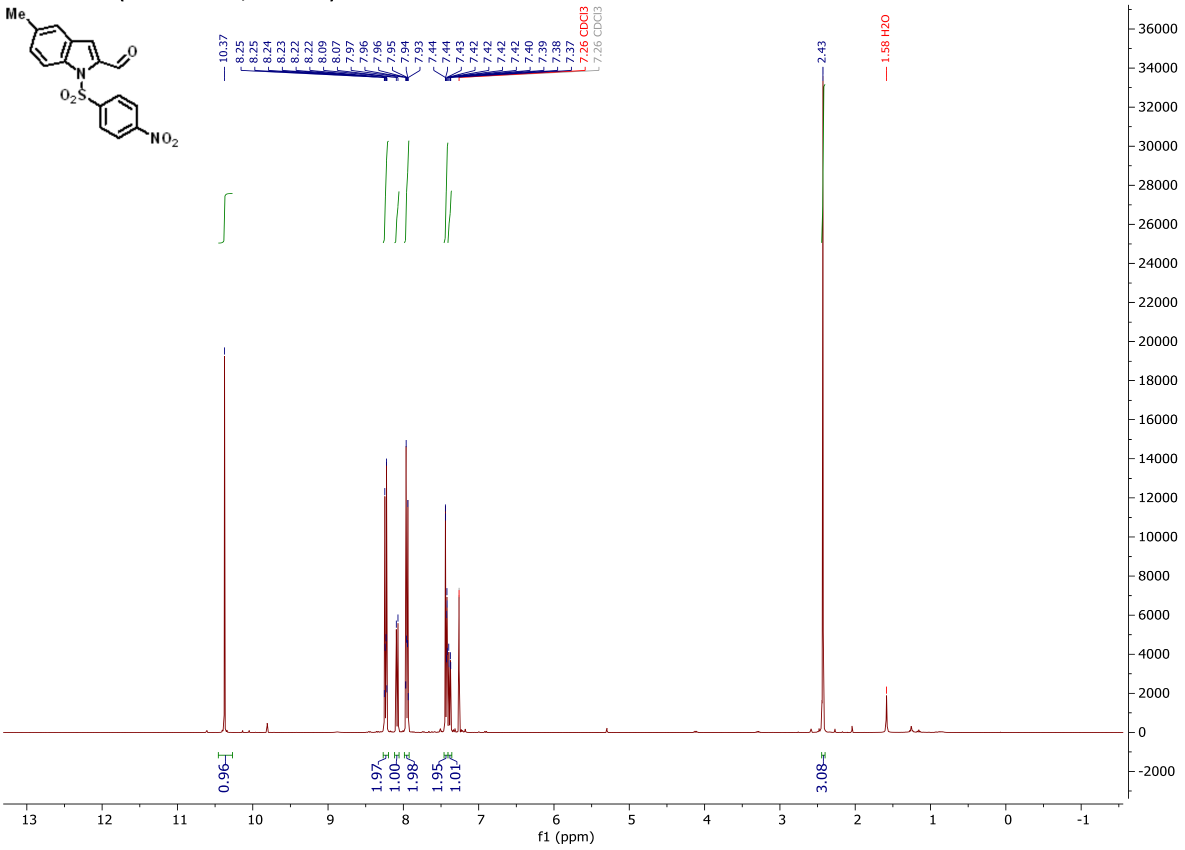

^{13}C{^{1}H} NMR (101 MHz, $CDCl_3$)

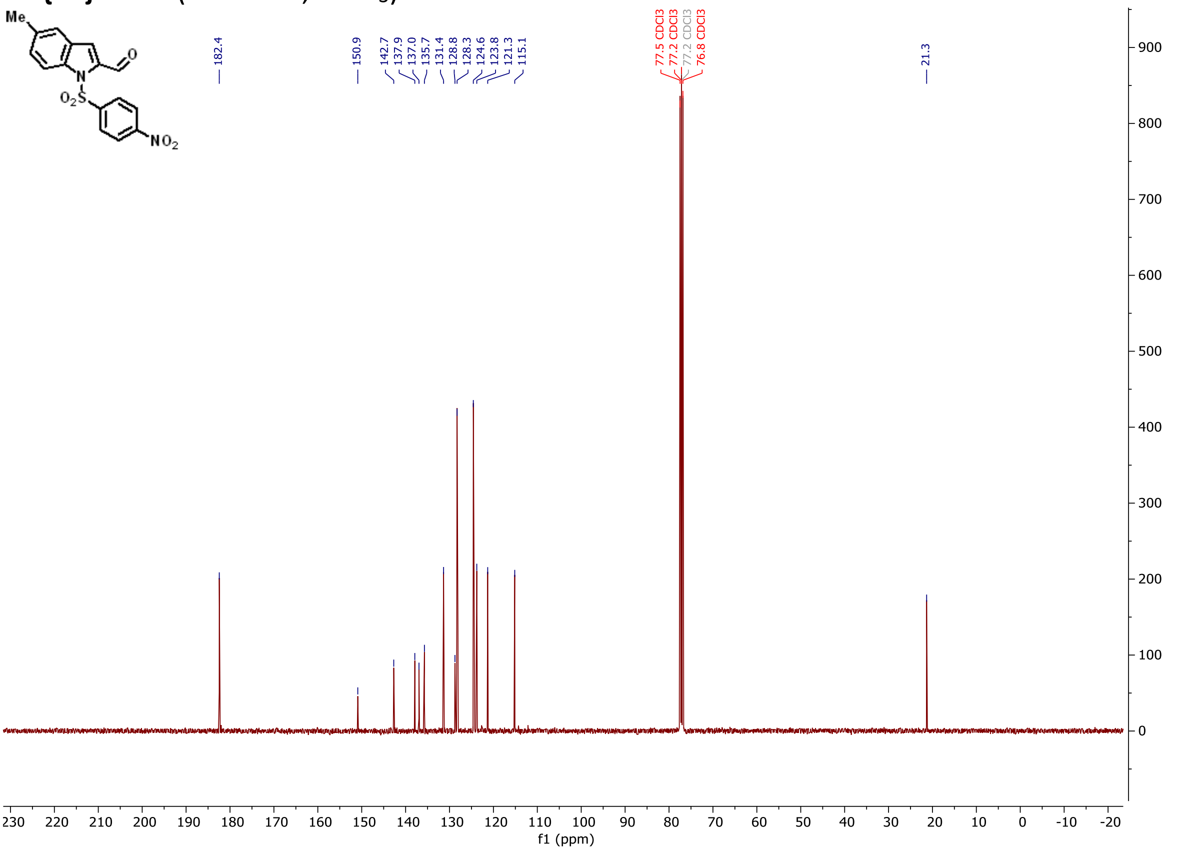

5-chloro-1-((4-nitropenyl)sulfonyl)-1H-indole-2-carbaldehyde (1j)

^{1}**H NMR** (400 MHz, $CDCl_3$)

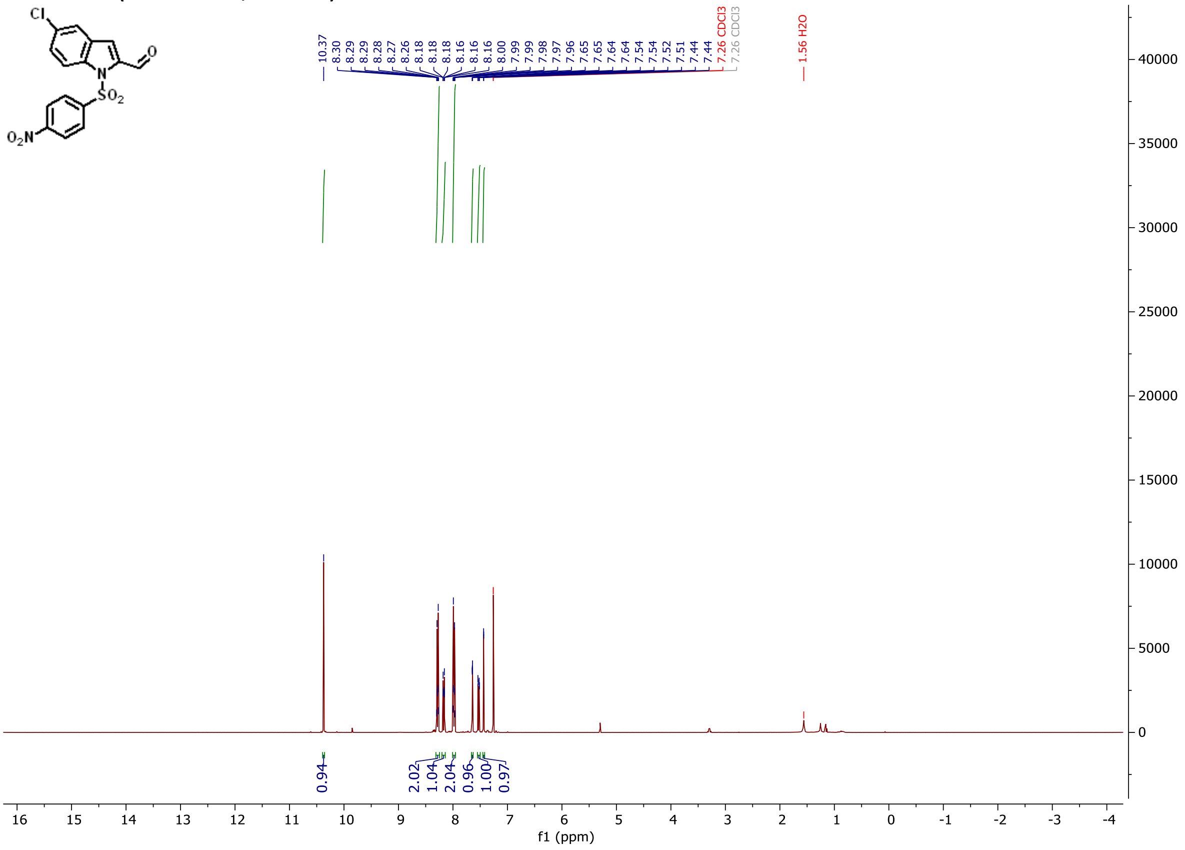

^{13}**C{^{1}H} NMR** (101 MHz, $CDCl_3$)

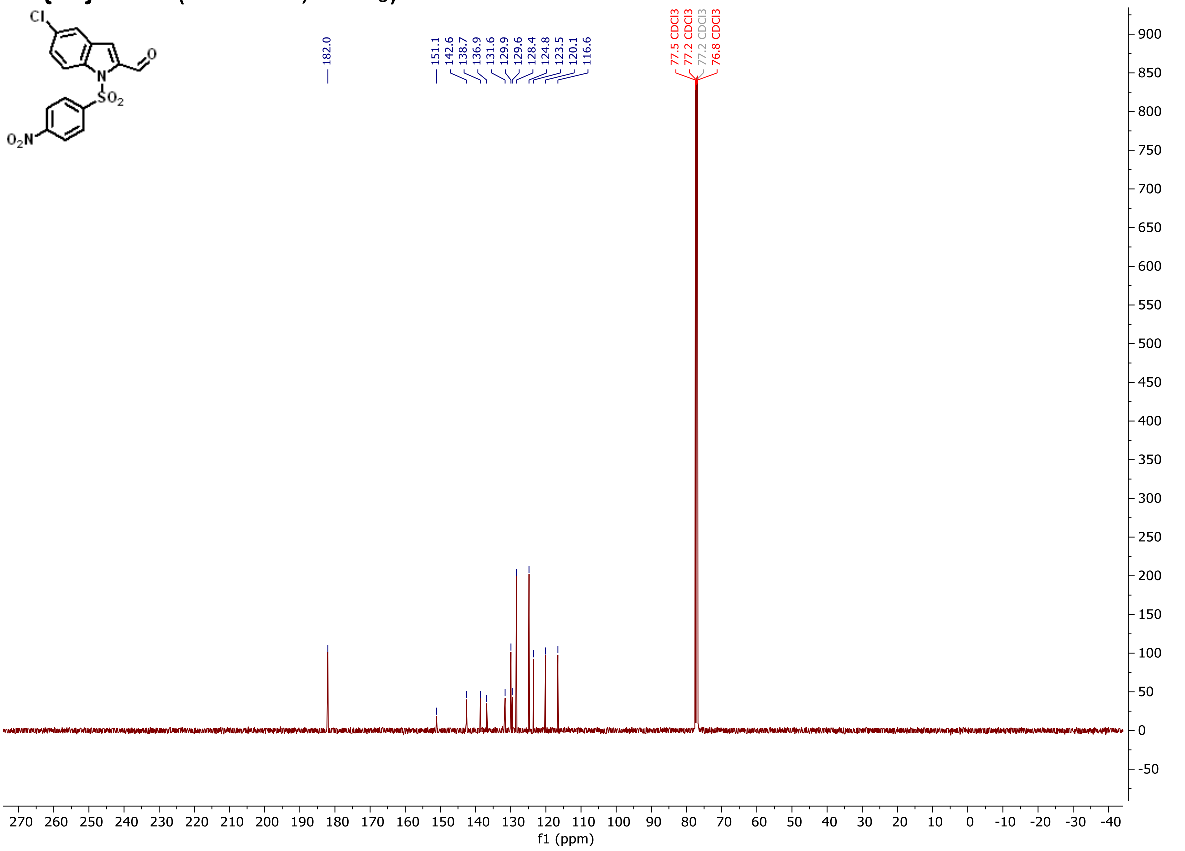

5-fluoro-1-((4-nitrophenyl)sulfonyl)-1H-indole-2-carbaldehyde (1k)

^{1}H NMR (400 MHz, $CDCl_3$)

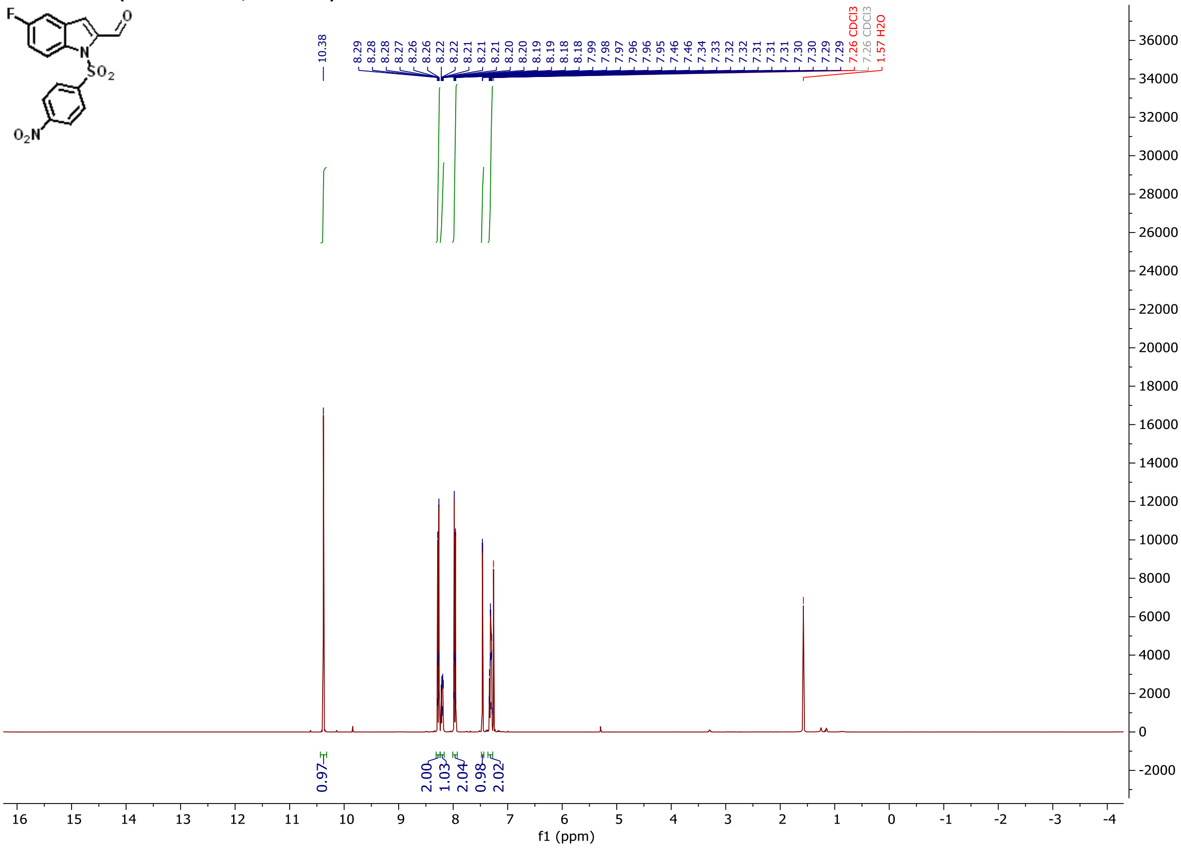

^{13}C{^{1}H} NMR (101 MHz, $CDCl_3$)

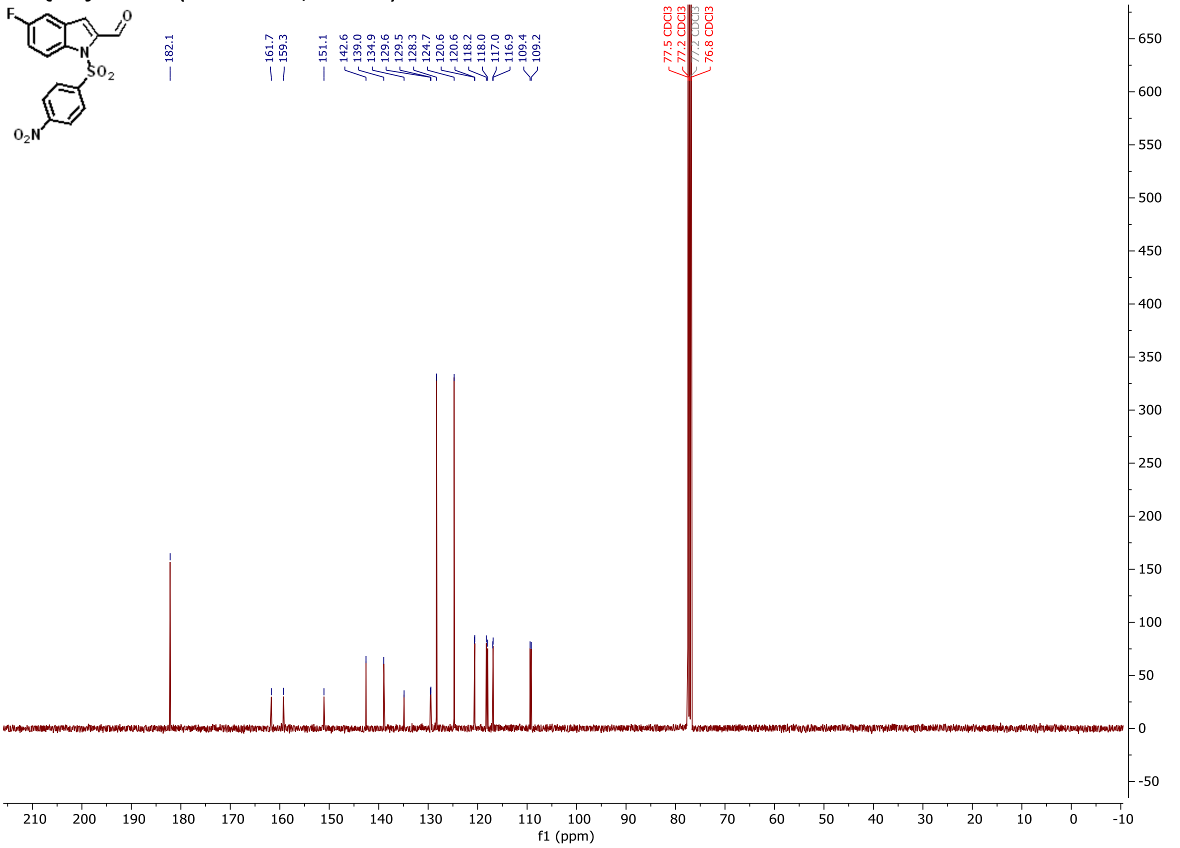

^{19}F NMR (471 MHz, $CDCl_3$)

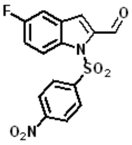

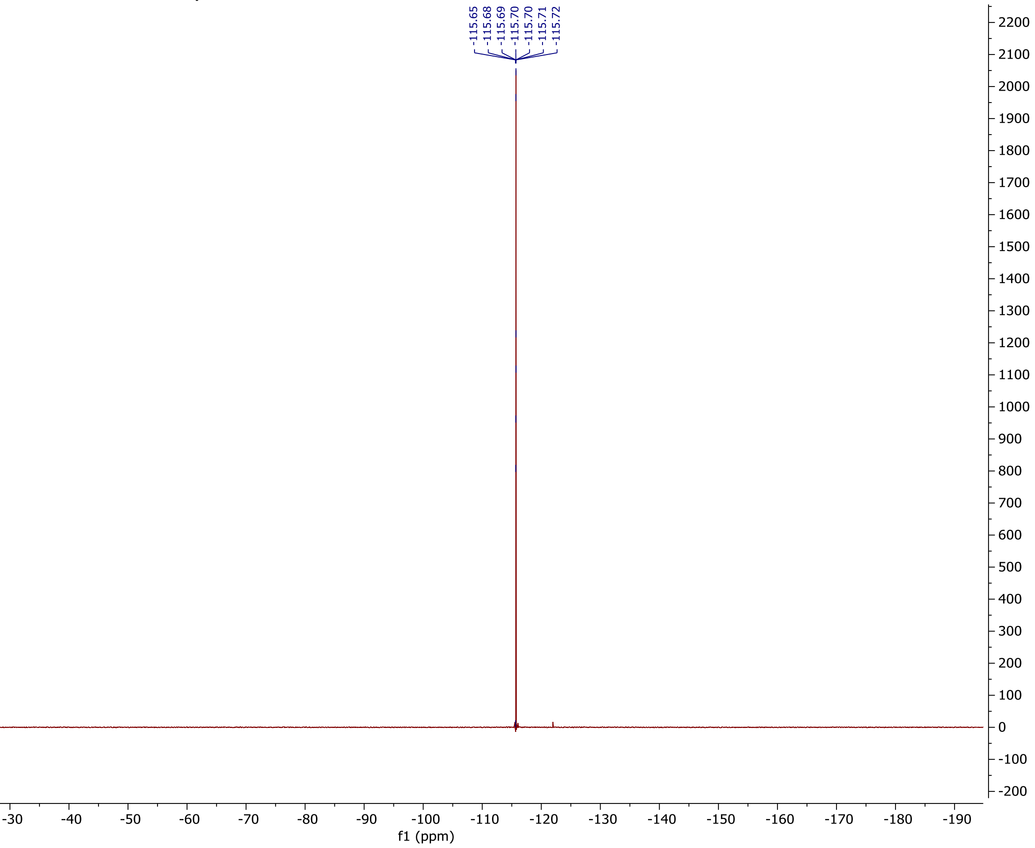

6-methoxy-1-((4-nitrophenyl)sulfonyl)-1H-indole-2-carbaldehyde (1l)

^{1}H NMR (400 MHz, $CDCl_3$)

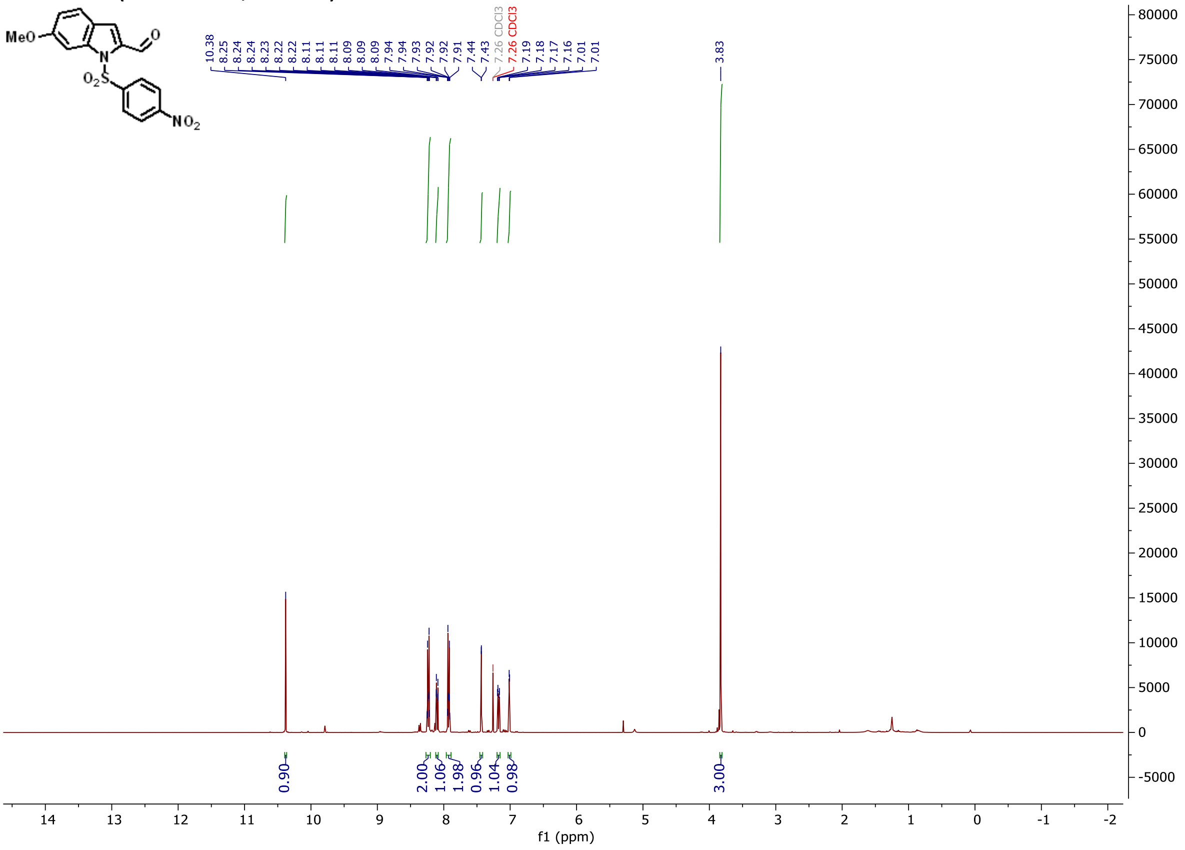

$^{13}C\{^{1}H\}$ NMR (101 MHz, $CDCl_3$)

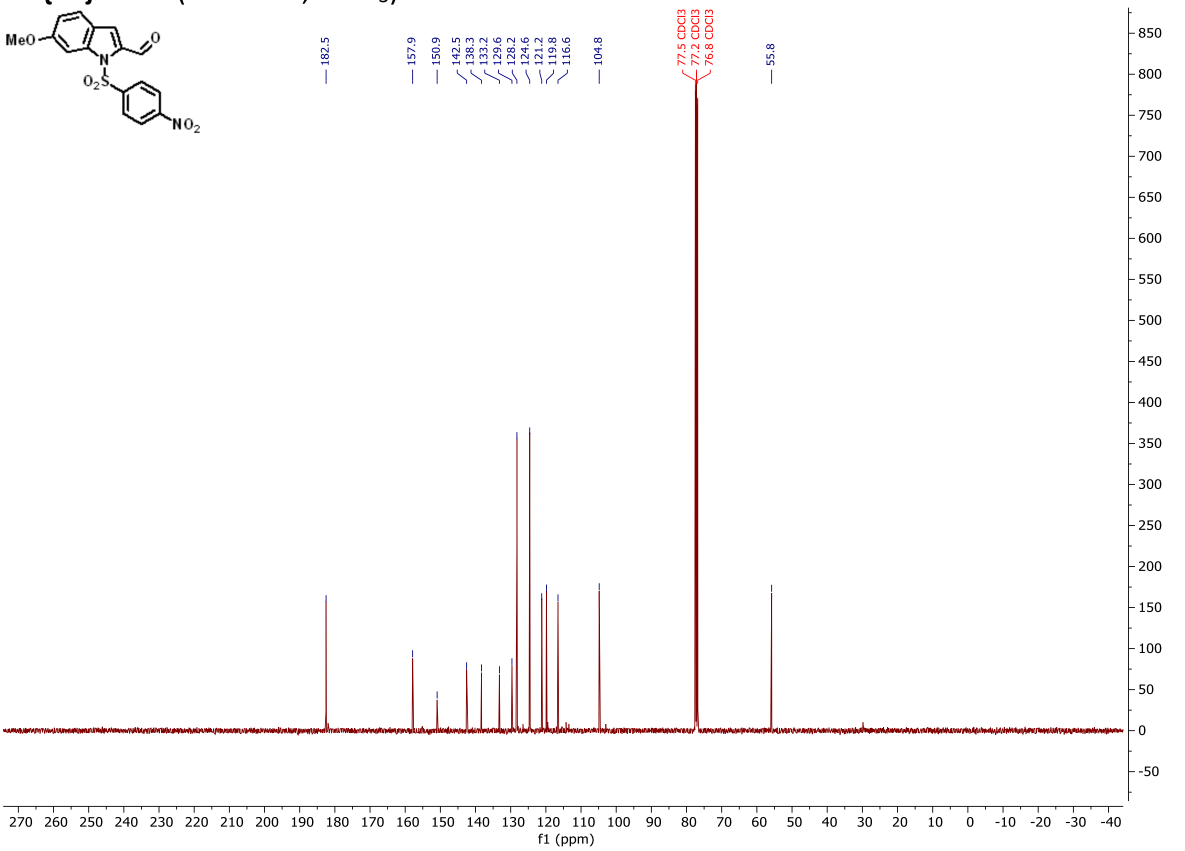

6-bromo-1-((4-nitrophenyl)sulfonyl)-1H-indole-2-carbaldehyde (1m)

^{1}H NMR (500 MHz, $CDCl_3$)

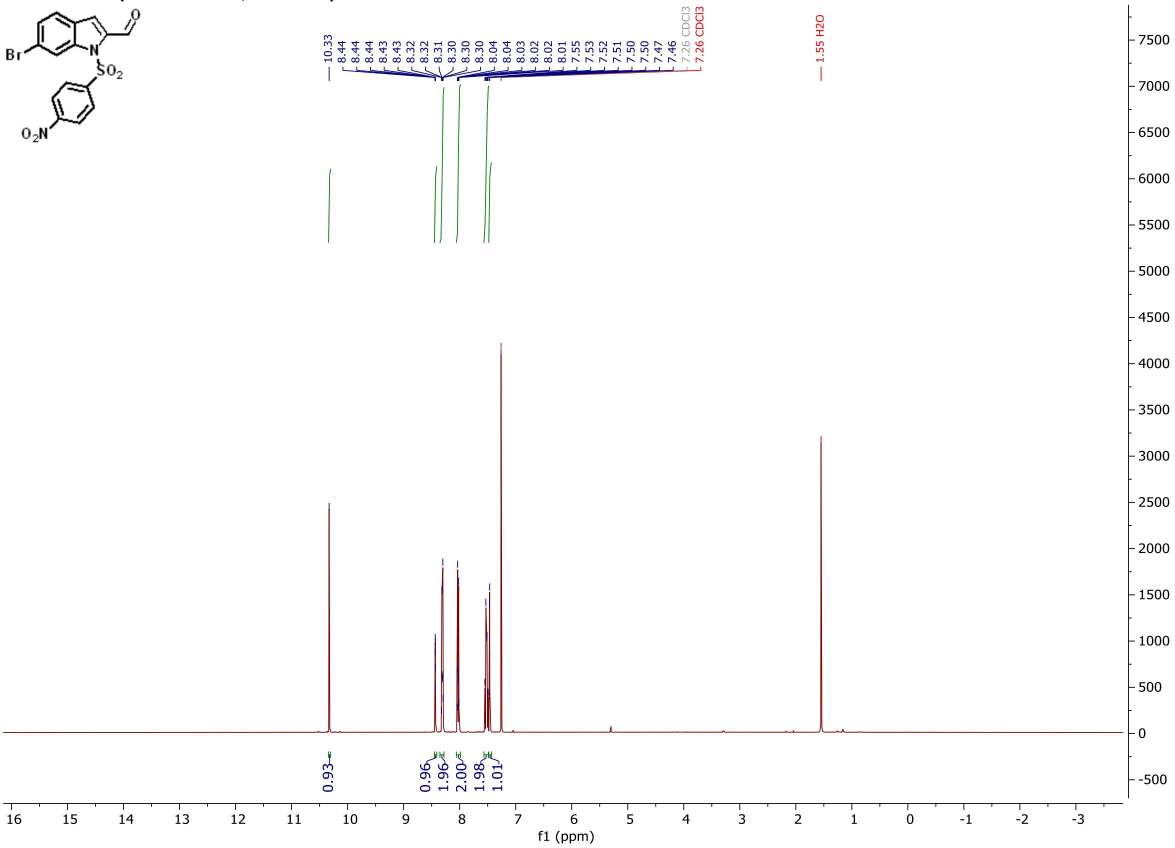

^{13}C{^{1}H} NMR (126 MHz, $CDCl_3$)

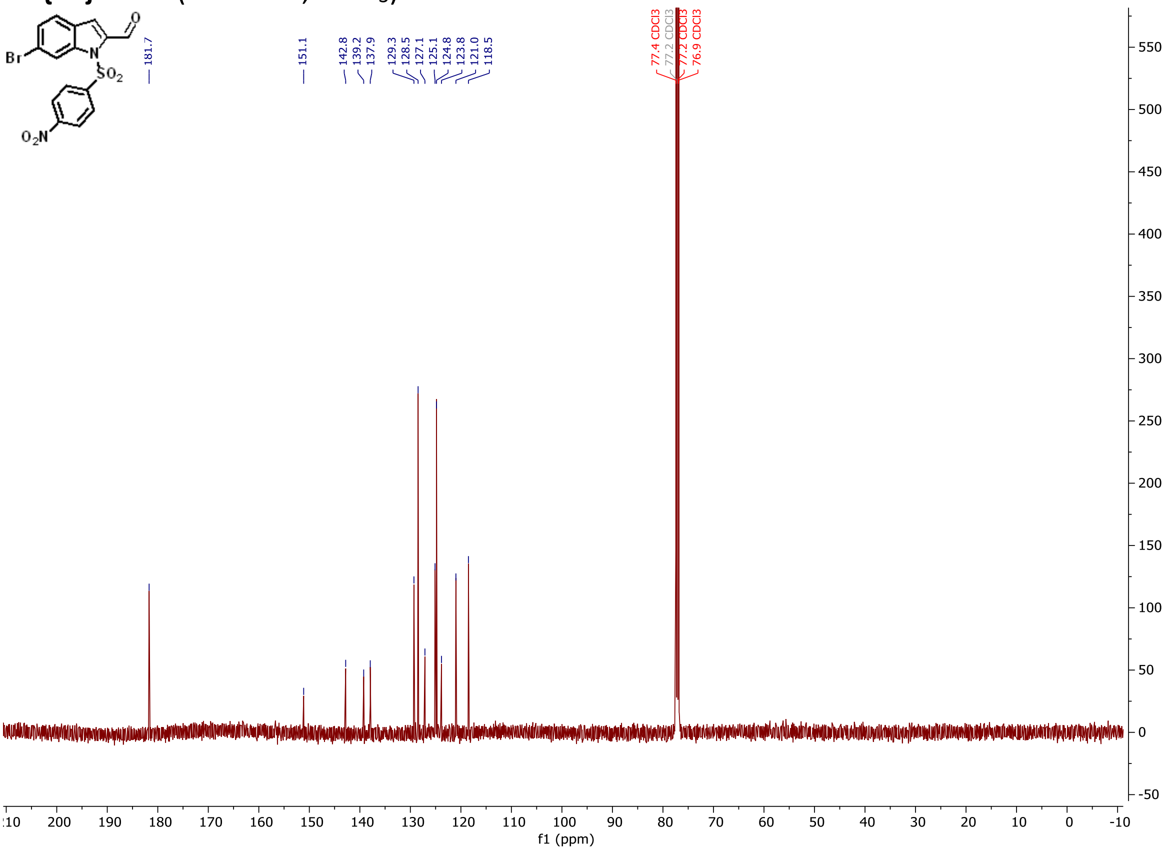

6-chloro-1-((4-nitrophenyl)sulfonyl)-1H-indole-2-carbaldehyde (1n)

^{1}H NMR (400 MHz, $CDCl_3$)

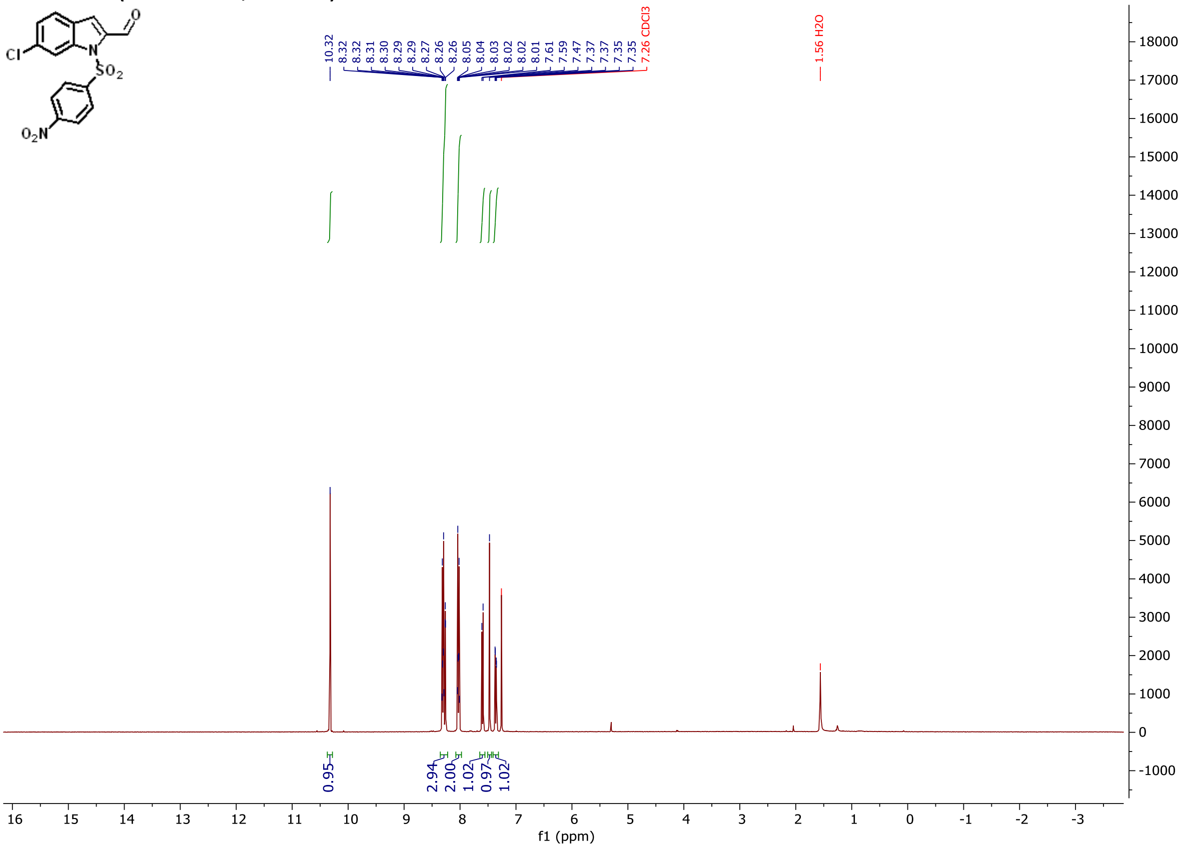

^{13}C{^{1}H} NMR (101 MHz, $CDCl_3$)

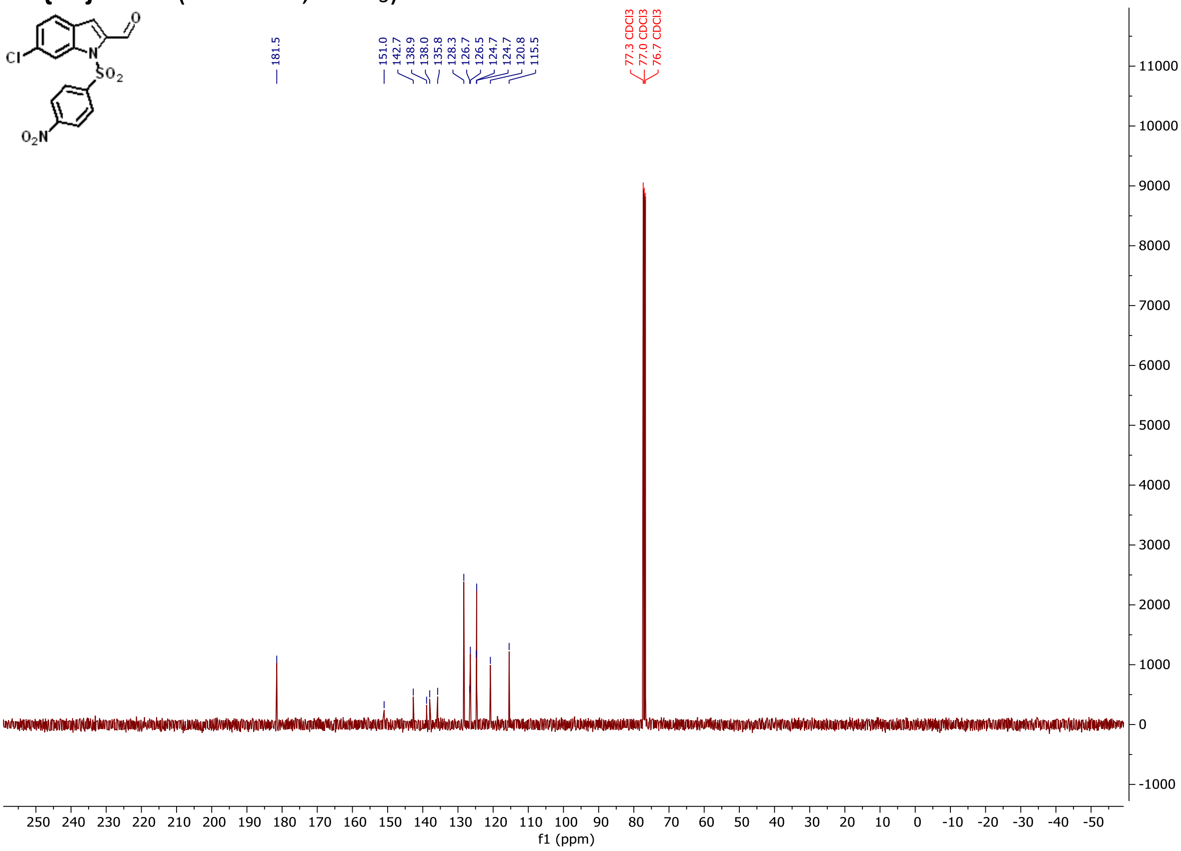

(1H-indol-2-yl)(4-nitrophenyl)methanone (2a)

^{1}H NMR (400 MHz, $CDCl_3$)

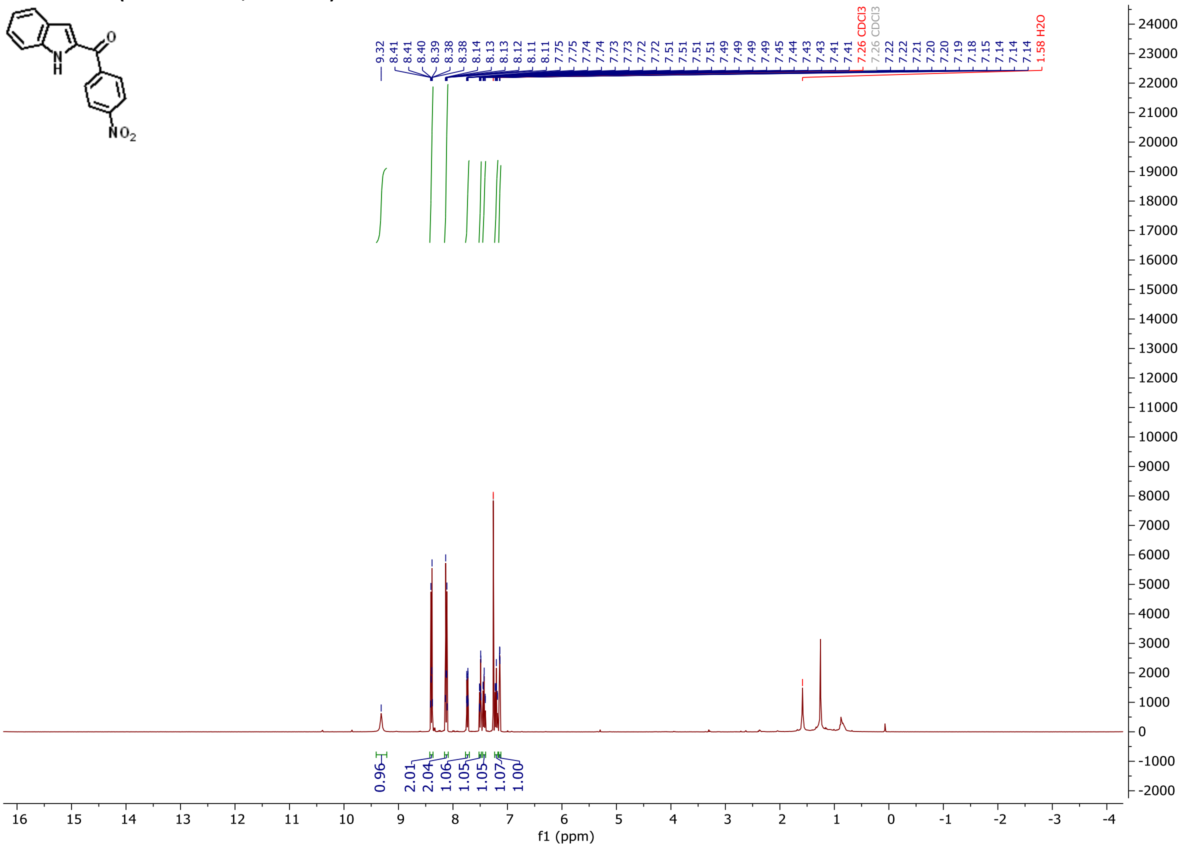

^{13}C{^{1}H} NMR (101 MHz, $CDCl_3$)

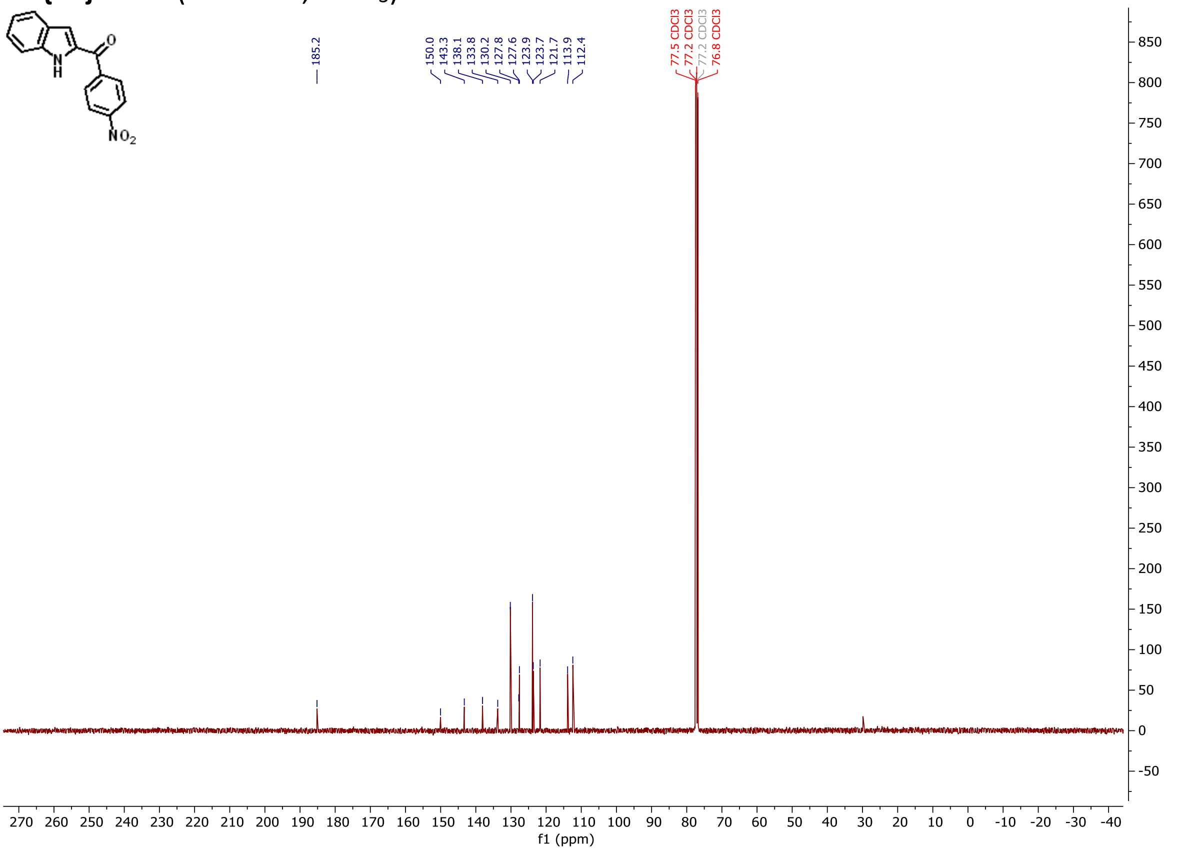

(1H-indol-2-yl)(4-nitro-3-(trifluoromethyl)phenyl)methanone (2b)

^{1}H NMR (500 MHz, $CDCl_3$)

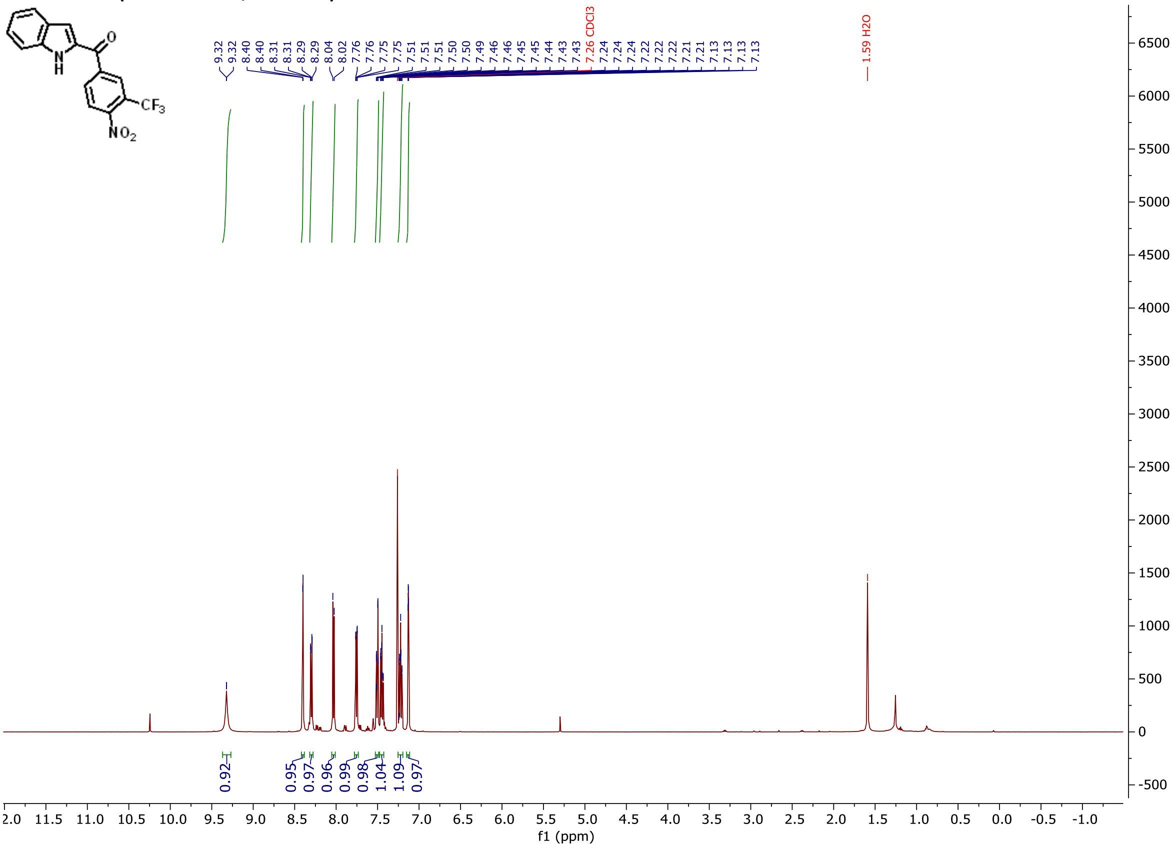

^{13}C{^{1}H} NMR (126 MHz, $CDCl_3$)

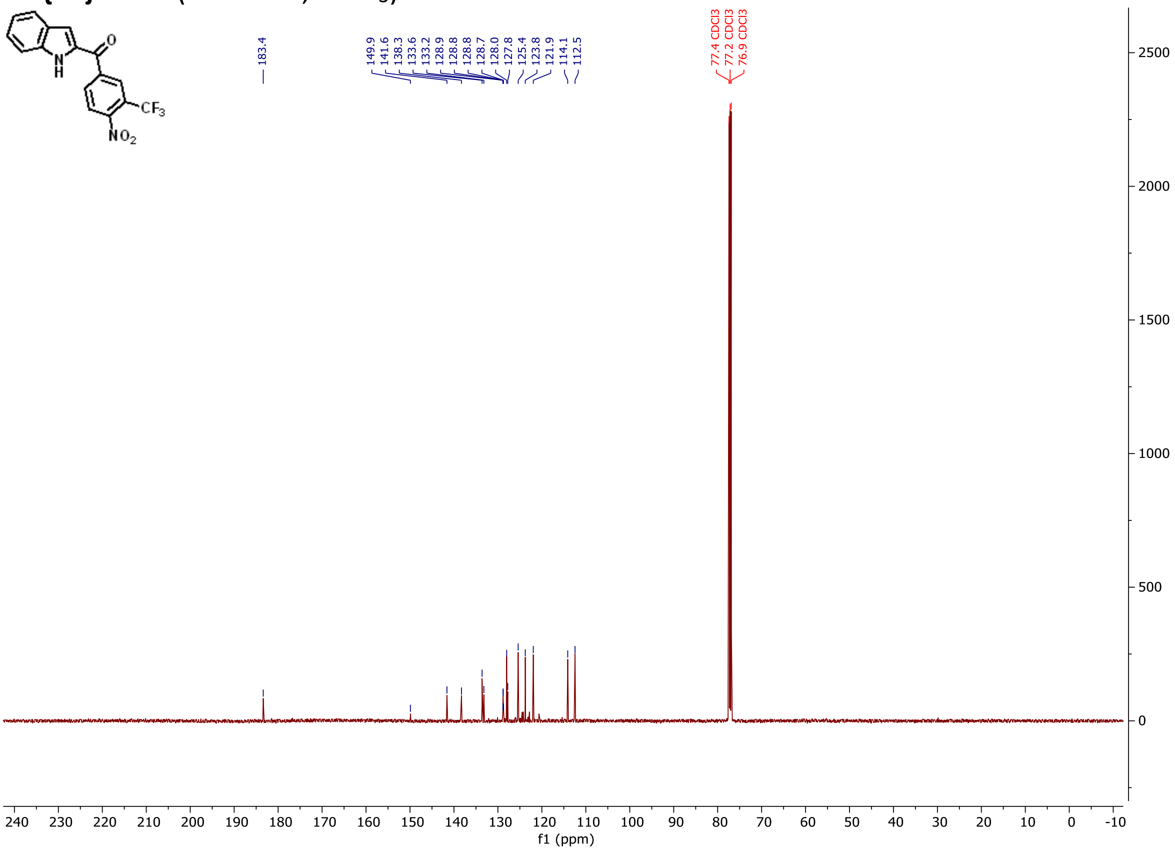

^{19}F NMR (471 MHz, $CDCl_3$)

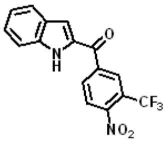

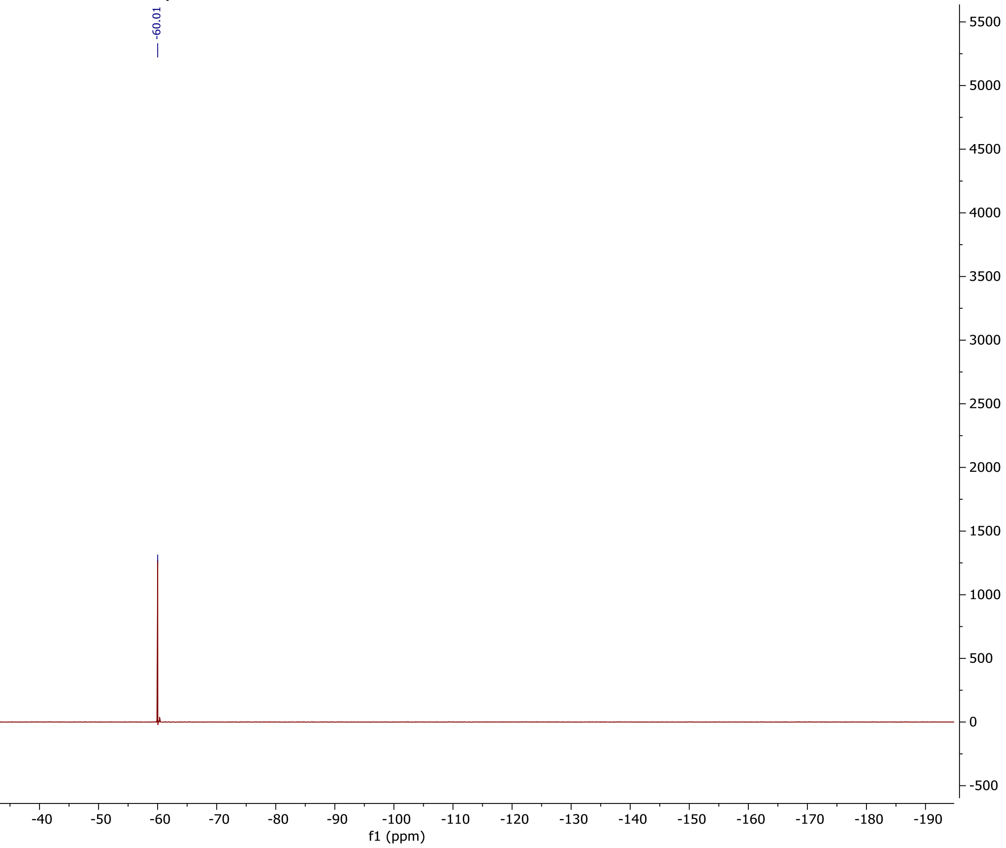

(2-fluoro-4-nitrophenyl)(1H-indol-2-yl)methanone (2d)

^{1}H NMR (400 MHz, $CDCl_3$)

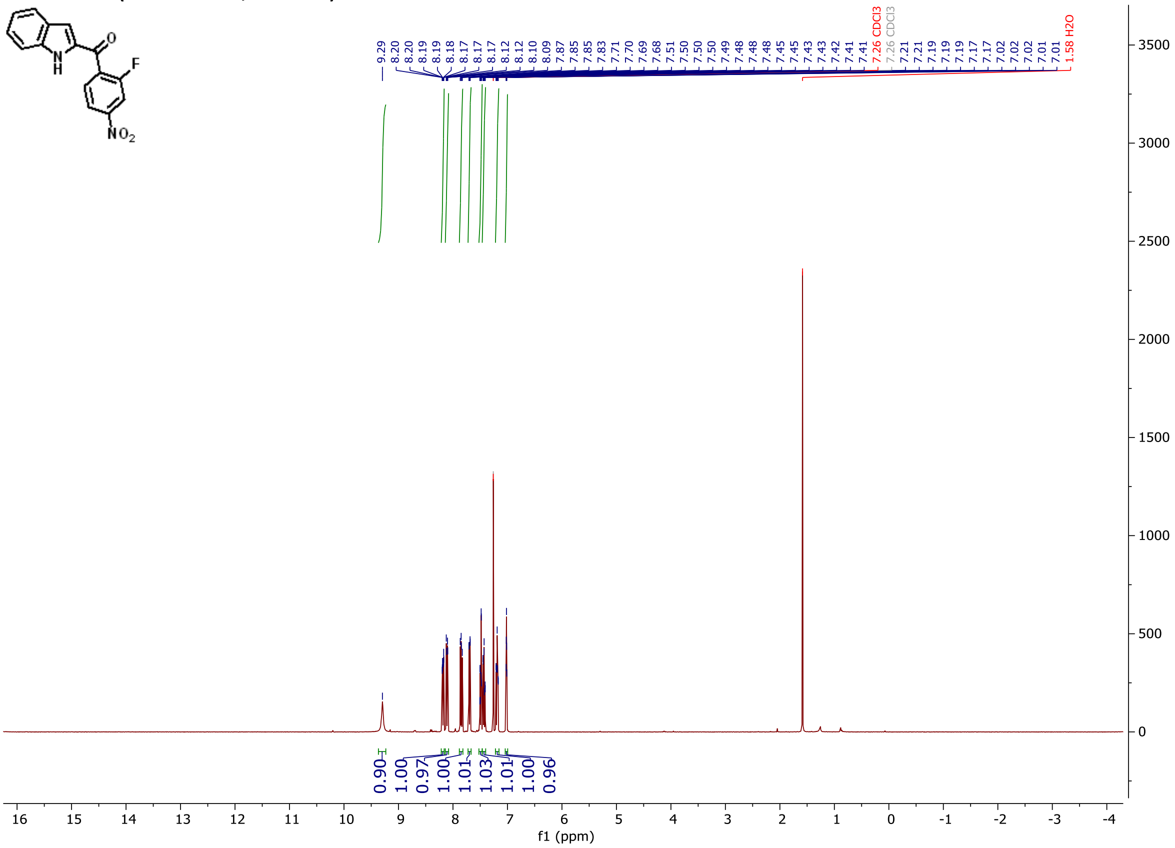

^{13}C{^{1}H} NMR (101 MHz, $CDCl_3$)

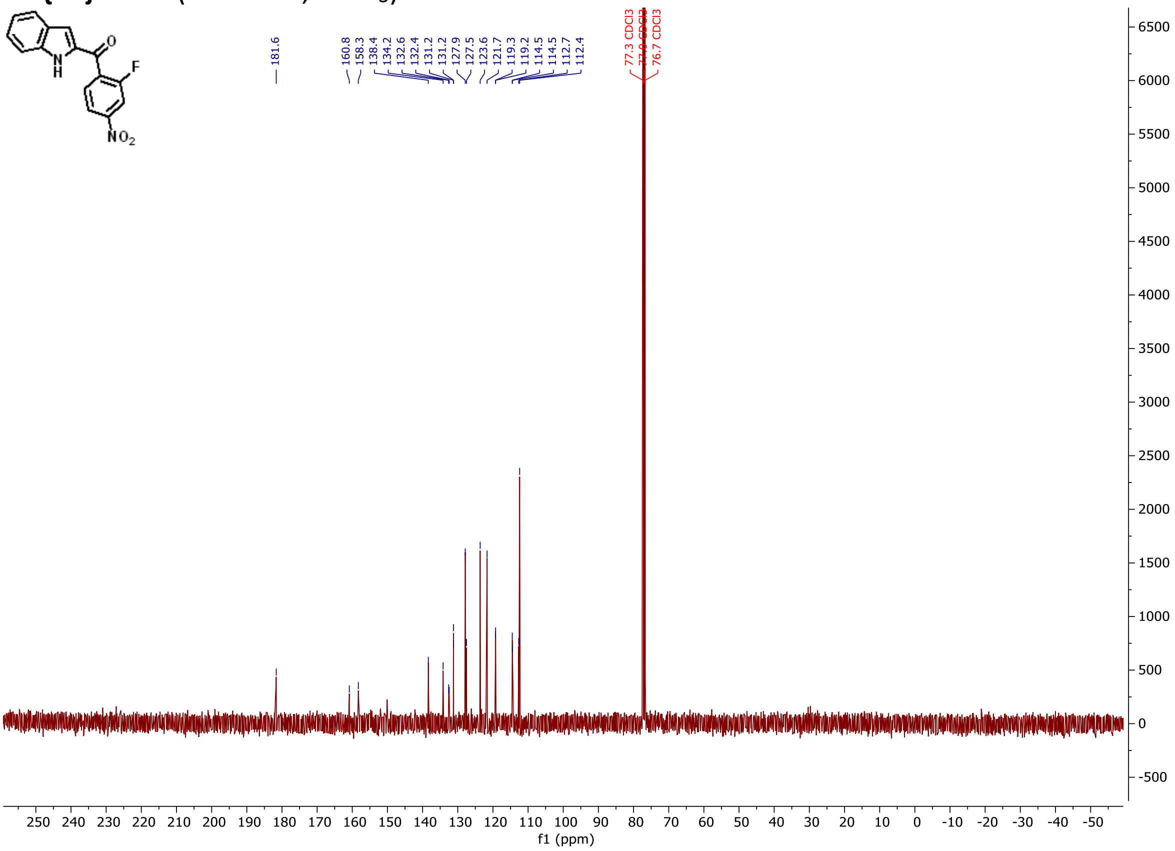

^{19}F NMR (471 MHz, $CDCl_3$)

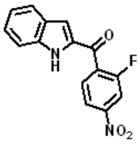

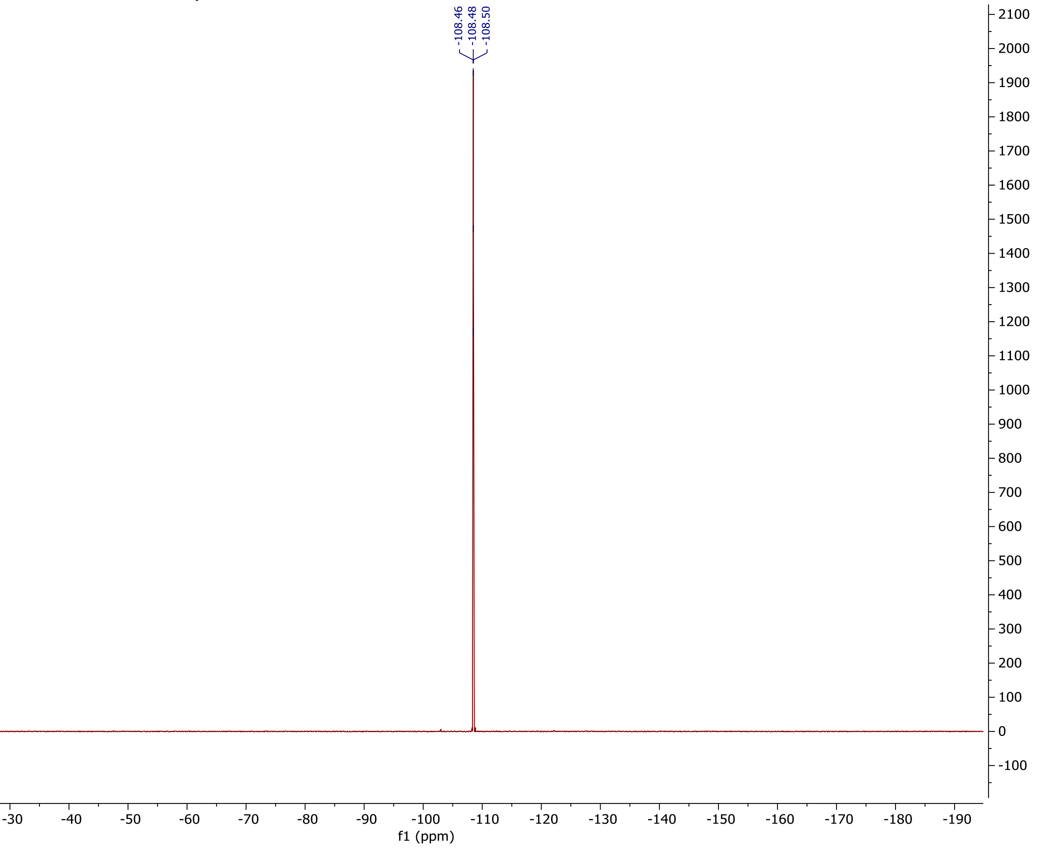

(1H-indol-2-yl)(2-methoxy-4-nitrophenyl)methanone (2c)

^{1}H NMR (500 MHz, $CDCl_3$)

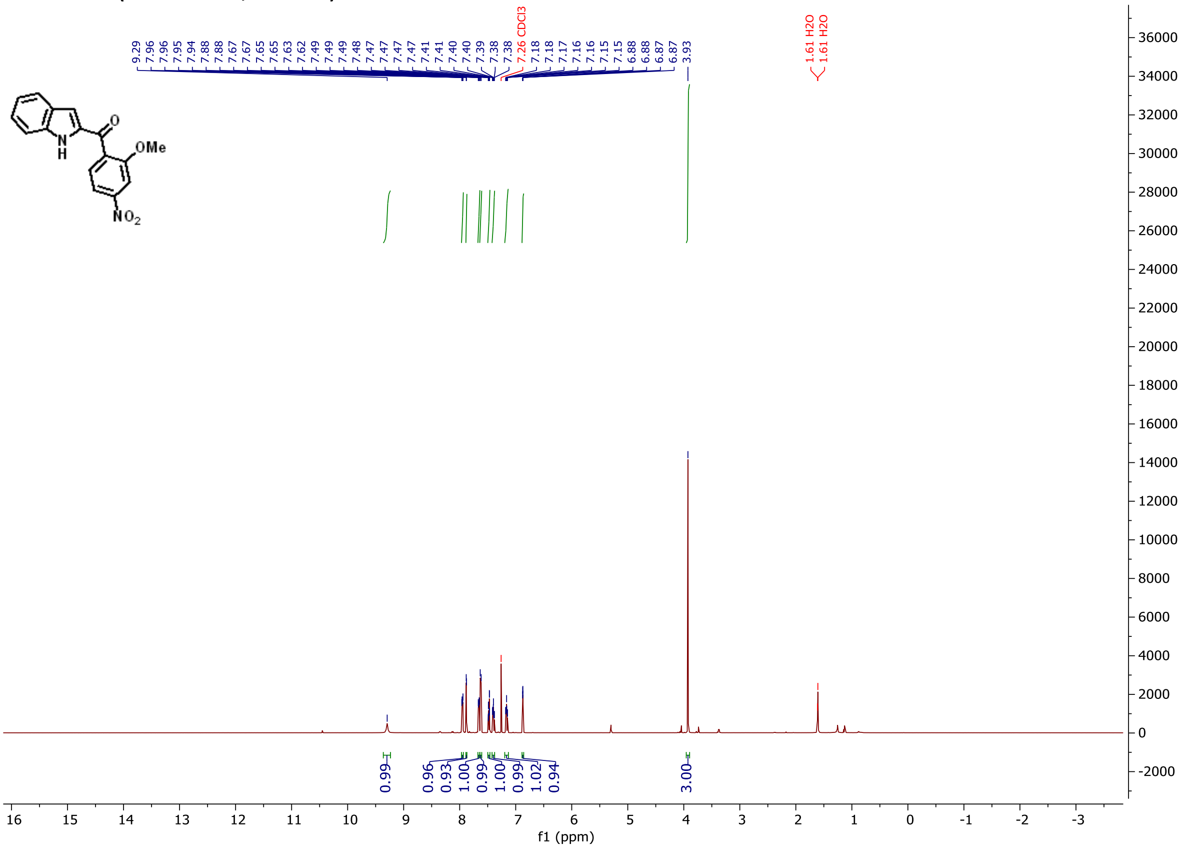

^{13}C{^{1}H} NMR (126 MHz, $CDCl_3$)

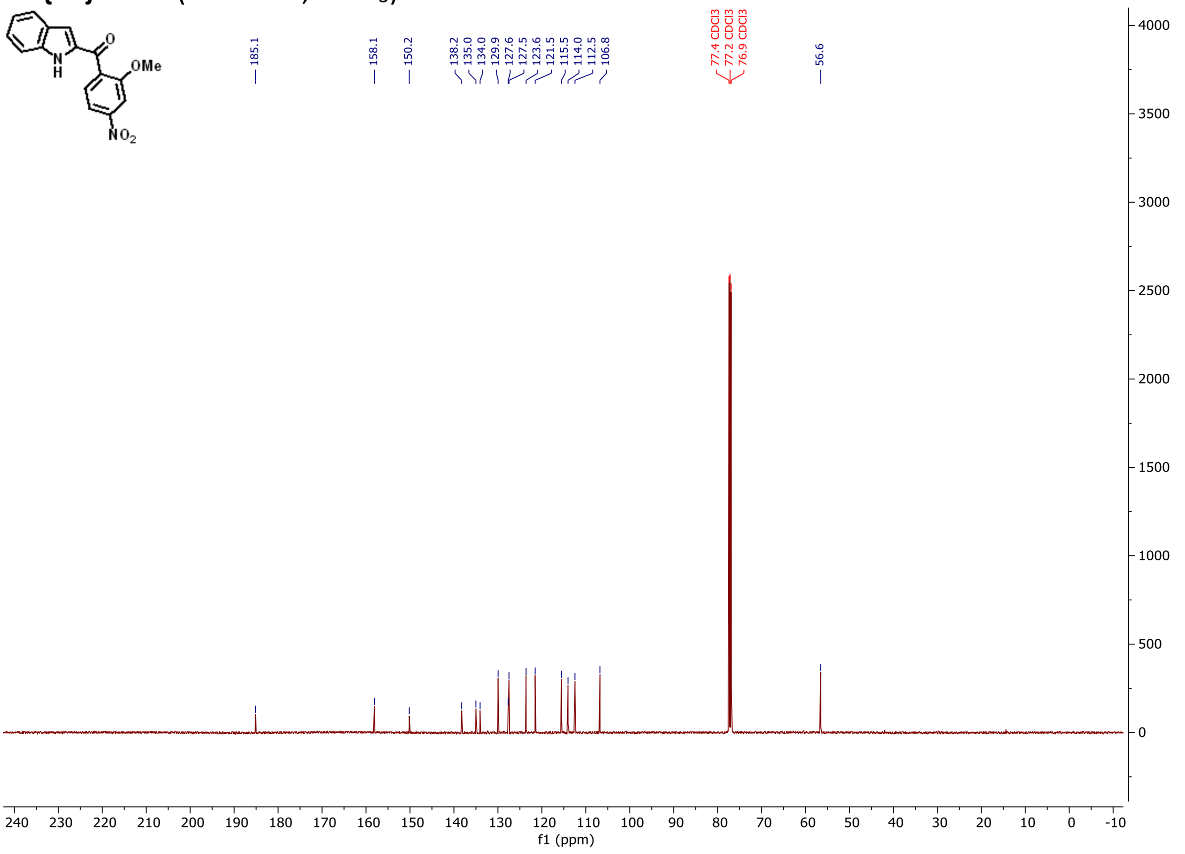

(1H-indol-2-yl)(2-nitrophenyl)methanone (2e)

^{1}H NMR (500 MHz,CDCl$_3$)

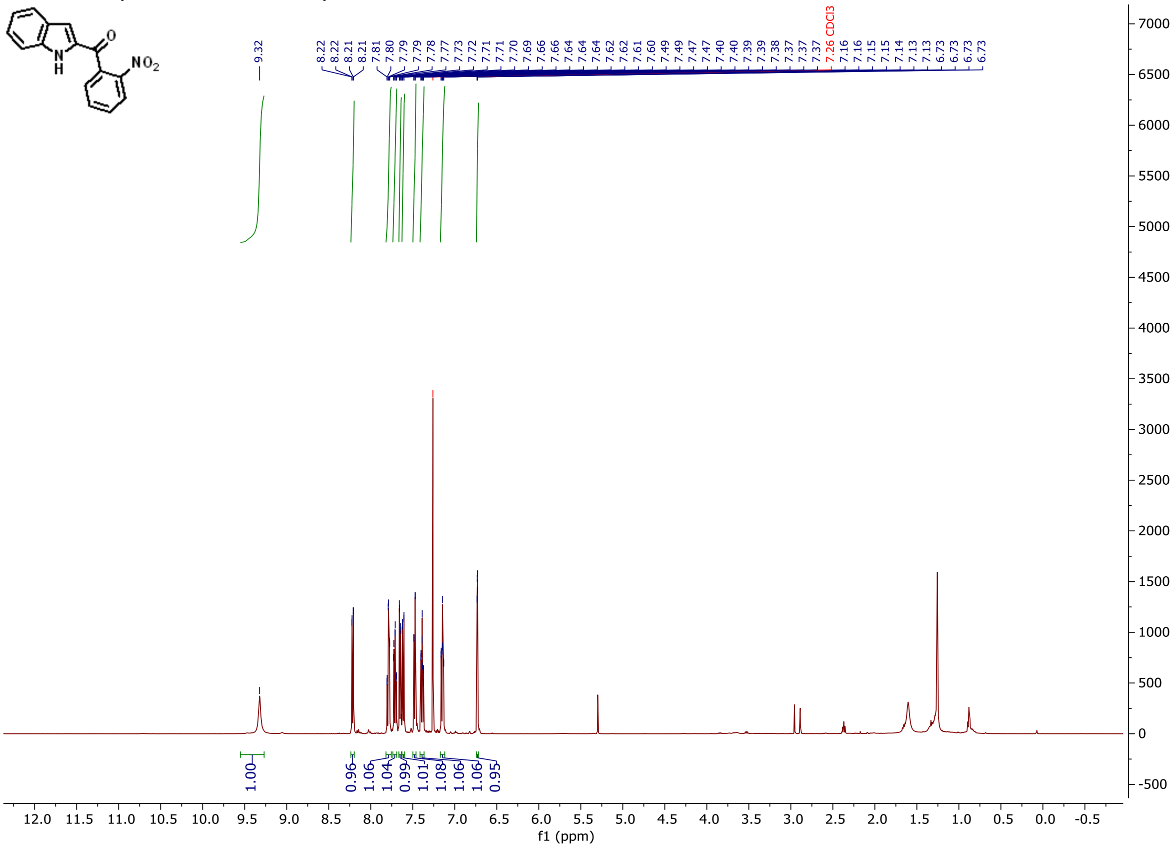

^{13}C{^{1}H} NMR (126 MHz, CDCl$_3$)

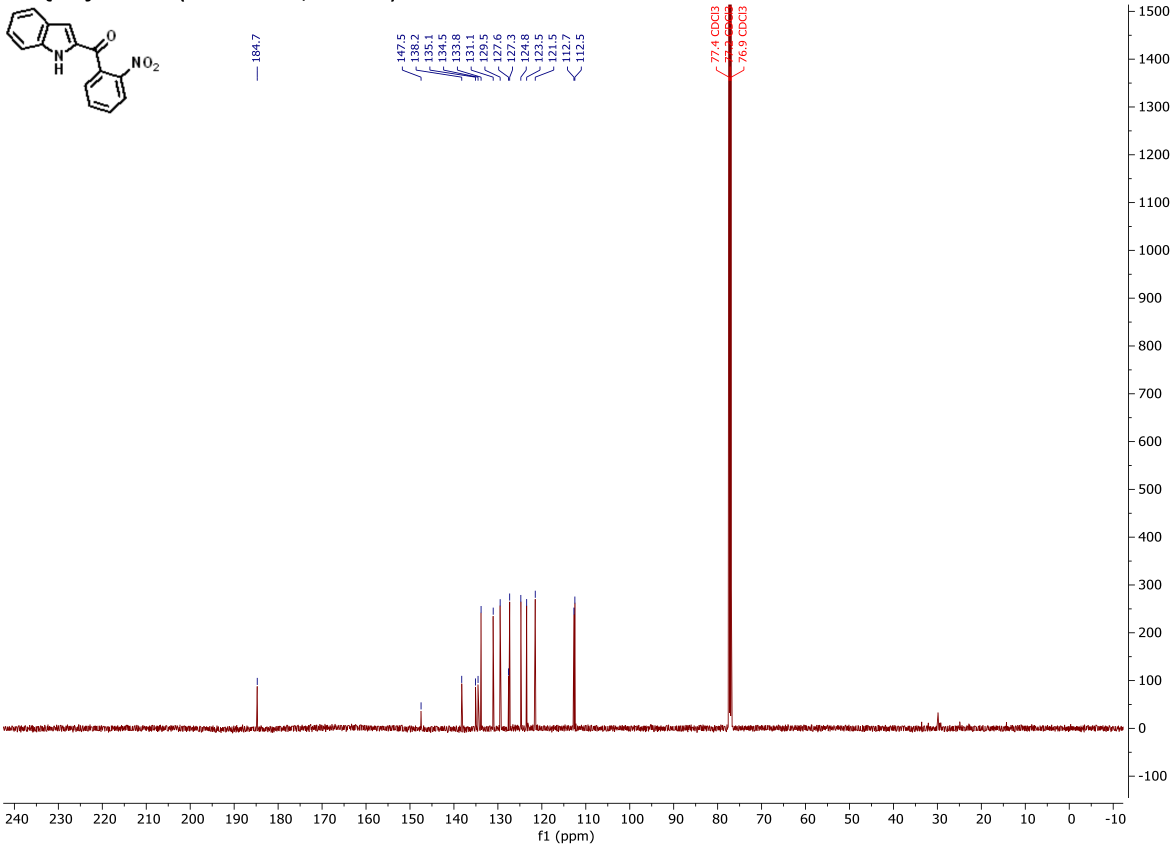

(4-bromo-2-nitrophenyl)(1H-indol-2-yl)methanone (2f)

^{1}H NMR (400 MHz, $CDCl_3$)

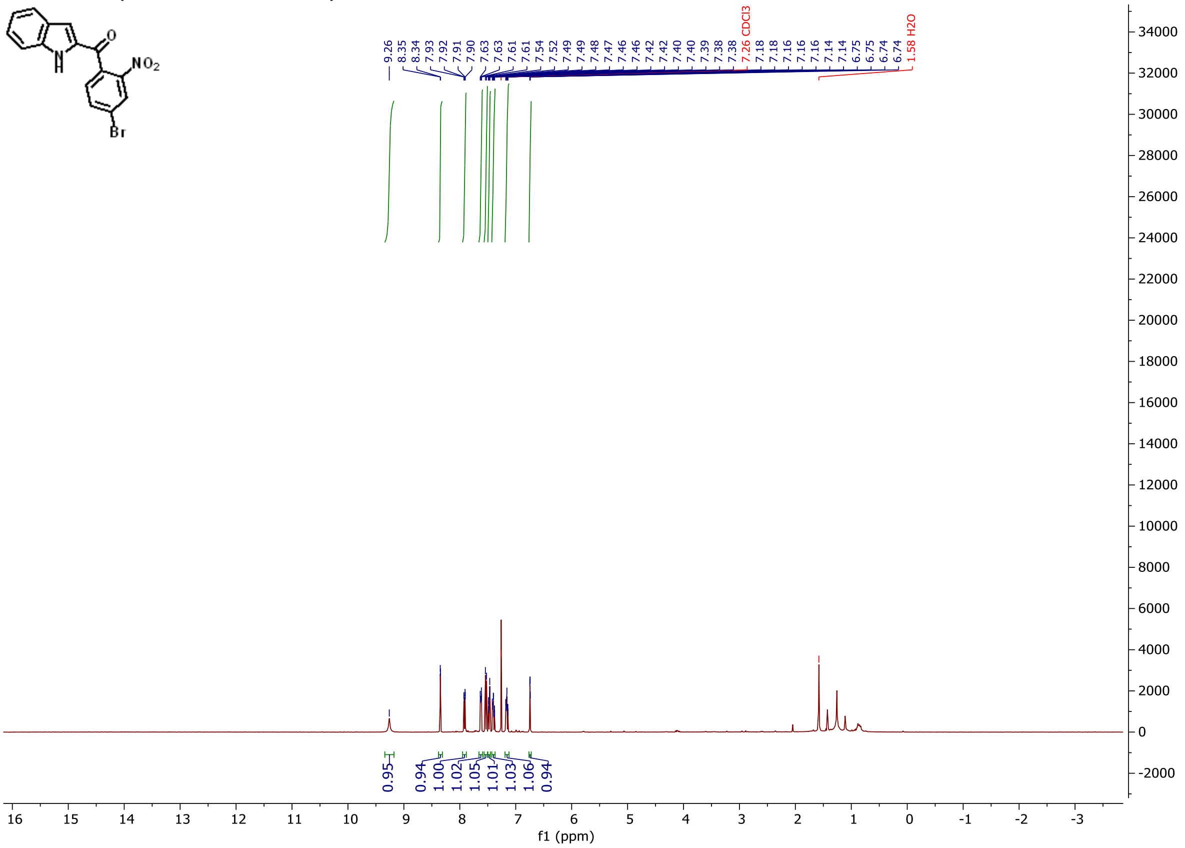

^{13}C{^{1}H} NMR (101 MHz, $CDCl_3$)

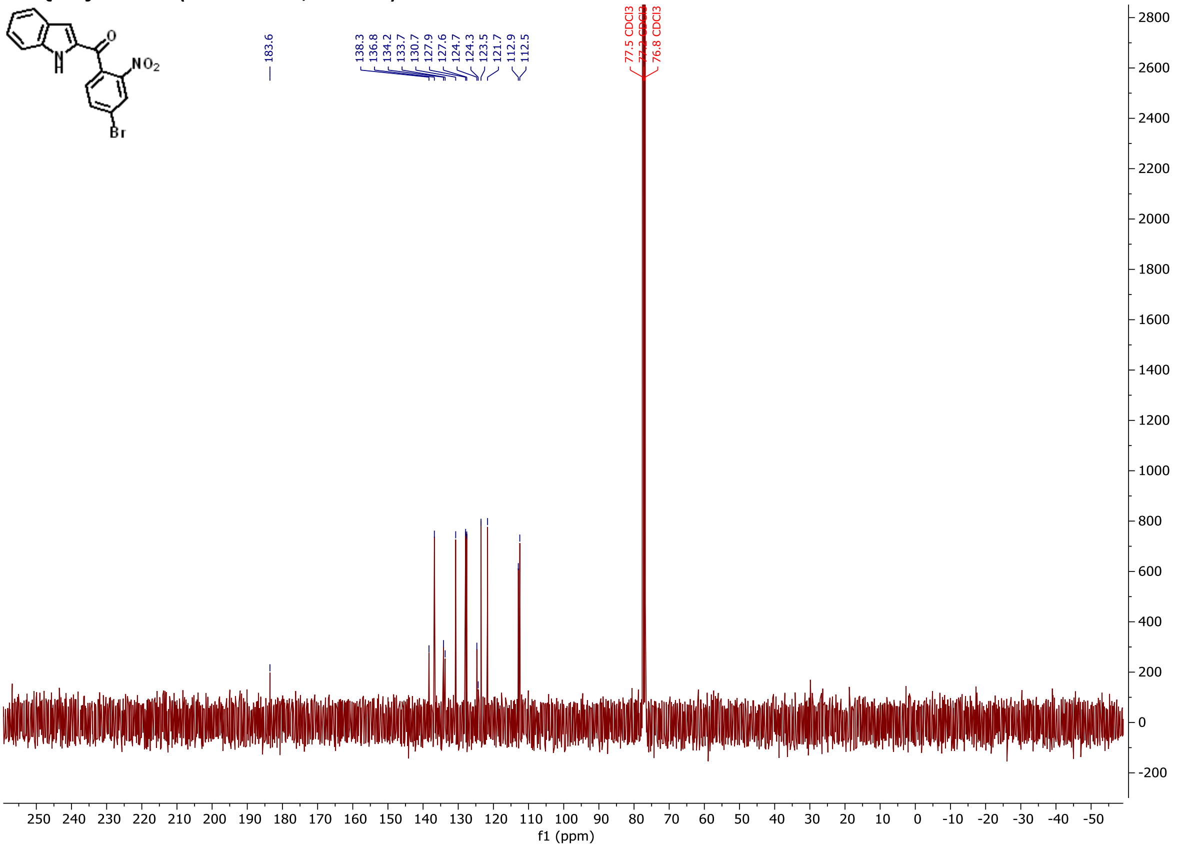

(1H-indol-2-yl)(pyridin-2-yl)methanone (2g)

^{1}H NMR (500 MHz, $CDCl_3$)

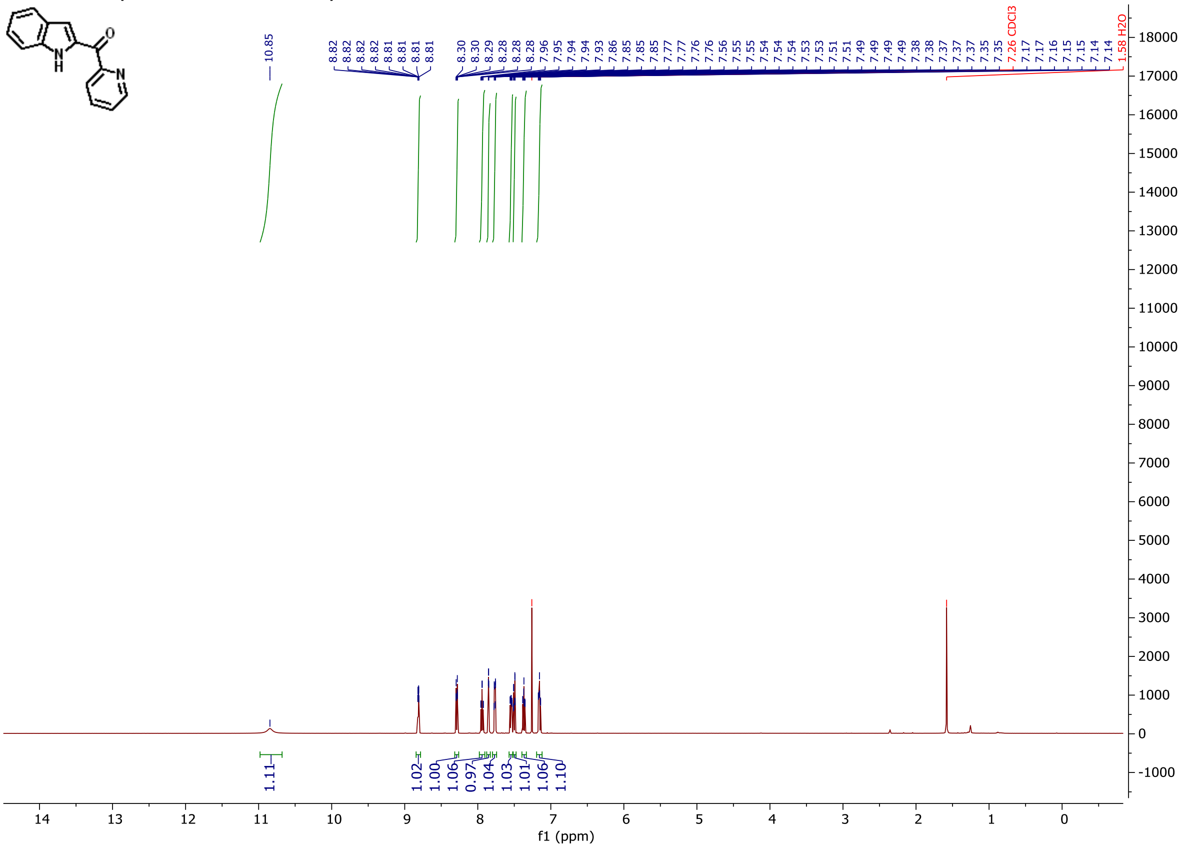

^{13}C{^{1}H} NMR (126 MHz, $CDCl_3$)

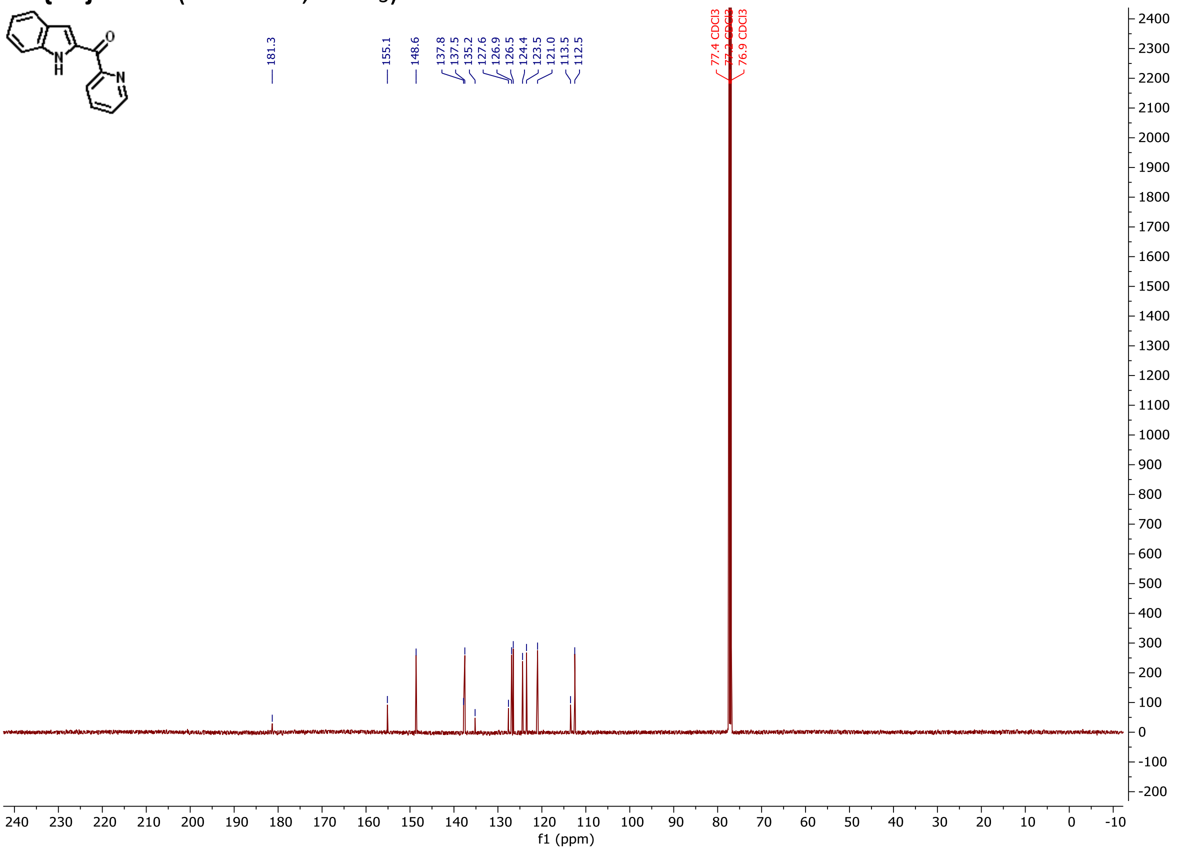

(3-methyl-1H-indol-2-yl)(4-nitrophenyl)methanone (2h)

^{1}H NMR (500 MHz, $CDCl_3$)

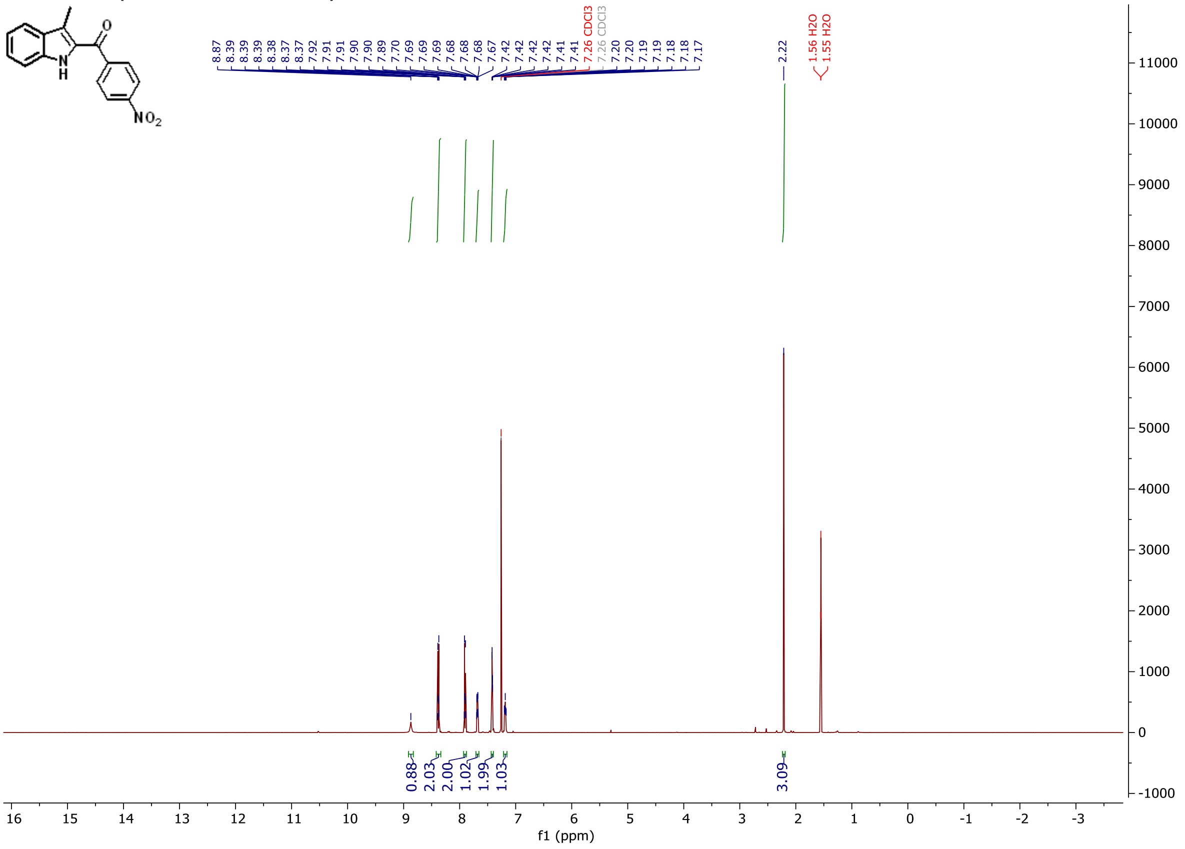

^{13}C{^{1}H} NMR (126 MHz, $CDCl_3$)

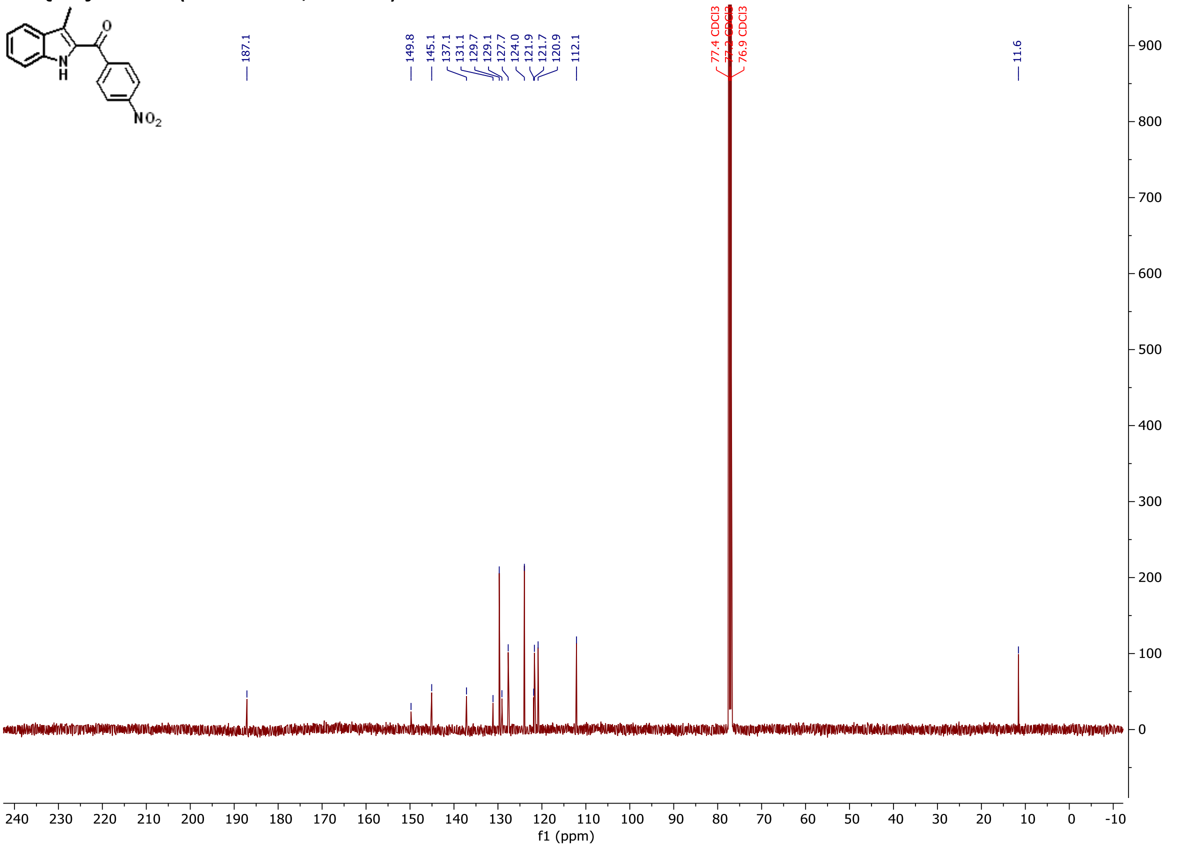

(5-methyl-1H-indol-2-yl)(4-nitrophenyl)methanone (2i)

^{1}H NMR (400 MHz, DMSO)

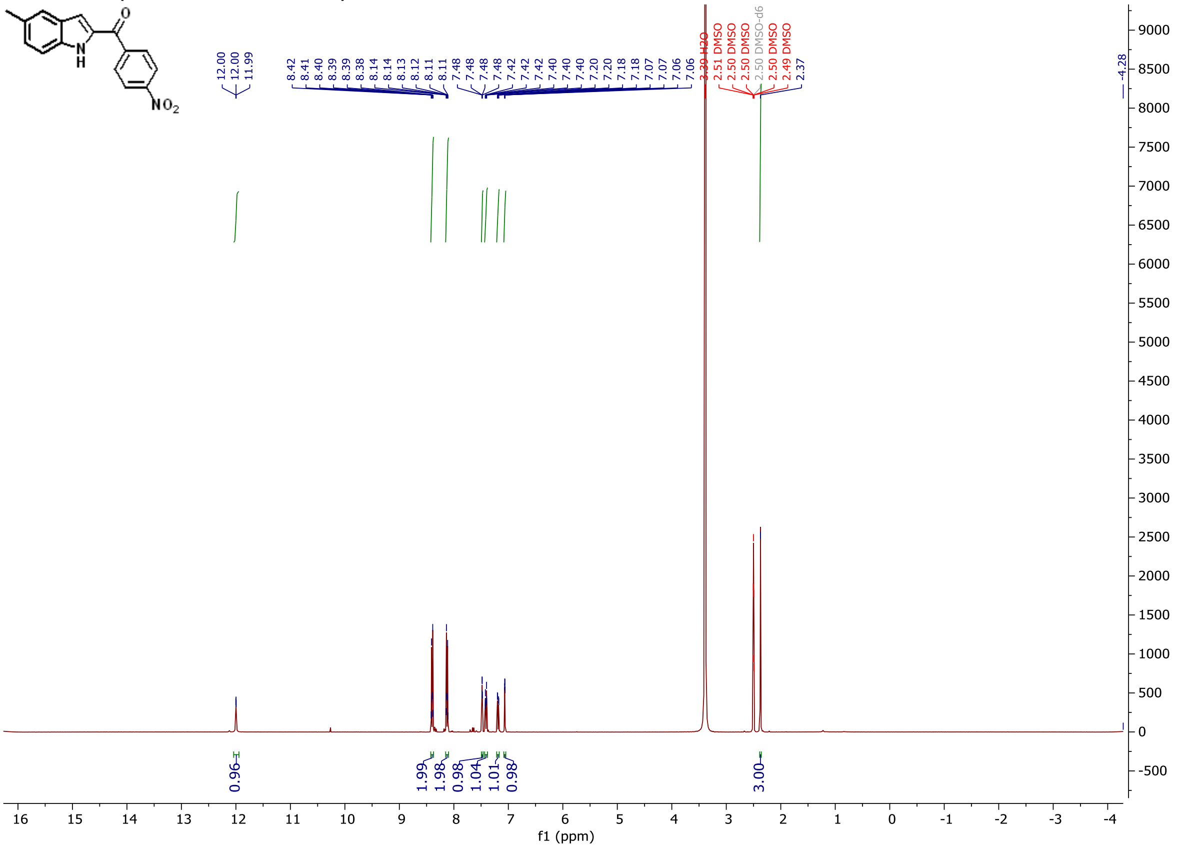

^{13}C{^{1}H} NMR (101 MHz, DMSO)

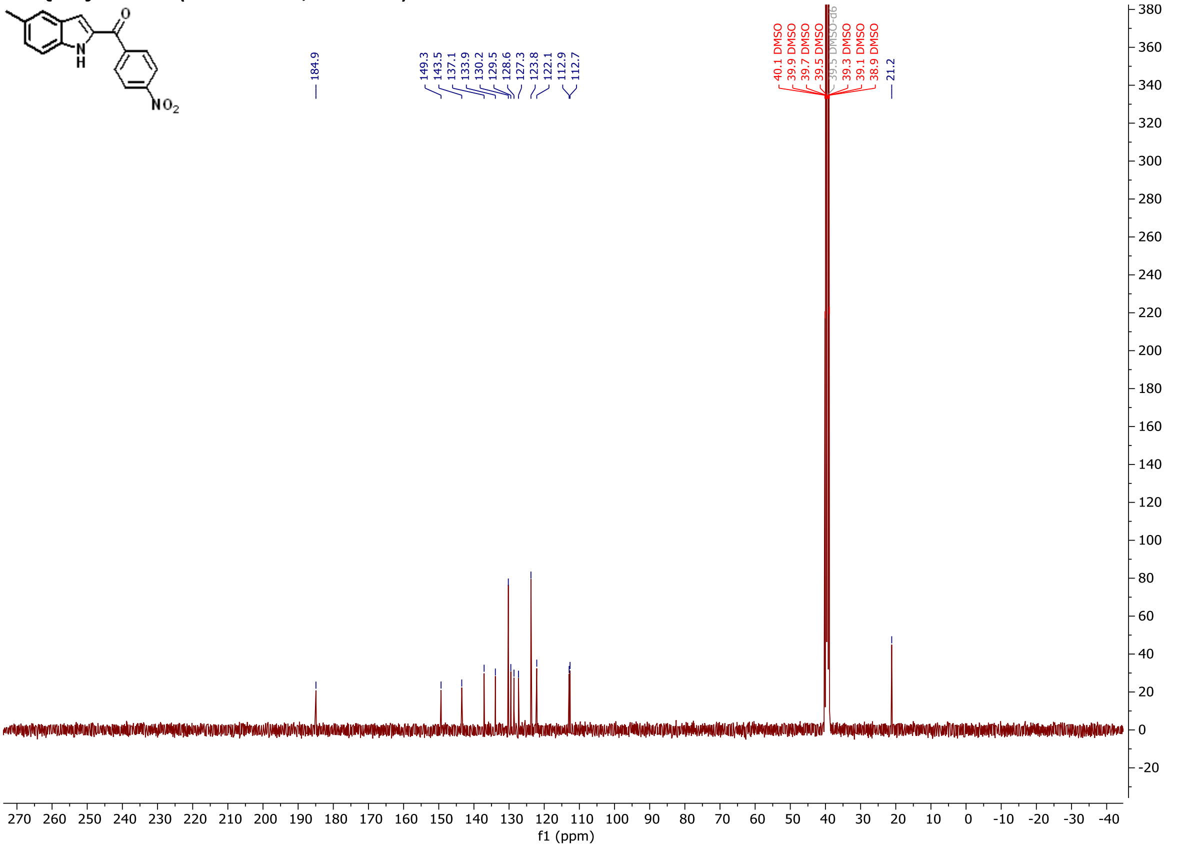

(5-chloro-1H-indol-2-yl)(4-nitrophenyl)methanone (2j)

^{1}H NMR (400 MHz, DMSO)

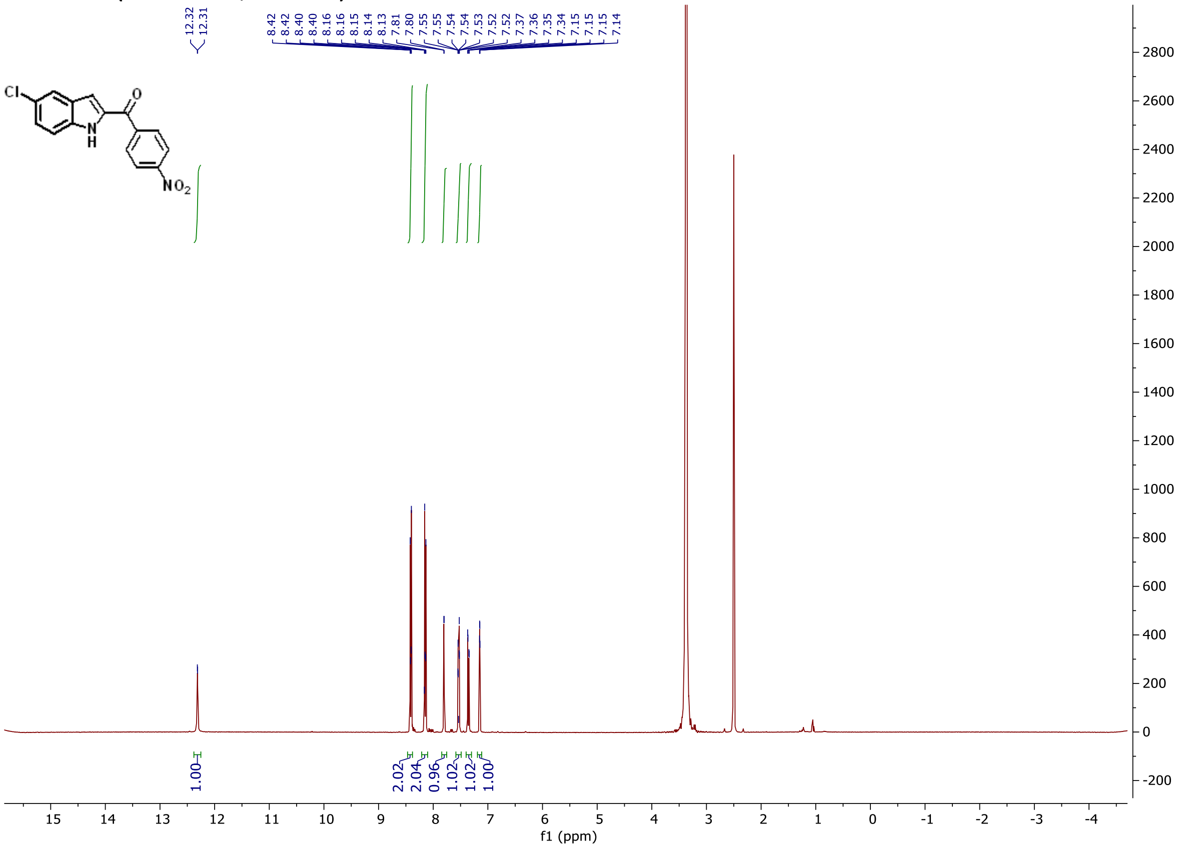

^{13}C{^{1}H} NMR (101 MHz, DMSO)

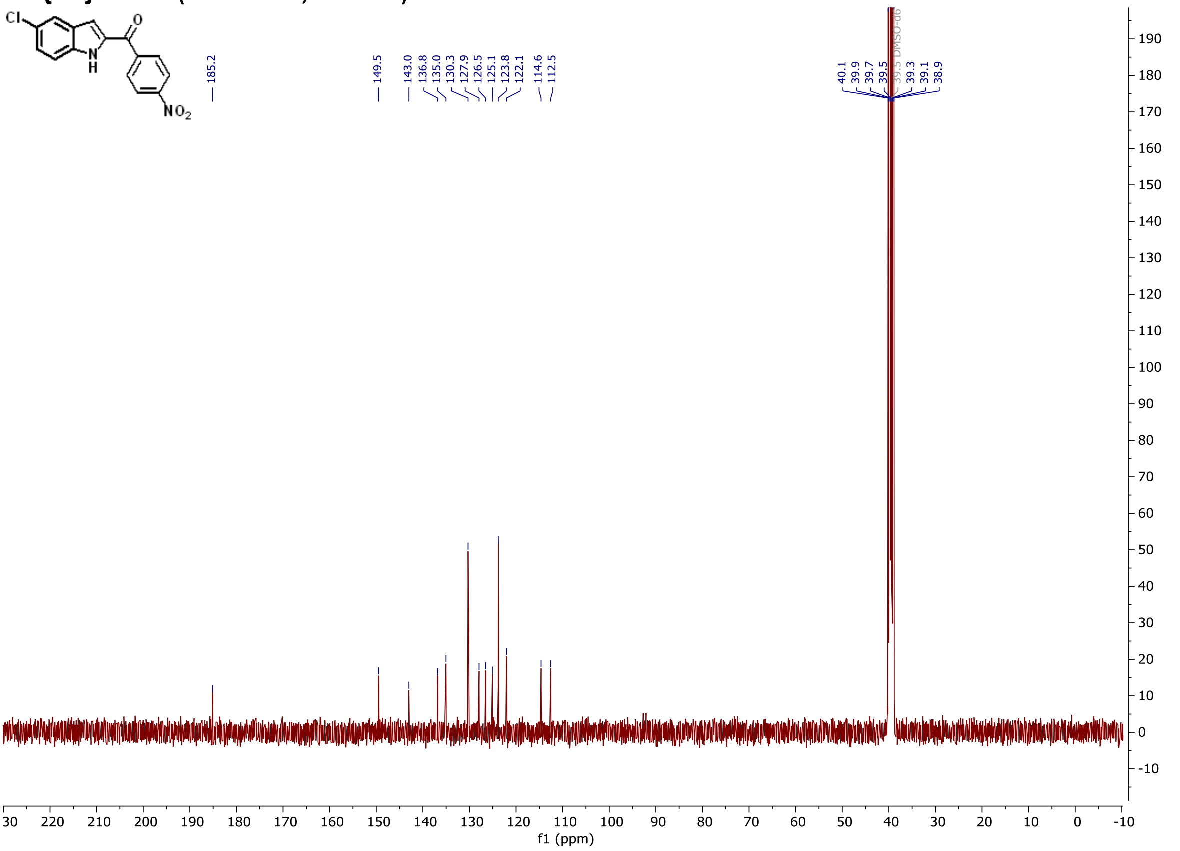

(5-fluoro-1H-indol-2-yl)(4-nitrophenyl)methanone (2k)

^{1}H NMR (400 MHz, DMSO)

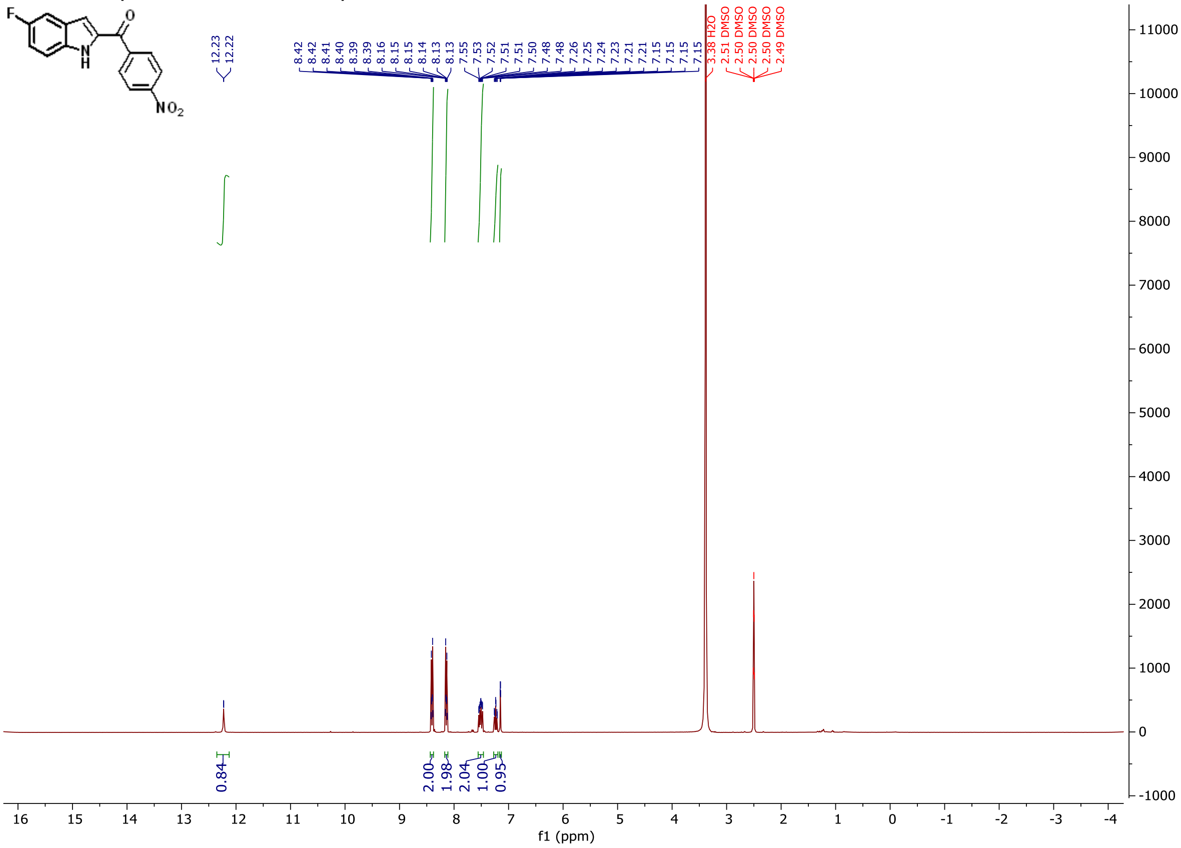

$^{13}C\{^{1}H\}$ NMR (400 MHz, DMSO)

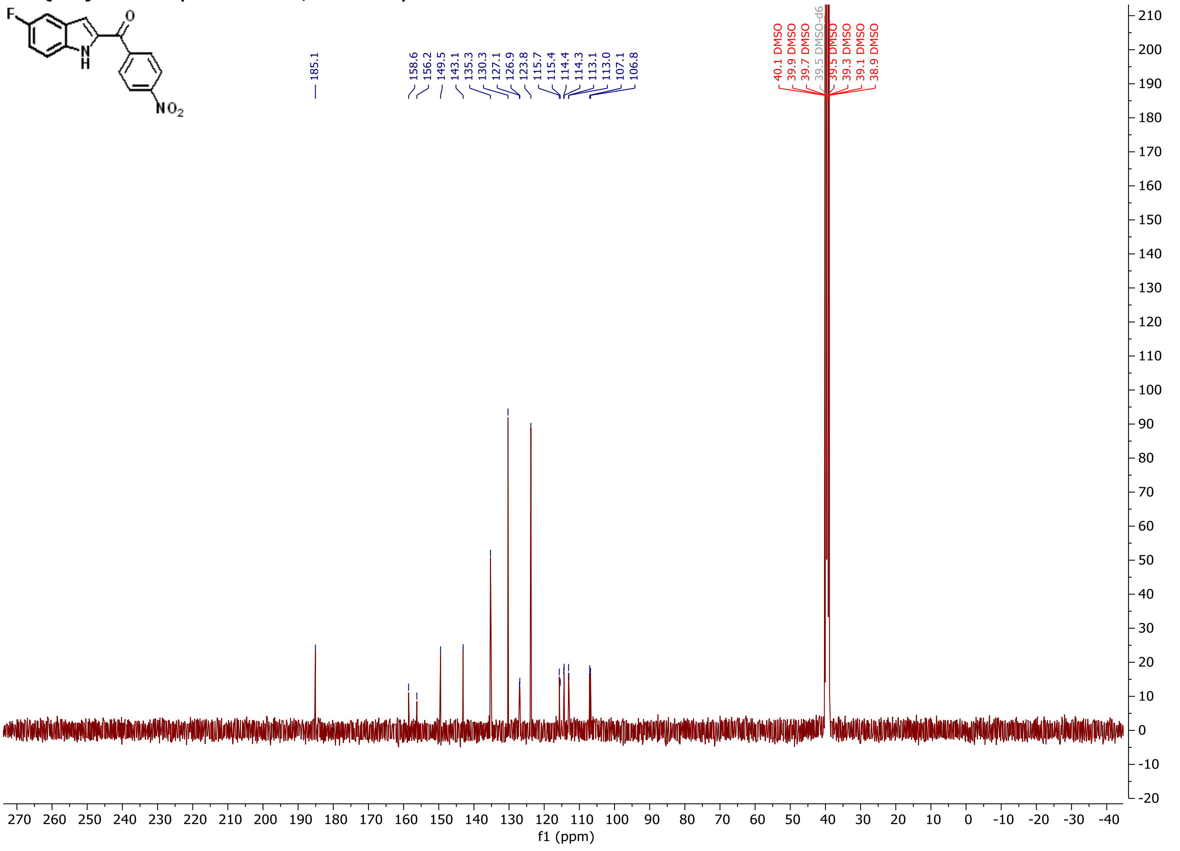

^{19}F NMR (376 MHz, DMSO)

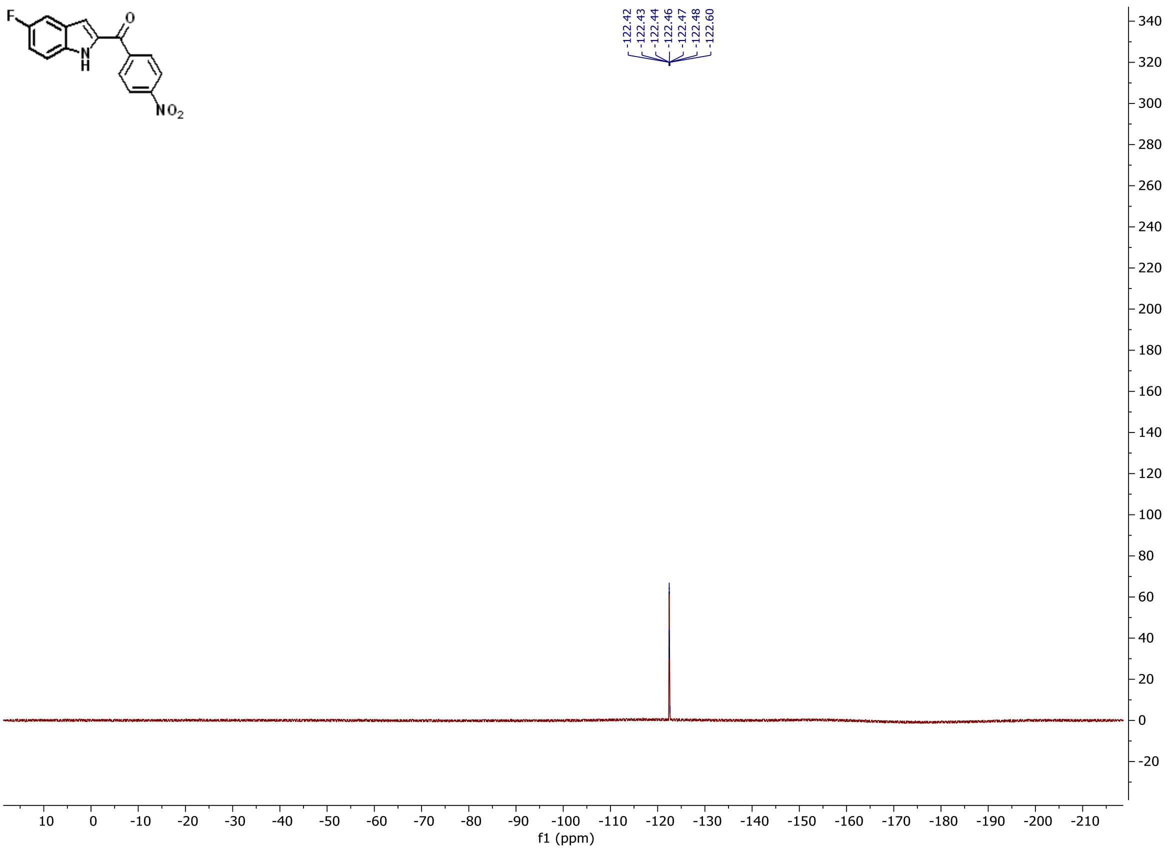

(6-methoxy-1H-indol-2-yl)(4-nitrophenyl)methanone (2l)

^{1}H NMR (400 MHz, $CDCl_3$)

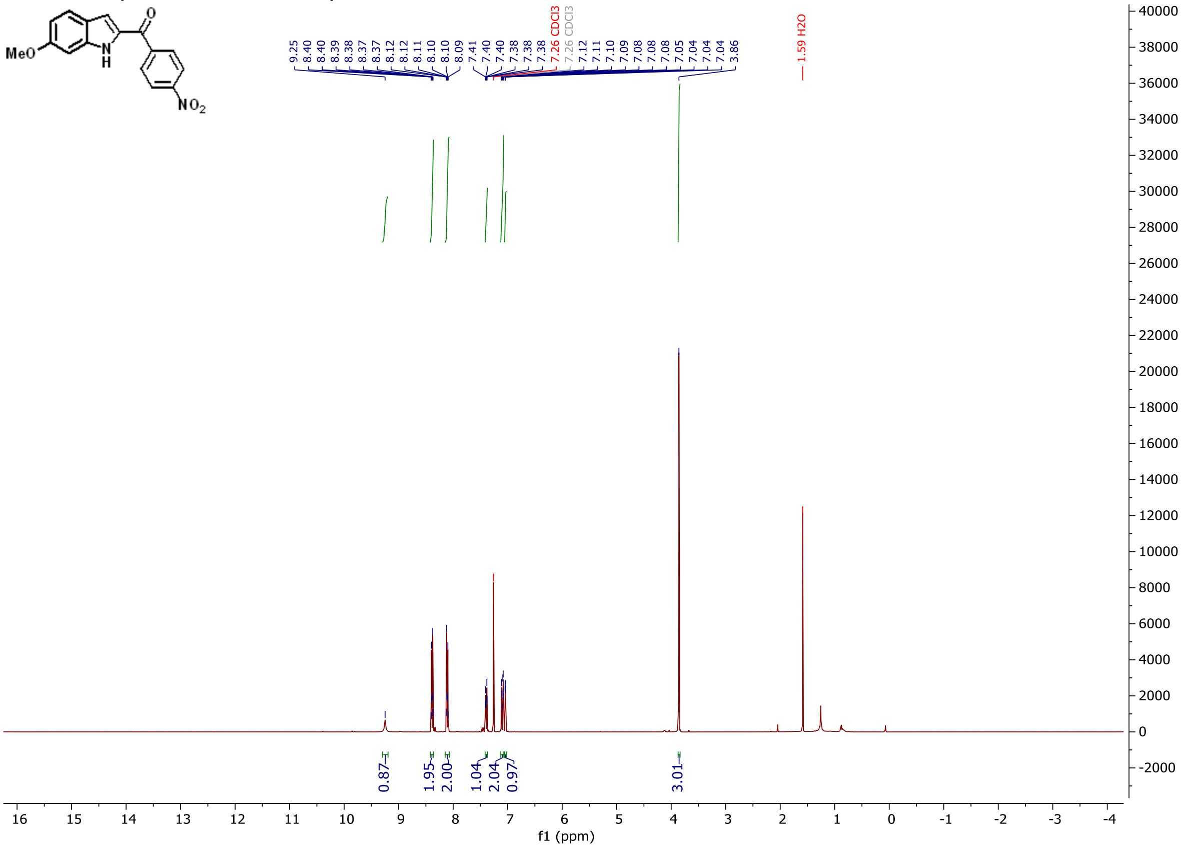

^{13}C{^{1}H} NMR (101 MHz, $CDCl_3$)

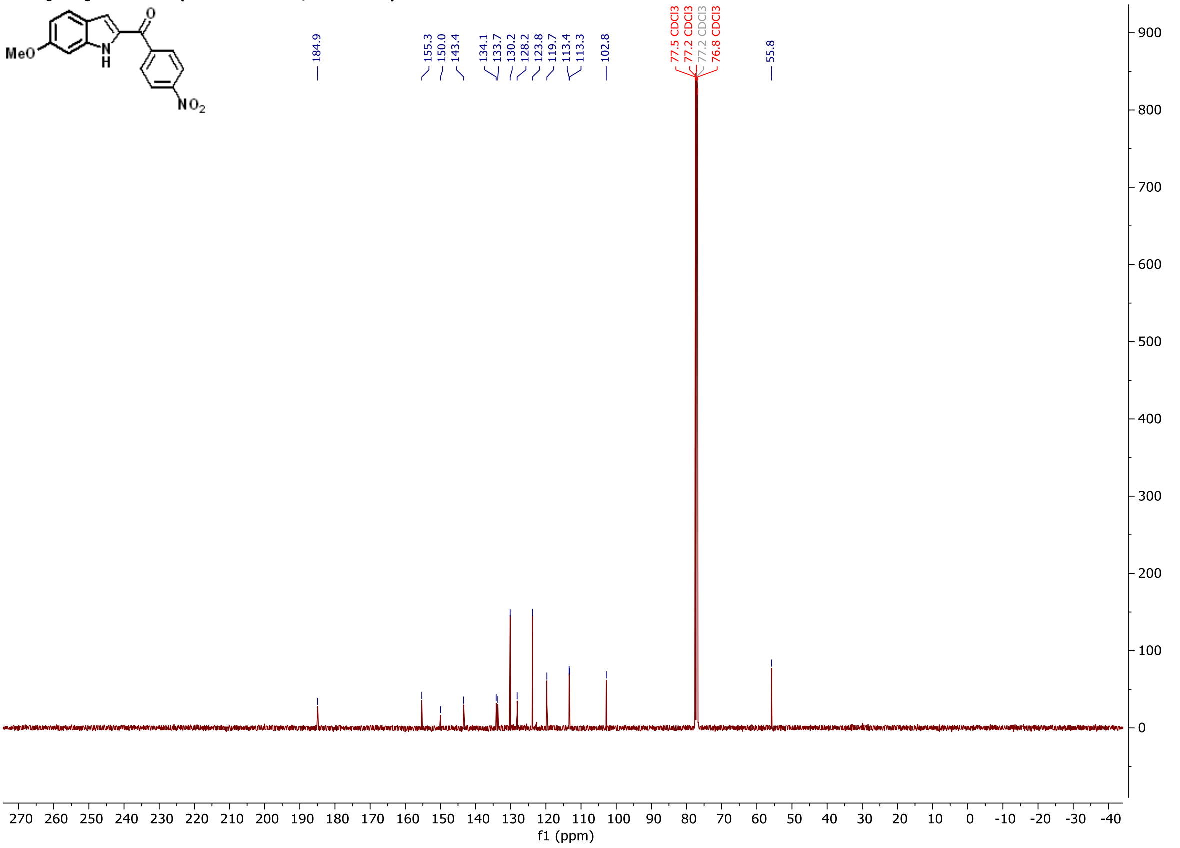

(6-bromo-1H-indol-2-yl)(4-nitrophenyl)methanone (2m)

^{1}H NMR (400 MHz, DMSO)

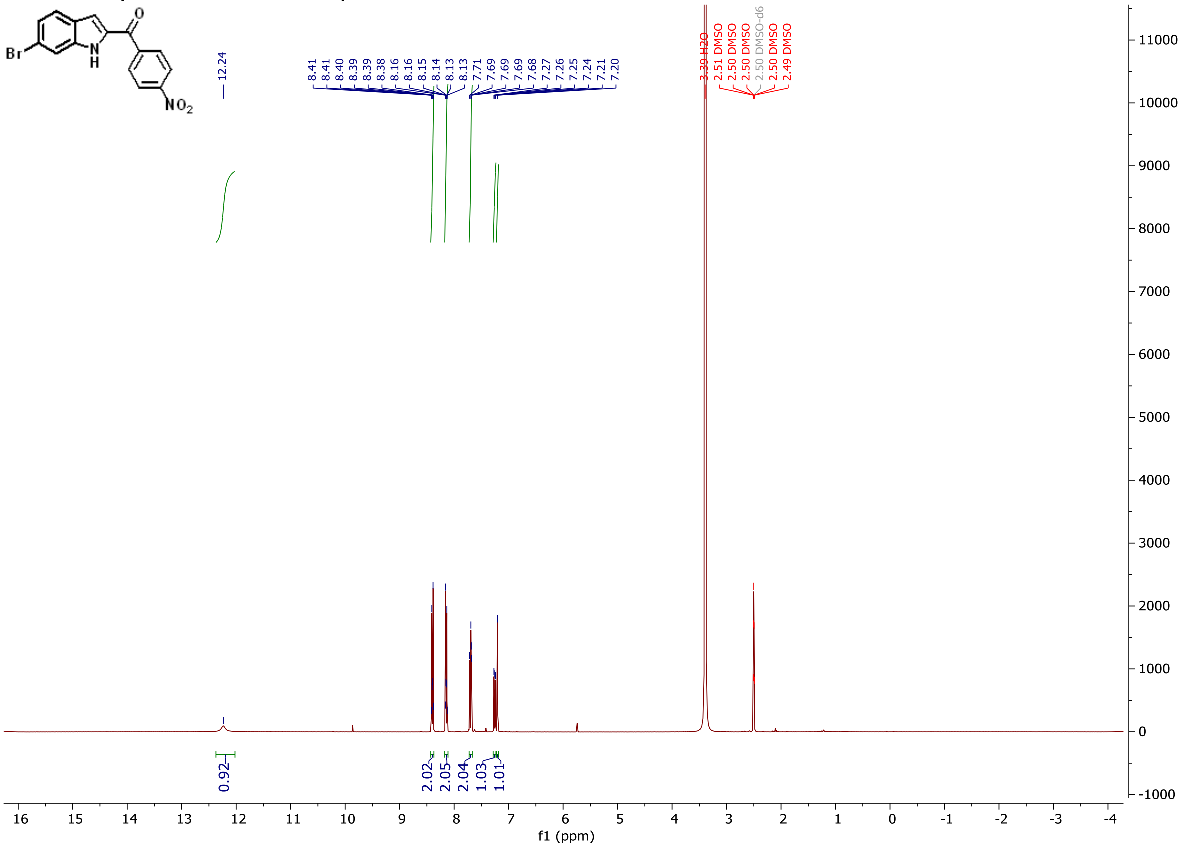

^{13}C{^{1}H} NMR (101 MHz, DMSO)

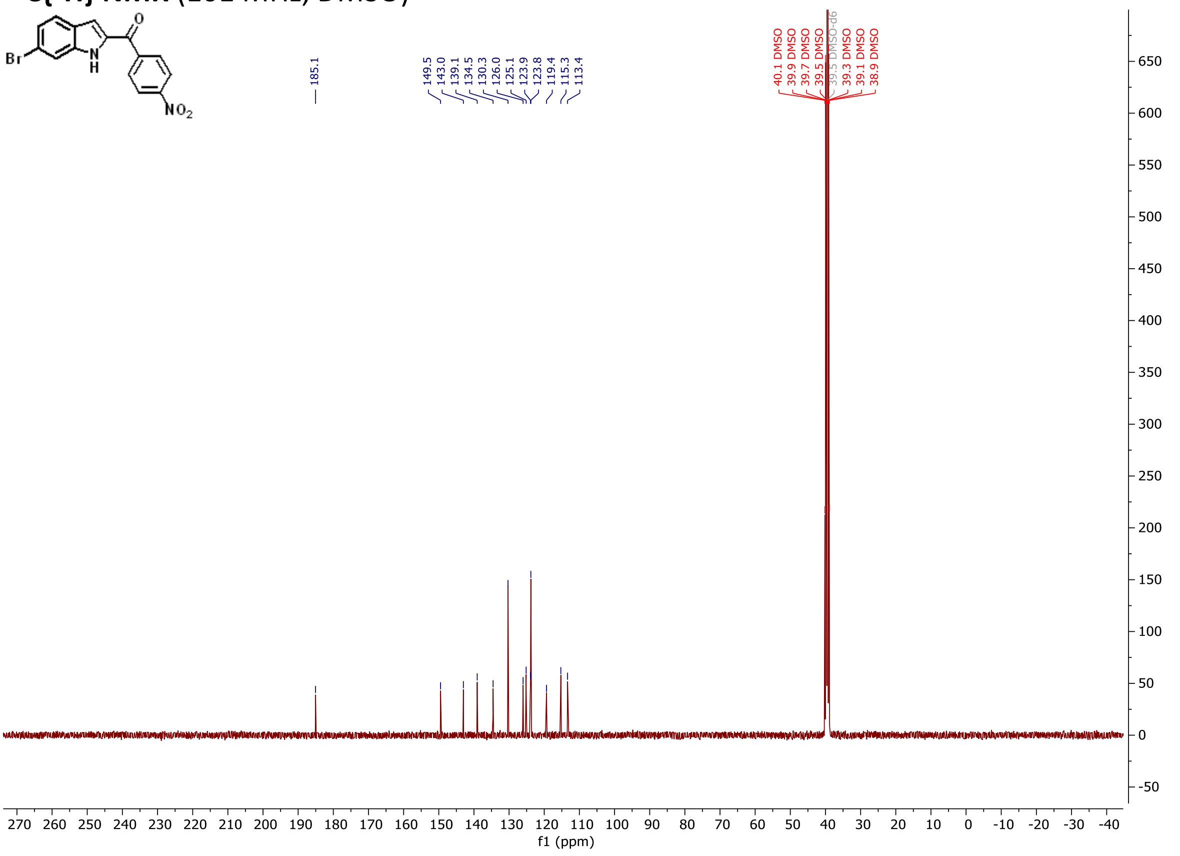

(6-chloro-1H-indol-2-yl)(4-nitrophenyl)methanone (2n)

^{1}H NMR (500 MHz, DMSO-d$_6$)

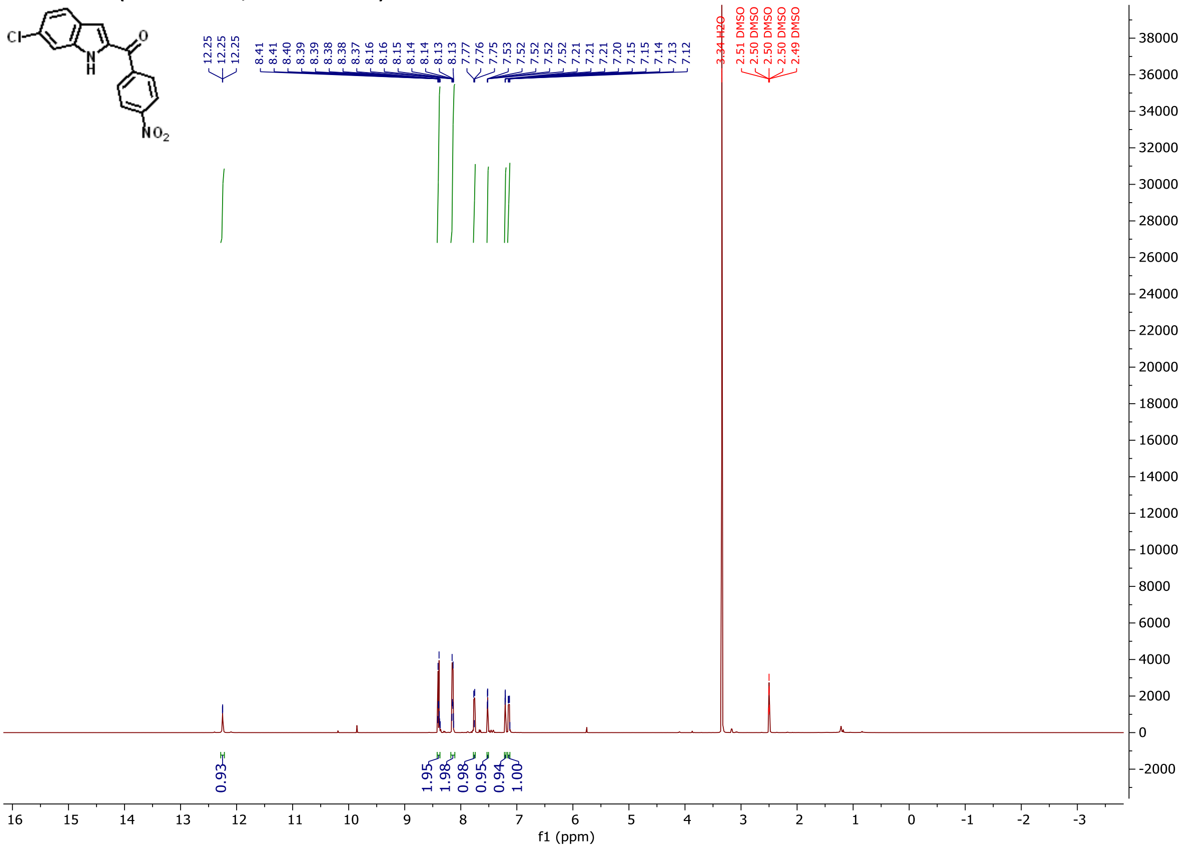

^{13}C{^{1}H} NMR (126 MHz, DMSO-d$_6$)

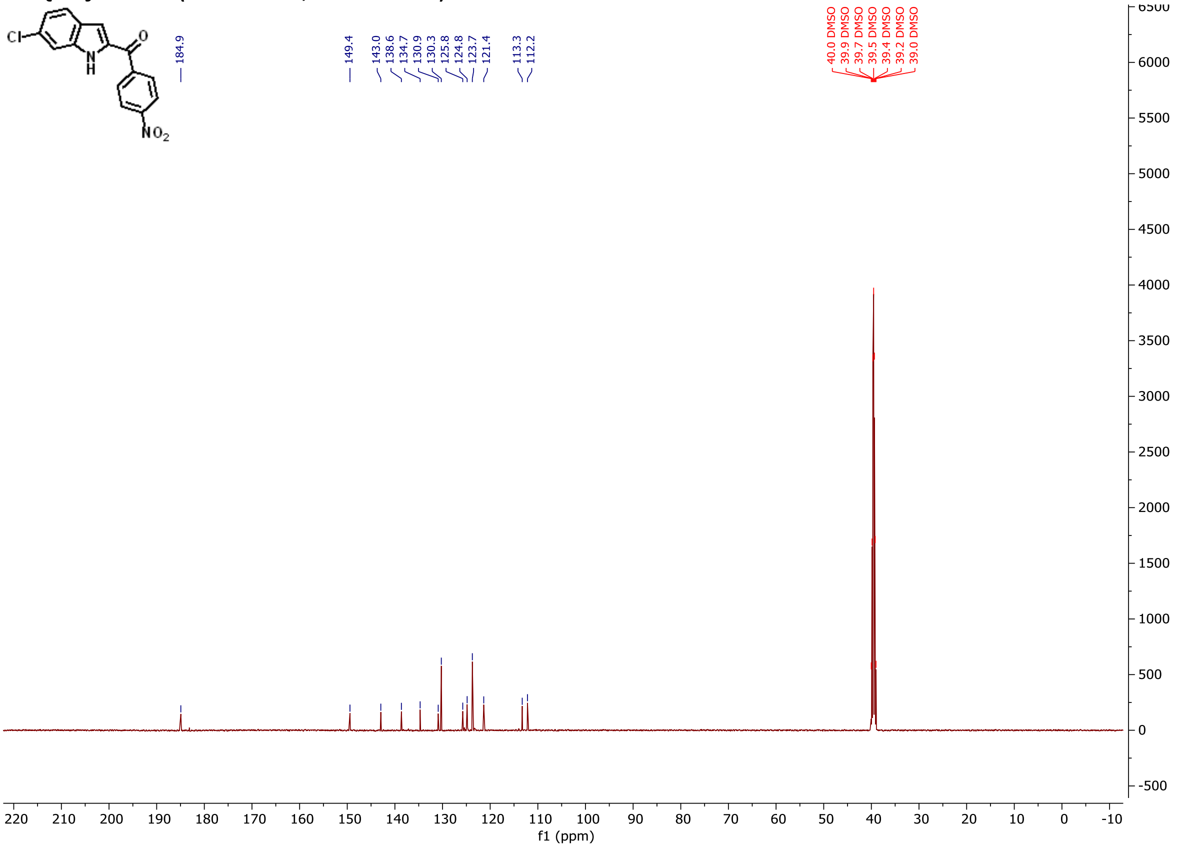

1-((4-nitrophenyl)sulfonyl)-1H-pyrrole-2-carbaldehyde (3a)

^{1}H NMR (400 MHz, $CDCl_3$)

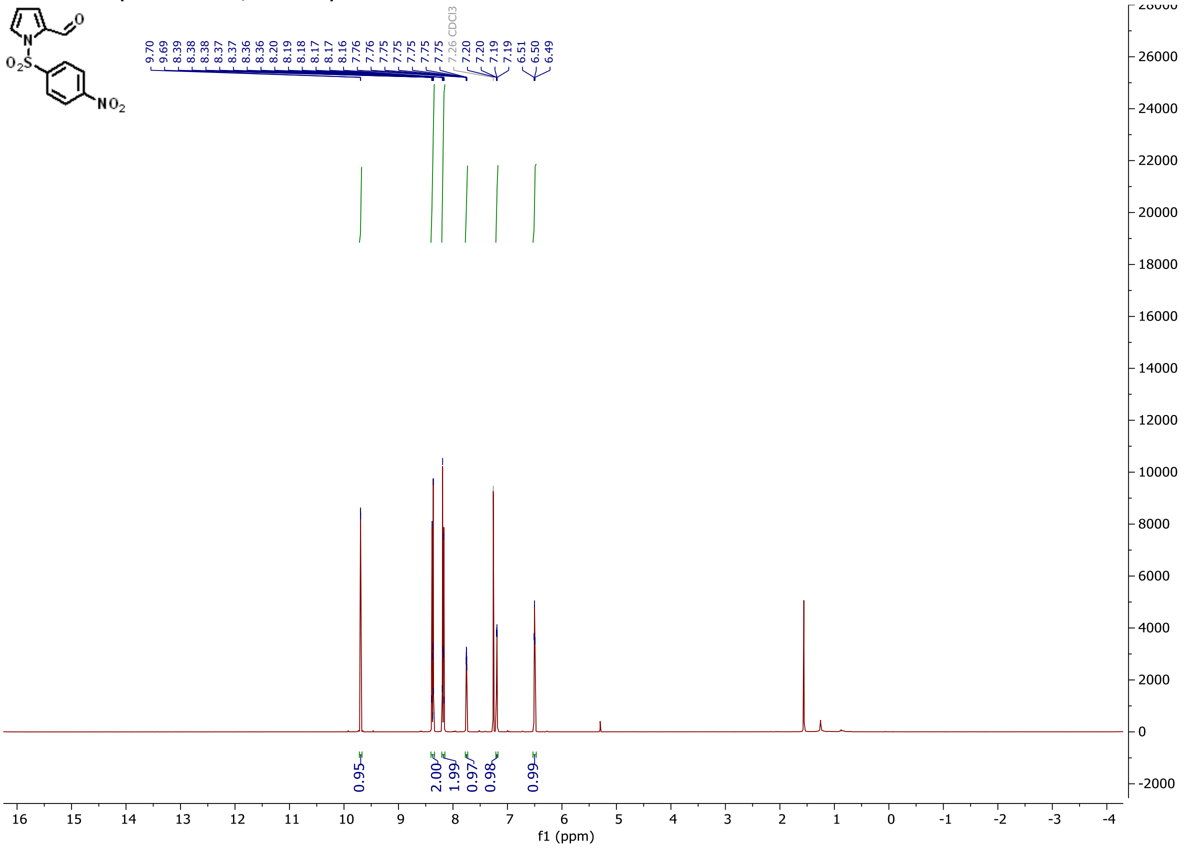

^{13}C{^{1}H} NMR (101 MHz, $CDCl_3$)

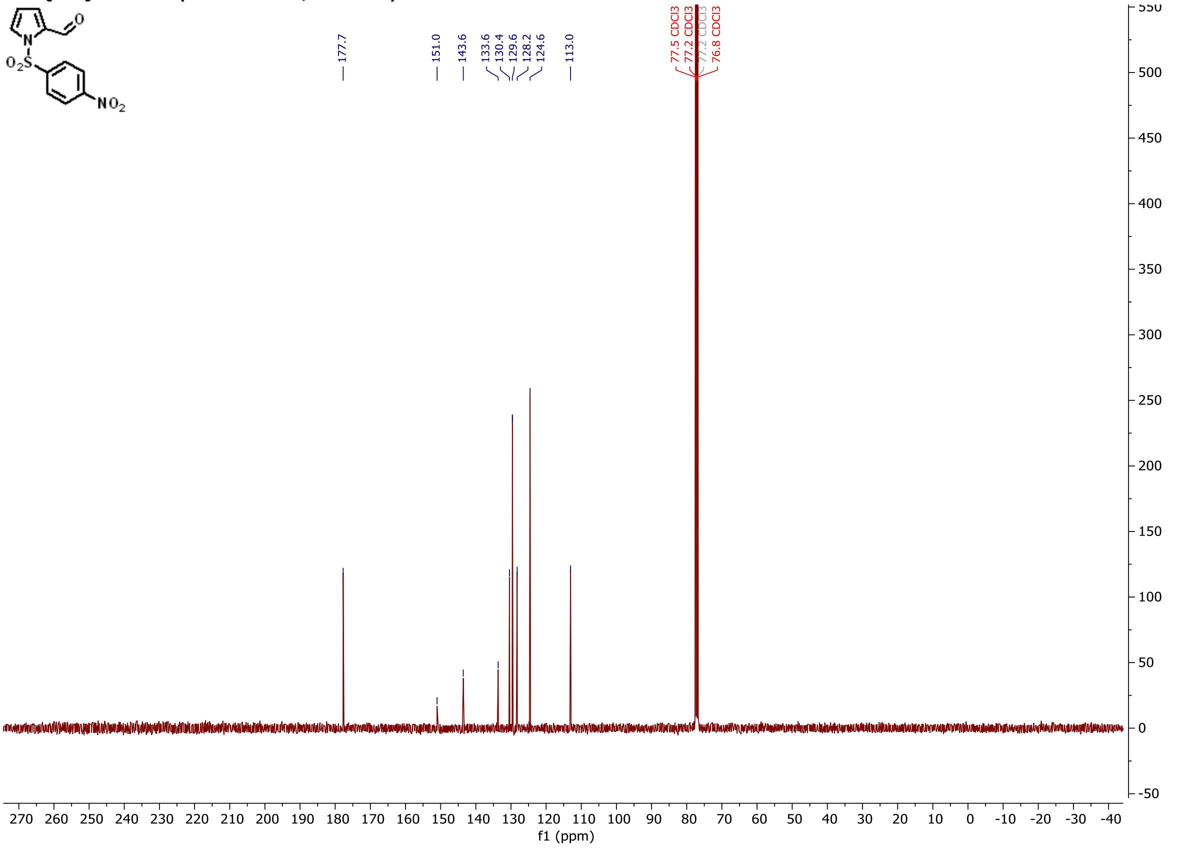

1-((2-nitrophenyl)sulfonyl)-1H-pyrrole-2-carbaldehyde (3b)

^{1}H NMR (500 MHz, $CDCl_3$)

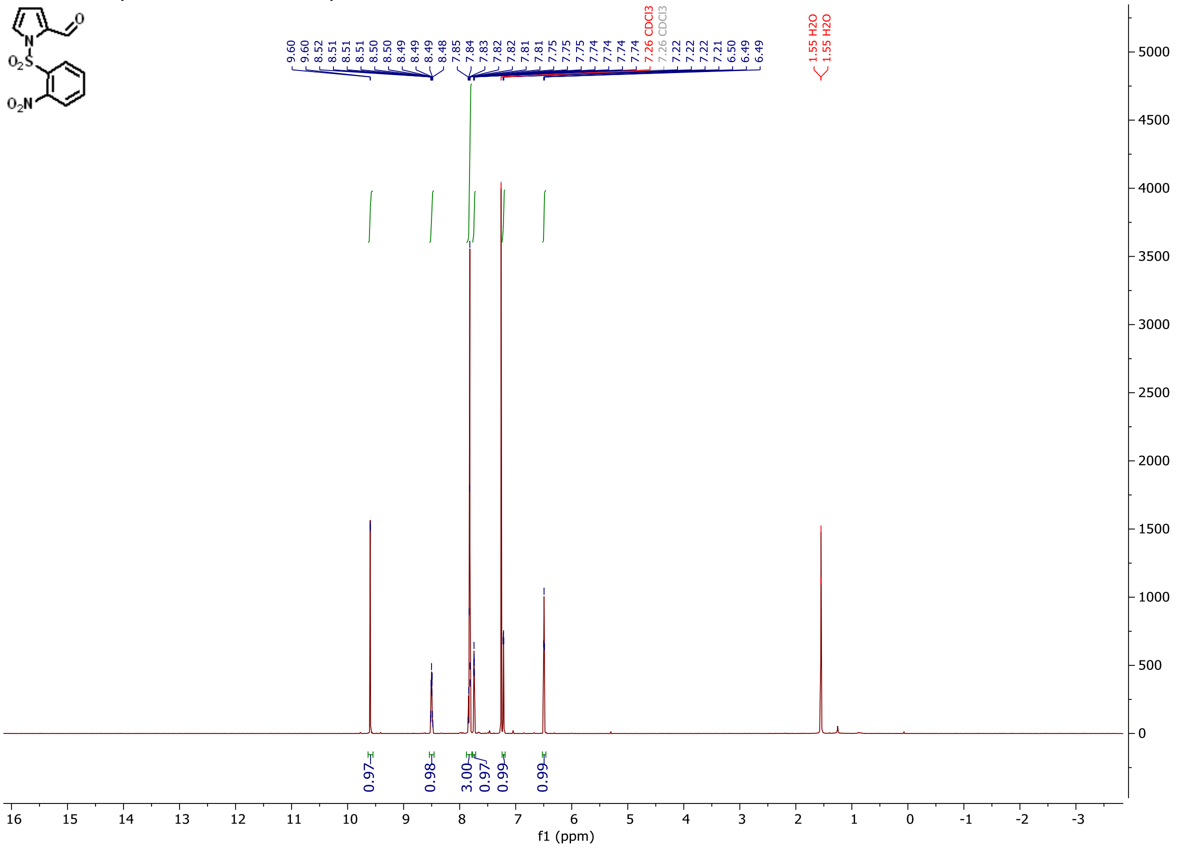

^{13}C{^{1}H} NMR (126 MHz, $CDCl_3$)

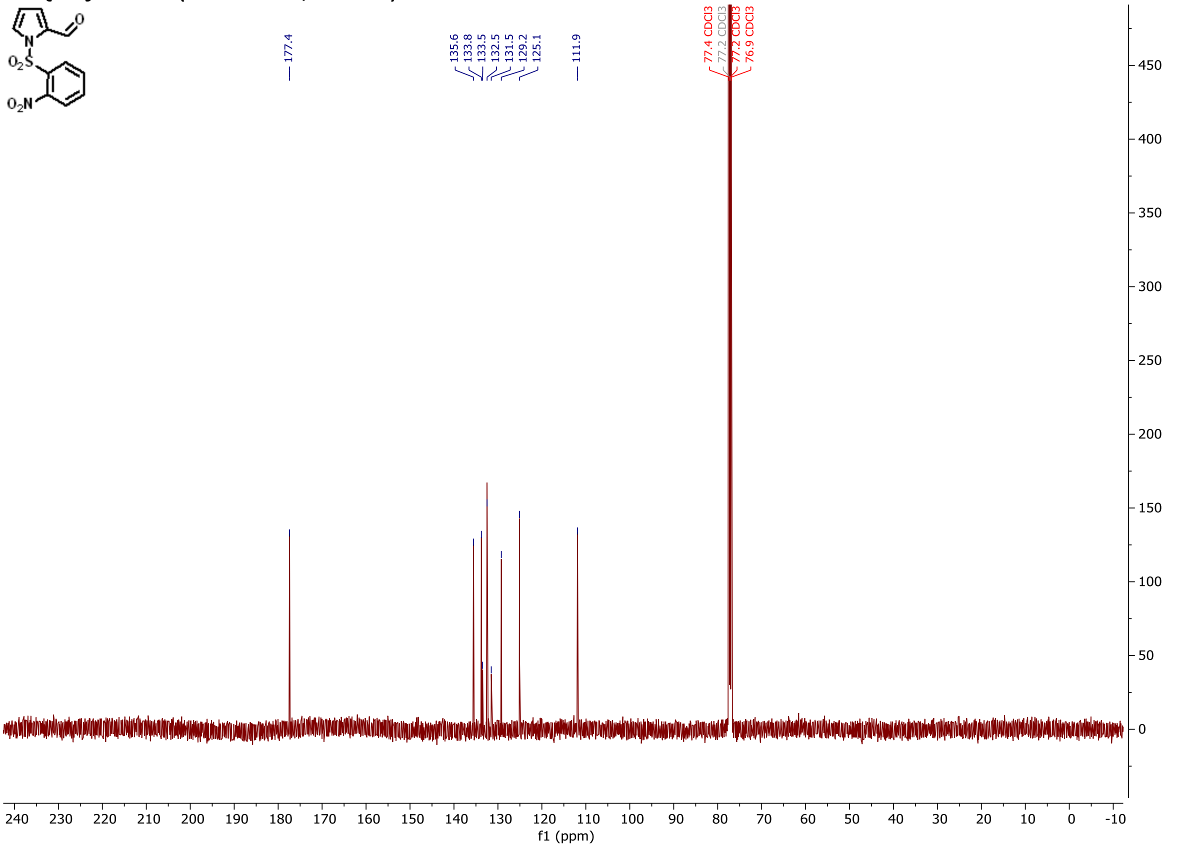

1-((5-fluoro-2-nitrophenyl)sulfonyl)-1H-pyrrole-2-carbaldehyde (3c)

^{1}H NMR (500 MHz, $CDCl_3$)

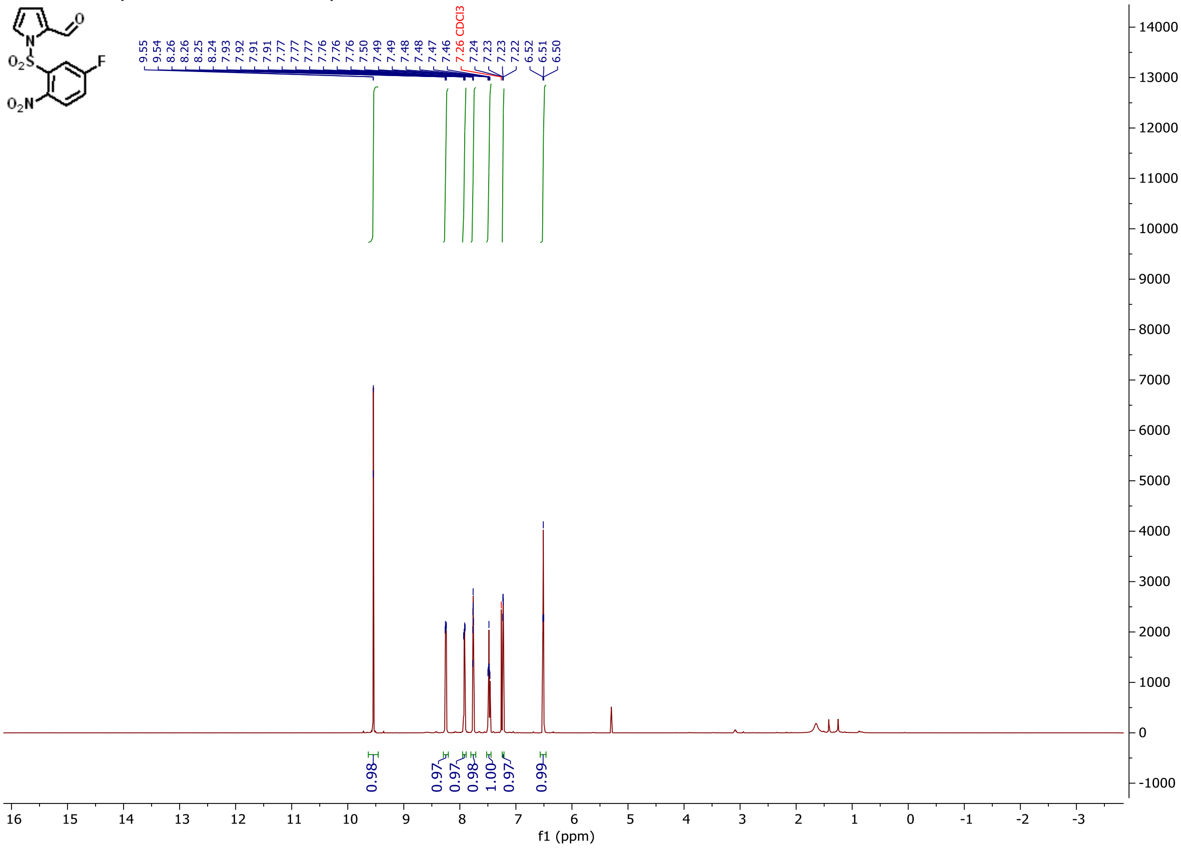

$^{13}C\{^1H\}$ NMR (126 MHz, $CDCl_3$)

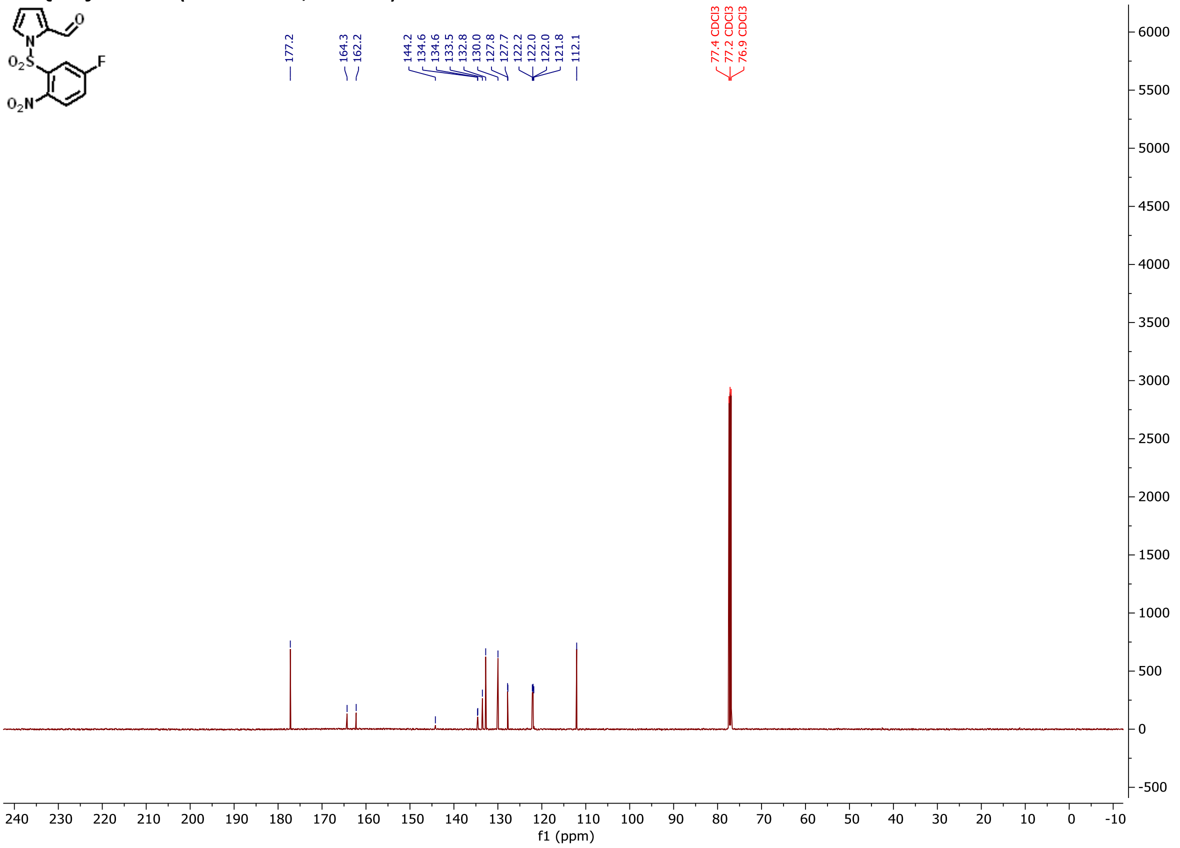

^{19}F NMR (471 MHz, $CDCl_3$)

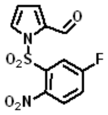

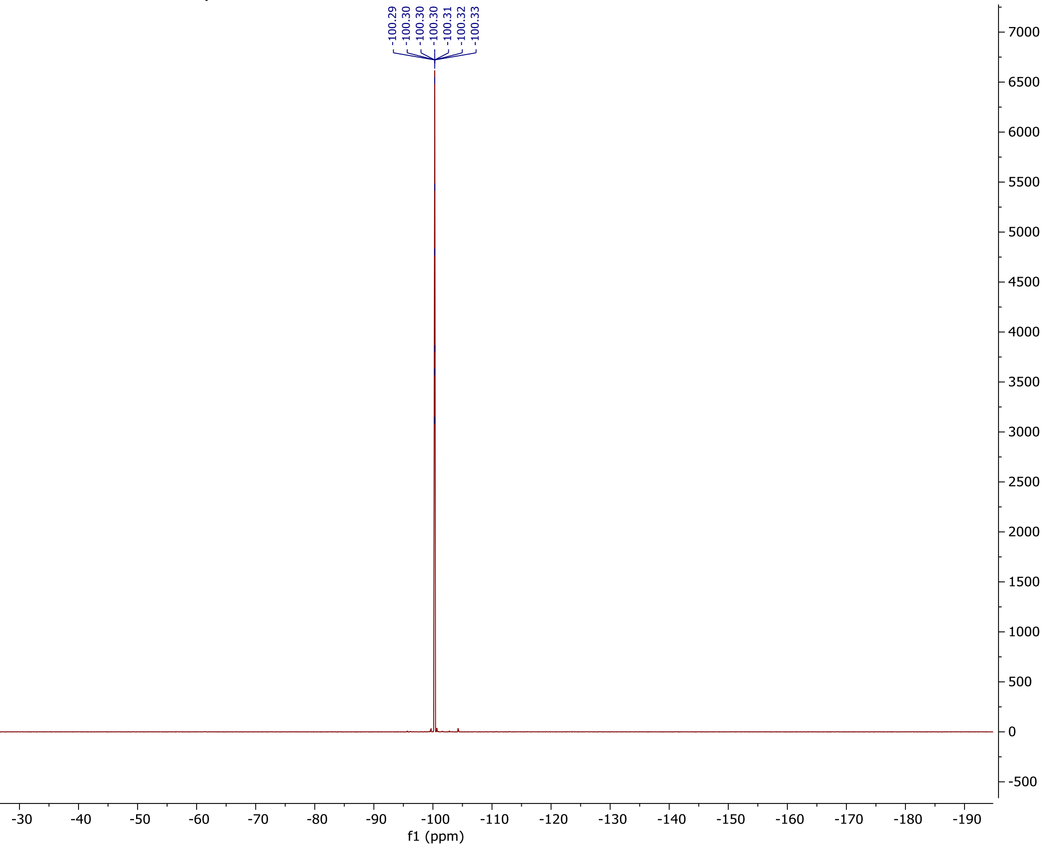

1-((4-bromo-2-nitrophenyl)sulfonyl)-1H-pyrrole-2-carbaldehyde (3d)

^{1}H NMR (500 MHz, $CDCl_3$)

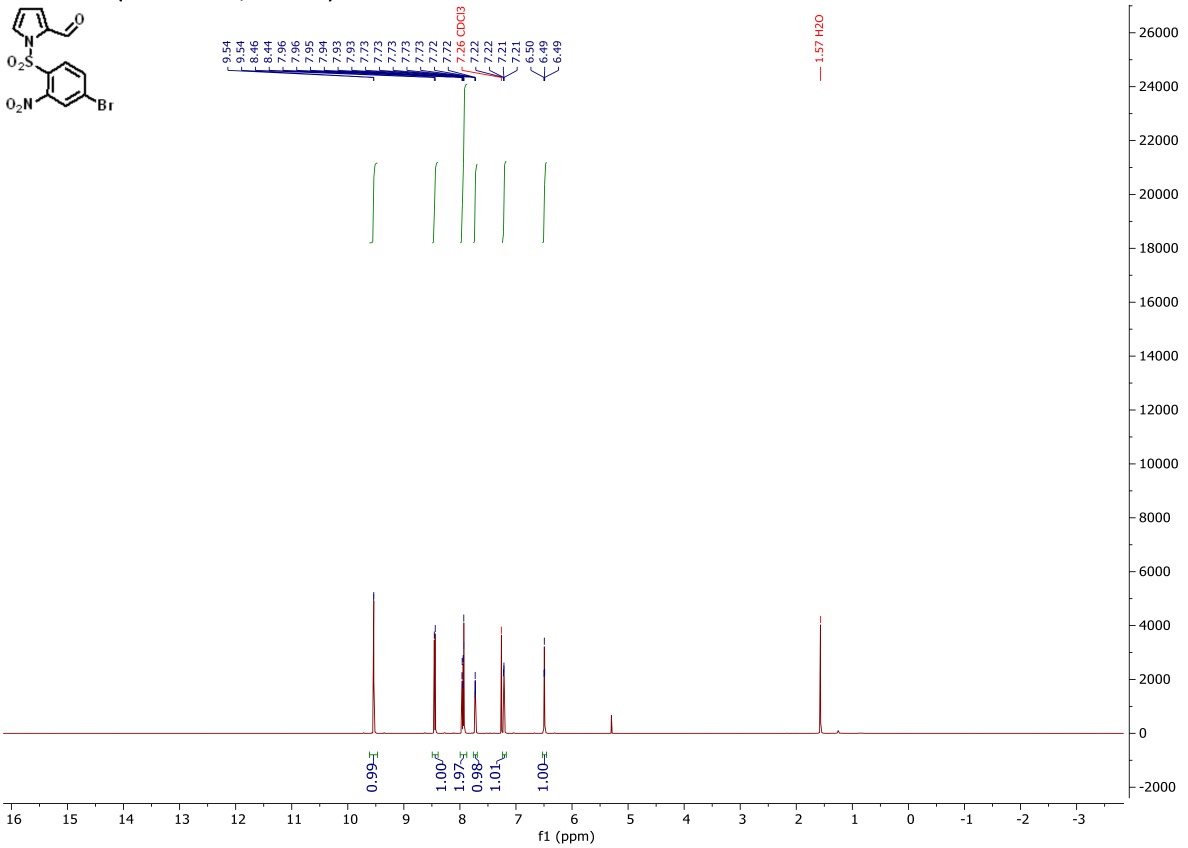

^{13}C{^{1}H} NMR (126 MHz, $CDCl_3$)

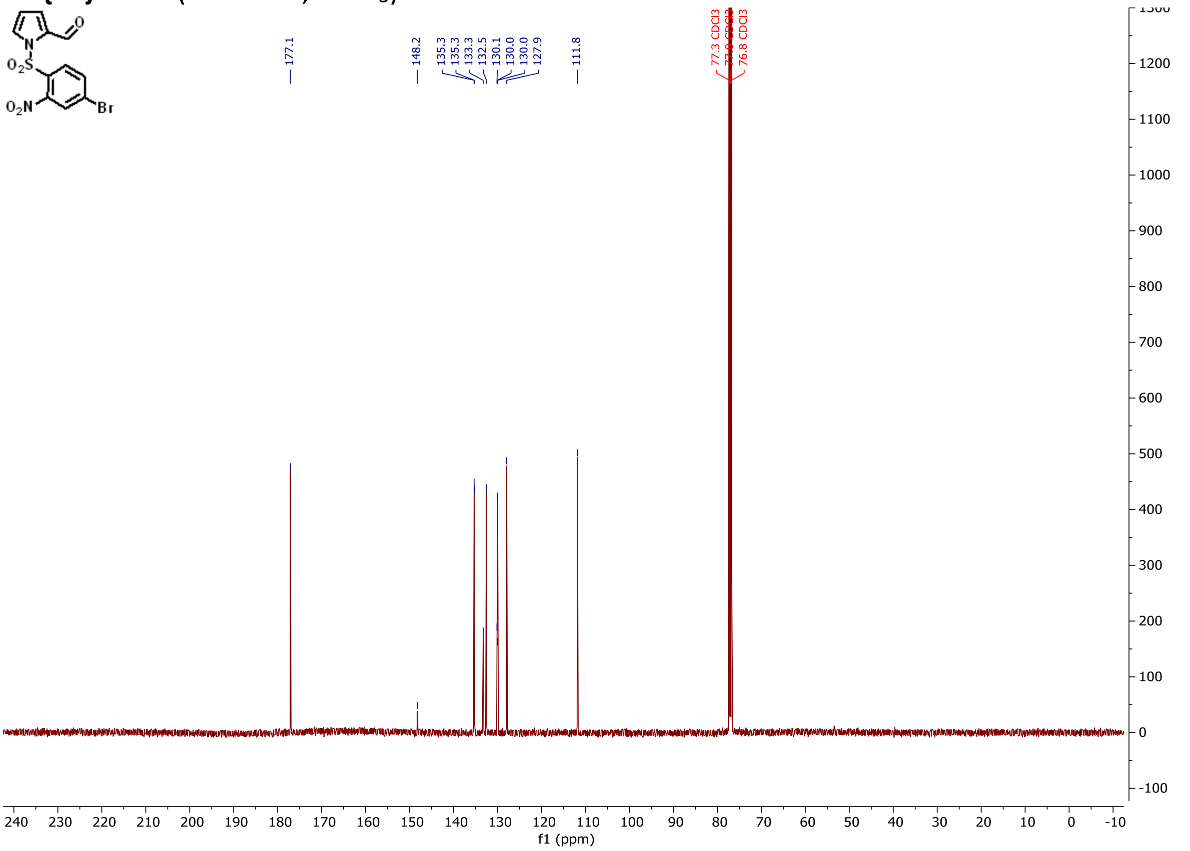

1-((2-fluoro-4-nitrophenyl)sulfonyl)-1H-pyrrole-2-carbaldehyde (3e)

^{1}H NMR (500 MHz, $CDCl_3$)

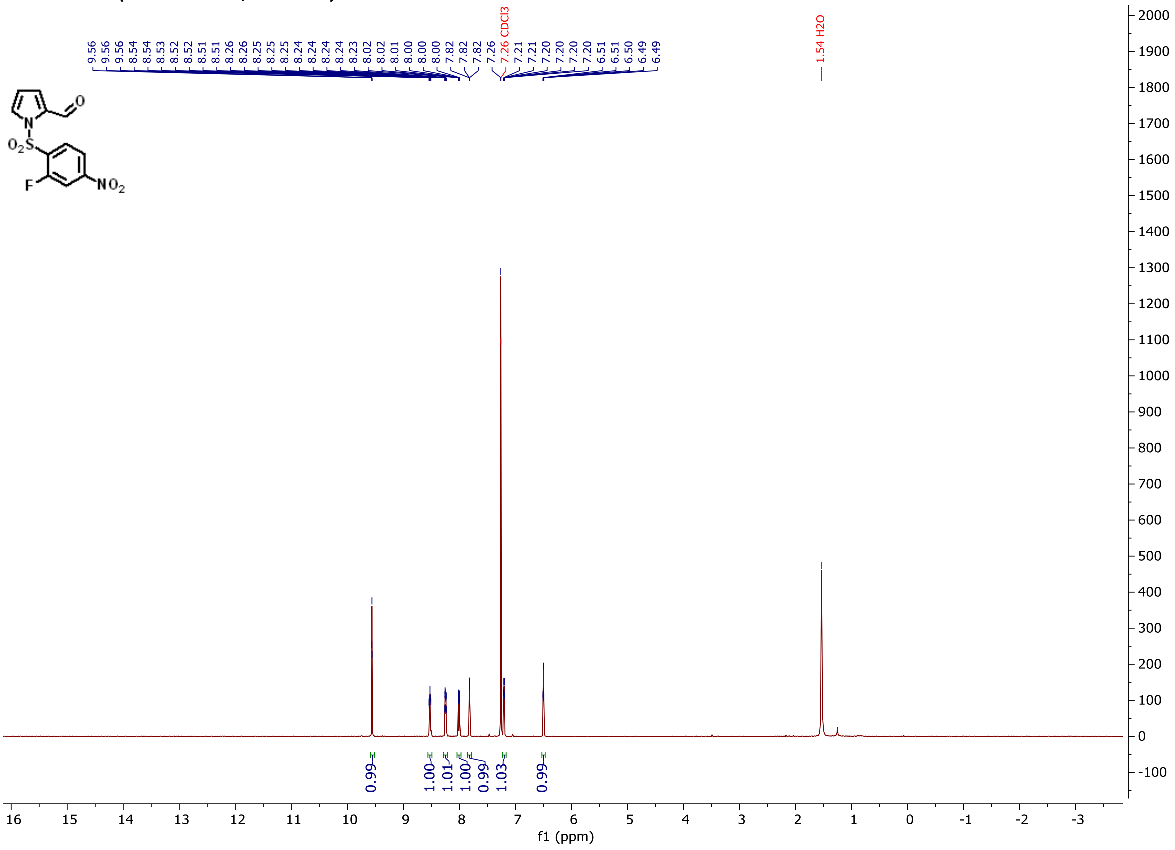

^{13}C{^{1}H} NMR (126 MHz, $CDCl_3$)

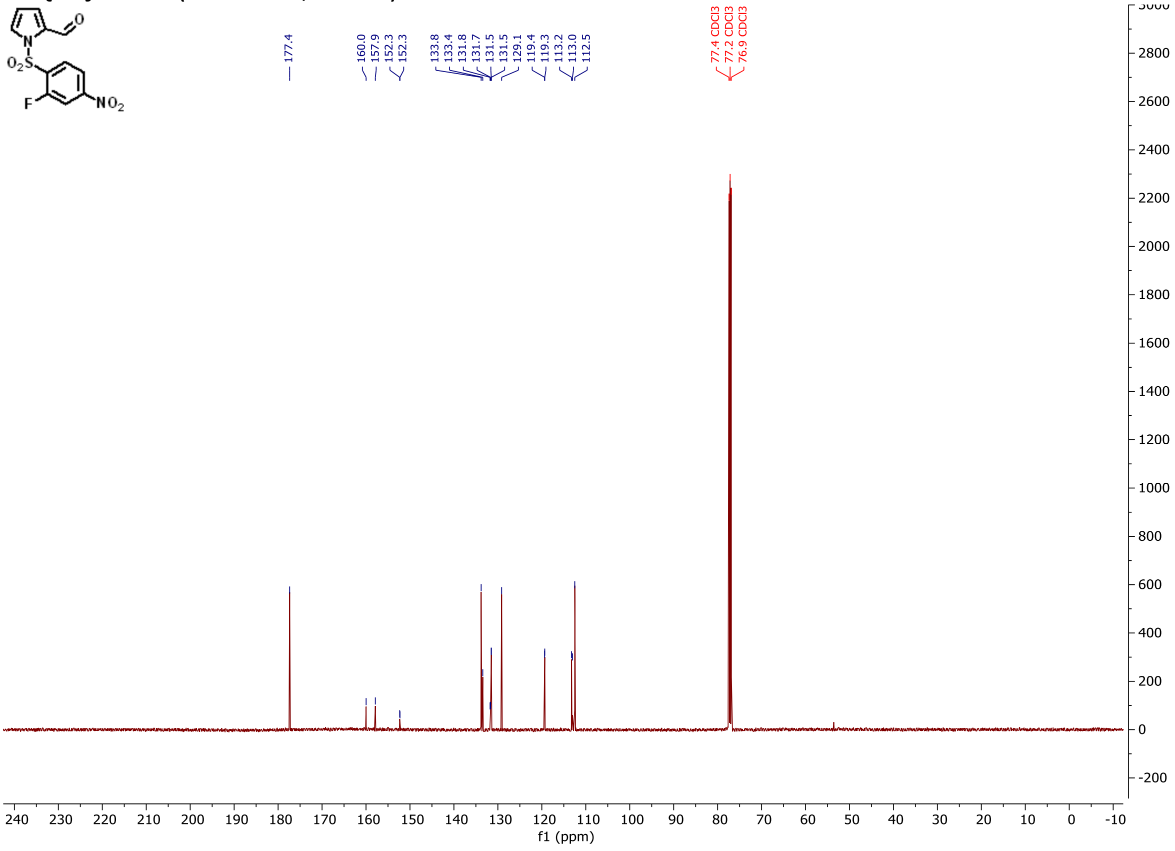

^{19}F NMR (471 MHz, $CDCl_3$)

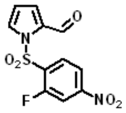

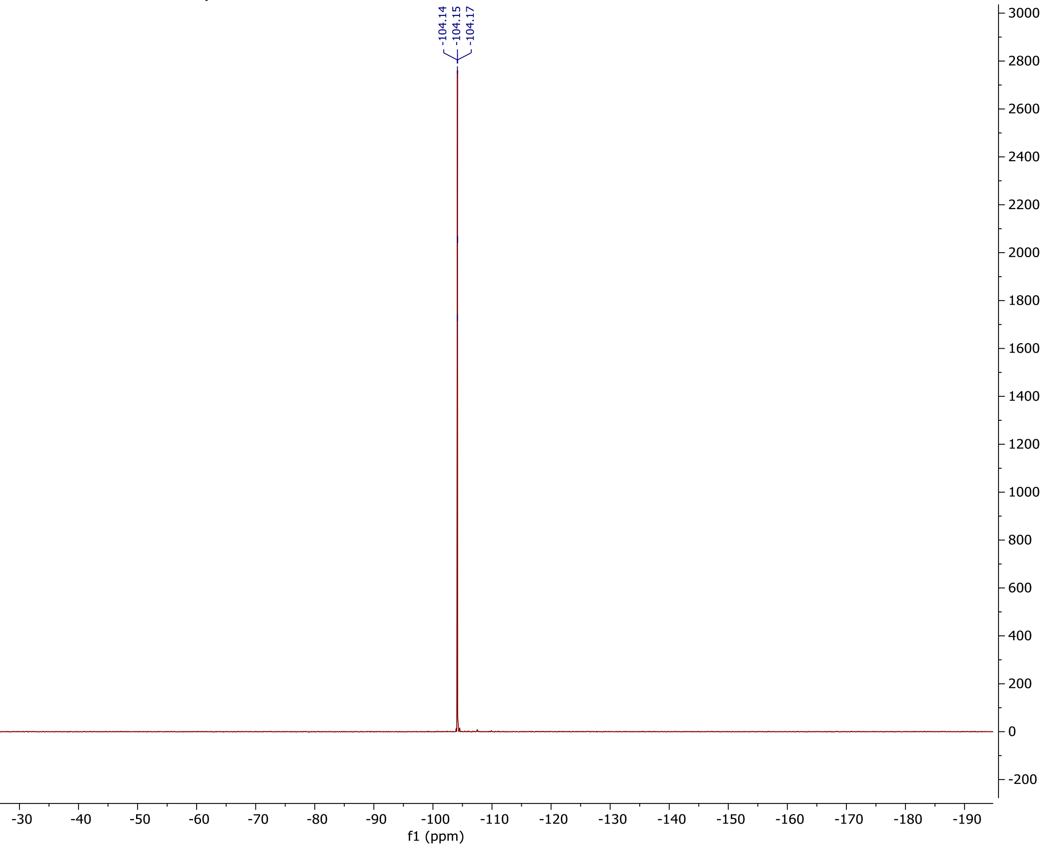

1-((4-nitro-3-(trifluoromethyl)phenyl)sulfonyl)-1H-pyrrole-2-carbaldehyde (3f)

^{1}H NMR (500 MHz, $CDCl_3$)

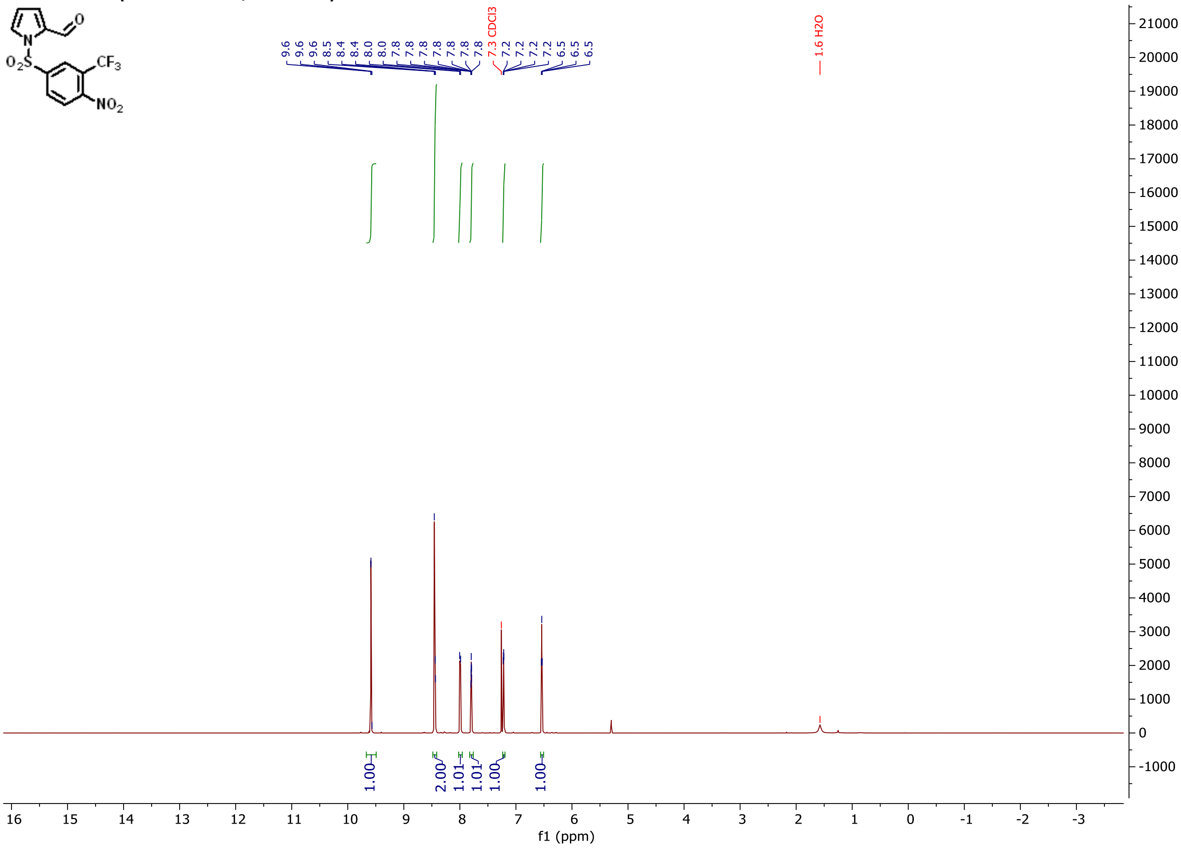

^{13}C{^{1}H} NMR (126 MHz, $CDCl_3$)

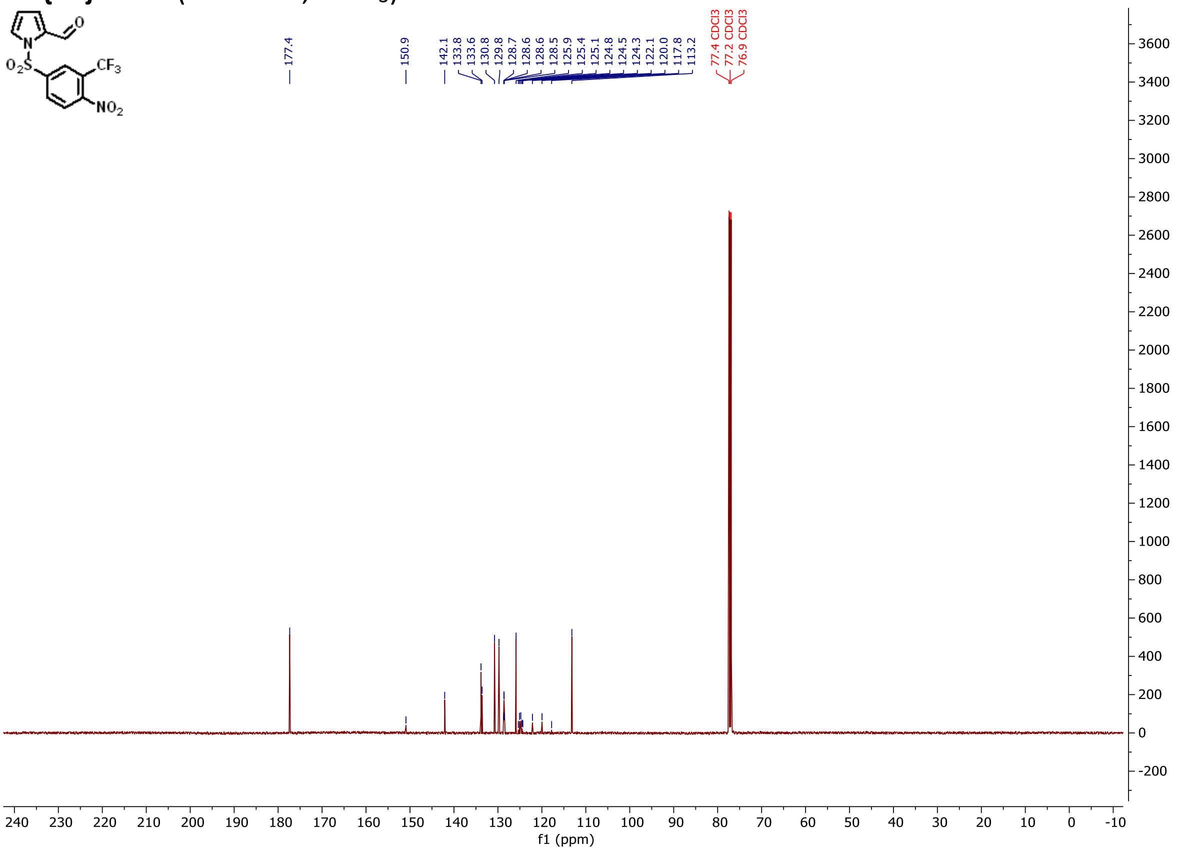

^{19}F NMR (471 MHz, $CDCl_3$)

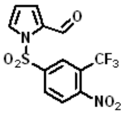

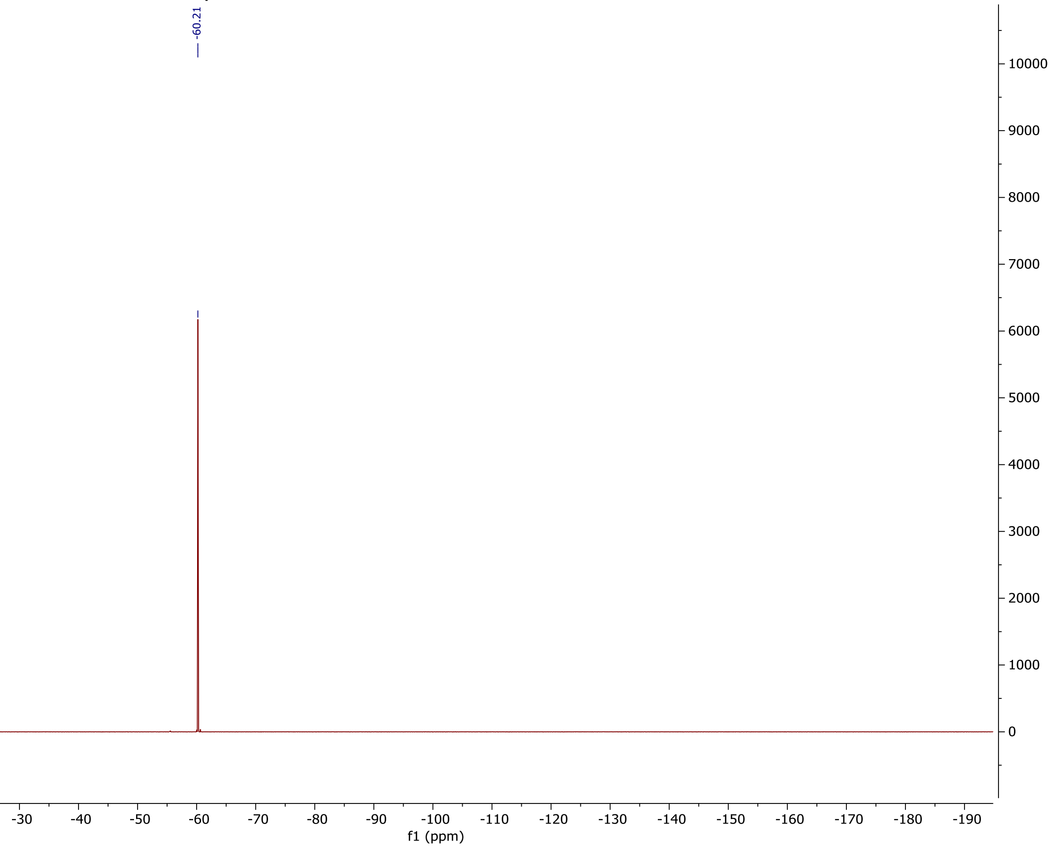

4-iodo-1-((4-nitrophenyl)sulfonyl)-1H-pyrrole-2-carbaldehyde 3(g)

^{1}H NMR (400 MHz, $CDCl_3$)

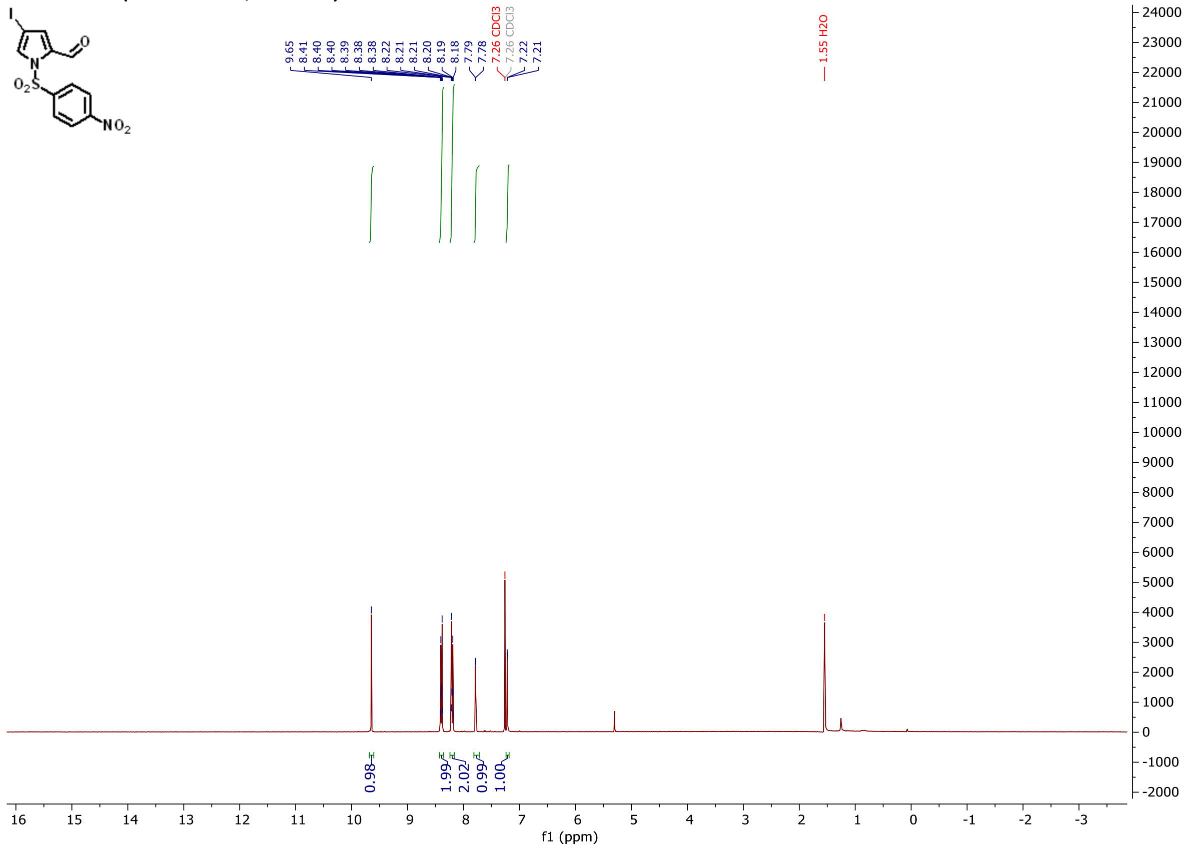

^{13}C{^{1}H} NMR (101 MHz, $CDCl_3$)

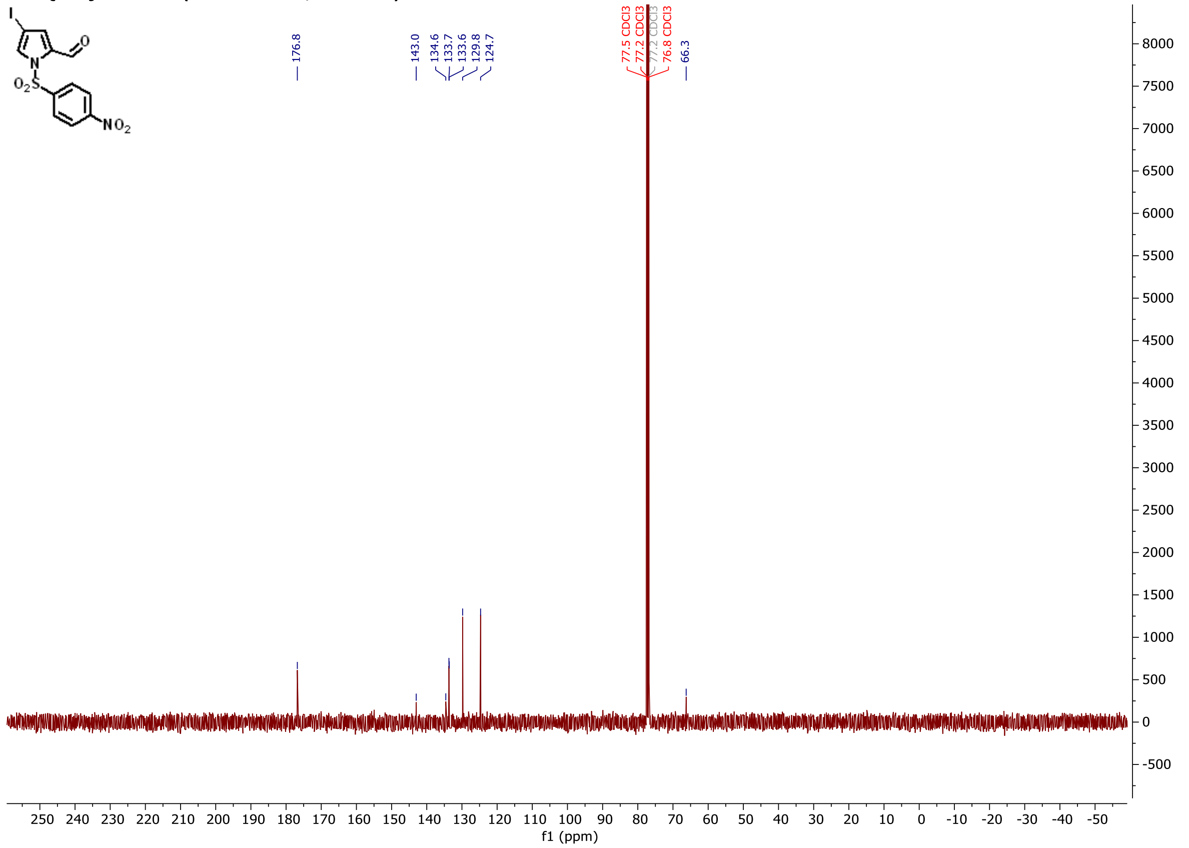

(4-nitrophenyl)(1H-pyrrol-2-yl)methanone (4a)

^{1}H NMR (400 MHz, $CDCl_3$)

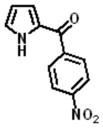

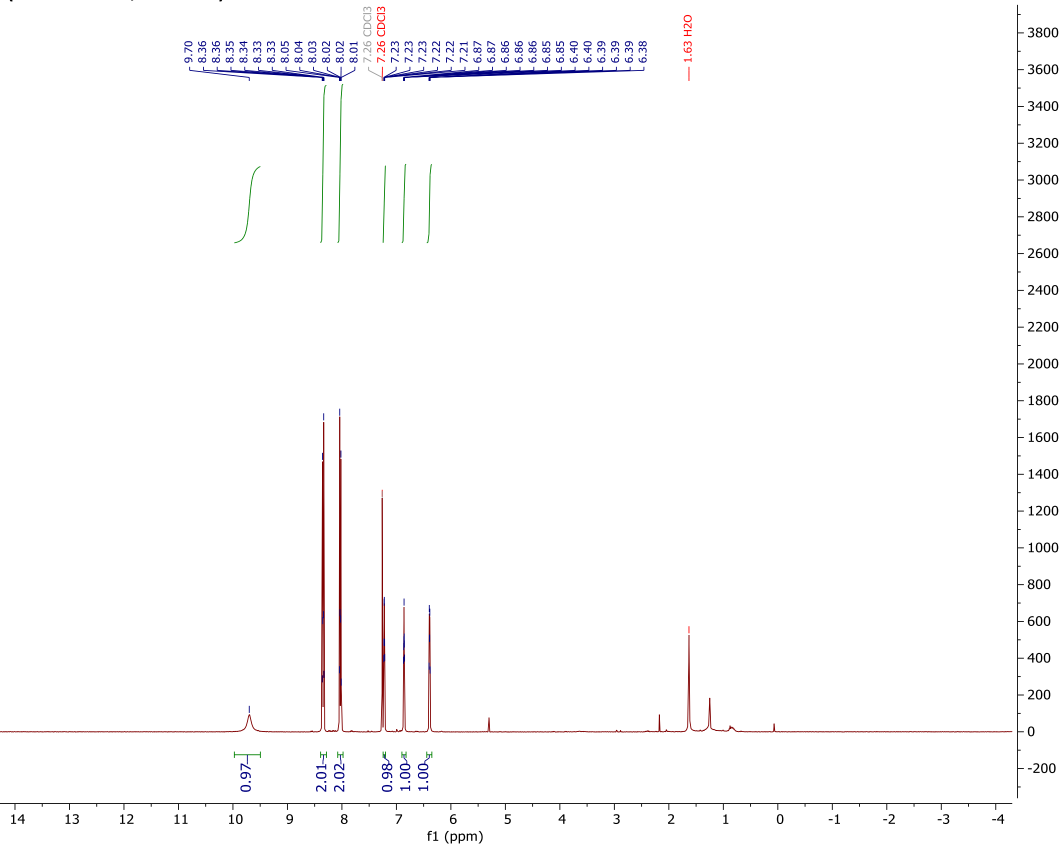

^{13}C{^{1}H} NMR (101 MHz, $CDCl_3$)

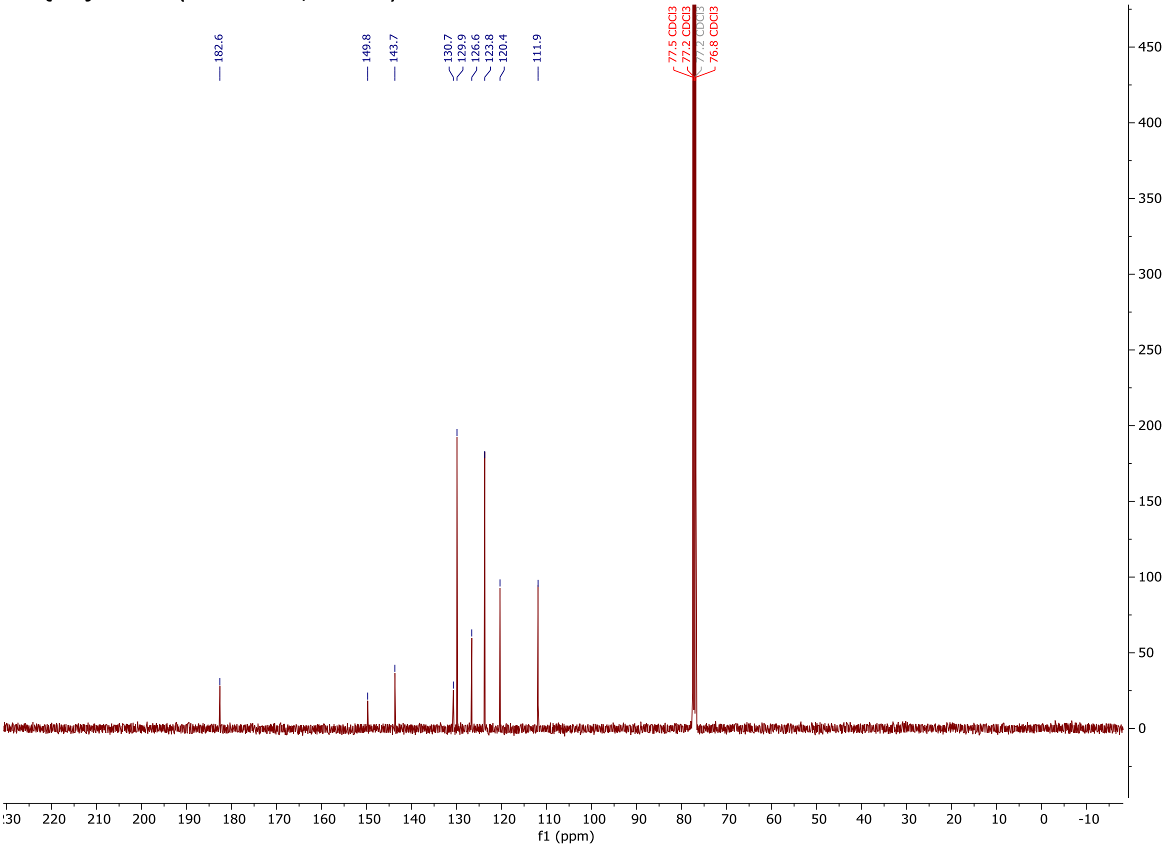

(2-nitrophenyl)(1H-pyrrol-2-yl)methanone (4b)

^{1}H NMR (500 MHz, $CDCl_3$)

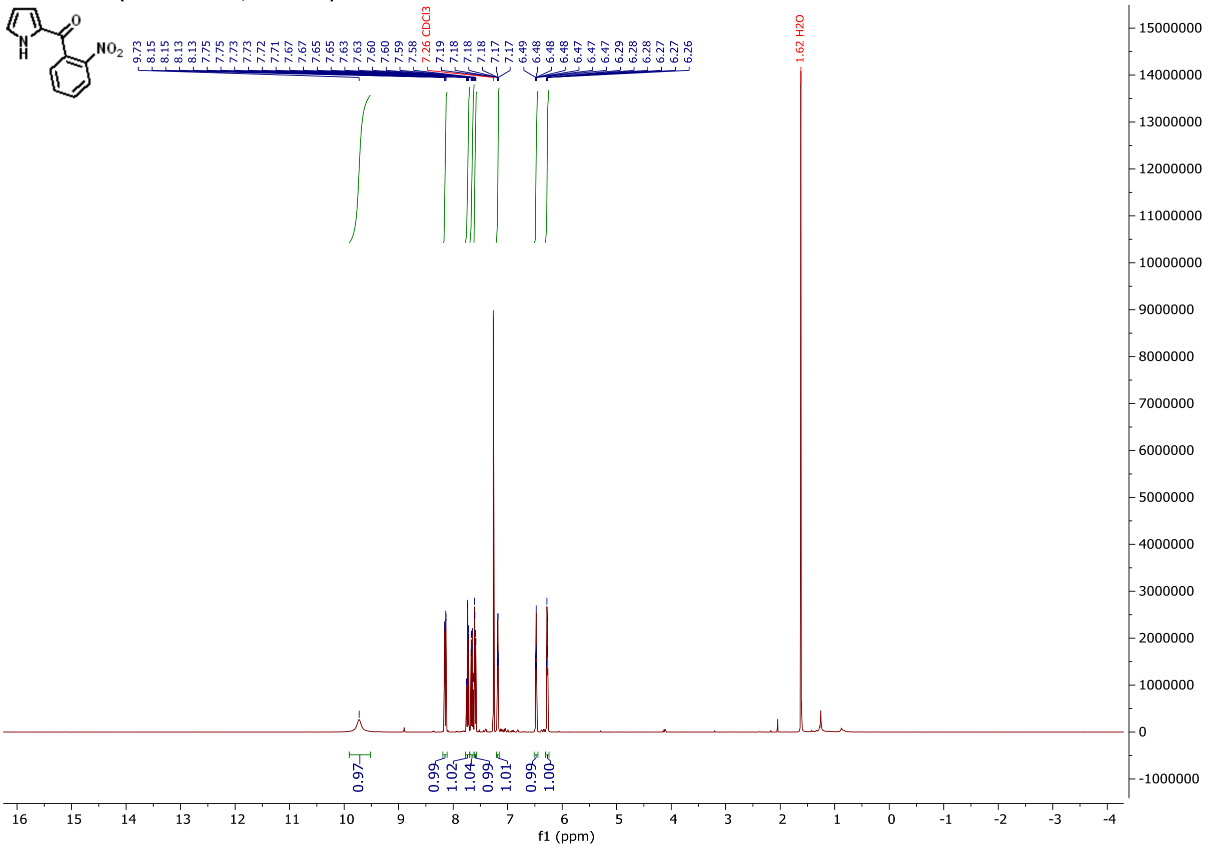

$^{13}C\{^{1}H\}$ NMR (126 MHz, $CDCl_3$)

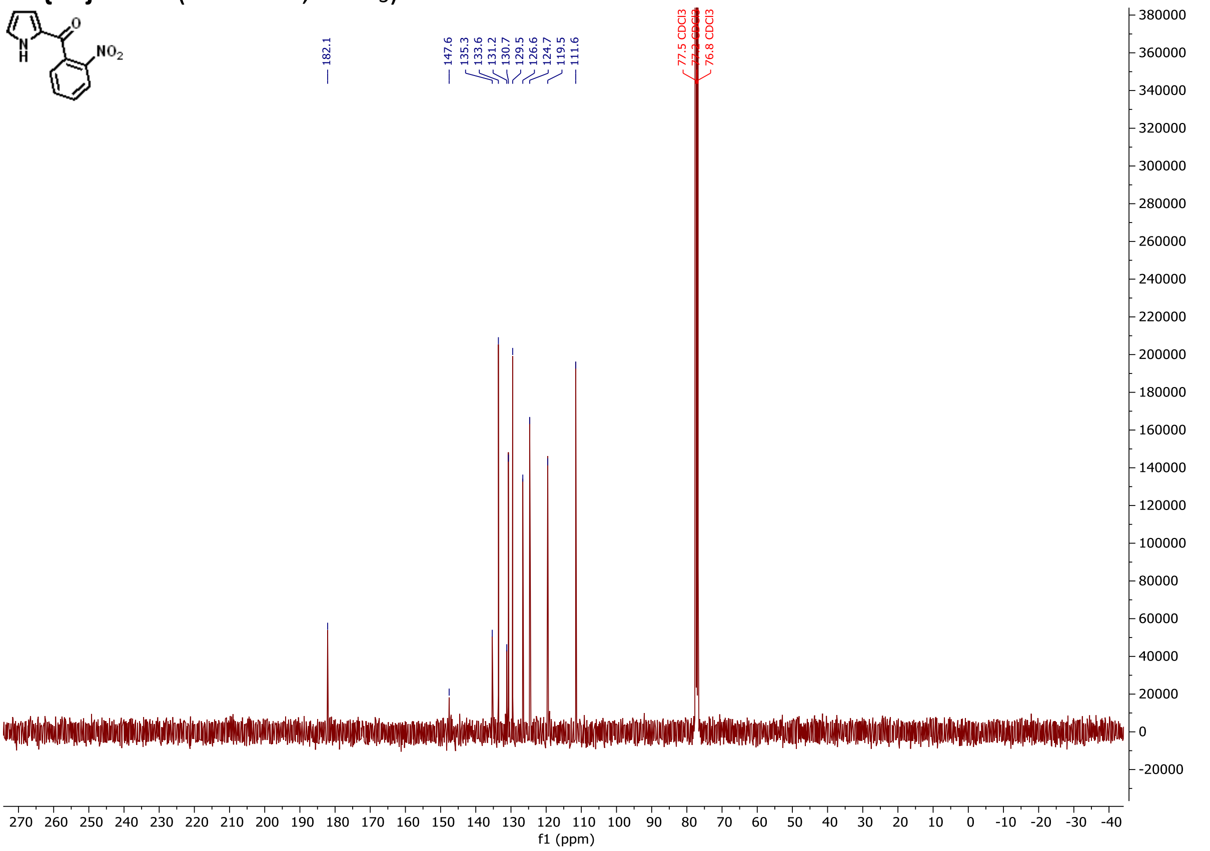

(5-fluoro-2-nitrophenyl)(1H-pyrrol-2-yl)methanone (4c)

¹H NMR (500 MHz, $CDCl_3$)

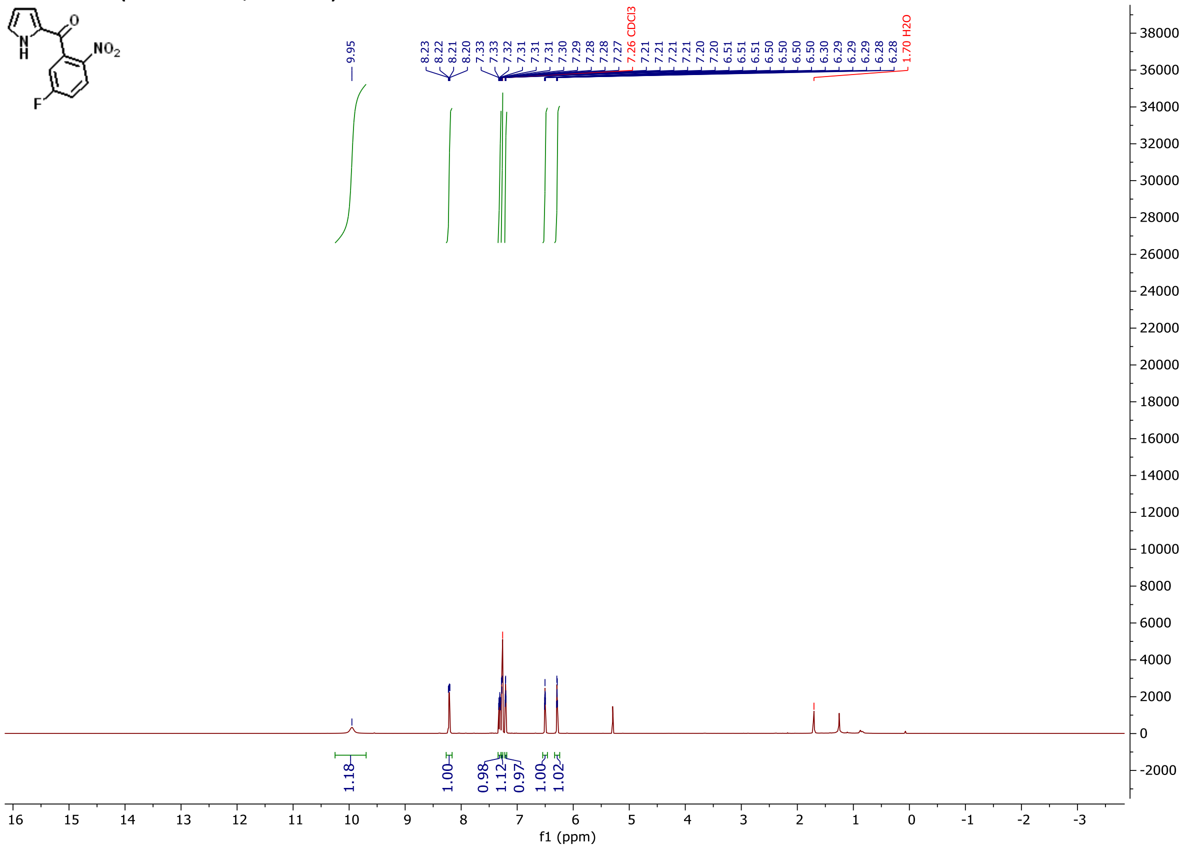

¹³C{¹H} NMR (126 MHz, $CDCl_3$)

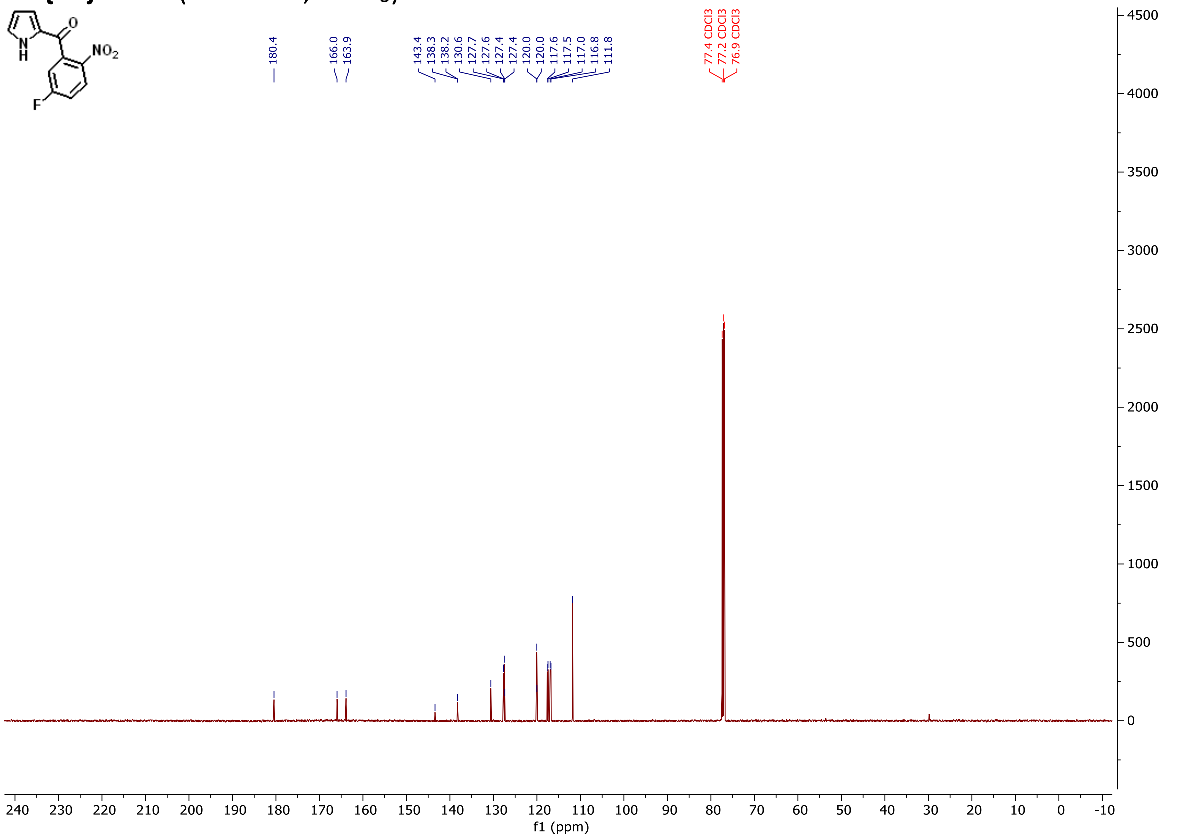

^{19}F NMR (471 MHz, $CDCl_3$)

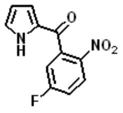

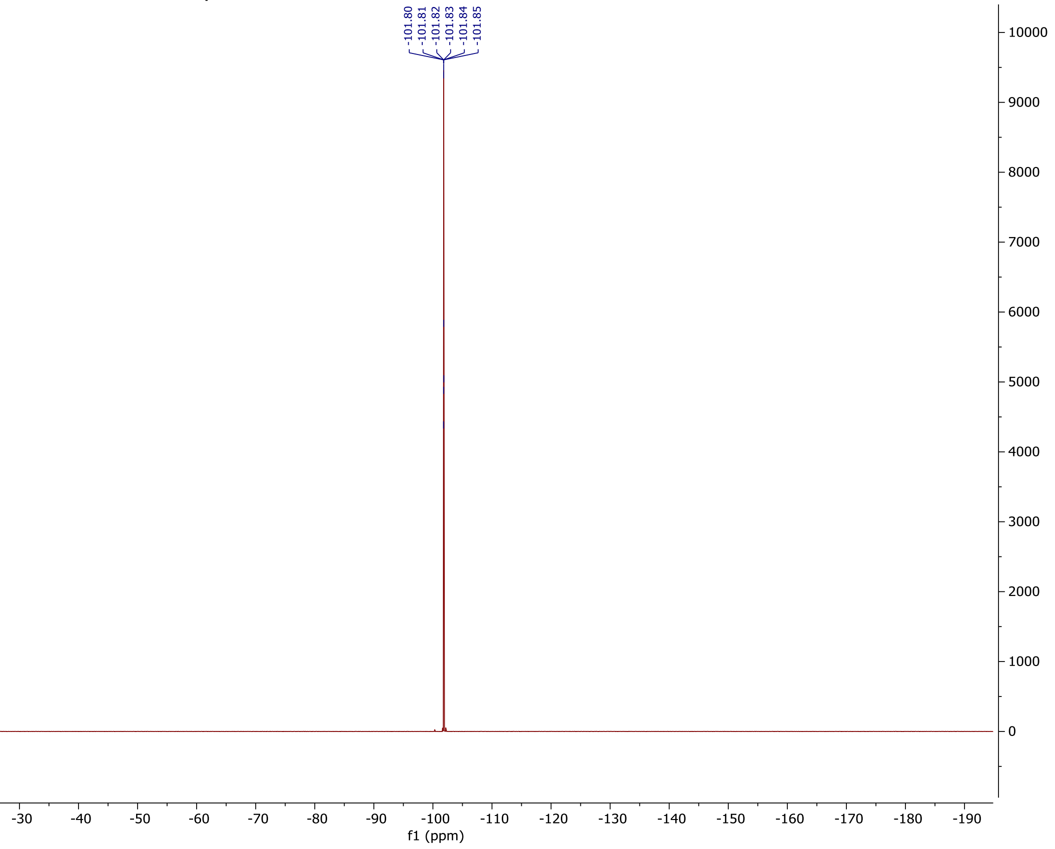

(4-bromo-2-nitrophenyl)(1H-pyrrol-2-yl)methanone (4d)

^{1}H NMR (400 MHz, $CDCl_3$)

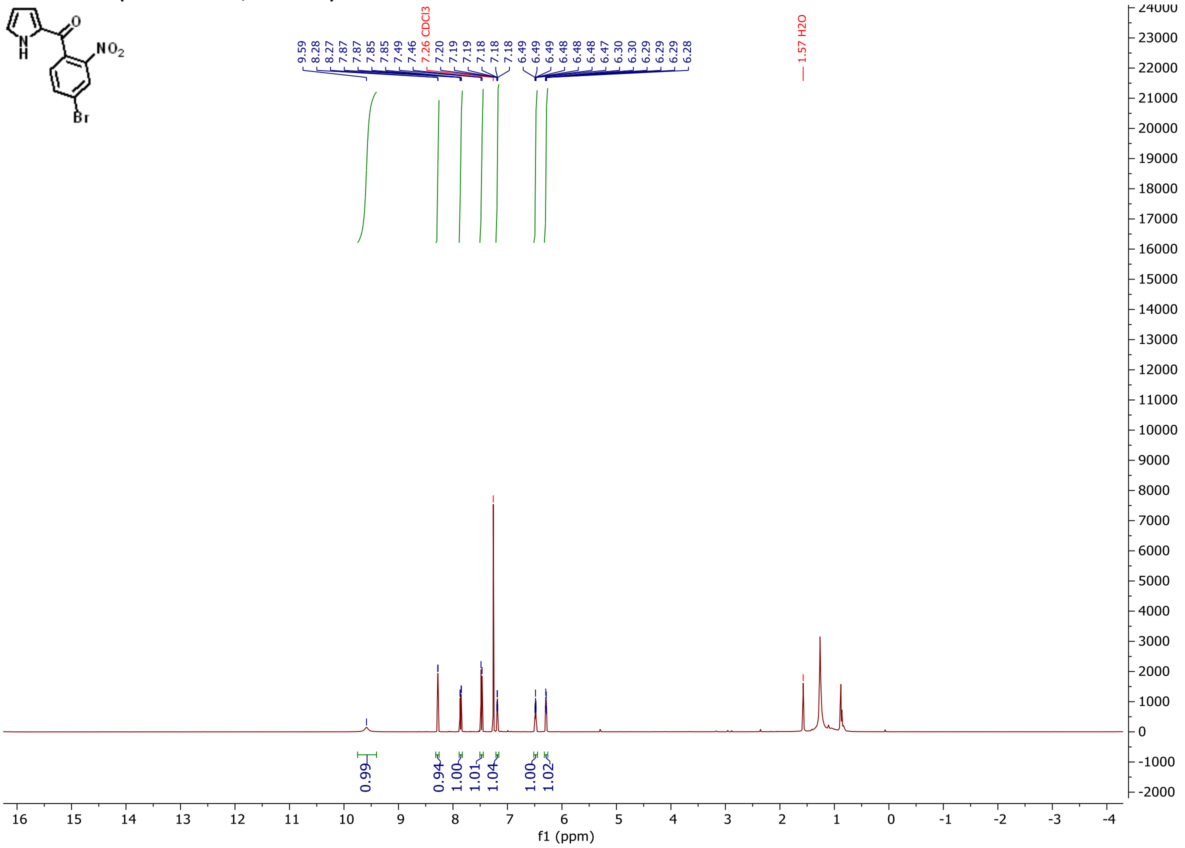

^{13}C{^{1}H} NMR (101 MHz, $CDCl_3$)

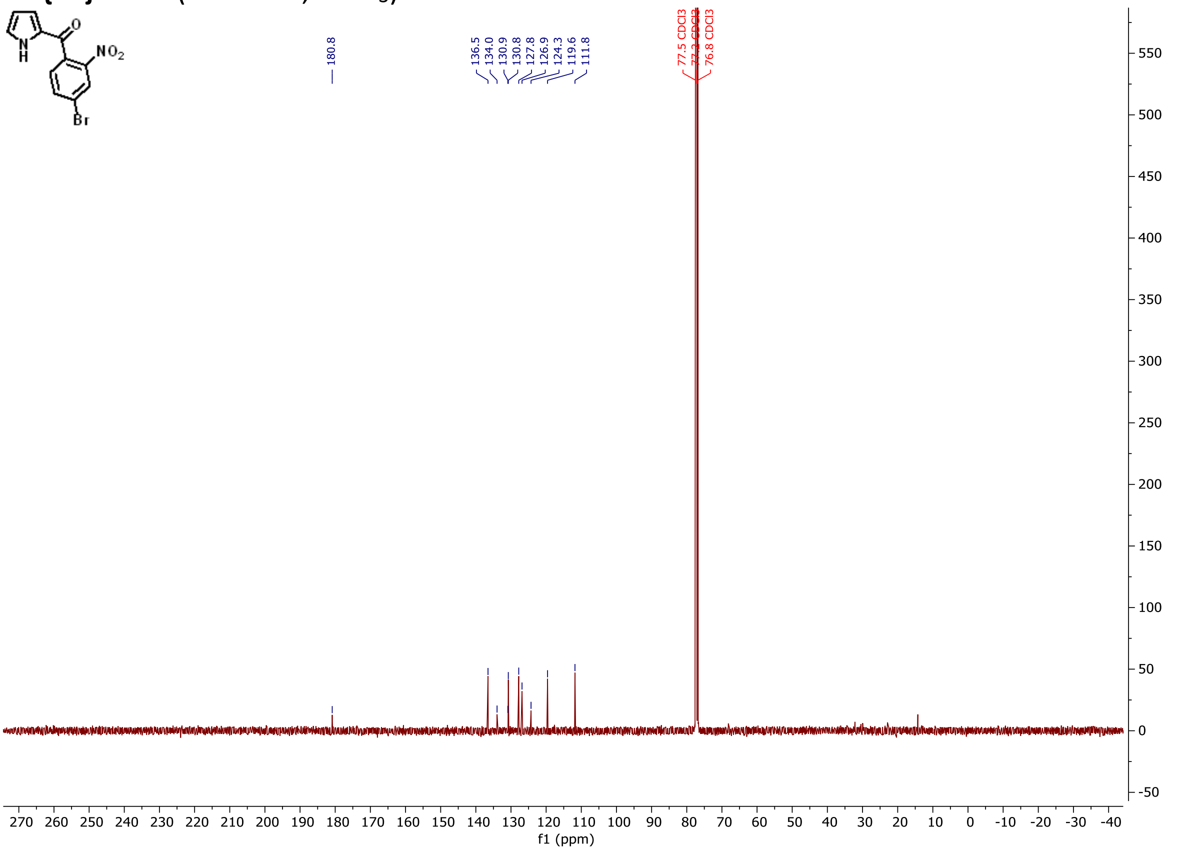

(2-fluoro-4-nitrophenyl)(1H-pyrrol-2-yl)methanone (4e)

^{1}H NMR (400 MHz, $CDCl_3$)

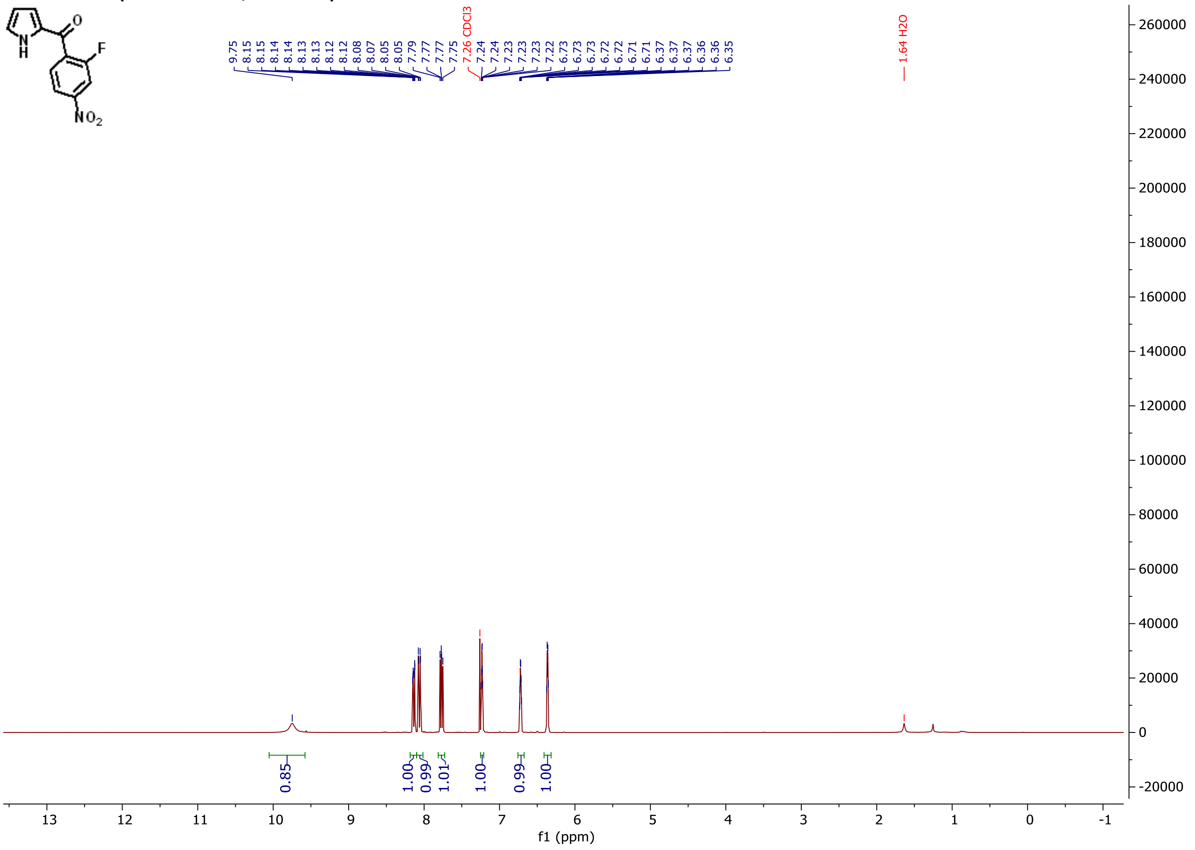

^{13}C{^{1}H} NMR (101 MHz, $CDCl_3$)

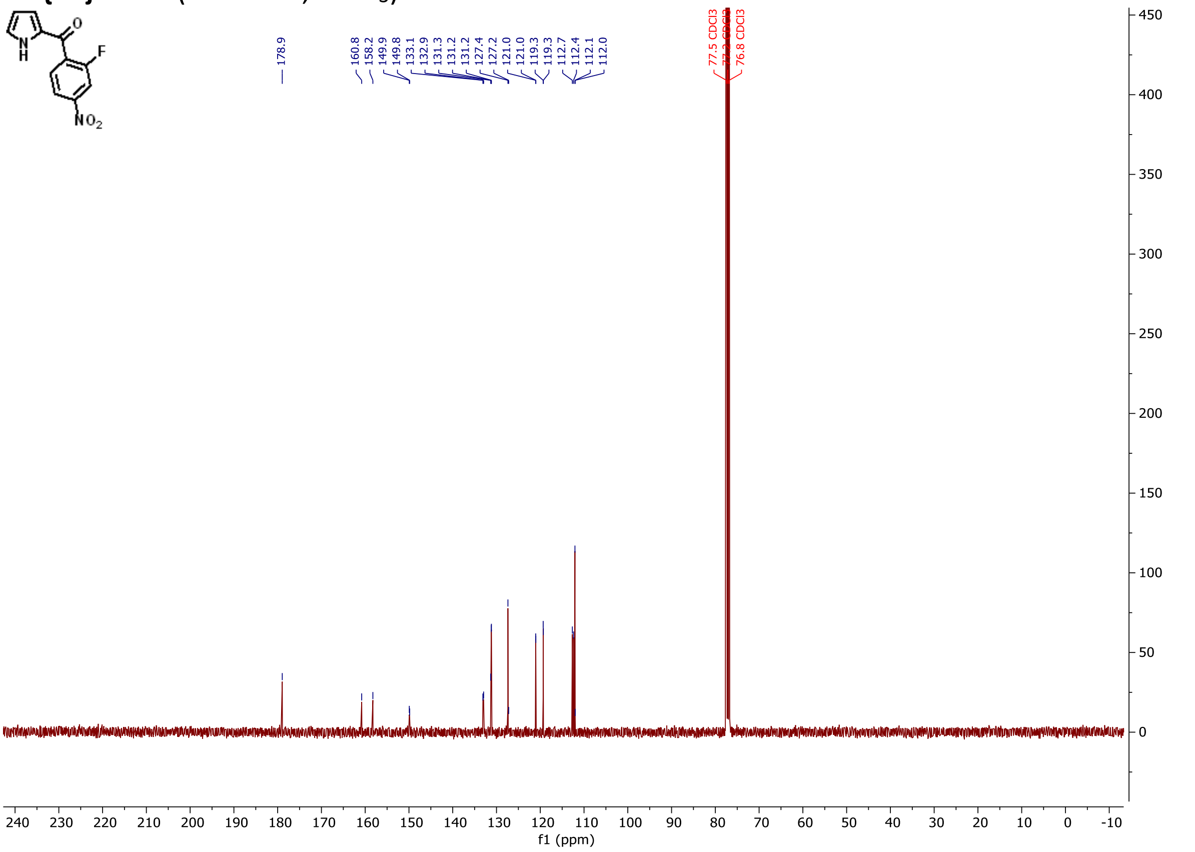

^{19}**F NMR** (471 MHz, $CDCl_3$)

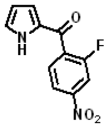

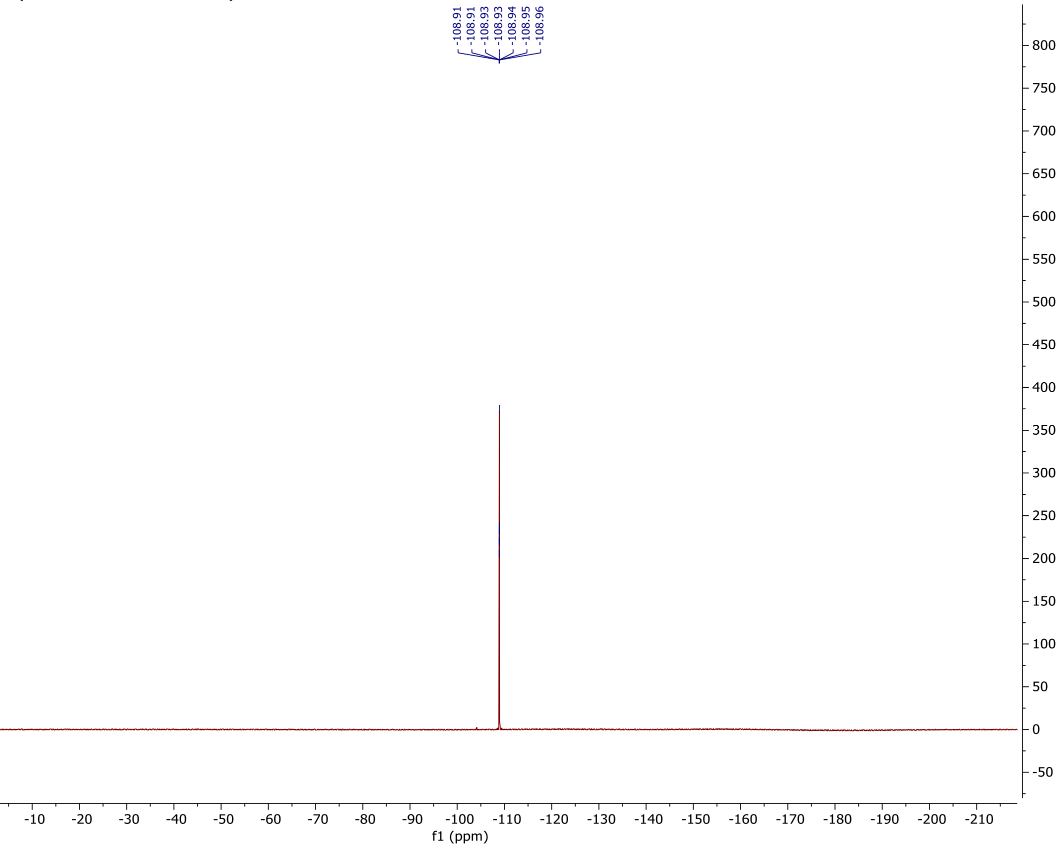

(4-nitro-3-(trifluoromethyl)phenyl)(1H-pyrrol-2-yl)methanone (4f)

^{1}H NMR (400 MHz, $CDCl_3$)

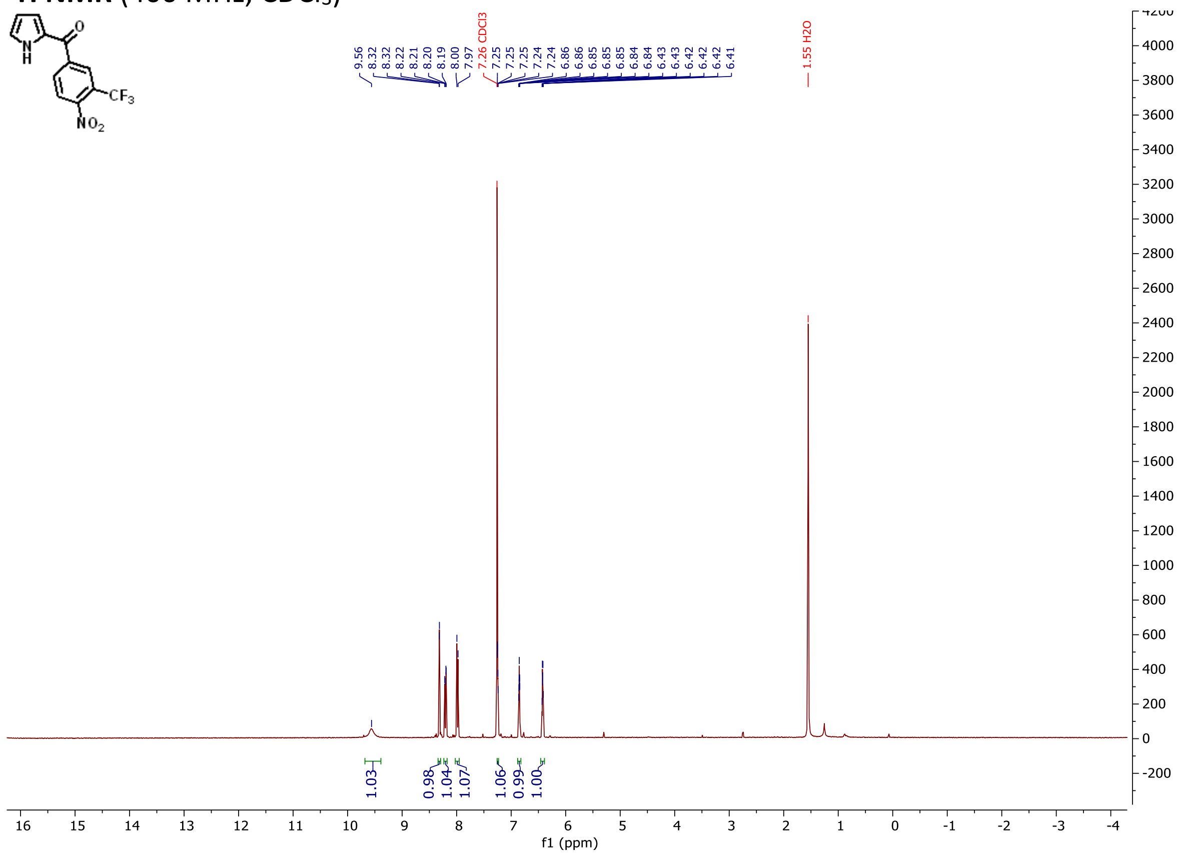

$^{13}C\{^1H\}$ NMR (101 MHz, $CDCl_3$)

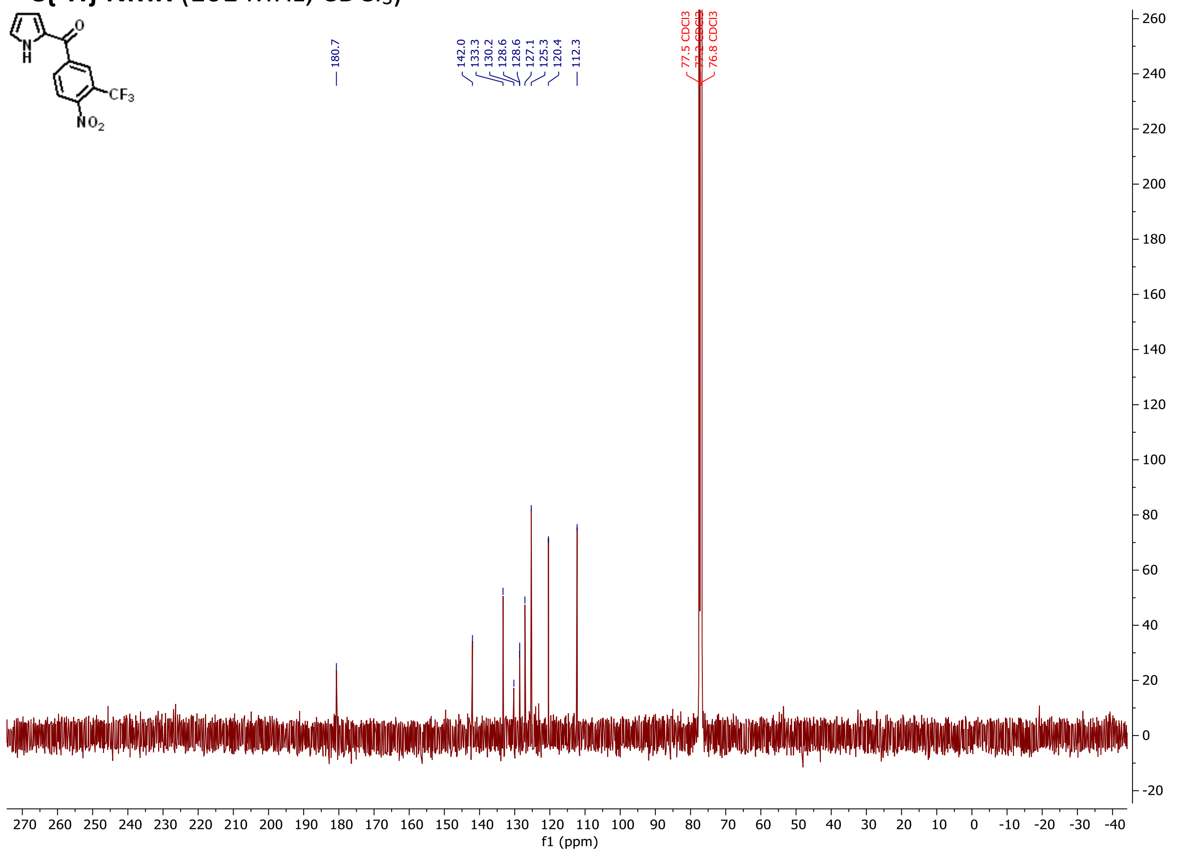

^{19}F NMR (471 MHz, $CDCl_3$)

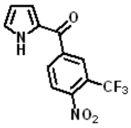

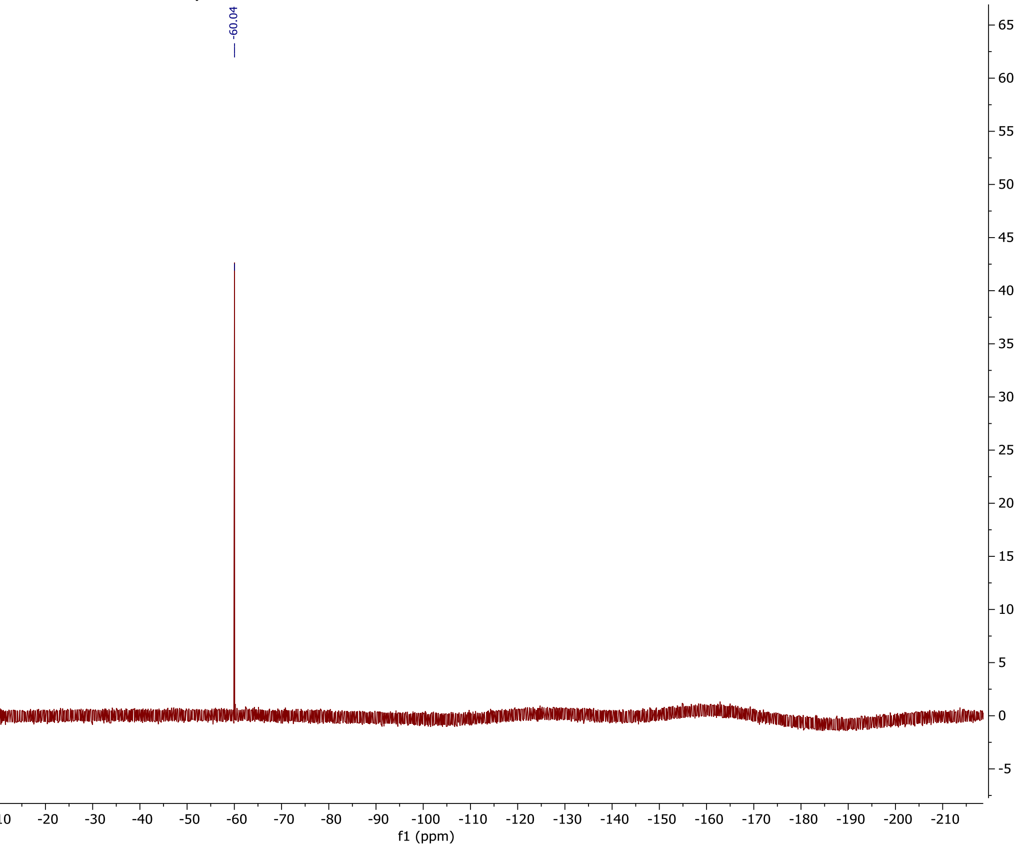

(4-iodo-1H-pyrrol-2-yl)(4-nitrophenyl)methanone (4g)

^{1}H NMR (500 MHz, $(CD_3)_2CO$)

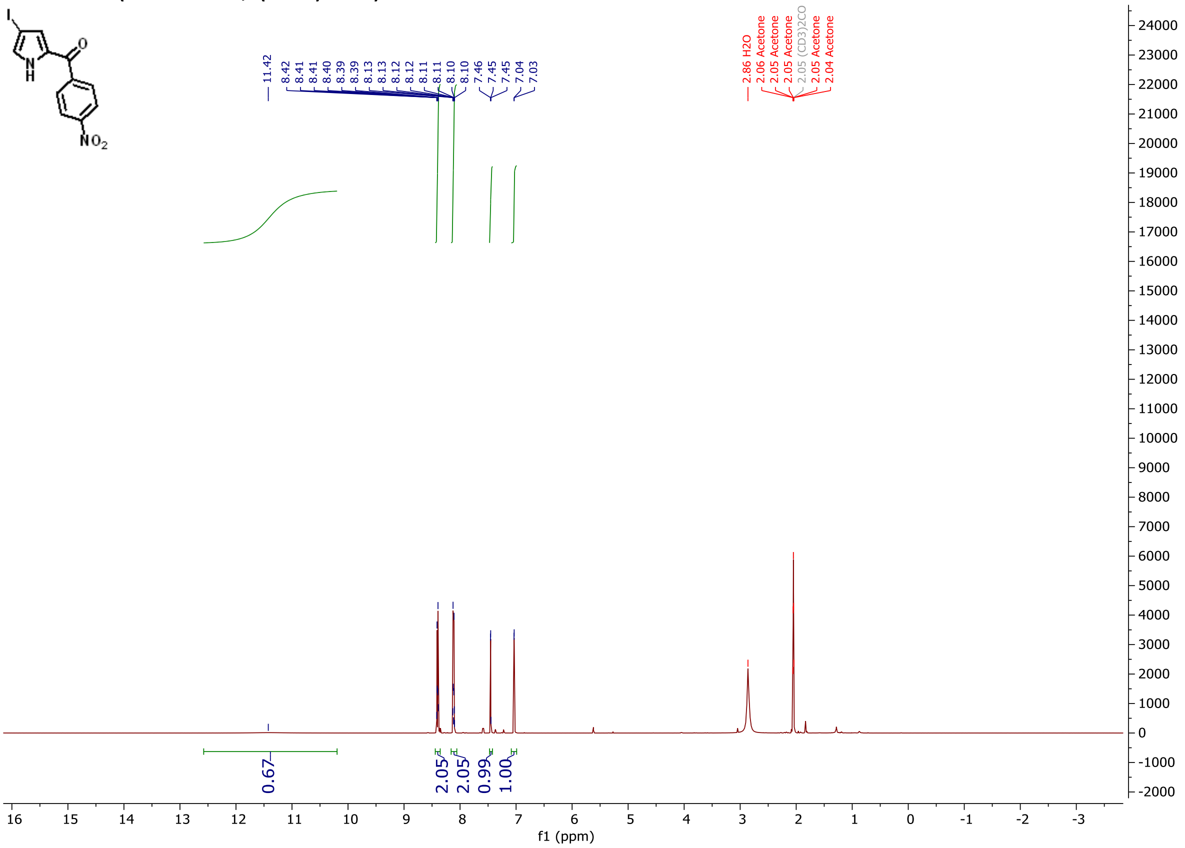

$^{13}C\{^{1}H\}$ NMR (126 MHz, $(CD_3)_2CO$)

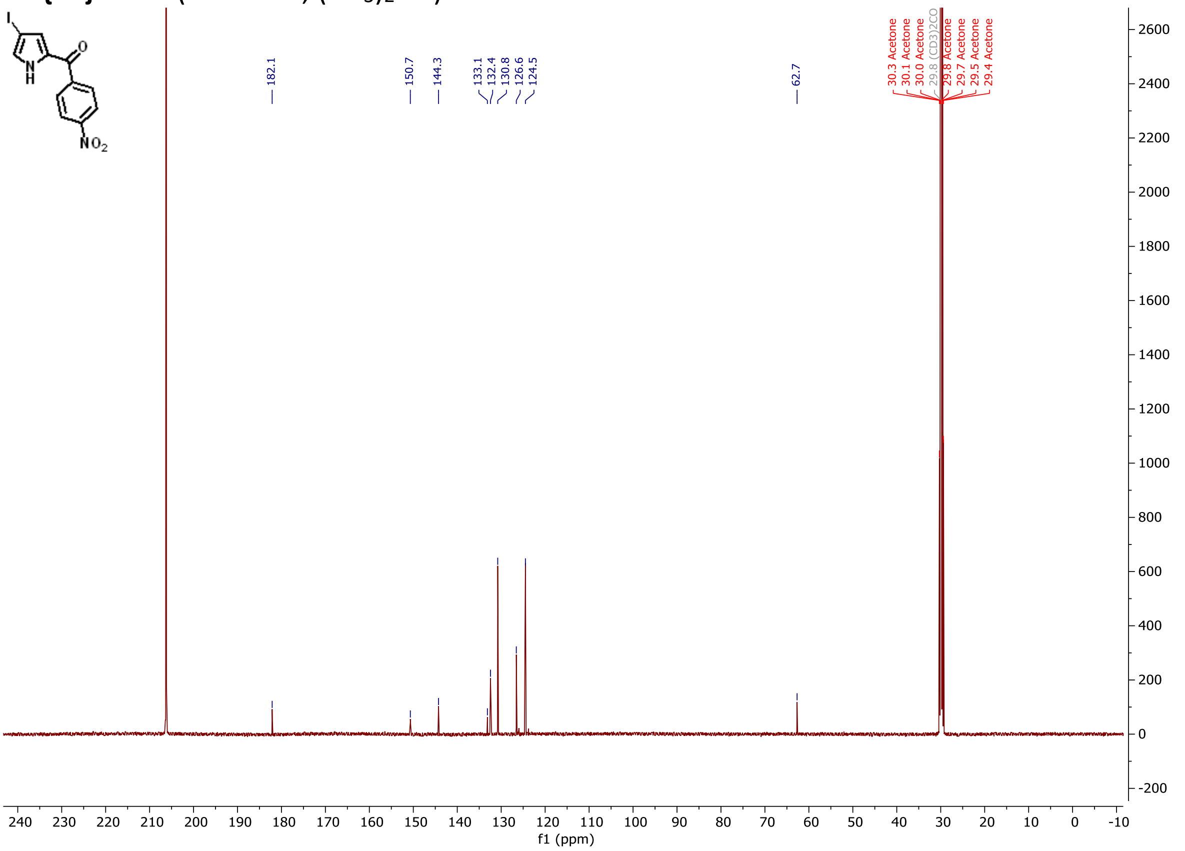

(3-bromo-1*H*-indol-2-yl)(4-nitrophenyl)methanone (5)

^{1}H NMR (400 MHz, $CDCl_3$)

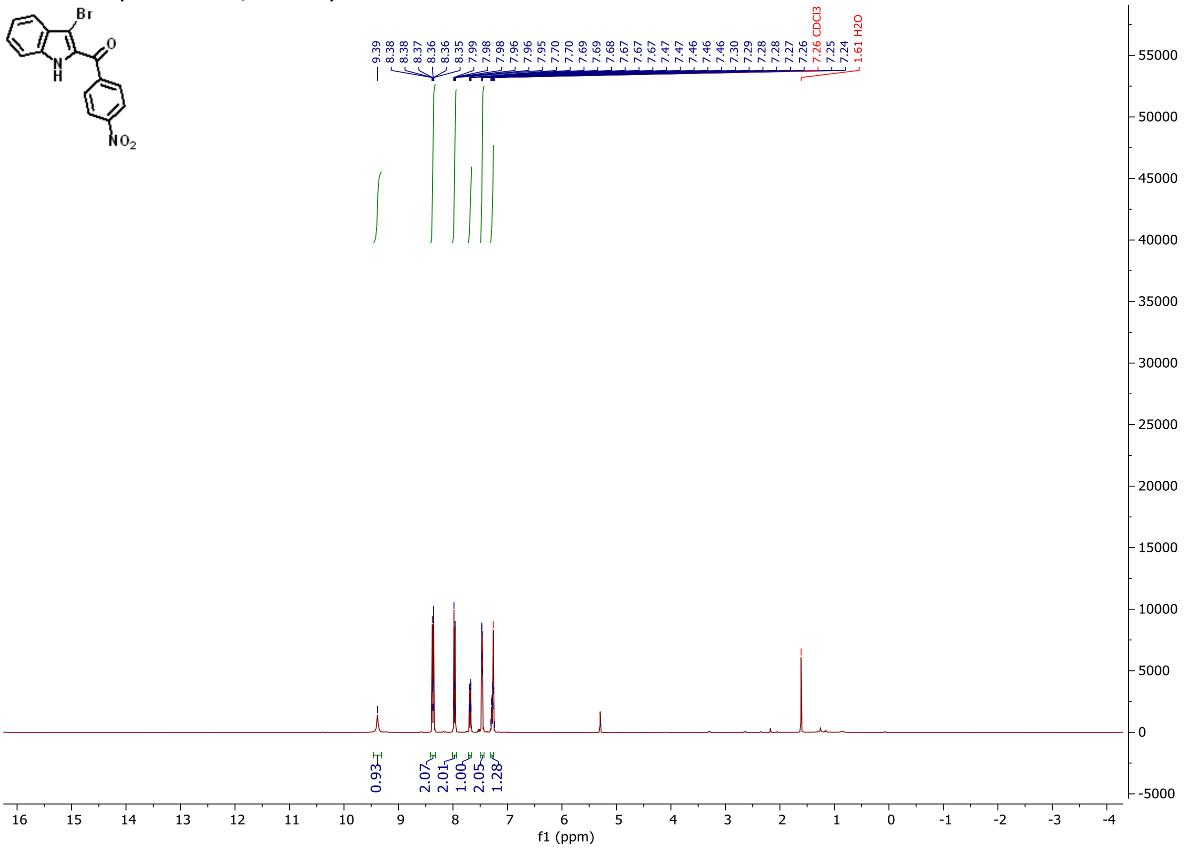

^{13}C{^{1}H} NMR (101 MHz, $CDCl_3$)

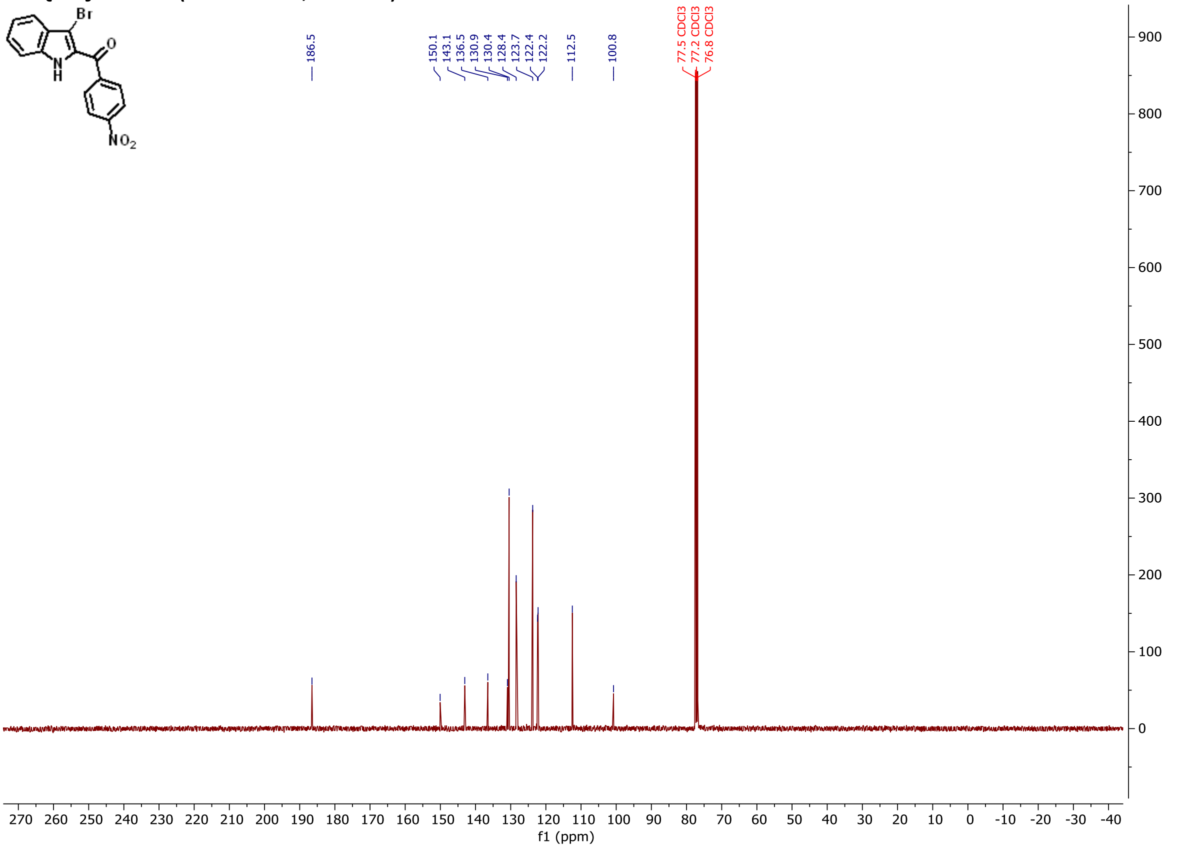

(1H-indol-2-yl)(4-nitrophenyl)methanol (6)

^{1}**H NMR** (400 MHz, $CDCl_3$)

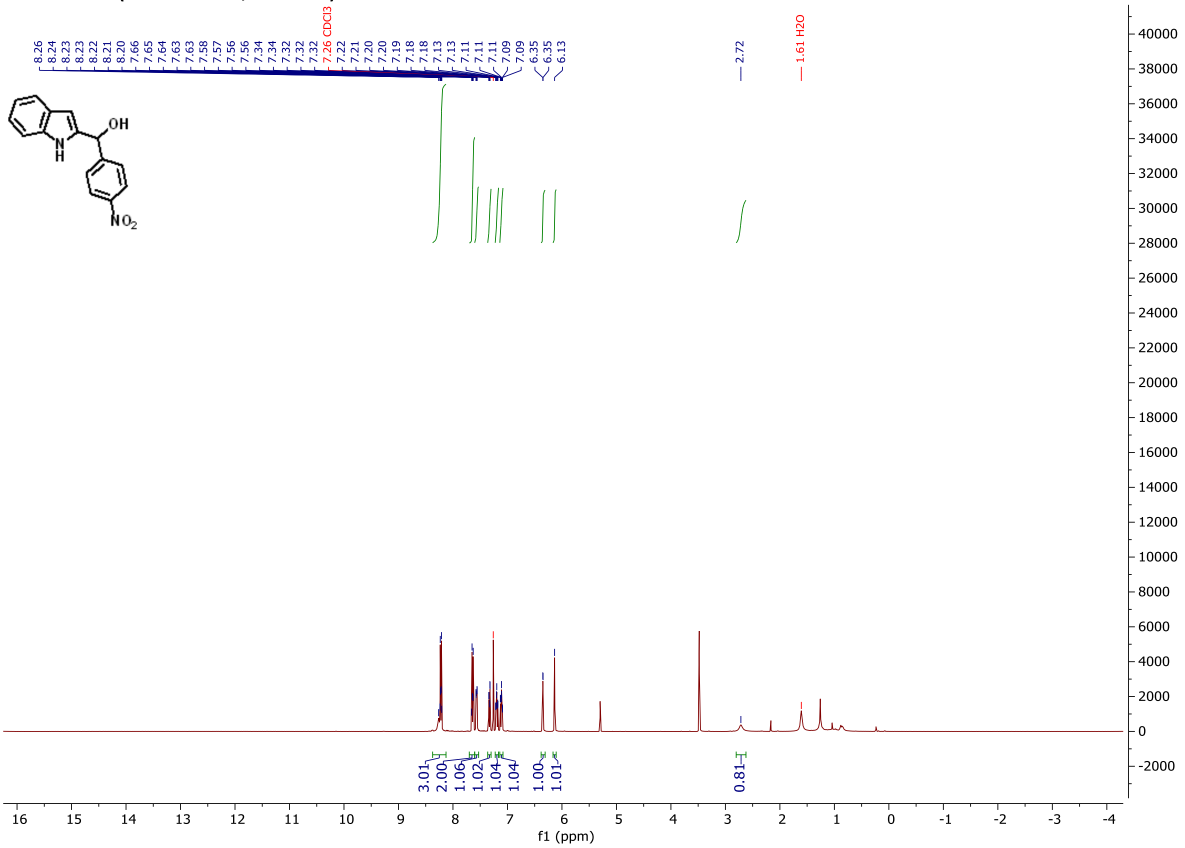

^{13}**C{^{1}H} NMR** (101 MHz, $CDCl_3$)

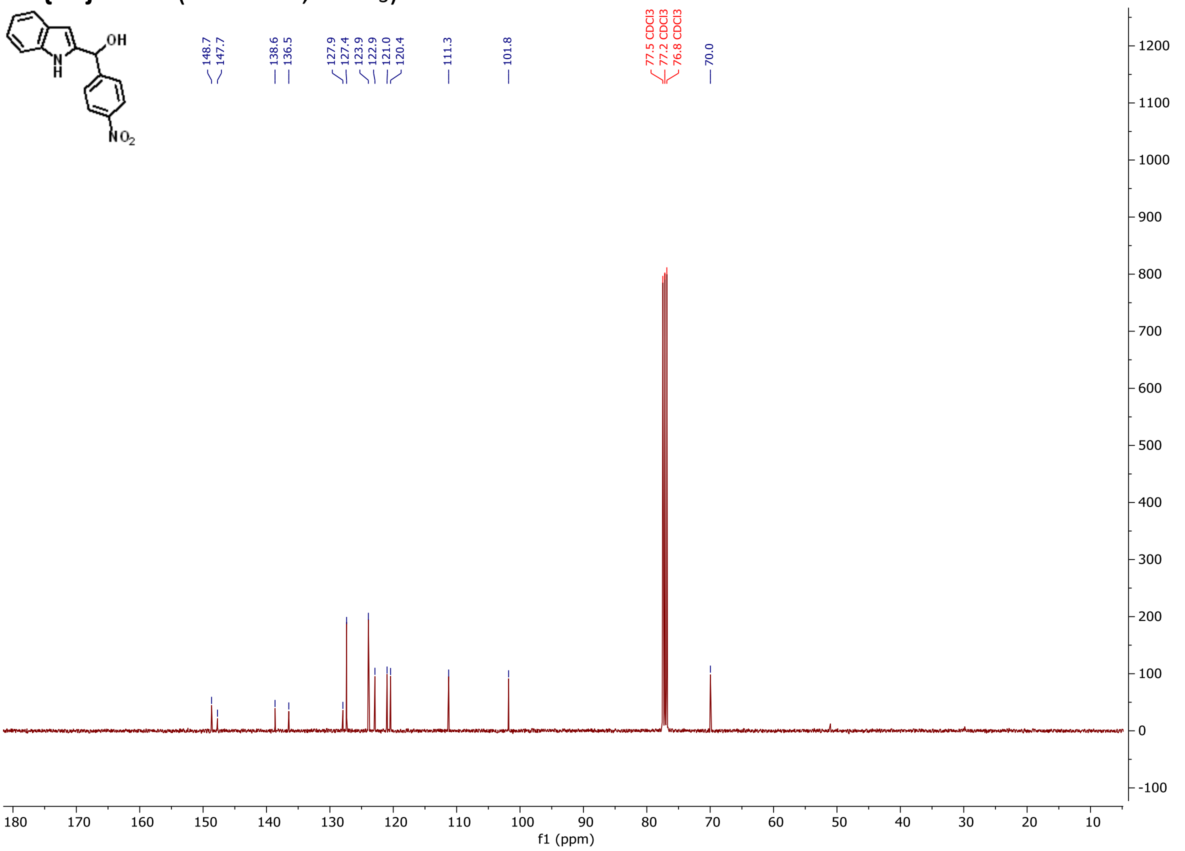

(1-methyl-1H-indol-2-yl)(4-nitrophenyl)methanone (7a)

^{1}H NMR (500 MHz, $CDCl_3$)

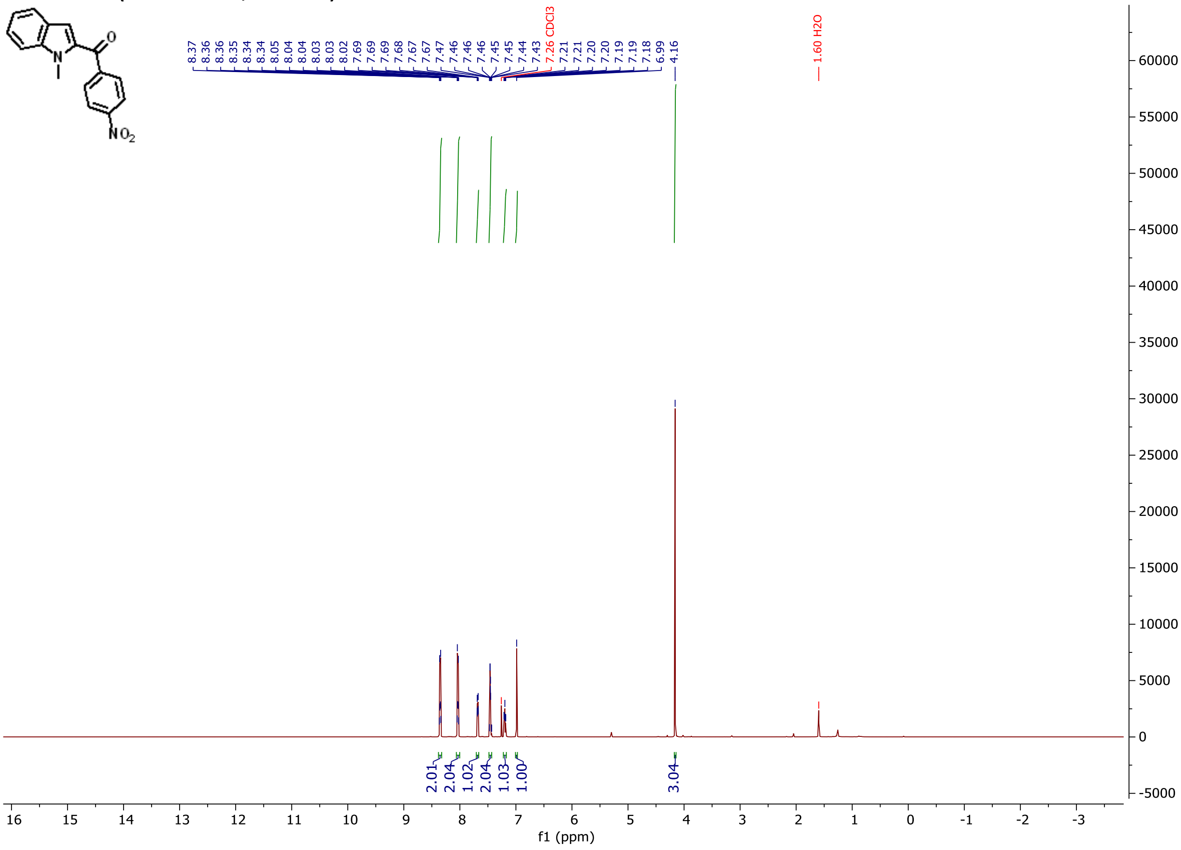

^{13}C{^{1}H} NMR (126 MHz, $CDCl_3$)

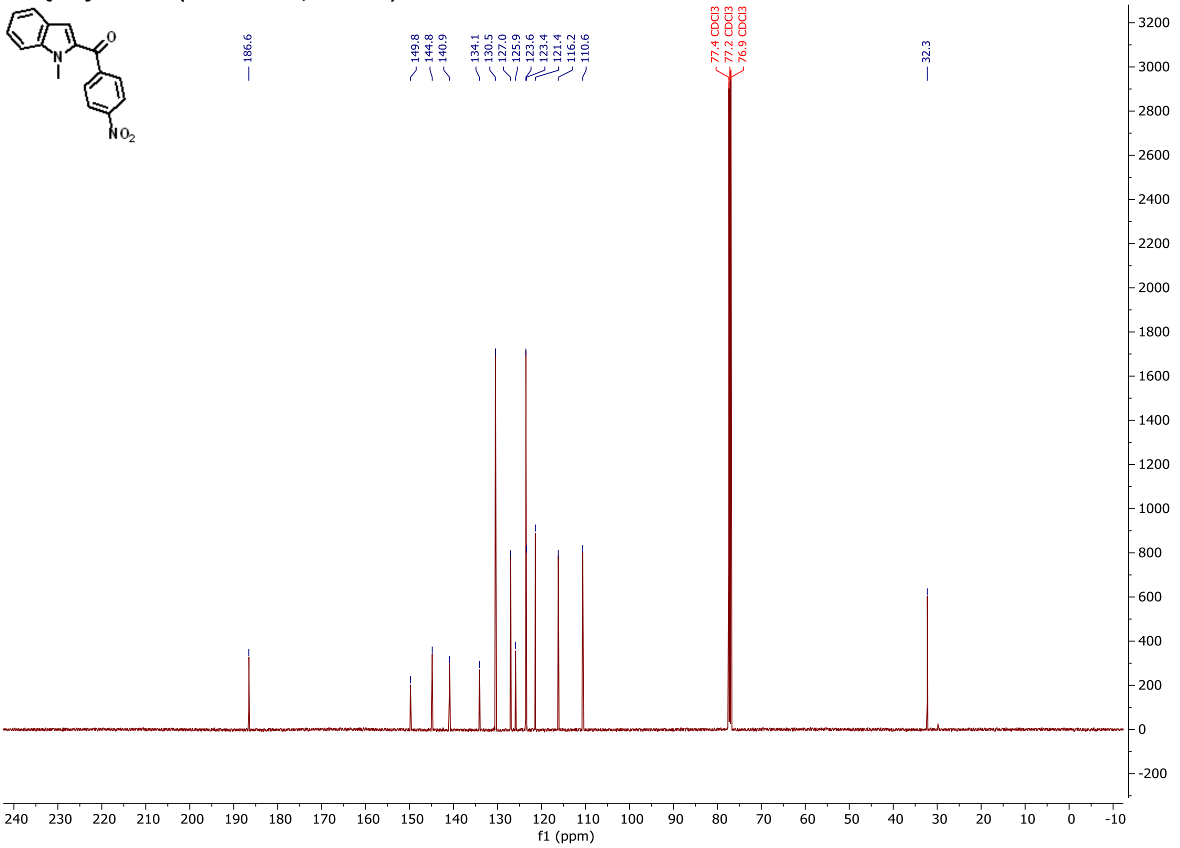

(1-methyl-1H-pyrrol-2-yl)(4-nitrophenyl)methanone (7b)

^{1}H NMR (400 MHz, $CDCl_3$)

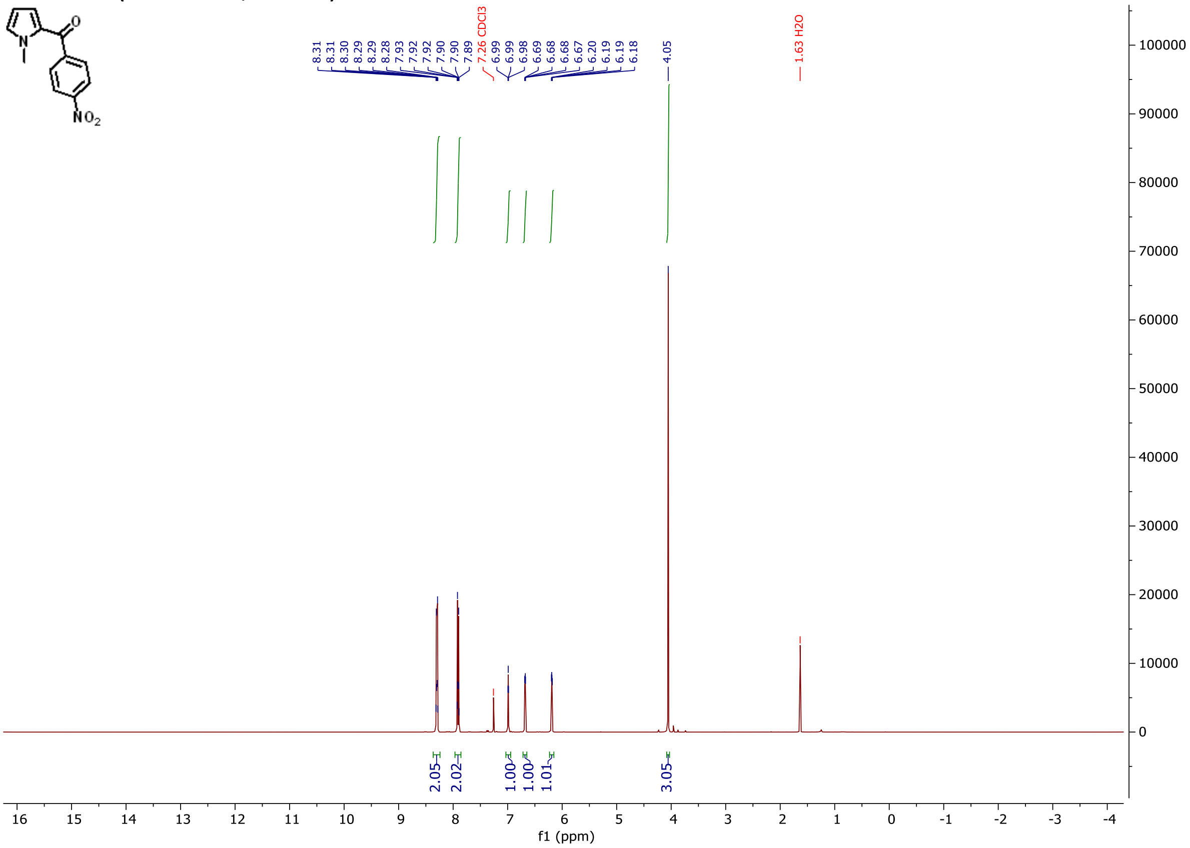

^{13}C{^{1}H} NMR (101 MHz, $CDCl_3$)

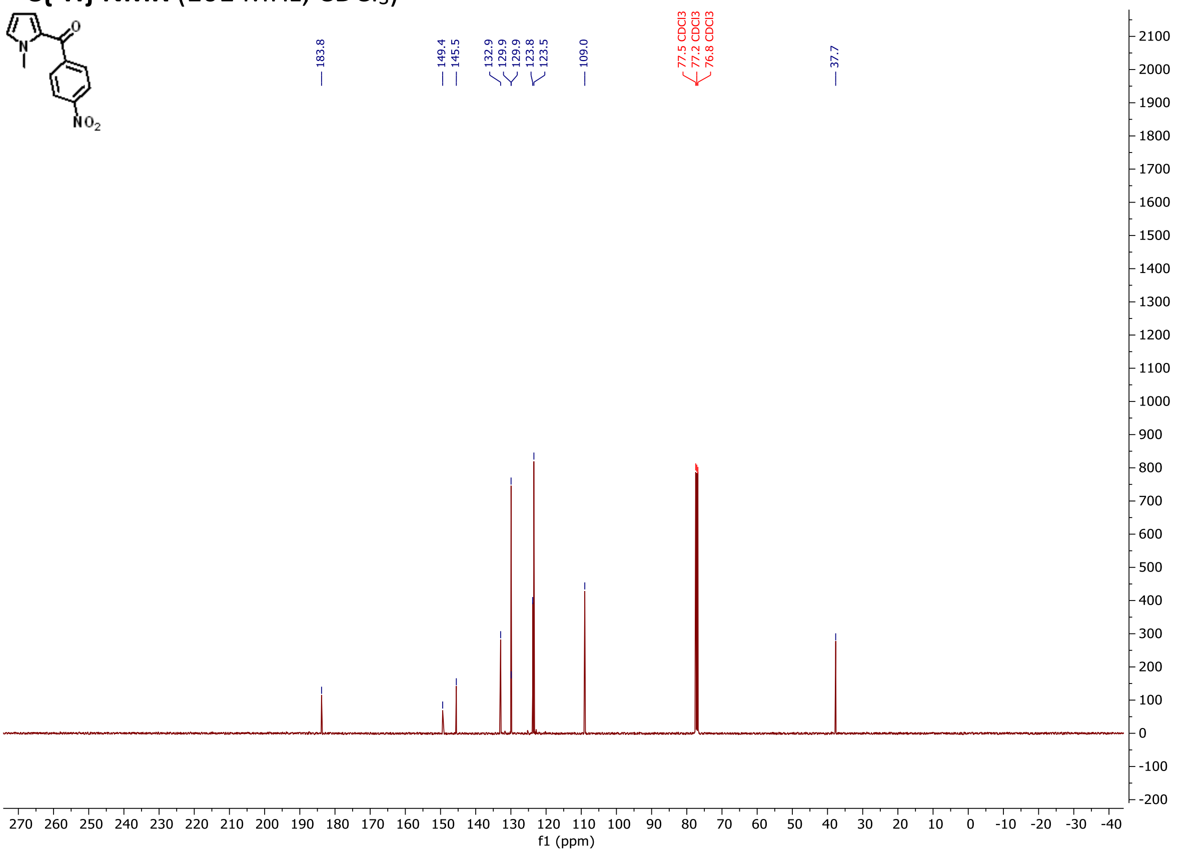

(4-aminophenyl)(1H-indol-2-yl)methanone (8a)

^{1}H NMR (500 MHz, $CDCl_3$)

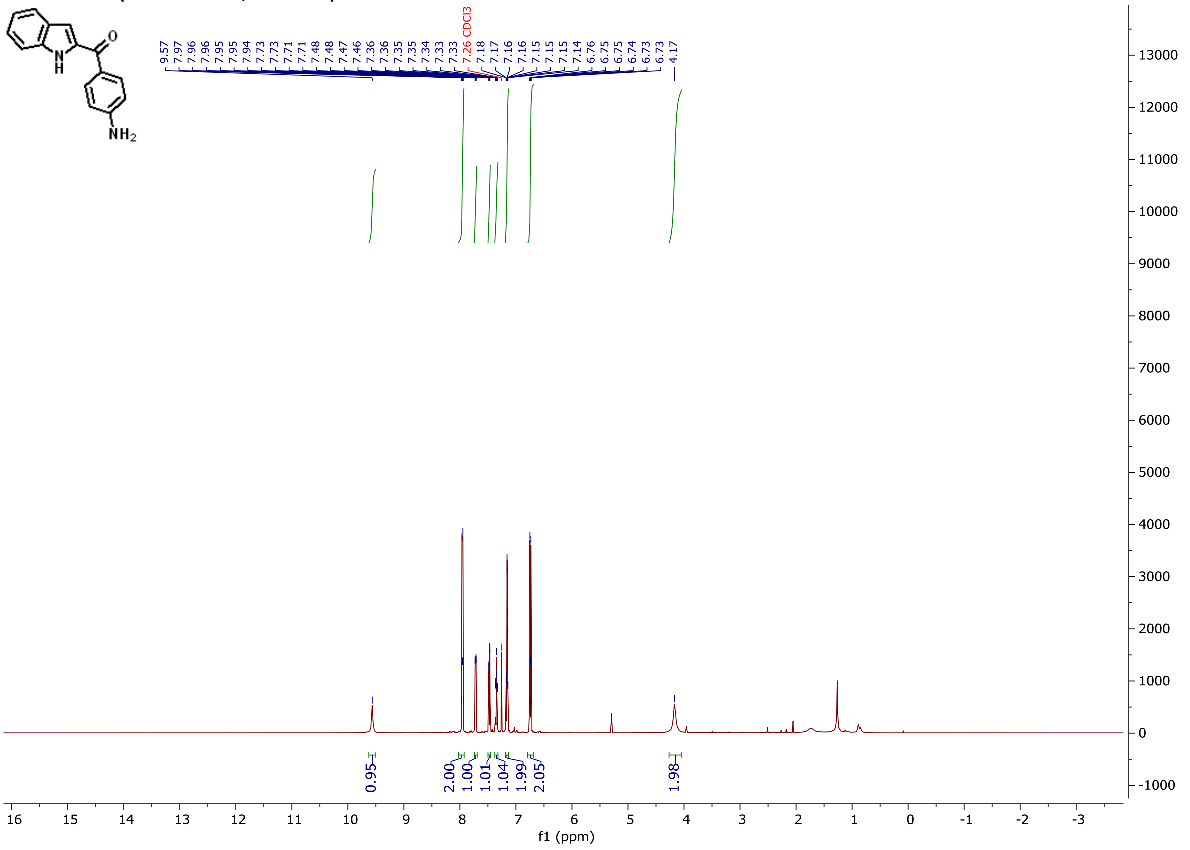

$^{13}C\{^{1}H\}$ NMR (126 MHz, $CDCl_3$)

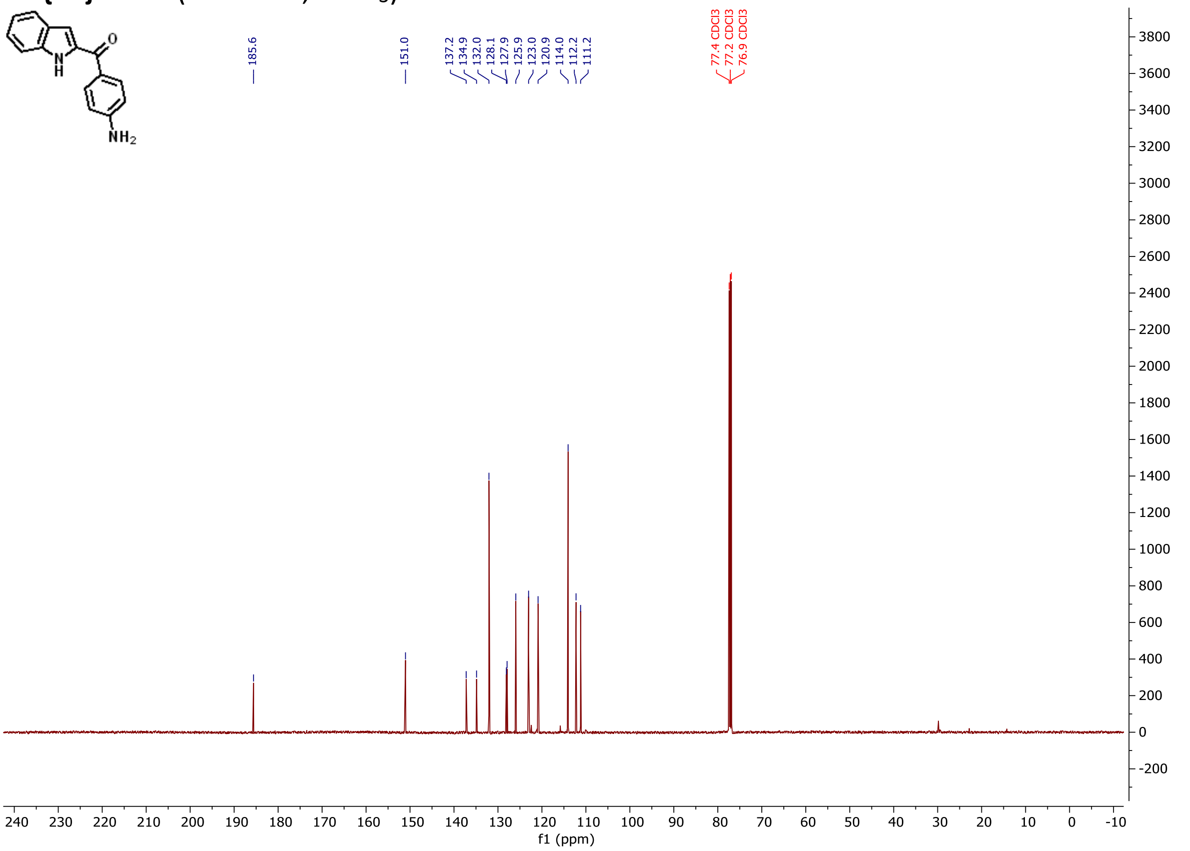

(4-aminophenyl)(1H-pyrrol-2-yl)methanone (8b)

^{1}H NMR (400 MHz, $CDCl_3$)

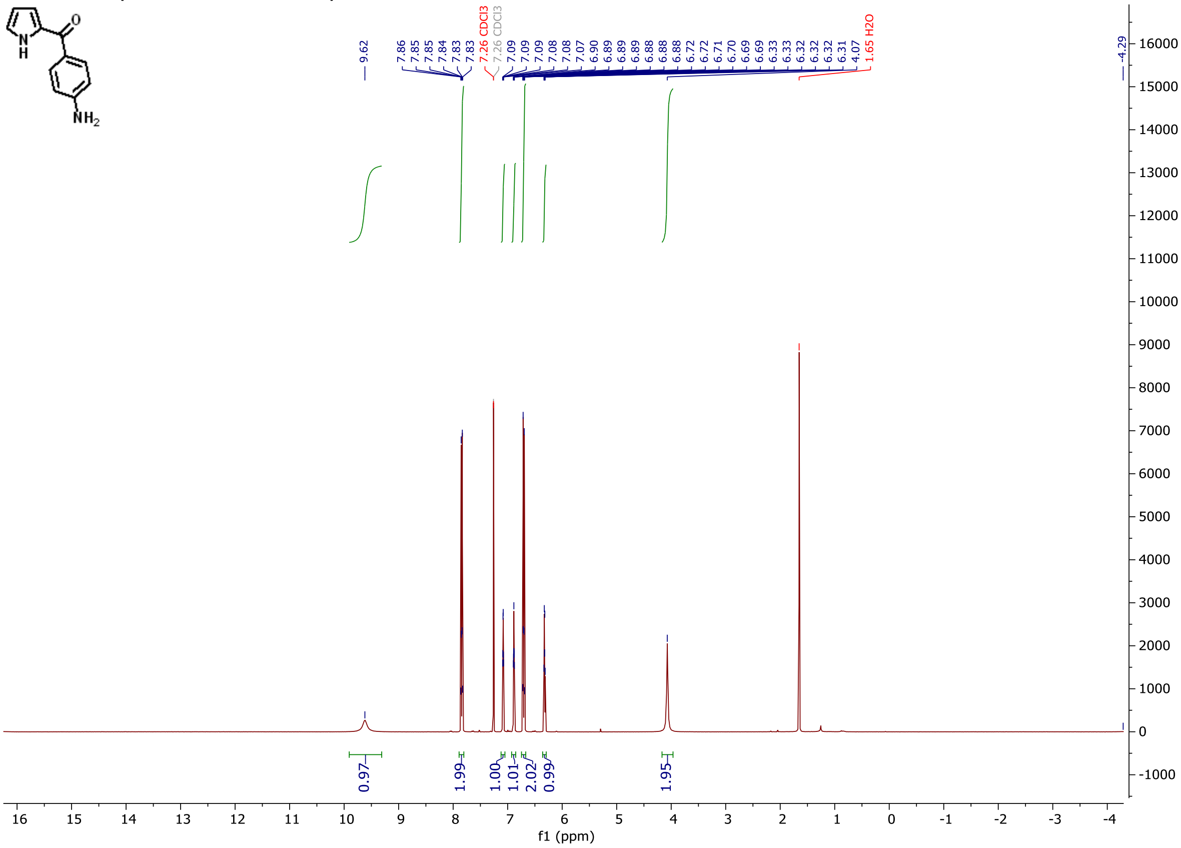

^{13}C{^{1}H} NMR (101 MHz, $CDCl_3$)

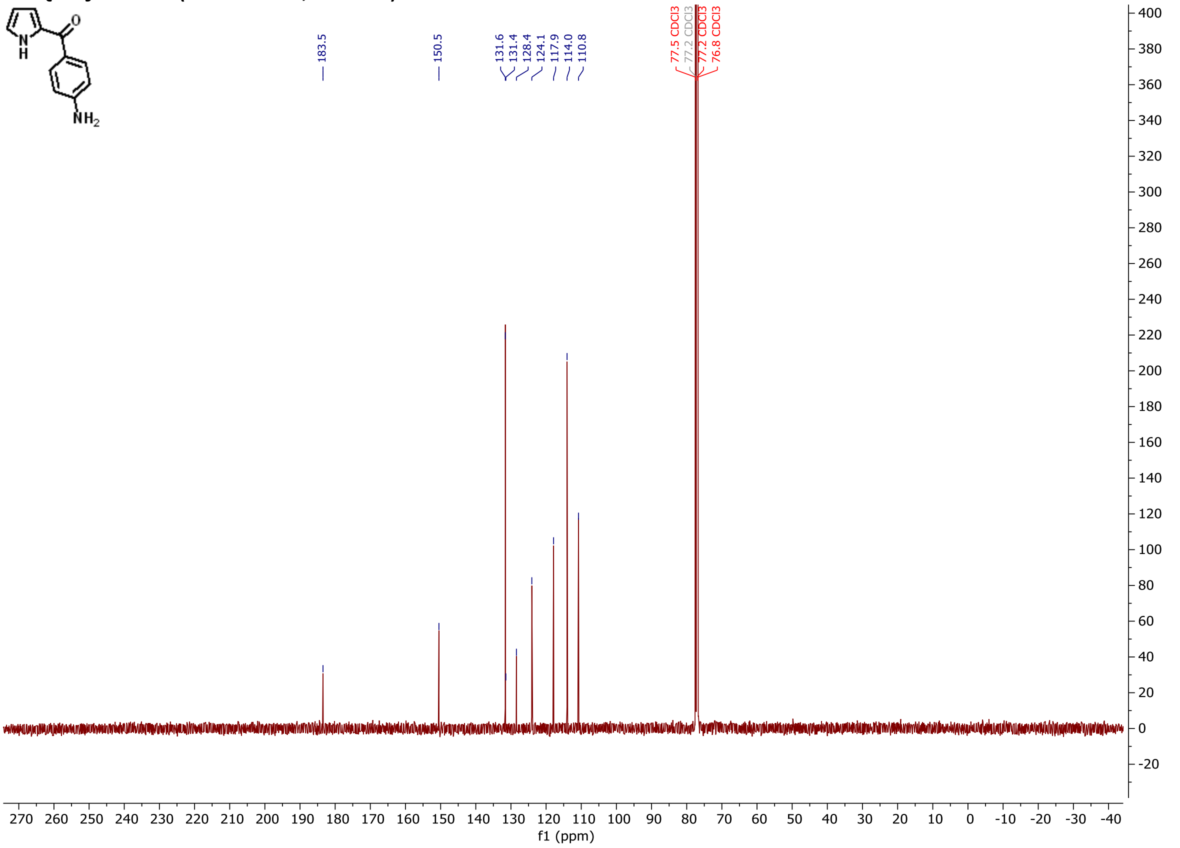

6-nitro-9H-pyrrolo[1,2-a]indol-9-one (9)

^{1}H NMR (400 MHz, $CDCl_3$)

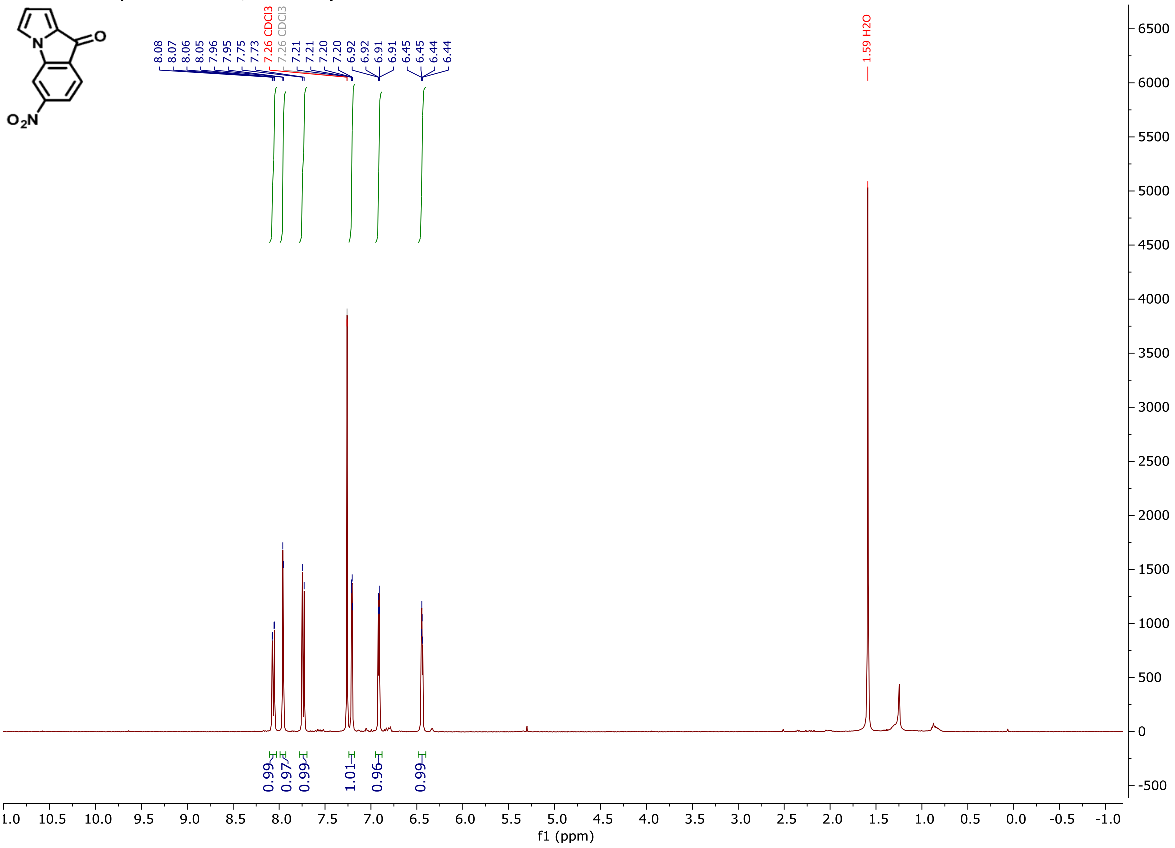

^{13}C{^{1}H} NMR (101 MHz, $CDCl_3$)

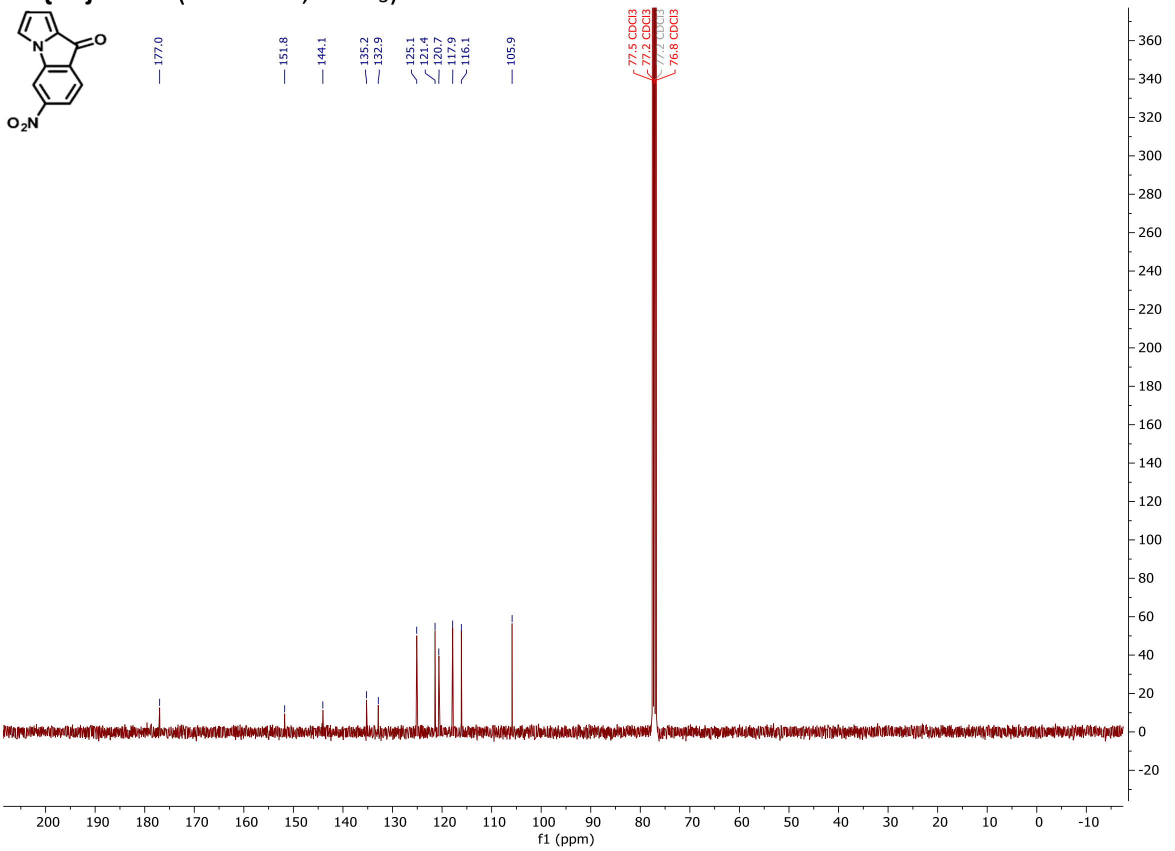

8. References

(1) Kerr, M. S.; Read de Alaniz, J.; Rovis, T. An efficient synthesis of achiral and chiral 1, 2, 4-triazolium salts: bench stable precursors for N-heterocyclic carbenes. *J. Org. Chem.* **2005**, *70*, 5725-5728.

(2) Meng, Q. Y.; Döben, N.; Studer, A. Cooperative NHC and Photoredox Catalysis for the Synthesis of β-Trifluoromethylated Alkyl Aryl Ketones. *Angew. Chem. Int. Ed.* **2020**, *59*, 19956-19960.

(3) Piel, I.; Pawelczyk, M. D.; Hirano, K.; Fröhlich, R.; Glorius, F. A Family of Thiazolium Salt Derived N-Heterocyclic Carbenes (NHCs) for Organocatalysis: Synthesis, Investigation and Application in Cross-Benzoin Condensation. *Eur. J. Org. Chem.* **2011**.

(4) Arduengo Iii, A. J.; Krafczyk, R.; Schmutzler, R.; Craig, H. A.; Goerlich, J. R.; Marshall, W. J.; Unverzagt, M. Imidazolylidenes, imidazolinylidenes and imidazolidines. *Tetrahedron.* **1999**, *55*, 14523-14534.

(5) Liu, F.; Bugaut, X.; Schedler, M.; Fröhlich, R.; Glorius, F. Designing N-Heterocyclic Carbenes: Simultaneous Enhancement of Reactivity and Enantioselectivity in the Asymmetric Hydroacylation of Cyclopropenes. *Angew. Chem. Int. Ed.* **2011**, *50*, 12626-12630.

(6) Zaghdane, H.; Boyd, M.; Colucci, J.; Simard, D.; Berthelette, C.; Leblanc, Y.; Wang, Z.; Houle, R.; Lévesque, J. F.; Molinaro, C. New indole amide derivatives as potent CRTH2 receptor antagonists. *Bioorganic Med. Chem. Lett.* **2011**, *21*, 3471-3474.

(7) Sergeev, M. E.; Pronin, V. B.; Voyushina, T. L. Procedure for the oxidation of β-amino alcohols to α-amino aldehydes. *Synlett.* **2005**, *2005*, 2802-2804.

(8) Carpita, A.; Ribecai, A.; Stabile, P. Microwave-assisted synthesis of indole-and azaindole-derivatives in water via cycloisomerization of 2-alkynylanilines and alkynylpyridinamines promoted by amines or catalytic amounts of neutral or basic salts. *Tetrahedron.* **2010**, *66*, 7169-7178.

(9) An, J.; Chang, N.-J.; Song, L.-D.; Jin, Y.-Q.; Ma, Y.; Chen, J.-R.; Xiao, W.-J. Efficient and general synthesis of oxazino [4, 3-a] indoles by cascade addition-cyclization reactions of (1 H-indol-2-yl) methanols and vinyl sulfonium salts. *Chem. Commun.* **2011**, *47*, 1869-1871.

(10) Liu, Y.; Luo, G.; Yang, X.; Jiang, S.; Xue, W.; Chi, Y. R.; Jin, Z. Carbene-Catalyzed Enantioselective Aromatic N-Nucleophilic Addition of Heteroarenes to Ketones. *Angew. Chem. Int. Ed.* **2020**, *59*, 442-448.

(11) Okuro, K.; Gurnham, J.; Alper, H. Ionic diamine rhodium complex catalyzed reductive N-heterocyclization of 2-nitrovinylarenes. *J. Org. Chem.* **2011**, *76*, 4715-4720.

(12) Wilson, R. M.; Hengge, A. Nucleophilic additions to triazolinedione ylides, extremely reactive carbonyl equivalents: a new class of condensation reactions. *J. Org. Chem.* **1987**, *52*, 2699-2707.

(13) Narayana, B.; Ashalatha, B. V.; Raj, K. K. V. Simple Syntheses Of 5-Fluoro/Chloro/Bromoindole-2-Methanols And 5-Fluoro/Chloro/Bromoindole-2-Aldehydes. *Org. Chem. Ind. J.* **2006**, *2*, 5-9.

(14) Tan, Y. J.; Li, M.; Gunawan, G. A.; Nyantakyi, S. A.; Dick, T.; Go, M.-L.; Lam, Y. Amide–amine replacement in indole-2-carboxamides yields potent mycobactericidal agents with improved water solubility. *ACS Medicinal Chem. Lett.* **2020**, *12*, 704-712.

(15) Pettersson, B.; Hasimbegovic, V.; Bergman, J. One-pot Eschenmoser episulfide contractions in DMSO: Applications to the synthesis of fuligocandins A and B and a number of vinylogous amides. *J. Org. Chem.* **2011**, *76*, 1554-1561.
(16) Roy, K.; Mandal, A. S. Development of linear and nonlinear predictive QSAR models and their external validation using molecular similarity principle for anti-HIV indolyl aryl sulfones. *J. Enzyme Inhib. Med. Chem.* **2008**, *23*, 980-995.
(17) Alcaide, B.; Almendros, P.; Escobar, A.; Herrera, F.; Luna, A. Gold-catalyzed cyclization of Baylis–Hillman adducts derived from formyl-indoles. *The 20th International Electronic Conference on Synthetic Organic Chemistry.* **2016**.
(18) Kothandaraman, P.; Lauw, S. J. L.; Chan, P. W. H. Metal-free synthesis of 1H-indole-2-carbaldehydes by N-iodosuccinimide-mediated cyclization of 1-(2′-anilinyl) prop-2-yn-1-ols in water. A formal synthesis of (R)-calindol. *Tetrahedron.* **2013**, *69*, 7471-7480.
(19) Zhao, Y.; Li, D.; Zhao, L.; Zhang, J. A Practical Synthesis of 2-Aroylindoles from N-(2-Formylphenyl) trifluoroacetamides in PEG-400. *Synthesis.* **2011**, *2011*, 873-880.
(20) Song, B.; Wang, M.; Xu, M.; Kong, L.; Xie, H.; Wang, C.; Li, Y. Controllable synthesis of pyrido [2, 3-b] indol-4-ones or indolo [3, 2-b] quinolines via formal intramolecular C (sp 2)–H functionalization. *Org. Biomol. Chem.* **2019**, *17*, 9960-9965.
(21) Demkiw, K.; Araki, H.; Elliott, E. L.; Franklin, C. L.; Fukuzumi, Y.; Hicks, F.; Hosoi, K.; Hukui, T.; Ishimaru, Y.; O'Brien, E. A nitrogen-assisted one-pot heteroaryl ketone synthesis from carboxylic acids and heteroaryl halides. *J. Org. Chem.* **2016**, *81*, 3447-3456.
(22) Abdel-Moty, S. G.; Abdel-Aal, A. M.; Kafafy, A. N.; El-Shorbagi, A. A. Synthesis of certain 2-aroylindole derivatives of potential analgesic, anti-inflammatory and antipyretic activities. *Bull. Pharm. Sci. Assiut.* **2005**, *28*, 213-223.
(23) Li, X.; Xie, H.; Fu, X.; Liu, J. t.; Wang, H. y.; Xi, B. m.; Liu, P.; Xu, X.; Tang, W. Rhodium (I)-Catalyzed Benzannulation of Heteroaryl Propargylic Esters: Synthesis of Indoles and Related Heterocycles. *Chem. Eur. J.* **2016**, *22*, 10410-10414.
(24) Paulvannan, K. An Atom-Economical Approach to Conformationally Constrained Tricyclic Nitrogen Heterocycles via Sequential and Tandem Ugi/Intramolecular Diels− Alder Reaction of Pyrrole1, 2. *J. Org. Chem.* **2004**, *69*, 1207-1214.
(25) Jeon, K. O.; Jun, J. H.; Yu, J. S.; Lee, C. K. Infrared and nuclear magnetic resonance properties of benzoyl derivatives of five-membered monoheterocycles and determination of aromaticity indices. *J. Heterocycl. Chem.* **2003**, *40* (5), 763-771.
(26) Llopart, C. C.; Joule, J. A. Synthetic studies related to the akuammiline alkaloids. *Arkivoc.* **2004**, *10*, 20-38.
(27) Labadie, S. S.; Teng, E. Indol-2-yltributylstannane: a versatile reagent for 2-substituted indoles. *J. Org. Chem.* **1994**, *59*, 4250-4254.
(28) Taylor, J. E.; Jones, M. D.; Williams, J. M. J.; Bull, S. D. Friedel− Crafts Acylation of Pyrroles and Indoles using 1, 5-Diazabicyclo [4.3. 0] non-5-ene (DBN) as a Nucleophilic Catalyst. *Org. Lett.* **2010**, *12*, 5740-5743.
